# Predicting drug-target interactions from drug structure and protein sequence using novel convolutional neural networks

**DOI:** 10.1186/s12859-019-3263-x

**Published:** 2019-12-24

**Authors:** ShanShan Hu, Chenglin Zhang, Peng Chen, Pengying Gu, Jun Zhang, Bing Wang

**Affiliations:** 1 School of Computer Science and Technology, Anhui University, Jiulong Road, Hefei, 230601 China; 2 Institutes of Physical Science and Information Technology, Anhui University, Jiulong Road, Hefei, 230601 China; 3 Cadre’s Ward (South District), The First Affiliated Hospital of USTC, Division of Life Sciences and Medicine, University of Science and Technology of China, Hefei, 230001 China; 4 School of Electrical and Information Engineering, Anhui University of Technology, Ma’anshan, 243032 China; 5 School of Electrical and Information Engineering, Anhui University, Hefei, 230601 China

**Keywords:** Drug-target interactions, CNN, Protein sequence, Ensemble method

## Abstract

**Background:**

Accurate identification of potential interactions between drugs and protein targets is a critical step to accelerate drug discovery. Despite many relative experimental researches have been done in the past decades, detecting drug-target interactions (DTIs) remains to be extremely resource-intensive and time-consuming. Therefore, many computational approaches have been developed for predicting drug-target associations on a large scale.

**Results:**

In this paper, we proposed an deep learning-based method to predict DTIs only using the information of drug structures and protein sequences. The final results showed that our method can achieve good performance with the accuracies up to 92.0%, 90.0%, 92.0% and 90.7% for the target families of enzymes, ion channels, GPCRs and nuclear receptors of our created dataset, respectively. Another dataset derived from DrugBank was used to further assess the generalization of the model, which yielded an accuracy of 0.9015 and an AUC value of 0.9557.

**Conclusion:**

It was elucidated that our model shows improved performance in comparison with other state-of-the-art computational methods on the common benchmark datasets. Experimental results demonstrated that our model successfully extracted more nuanced yet useful features, and therefore can be used as a practical tool to discover new drugs.

**Availability:**

http://deeplearner.ahu.edu.cn/web/CnnDTI.htm.

## Introduction

Exploring a chemical compound which selectively binds to potential target is a highly challenging and extremely expensive work in drug development process. Only small amount of candidate chemical molecules have been identified to be approved drugs, while massive compounds still have unknown interaction profiles with proteins [1–3]. The identification of drug-target interactions (DTIs) is imperative for new drugs, as this would aid to narrow down the amount of prospective drug candidates and detect side effects in advance [4]. Detecting such interactions between drugs and targets also provides insights into experimental design of drug discovery [5].

In the past decades, in silico approaches have been regularly developed to complement drug discovery research, which are capable of speeding up the experimental wet lab research and reduce tedious and laborious work [6–9]. These approaches play a vital role in discriminating potential associations between drugs and targets, which provides a clue to uncover the underlying functions of many classes of pharmaceutically useful protein targets including enzymes, ion channels, G protein-coupled receptors(GPCRs) and nuclear receptors [10].

Significant computational methods have been developed to predict DTIs, which are mainly categorized into three strategies: ligand-based approaches [11], docking approaches[12, 13] and chemogenomic approaches. The prerequisite of docking methods is the available 3D structure of a drug or a protein; while ligand-based methods often produce unreliable results when the number of known binding ligands of a target protein is insufficient [14]. Thus, many studies have focused on chemogenomic methods, which can be used to yield successful predictions of DTIs on widely abundant biological data.

Most of chemogenomic methods are based on a key assumption that similar drugs may bind to similar targets, and vice versa [15]. For example, Chen et al. [16] proposed an ensemble system to predict protein ligand binding sites in DTI. Yamanishi et al. [17] developed a new unified framework integrated with chemical, genomic and pharmacological spaces to increase research productivity toward genomic drug discovery. It was elucidated that pharmacological effect similarity is more important than chemical structures similarity in the prediction of unknown DTIs. Keiser et al. [18] used a chemical similarity method following the underlying assumption that similar drugs usually interact with similar protein targets for each drug-protein connection. However, limited known small molecules and different protein families render it unsuitable for large-scale applications. A semi-supervised learning approach, a new kernel from known drug-target interaction network based on the standard Laplacian regularized least square [19], exploited not only small amount of labeled data but also sufficient unlabeled data in order to obtain the maximum generalization ability from heterogeneous biological spaces. A systematic approach based on both Random Forest (RF) and Support Vector Machine (SVM) classifier took into accounts the structural and physicochemical properties of proteins derived from primary sequences of proteins, which was a robust and efficient tool to distinguish the novel scaffold hopping ligands of the receptors [20].

It is well known that traditional machine learning approaches have achieved remarkably successful applications in different fields, but at the expense of manually selected and tuned features [21]. Deep learning techniques have attracted growing attention for the ability of automatically learning informative features. The reason for this is in that deep learning method simplifies the progress of manual feature selection and outperforms other competitive methods. In recent years, deep learning has been a promising and attractive tool for dealing with large, high-dimensional, and complex biological and chemical data. A multi-scale feature deep representations (MFDR) inferring interactions firstly reconstructed drug and protein features with low-dimensional vectors by Auto-Encoders and then these features was used to train a prediction model by SVM [22]. Wen et al. [23] developed a model termed as DeepDTIs to accurately identify drug-target associations. It was the first time to exploit deep-belief network to automatically extract meaningful features from simple chemical substructures and sequence order information.

In this paper, we proposed an deep-learning-based predictive model to discriminate potential associations between drugs and target proteins. The features of a drug-target pair were characterized as two parts. One consists of target descriptors that are encoded by amino acid physicochemical properties extracted from AAindex1 database. The other consists of drug descriptors that are computed by a PaDEL-Descriptor toolbox. The concatenated vectors of pairs of drugs and targets were projected into a 784-dimensional subspace by random projection methodology and sequently they were reshaped into a 28×28 matrix. Thereafter the predictive deep-learning-based model was built using different types of image-like matrices generated by random matrices. The final results were the ensemble of several predictors by majority voting technique on the same drug-target pairs. The method was evaluated by several different benchmark datasets and showed significant performance.

## Methods

### Datasets

In this work, potential interactions between drugs and target proteins were investigated on three benchmark datasets. Two datasets were derived from KEGG DRUG database while the other one was built from DrugBank database (http://www.drugbank.ca/). KEGG DRUG captures abundant approved drugs in Japan, USA and Europe based on the chemical structure and molecular interaction network information, of which most drugs reported corresponding the information of target proteins [24]. While DrugBank offers an appealing freely available resource to the public, including 2555 approved small molecule drugs and 5121 detailed non-redundant sequences of target proteins [25].

The first dataset is provided from reference [26], called as *Dataset1*. In *Dataset1*, 4797 drug-target pairs were regarded as positive samples, where are 2719 pairs for enzymes, 1372 for ion channels, 630 for GPCRs and 86 for nuclear receptors. The corresponding negative samples were generated by random selection. The detailed progress is described in the following steps: (i) re-coupling all drugs and targets in the benchmark dataset into pairs after removing those known drug-target interactions in the positive samples. (ii) randomly selecting negative samples until the number of negative samples reached exactly two times as many as that of positive samples.

**Fig. 1:**
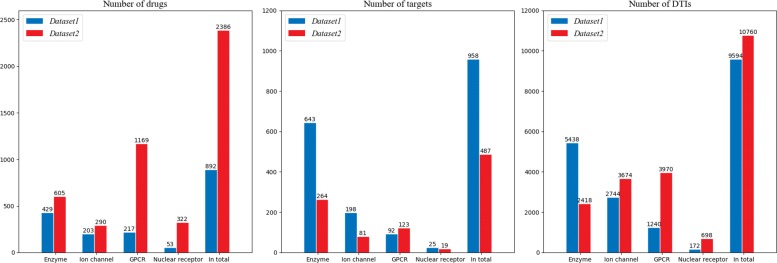
Data statistics. The distribution of the numbers of drugs, targets and drug-target pairs on two benchmark datasets (*Dataset1* and *Dataset2*)

The second dataset of DTIs, called *Dataset2*, was manually collected. Protein kinases were integrated into enzymes in the database. Besides, drugs without structural information and target proteins without primary sequence were discarded in the dataset. The drug-target pairs in *Dataset2* which is redundant and overlapping with *Dataset1* were also omitted. As like *Dataset1*, the number of corresponding negative samples in *Dataset2* is twice as many as positive samples. Ultimately, 16140 drug-target pairs were obtained in *Dataset2*, where 3627 for enzymes, 5511 for ion channels, 5955 for GPCRs and 1047 for nuclear receptors. Figure 1 illustrates the number of drugs, target proteins as well as drug-target pairs on both *Dataset1* and *Dataset2*. The detailed information can be referred to Additional file 1.

The last dataset is from reference [23], called *Dataset3*, where inorganic compounds or very small molecule compounds were omitted. The dataset consists of 6262 positive samples, and negative samples with the same number as that of positive samples which were generated by random selection. Thus, 12524 potential drug-target pairs were used in this work.

### Drug representations

Molecular descriptor is indeed a series of fixed-length number to represent the effective chemical information encoded within a symbolic representation of small molecule [27]. Currently, it has been routinely applied in cheminformatics area such as QSAR analysis, virtual screening and drug ADME/T prediction, as well as other drug discovery processes. PaDEL-Descriptor is an appealing graphical user interface (GUI) toolbox using Java language to calculate the descriptors of chemical small molecules, which can work on different platforms. It currently involves 1444-dimensional 1D, 2D descriptors, 134-dimensional 3D descriptors and 10 kinds of fingerprints [28]. In this study, 1444-dimensional 1D and 2D descriptors were employed to represent the drug candidates, which can be formulated as *D*=[*D*_1_,*D*_2_,*D*_3_,…,*D*_1444_].

### Protein representations

For predicting DTIs, the sequences of target proteins are encoded by different physicochemical properties of amino acids. In order to improve the final predictive performance, 34 properties were extracted from AAindex1 database with the correlation coefficient less than 0.5 [29]. In this process, the correlation coefficients between two properties were calculated and ranked in order. Then, for each property, the number of correlation coefficients more than 0.5 between the property and the other properties was recorded. These properties were ranked in descend order. Beginning from the top property to the lowest one, other properties having correlation coefficient with the beginning property were subsequently removed if the value is more than 0.5. Finally, 34 properties are retained when the process was completed. Protein targets were encoded using these properties by Moran autocorrelation descriptors algorithm [30, 31]. Moran autocorrelation has been widely applied in the prediction of helix contents, and it mainly takes account of the influence of neighboring amino acids around a certain central amino acid[32]. The encoded Moran autocorrelation descriptors of target proteins, called as *T*, is formulated as follows:

1 $$  T(d)=\frac{\frac{1}{N-d}\sum_{i=1}^{N-d}(P_{i}-\overline{P})(P_{i+d}-\overline{P})}{\frac{1}{N}\sum_{i=1}^{N-d}(P_{i}-\overline{P})^{2}}  $$

where *P*_*i*_ and *P*_*i*+*d*_ are property values in one of 34 amino acid properties at sequence positions *i* and *i+d*, respectively; *d* is the distance between the *i*-th residue and neighboring residue; *N* is the length of the protein sequence; $\overline {P}$ is the average value of *P*_*i*_, i.e. $\overline {P}=\left (\sum _{i=1}^{N}P_{i}\right)/N$, and *d* is set as 13 in this work. Therefore, for each of the 34 properties, one protein is represented by vector *T*^*m*^=[*T*_1_,*T*_2_,*T*_3_,…,*T*_13_],*m*=1∼34. Then 34 vectors are concatenated so that target descriptors are characterized by vector *T*_*p*_=[*T*_1_,*T*_2_,*T*_3_,…,*T*_442_].

### Convolutional neural network

An effective deep learning architecture called Convolutional Neural Network (CNN) was widely applied in many areas involving image and video recognition, recommender systems and natural language processing [33]. In addition, a growth number of interesting results has been seen in biomedical applications such as neuronal membrane segmentation and drug discovery. CNN is well-known as feed-forward artificial neural networks inspired by biological processes in that the connectivity pattern between neurons simulates the cognition function of human neural systems [34]. Compared with traditional multilayer perceptron (MLP), the training parameters of CNN are immensely reduced, allowing the network to be deeper with fewer parameters. Thus, CNN can effectively address the problem of vanishing or exploding gradients in the progress of back propagation [35, 36]. A CNN architecture is formed by a stack of distinct layers including convolutional layers, pooling layers and fully connected layers. The convolutional layer represents the core building block of a CNN topology, which is parameterized by a set of learnable filters (or kernels) sliding over a vector or matrix and the result of each filter is called a feature map [37]. Pooling is an operation mostly applied after each convolutional layer, which combines responses at different locations and adds robustness to small spatial variations. Thus, it speeds up the convergence and reduces the amount of computation of neural networks. The outputs of the *l*-th layer and its previous layer are respectively denoted as **V**_*l*_, **V**_*l*−1_, involving only two parts of trainable parameters (i.e. the weight matrix **W**_*l*_ and the bias vector **b**_*l*_). The process can be formulated as:

2 $$  \mathbf{V}_{l}= pool (f(\mathbf{V}_{l-1} \ast \mathbf{W}_{l}+\mathbf{b}_{l})),  $$

where ∗ represents the convolution operation, *pool* denotes the max-pooling operation, and *f*(·) is the activation function.

A dropout layer as a regularization strategy is designed to alleviate the overfitting issue by the means that stochastically adds noise to the hidden layers. The nodes defined as ’dropped out’ do not contribute to the forward pass and do not participate in backpropagation. Fully connection layer usually represents the final layers of a deep neural network topology, of which each neuron is completely connected to all of the nodes in previous and the next layers [38].

**Fig. 2:**
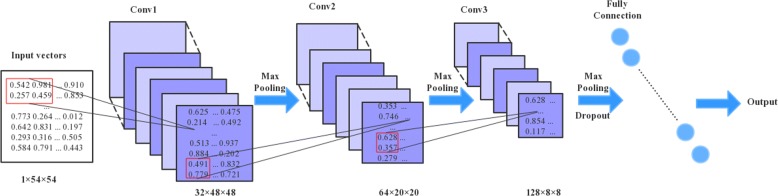
The architecture of our convolutional neural networks. This topology is similar to LeNet-5 networks which contains three convolutional layers, two pooling layers and one fully connected layer

Figure 2 illustrates the CNN-based prediction model, which resembles the LeNet-5 framework, adding only one convolutional layer and one pooling layer. In this work, LeNet-5 is considered as a baseline for the comparison of deep learning algorithms due to containing small amount of parameters.

### The construction of CNN-based model

**Fig. 3:**
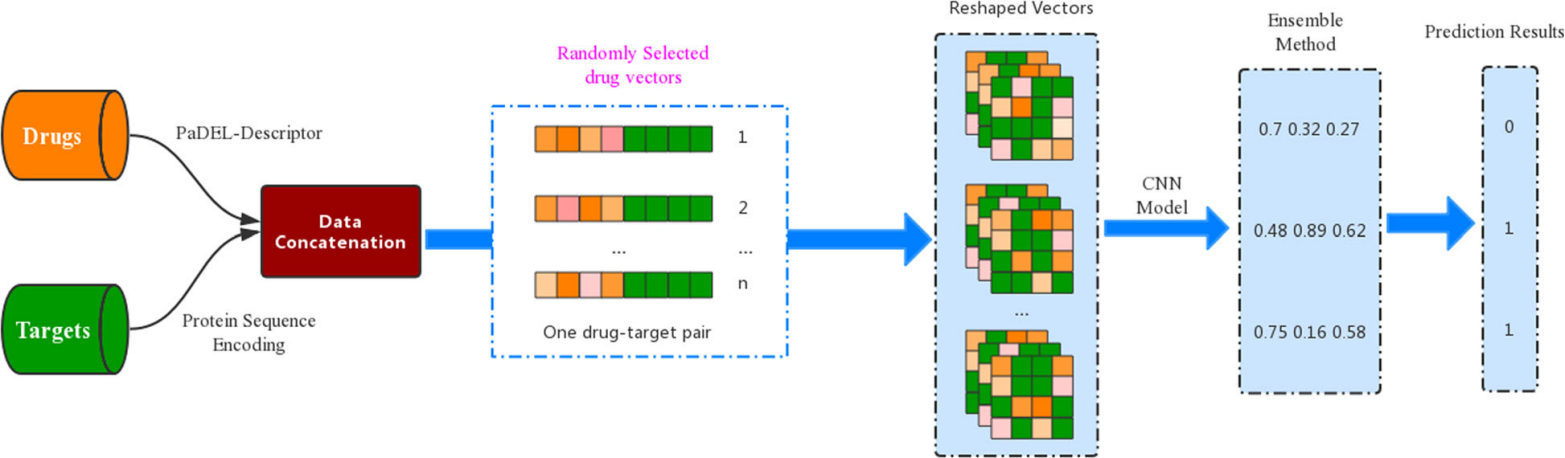
The flowchart of the CNN-based DTI predictions. The final result is represented by 0 or 1 which means non-DTI or DTI

Since small dataset used for deep learning model may reduce the generalization ability of the model, data augmentation schema was adopted. First, let’s see one potential drug-target pair that was represented by [*D*,*T*], where the vectors is simply a concatenation of 342-dimensional drug vectors *D*=[*D*_1_,*D*_2_,*D*_3_,…,*D*_342_] and 442-dimensional vectors of protein descriptors *T*=[*T*_1_,*T*_2_,*T*_3_,…,*T*_442_]. In this way, the input vectors of our training model comprehensively consider the information of small chemical molecules and target proteins. Additionally, drug vectors with almost the same number of target vectors, which decrease the biases caused by the different amount of vectors. So it makes easier and fairer to train an appropriate model to identify DTIs. As shown in Fig. 3, 342-dimensional drug vectors of each drug-target pair (i.e. [*D*,*T*]=[*D*_1_,*D*_2_,*D*_3_,…,*D*_342_,*T*_1_,*T*_2_,*T*_3_,…,*T*_442_]) were generated by random selection. Then the process was repeated *n* times and *n* sets of drug vectors were respectively joined to the 442-dimensional target descriptors. That is to say, one drug-target pair was represented by *n* sets of generated drug-target pair (*V*_*n*_=[*D*_*n*_,*T*]^1×784^, *n*=1,2,…) which involving *n* different randomly selected drug vectors. The progress is terminated until the number of all drug-target pairs is around 40000∼50000. Thus, the *n*-times pairs were regarded as the characterization of one drug-target pair.

After that, the features of each drug-target pair were reshaped into a 28×28 matrix, which is similar to digital image recognition and easily used to train a predictive model through CNN algorithm. The final predictions were yielded by the ensemble of *n*-times pairs’ prediction values. In the ensemble, one drug-target pair is predicted to be interacting if at least half of the *n*-pairs were predicted as positive samples, otherwise it is a non-interaction pair. The construction of our model pipeline is illustrated in Fig. 3.

### Measurement of prediction quality

In this work, four metrics, accuracy (Acc), sensitivity (Sen), precision (Pre) and F1 score (F1), were exploited to evaluate whether the candidate drug and a target protein are interacting. Specifically, F1 score comprehensively measures the rate of sensitivity and precision which is proved to be more credible and objective[39, 40]. Meanwhile, area under the receiver operator characteristic curve (AUC) value is also a common evaluation metric used in machine learning and data mining research to check the ability of a binary classifier system, as its discrimination threshold is varied [41]. The following formulas illustrate the detailed calculation of these metrics.

3 $$ {\begin{aligned} Acc &=\frac{TP+TN}{TP+FP+TN+FN} \\ Pre&=\frac{TP}{TP+FP}\\ Sen&=\frac{TP}{TP+FN} \\ F1&=\frac{2\times Sen\times Pre}{Sen+Pre} \\ \end{aligned}}  $$

In which, TP (True Positive) and TN (True Negative) respectively represent the correctly predicted drug-target interaction pairs and non-interaction pairs. FP (False Positive) means non-interaction pairs predicted as positive samples and FN (False Negative) is that negative instances are wrongly predicted as DTIs pairs.

## Results

### Performance for predictive drug-target interactions

Since target proteins are classified into four pharmaceutically types of drug-target classes, four different predictors using the same parameters were constructed by 10-fold cross-validation to evaluate the performance of our models. That is to say, our dataset was randomly partitioned into 10 disjoint subsets, where one subset is considered as test set while the remaining subsets are regarded as training set. This progress was repeated 10 times until all instances are tested.

First, *Dataset2* was used to train the model to distinguish DTIs. As shown in Table 1, the model achieves accuracies and AUCs for four DTI classes all more than 0.90 on *Dataset2*. The model for enzymes yields the highest performance among the four DTIs classes, with an accuracy of 0.920, a sensitivity of 0.881, a precision of 0.880, an F1 of 0.881 and an AUC value of 0.973. It is noticed that both enzymes and GPCRs classes achieve the highest accuracy values (Acc= 0.920). It is well known that GPCRs are the most difficult cases in the identification of DTIs due to a few of known 3D structure information for GPCRs. That is to say, some unknown or noise features are confused the characterization of GPCR targets. The results indicated that our model has a strong ability to discriminate DTIs on GPCRs.

**Table 1:** The detailed performance for the four protein families on both Dataset1 and Dataset2 by 10-fold cross validation

Type		Acc	Sen	Pre	F1	AUC
Enzymes	*Dataset1*	0.943	0.927	0.903	0.915	0.985
	*Dataset2*	0.920	0.881	0.880	0.881	0.973
Ion channels	*Dataset1*	0.919	0.894	0.867	0.881	0.970
	*Dataset2*	0.900	0.948	0.792	0.863	0.949
GPCRs	*Dataset1*	0.884	0.818	0.831	0.824	0.945
	*Dataset2*	0.920	0.899	0.866	0.882	0.968
Nuclear receptors	*Dataset1*	0.884	0.872	0.798	0.833	0.936
	*Dataset2*	0.907	0.891	0.841	0.865	0.966

Subsequently, *Dataset1* was used to further assess the generalization ability of our model. To fully evaluate the performance of our proposed model, the same parameters and neural network topology were used in corresponding experiments. In consistent with the evaluation on *Dataset2*, the model on the enzymes of *Dataset1* obtains the best performance again with an AUC of 0.985, which demonstrated that our model has an advantage on detecting drug-enzyme interactions. However, the performances on nuclear receptors and GPCRs of Dataset1 are worse than those on *Dataset2*, whose AUC values are respectively falling into 0.936 and 0.945. The possible reason might be that the number of DTIs in nuclear receptors and GPCRs classes is smaller than others, especially for nuclear receptors with only 258 drug-target pairs.

**Fig. 4:**
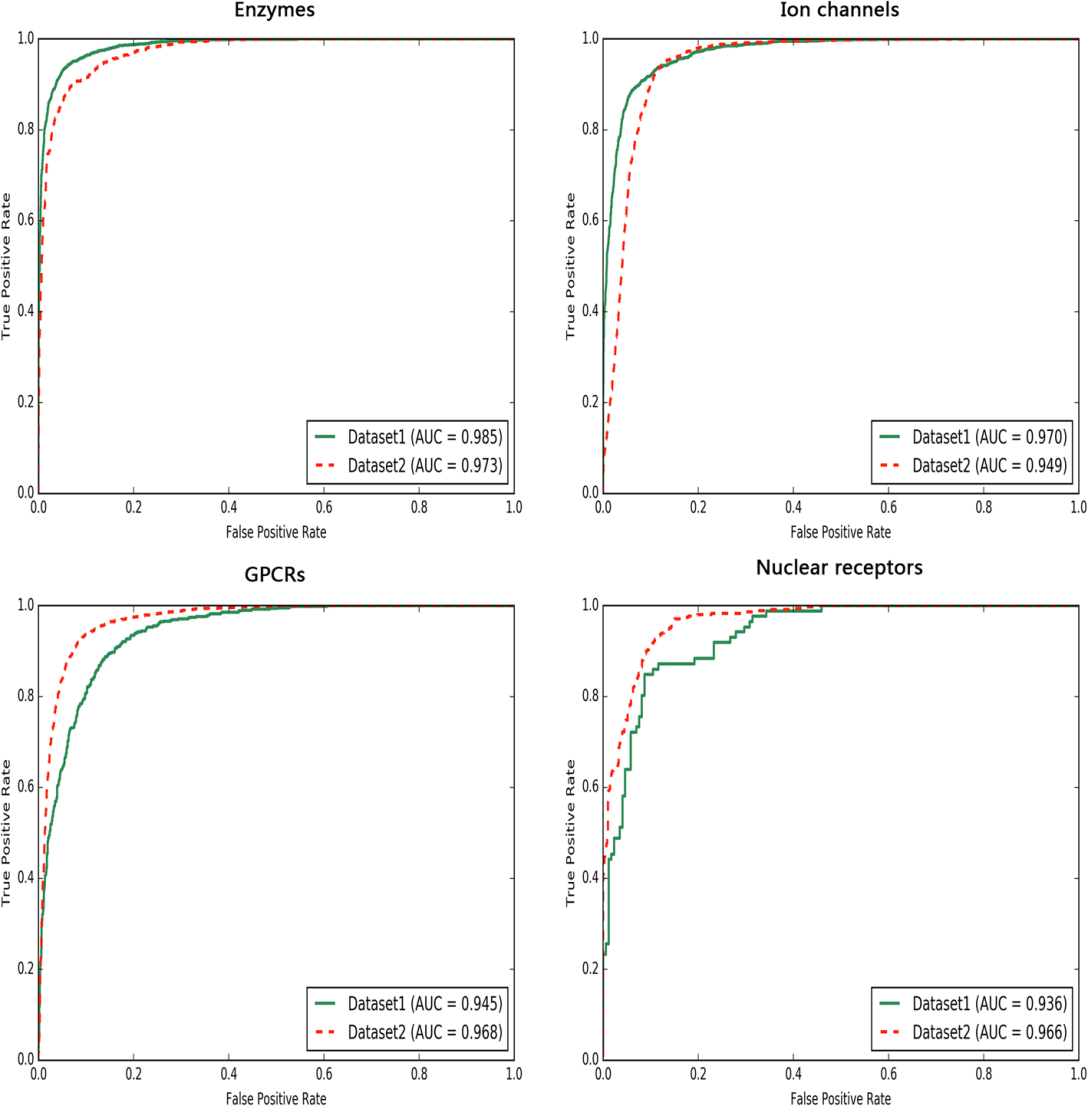
The ROC curves of our model. ROC performance of our method for classes of DTIs: enzymes, ion channels, GPCRs, and nuclear receptors on both *Dataset1* and *Dataset2*

Figure 4 shows ROC curves for the four drug-target interaction classes on both *Dataset1* and *Dataset2*. It indicates that our proposed model can catch sufficient and effective features by deep learning method to detect true drug-target interactions at high true-positive rates against low false-positive rates at any threshold.

### Comparison of other machine-learning-based approaches

In order to further show the robustness and reliability of our method, we made a comparison with the state-of-the-art machine learning methods on the type of GPCRs in *Dataset1*, such as random forests (RF) and k-Nearest Neighbor (KNN) algorithm. For each machine-learning method, all results were obtained with the most suitable hyper-parameters. As displayed in Table 2, our method yields the optimal accuracy, sensitivity, F1 score and AUC value over these machine-learning approaches, although the RF achieves the highest precision of 0.878. However, F1 score is regarded as a more important evaluation metric for DTIs predictions because it measures the balance between precision and sensitivity. The AUC obtained by our method is 0.968, which is respectively 1.5% and 11.2% better than RF and KNN. In summary, under the same dataset, our model outperformed other competitive machine-learning methods, which suggested that deep learning technique is an effective tool to excavate more nuanced features to complete the classification of drug-target interactions.

**Table 2:** Comparison with other machine learning methods on GPCRs classes

Methods	Acc	Sen	Pre	F1	AUC
Our model	**0.920**	0.899	0.866	0.882	**0.968**
KNN	0.830	0.852	0.703	0.770	0.897
Random Forests	0.912	0.856	0.878	0.867	0.958

The highest values are highlighted in bold

### Comparison with other works

Our method was also compared with the following two methods, namely, the work in reference [26] and Zhang’s research [42], by testing the capabilities on *Dataset1*. The comparative results showed that our model outperformed other existing methods in accuracy, even though the accuracy of DrugRPE on nuclear receptors is 2.7% higher than our model (Table 3). It may be most likely due to the limited number of DTIs on nuclear receptors (only 258 samples), so our model cannot be fully trained to reach the optimal prediction performance of the model. For deep learning-based model, large-scale data tends to produce robust and powerful performance.

**Table 3:** Performance comparison in accuracy of our method with two methods on *Dataset1*

Methods	Enzymes	Ion channels	GPCRs	Nuclear receptors
Our method	0.943	**0.919**	0.884	0.884
DrugRPE	0.900	0.890	0.852	0.911
Ref. [26]	0.855	0.808	0.785	0.857

The highest values are highlighted in bold

However, our model achieves the best results on enzymes, ion channels and GPCRs, which is respectively 4.3%, 2.9% and 3.2% higher than DrugRPE. Compared with the reference [26], our model obtains much higher accuracy values on all four protein families.

### Comparison with deep-belief network

More experiments were also investigated to further verify the performance of our method on different datasets. There were many attractive implementations of deep learning technique in various research areas since Hinton et al. [43, 44] first proposed deep-belief network (DBN) which is composed of several simple, unsupervised stacking restricted Boltzmann machines (RBMs), where each subnetwork’s hidden layer serves as the visible layer for the next. Owing to connections between layers but not between units within each hidden layer, DBN can be designed more deeper than traditional artificial neural networks. This prevailing algorithm has gradually provided potent insights in the progression of drug discovery.

At present, convolutional neural network almost has been less applied in the identification of DTIs. Herein, experiments of our model were made on the same dataset (i.e. *Dataset3*) in comparison with reference [23]. So it is another way to verify that convolutional neural network is also an excellent deep learning technique in the discrimination of drug-target associations. To make the comparison fair, the experimental data for the two works are the same as above. The performance comparison is shown in Table 4. In Table 4, our model achieves an accuracy of 90.15% and an AUC of 95.57%, which are 4.30% and 3.99% higher than baseline method, respectively. The noticeable performance improvement of our model further demonstrated that our model has superior ability in DTIs predictions even though databases derived from various resources.

**Table 4:** Overall performance of DBN and our method on *Dataset3*

Methods	TPR	TNR	Acc	AUC
DBN	0.8227	**0.8953**	0.8588	0.9158
Our model	0.9482	0.8673	**0.9015**	**0.9557**

The highest values are highlighted in bold

### The influence of hyper-parameters

Three key hyper-parameters were explored to get the optimal performance for our CNN model, namely neural networks topology, learning rate, and batch normalization layer. In this work, experiments for one hyper-parameter in a range were implemented with fixing other hyper-parameters, and then searched the hyper-parameter for achieving the best performance.

#### Neural networks topology

**Fig. 5:**
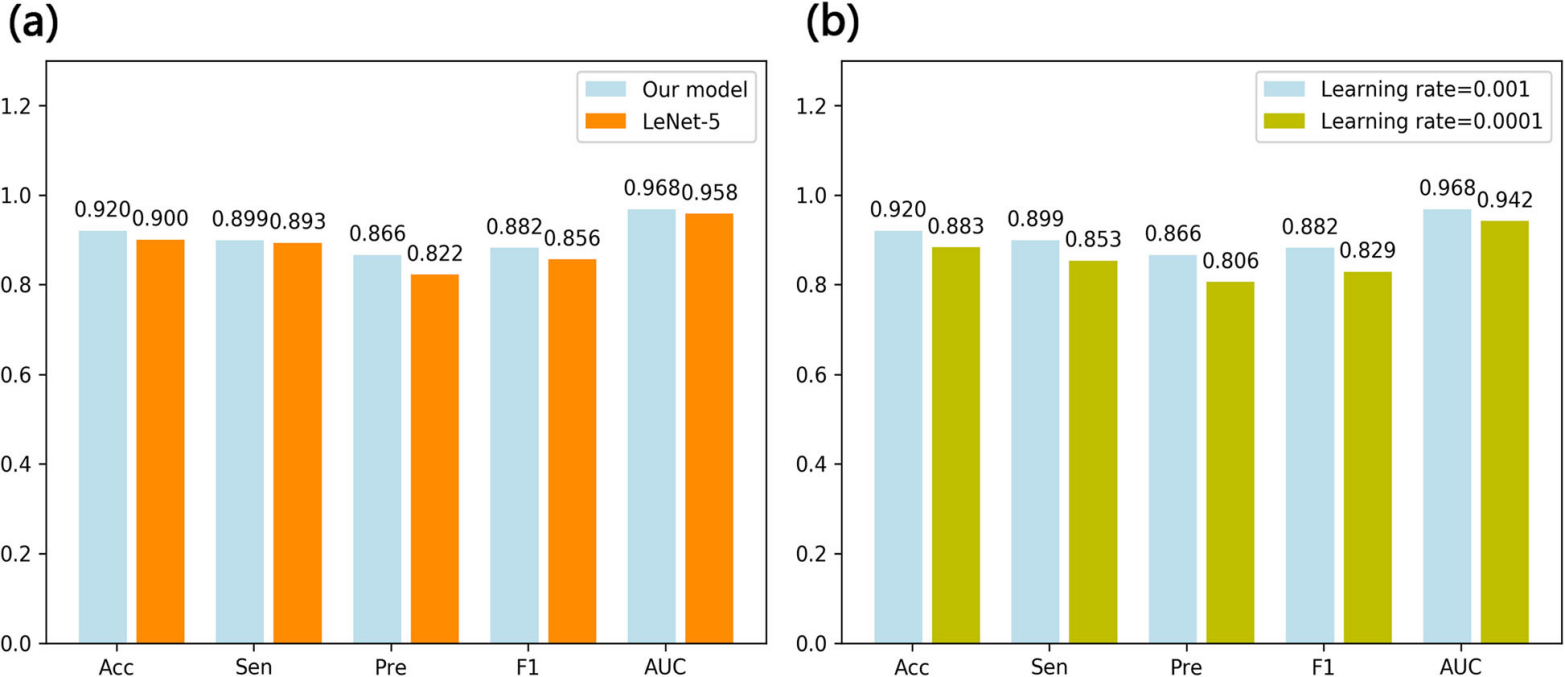
The predictive performance with different hyper-parameters. **a** comparison predictive performance results of our model with LeNet-5 model **b** performance of our model with different learning rates

In this study, LeNet-5 was regarded as the baseline method and compared with our proposed CNN architecture on the same dataset. Indeed, LeNet-5 performed slightly worse (Acc=0.900, Sen=0.893, Pre=0.822, F1=0.856 and AUC=0.958, shown in Fig. 5a) than our model. It indicated that deeper neural network is able to extract more useful information, which makes the accuracy of the model further enhanced.

#### Learning rate

Learning rates(*lr*) of 0.01, 0.001 and 0.0001 were also investigated for CNN in our study. In fact, learning rate greatly influences the final performance of CNN, and it is worth exploring a fit value on different datasets. Large learning rate leads to loss vanish so that valuable features cannot be excavated; while small one results in hardly converging for specific model which takes a long time to train a model and makes the model yielding slightly worse performance. Figure 5b shows the performance comparison of model with different learning rates on the GPCRs family of *Dataset2*. From the Fig. 5b, it is noted that the model with the learning rate of 0.001 performs the best as it achieved an AUC of 2.6% higher than that with the learning rate of 0.0001. When the learning rate is set to 0.01, the loss of the model is emerged as a vacant value, which is why it wasn’t illustrated in this work.

#### Batch normalization layer

Batch normalization (BN) is a crucial trick for achieving successful performance of deep learning methods, which allows for easily training and converging models. Models without BN layer make the output distributions of models inconsistent that leads to a severe increase in error rate. In our experiments, the CNN architecture with BN layer yielded better generalization ability, while that without BN may be ended up with vacant loss. Thus, BN can effectively normalize data into an opposite range so that our model can easily predict input instances to be either interaction or non-interaction.

## Discussion

Motivated by those above results, three common issues which probably affected the final prediction performance of our proposed model were raised and they are then discussed in this section.

### Are loss values reasonable for each protein family?

**Fig. 6:**
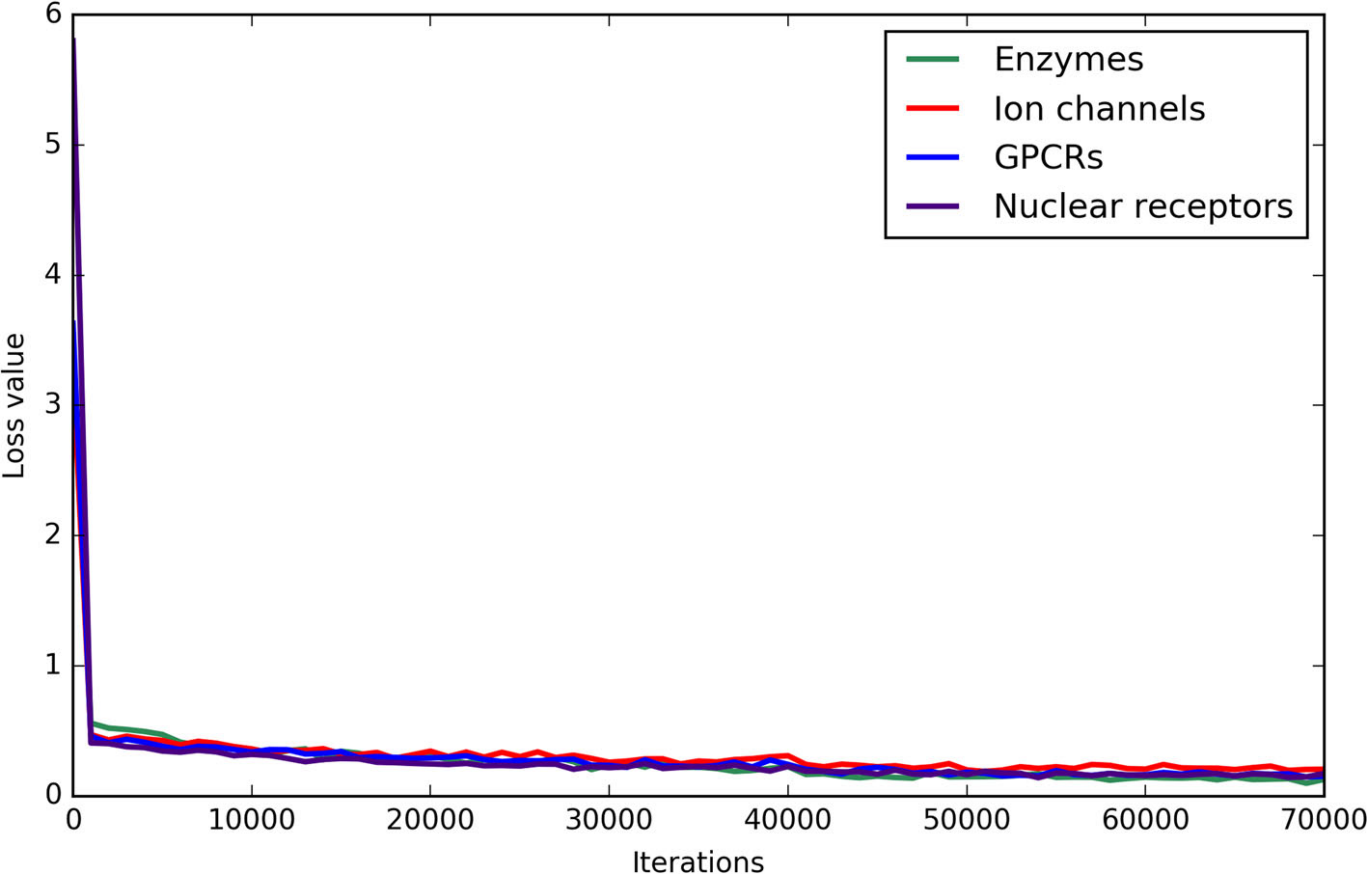
The loss function curves for the four different classes of DTIs

In the training process of CNN, loss value is usually regarded as an essential indicator to inspect the convergence of model, which means the time that our model learns useful features to correctly predict associations between drugs and target proteins. Since the same parameters were used on the four classes of DTIs, their loss tendencies are similar (shown in Fig. 6). For the four classes, the loss values showed the sharpest drop during the first 1000 iterations, which achieved accuracies about 85%. However, afterwards, it decreased slowly with the increase of iterations. At last, loss values fluctuated within a range of 0.1 after 40000 iterations. It demonstrated that our model for the four classes of DTIs has the advantages of strong prediction ability and quick convergence speed.

### Does the final result depend on the amount of non-DTIs pairs?

**Fig. 7:**
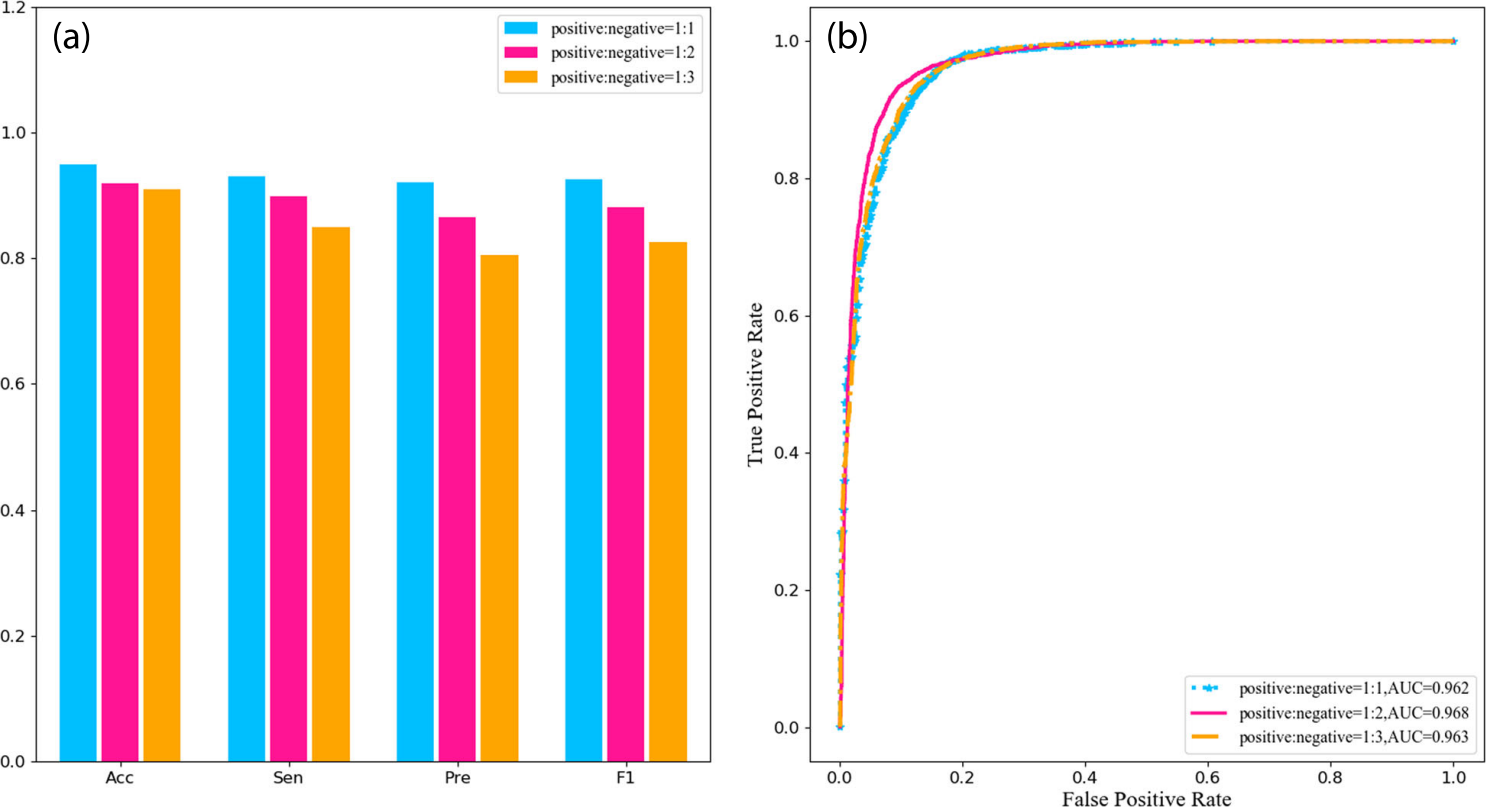
Performance comparison of models with different ratios of positive samples to negative samples on the GPCRs family of *Dataset2*. **a** prediction results for the three different ratios; **b** ROC curves for the three different ratios

In order to investigate whether the final results depend on the number of sampling negative instances, the number of non-DTIs pairs were changed to explore the variance of prediction performance. The results with the three different ratios of positive samples to negative samples (1:1, 1:2 and 1:3) are shown in Fig. 7. The comparison of these three ratios illustrates that the AUC value almost keeps stable with no obvious changes (shown in Fig. 7b). Thus, it proved that our CNN model has a strong and powerful classification ability to discriminate DTIs. However, less number of negative samples represent higher Acc, Sen, Pre and F1, which is more easy to discriminate interactions between drugs and targets (shown in Fig. 7a). Therefore, the final results of our model are fewly concerned with the amount of non-DTIs pairs. It also indicated that randomly selecting experimentally unverified negative instances as non-DTIs pairs makes little influence on the final performance of our model.

### Is learning rate 0.001 optimal for our model?

**Fig. 8:**
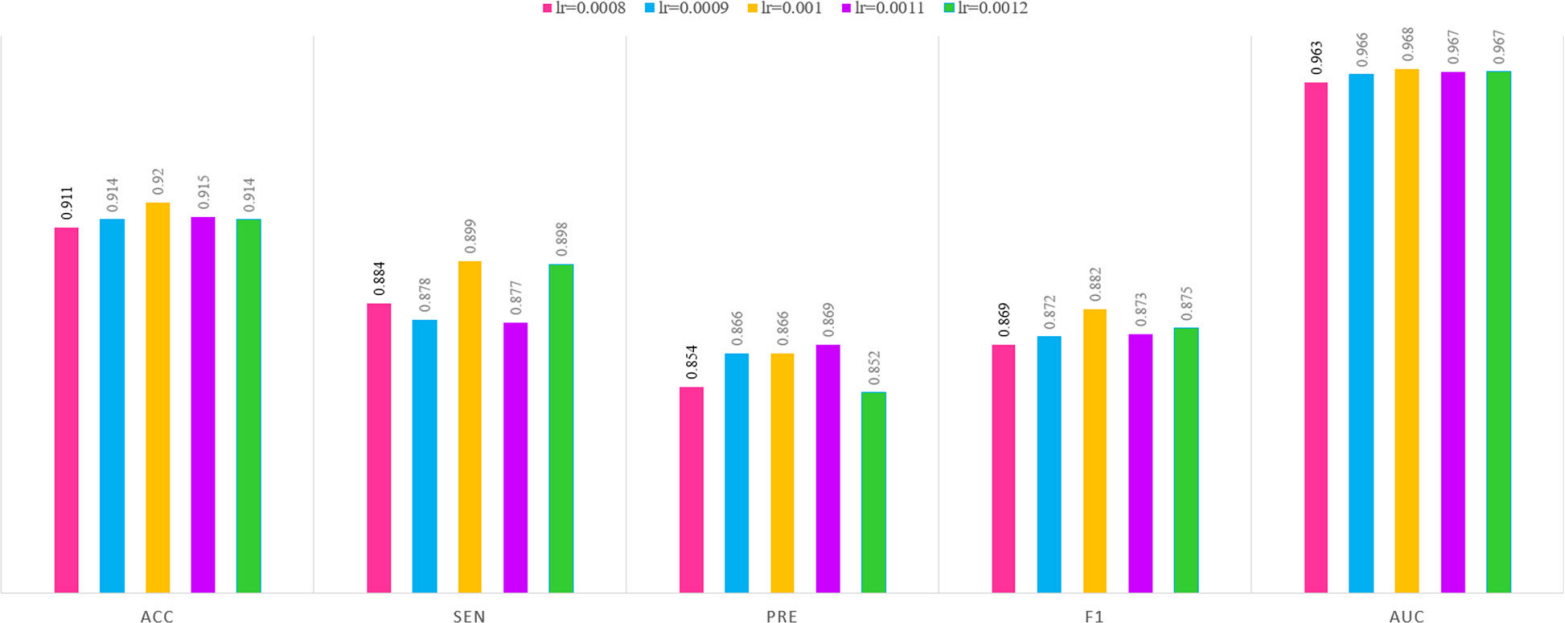
Predictions of our model with learning rates around 0.001 on GPCRs family of *Dataset2*

Although our model performed successful performance on the GPCRs family of *Dataset2* when the learning rate is set to 0.001, the parameter is not fully demonstrated the optimal one for the CNN model. Here, the parameter for the model was investigated in two aspects. First, the learning rate around 0.001 (i.e. lr=0.0008, 0.0009, 0.001, 0.0011 and 0.0012) was explored. As shown in Fig. 8, all parameters yielded nearly similar performance with no significant difference. When the learning rate is 0.001, our model is more benefited on the GPCRs family of *Dataset2* with slightly higher Acc, Sen, F1 and AUC values. But generally speaking, small range of learning rate values makes few influence on the final results for our model. Subsequently, a series of learning rate as above were explored on the other protein families of both *Dataset1* and *Dataset2* (shown in Additional file 2). Not all protein families performed the best when the learning rate is 0.001, that is, 6 (except for enzymes and nuclear receptors of *Dataset2*) of 8 protein families yielded the optimal performance. By the comparison of all types of proteins on two datasets, the learning rate 0.001 is still considered as the optimal one for the CNN model.

## Case study

**Fig. 9:**
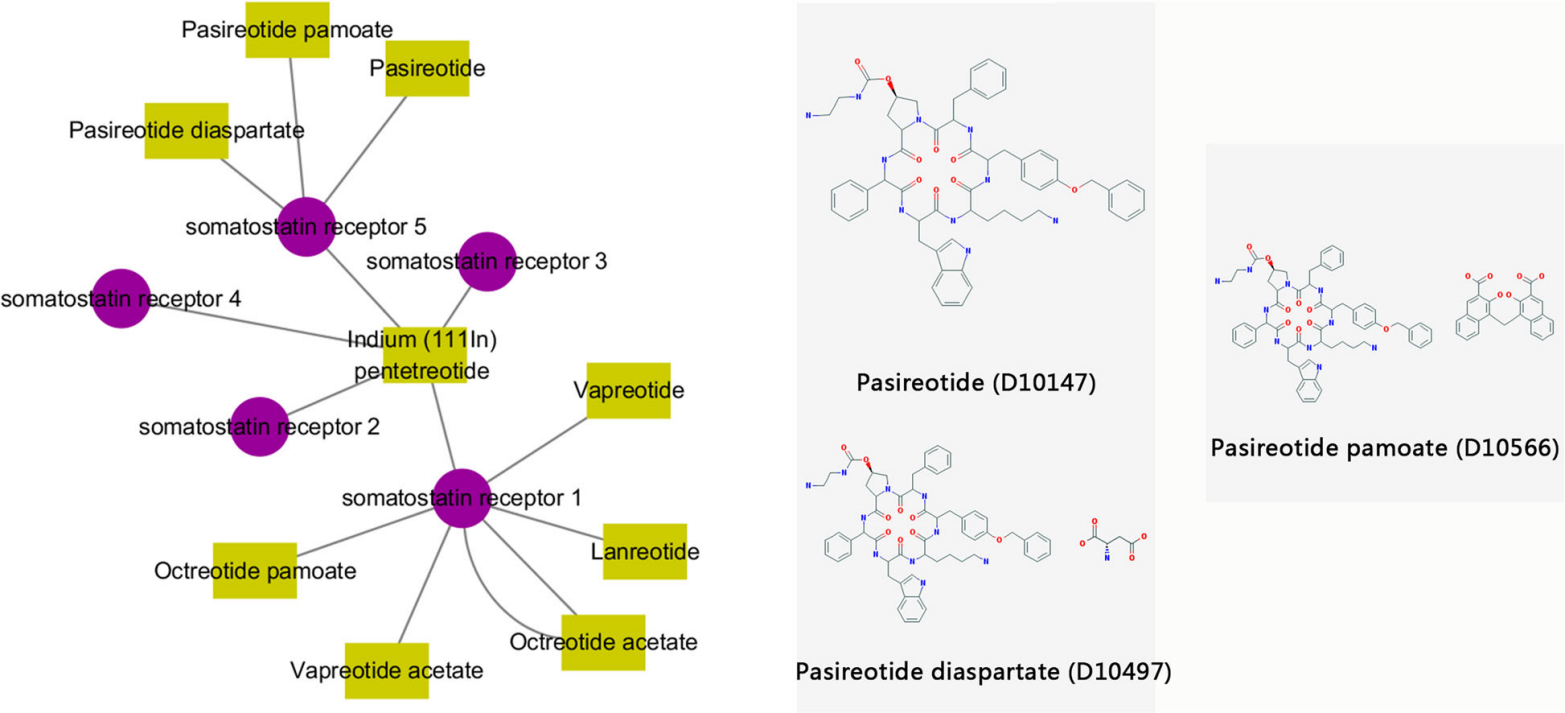
One case of predicted DTIs. Visualization of the predicted interaction sub-network between drugs and GPCRs, where the purple circles and yellow boxes indicate the targets and drugs, respectively; the gray edges indicate the correctly predicted DTIs

We investigated several distinct interactions between 9 drugs and 5 targets on *Dataset2*. As depicted in Fig. 9, the drug-target network contains 14 interactions, in which 5 targets belong to the same subtype of GPCRs, i.e., somatostatin receptor, which is a type of hypothalamic hormone. All of these target proteins participate in the pathway of neuroactive ligand-receptor interaction, where 3 targets (somatostatin receptor 1, somatostatin receptor 2 and somatostatin receptor 5; i.e., hsa:6751, hsa:6752 and hsa:6755) are crucial for cAMP signaling pathway. The drugs are roughly categorized as four classes: vapreotide, pasireotide, octreotide and Indium (111In) pentetreotide. The 2D structures of three drugs (D10147, D10566, D10497) interacting with target hsa:6755 are shown in Fig. 9, which clearly reflects highly structural similarity. Furthermore, the similarity score between D10497 and D10147 operated by SIMCOMP is 0.8400. It’s also demonstrated the hypothesis of similar drugs binding to similar targets. In our study, these DTIs are all correctly predicted using our proposed method, which provides a clue for seeking massive similar chemical molecules binding to a specific target. Thus, our model still has the advantage on discriminating the case of drug-target associations where multiple target proteins interacting with multiple chemical molecules. It will be a helpful tool in the application of *drug repositioning* or *drug repurposing*.

## Conclusion

Since experimental identification of drug-target interactions is laborious and time-consuming, the in silico determination of interactions between drugs and target proteins has become a significant step in the drug discovery process for detecting new drugs or novel targets of existing drugs [45]. In this work, we proposed an intuitive powerful CNN-based classifier that utilized only 1D, 2D structural descriptors of drugs and sequences of proteins as the information of DTIs to discriminate true interaction pairs. A part of drug descriptors by randomly selection and all target proteins encoded descriptors were concatenated and reshaped into an image-like matrix as input vectors to characterize one drug-target pair. The whole model was trained by CNN algorithm which achieved satisfactory predictions than other baseline methods for the identification of drug-target interactions on three different benchmark datasets. Therefore, we showed that our established CNN architecture is capable to capture more potent and informative features among massive features. We envisioned that deep learning technique will be a prevailing algorithm in a wide range of drug research areas to discriminate possible associations between drugs and target proteins.

## Supplementary information

**Additional file 1** This file records the detailed drug-target pairs on enzymes, ion channels, GPCRs and nuclear receptors of the *Dataset2*.

**Additional file 2** In this file, to obtain the optimal learning rate of our model, a series of different learning rates are explored on each types of protein families of both Dataset1 and Dataset2.

## Data Availability

Not applicable.

## Abbreviations

ACC: Accuracy
AUC: Area Under the Curve
BN: Batch normalization
CNN: Convolutional Neural Network
DTIs: Drug-target interactions
DBN: Deep-belief network
F1: F1 score
FN: False negative
FP: False positive
GPCRs: G protein-coupled receptors
GUI: Graphical user interface
KNN: k-Nearest Neighbor
MFDR: Multi-scale feature deep representations
MLP: Multilayer perceptron
Pre: Precision
RBMs: Boltzmann machines
RF: Random Forest
SVM: Support vector machine
TN: True negative
TP: True positive
