# Supplementary material for: Predicting drug-target interactions from drug structure and protein sequence using novel convolutional neural networks
Source: BMC Bioinformatics. 2019 Dec 24;20(Suppl 25):689. doi: 10.1186/s12859-019-3263-x (PMC6929541; doi:10.1186/s12859-019-3263-x)
Supplement: Supplementary file 1 — Additional file 1 This file records the detailed drug-target pairs on enzymes, ion channels, GPCRs and nuclear receptors of the Dataset2. [file 12859_2019_3263_MOESM1_ESM.docx]

**Additional file 1: Drug-target interaction pairs on *Dataset2***

The detailed drug-target interaction system for enzymes, ion channels, GPCRs and nuclear receptors on benchmark *Dataset2* were shown in Table I, II, III and IV, respectively. It contains 16140 drug-target pairs in total, of which 3627 for enzymes, 5511 for ion channels, 5955 for GPCRs, and 1047 for nuclear receptors. In each table, the column named class is the label indicates the pair with 1 for positive samples and 0 for negative samples. All detailed information about these interactions can be found in KEGG BRITE database. In addition, our data is an independent dataset which not contain redundant information from reference (He, Zhisong, et al. "Predicting Drug-Target Interaction Networks Based on Functional Groups and Biological Features." *Plos One* 5.3 (2010): e9603.) (in our study called *Dataset1*).

Table I The overall of drug-target interactions for enzymes class on *Dataset2*

| **class** | **Target** | **Drug** | **class** | **Target** | **Drug** | **class** | **Target** | **Drug** |
| --- | --- | --- | --- | --- | --- | --- | --- | --- |
| 1 | hsa_983 | D09604 | 0 | hsa_983 | D08688 | 0 | hsa_47 | D10450 |
| 1 | hsa_9759 | D06637 | 0 | hsa_983 | D03208 | 0 | hsa_47 | D05900 |
| 1 | hsa_9759 | D08870 | 0 | hsa_983 | D08131 | 0 | hsa_47 | D02218 |
| 1 | hsa_9759 | D10019 | 0 | hsa_983 | D08102 | 0 | hsa_47 | D10431 |
| 1 | hsa_9759 | D10060 | 0 | hsa_983 | D09730 | 0 | hsa_4322 | D08878 |
| 1 | hsa_9759 | D10084 | 0 | hsa_983 | D10229 | 0 | hsa_4322 | D01896 |
| 1 | hsa_9759 | D10319 | 0 | hsa_983 | D08363 | 0 | hsa_4322 | D10641 |
| 1 | hsa_9734 | D06637 | 0 | hsa_983 | D07439 | 0 | hsa_4322 | D07080 |
| 1 | hsa_9734 | D08870 | 0 | hsa_983 | D08344 | 0 | hsa_4322 | D00718 |
| 1 | hsa_9734 | D10019 | 0 | hsa_9759 | D09750 | 0 | hsa_4322 | D05096 |
| 1 | hsa_9734 | D10060 | 0 | hsa_9759 | D07080 | 0 | hsa_4322 | D10091 |
| 1 | hsa_9734 | D10084 | 0 | hsa_9759 | D08556 | 0 | hsa_4322 | D09334 |
| 1 | hsa_9734 | D10319 | 0 | hsa_9759 | D09750 | 0 | hsa_4322 | D09546 |
| 1 | hsa_9475 | D01840 | 0 | hsa_9759 | D07941 | 0 | hsa_4313 | D05728 |
| 1 | hsa_9475 | D03115 | 0 | hsa_9759 | D10529 | 0 | hsa_4313 | D07969 |
| 1 | hsa_9475 | D07941 | 0 | hsa_9759 | D03099 | 0 | hsa_4313 | D09640 |
| 1 | hsa_9475 | D10737 | 0 | hsa_9759 | D00181 | 0 | hsa_4313 | D01683 |
| 1 | hsa_9475 | D10738 | 0 | hsa_9759 | D03378 | 0 | hsa_4313 | D08953 |
| 1 | hsa_9212 | D08279 | 0 | hsa_9759 | D10635 | 0 | hsa_4313 | D03938 |
| 1 | hsa_9212 | D08344 | 0 | hsa_9759 | D05502 | 0 | hsa_4312 | D08668 |
| 1 | hsa_8974 | D04793 | 0 | hsa_9734 | D09621 | 0 | hsa_4312 | D08238 |
| 1 | hsa_8972 | D09779 | 0 | hsa_9734 | D06068 | 0 | hsa_4312 | D08668 |
| 1 | hsa_8841 | D06637 | 0 | hsa_9734 | D04720 | 0 | hsa_4312 | D08162 |
| 1 | hsa_8841 | D08870 | 0 | hsa_9734 | D07144 | 0 | hsa_4312 | D05215 |
| 1 | hsa_8841 | D10019 | 0 | hsa_9734 | D07983 | 0 | hsa_4312 | D06320 |
| 1 | hsa_8841 | D10060 | 0 | hsa_9734 | D05033 | 0 | hsa_4312 | D10432 |
| 1 | hsa_8841 | D10084 | 0 | hsa_9734 | D02714 | 0 | hsa_4312 | D00112 |
| 1 | hsa_8841 | D10319 | 0 | hsa_9734 | D06578 | 0 | hsa_4311 | D09956 |
| 1 | hsa_8694 | D10657 | 0 | hsa_9734 | D10405 | 0 | hsa_4311 | D10130 |
| 1 | hsa_8694 | D10664 | 0 | hsa_9734 | D08261 | 0 | hsa_4311 | D08463 |
| 1 | hsa_8654 | D03217 | 0 | hsa_9734 | D08073 | 0 | hsa_4311 | D08516 |
| 1 | hsa_8654 | D03260 | 0 | hsa_9475 | D07582 | 0 | hsa_4311 | D07940 |
| 1 | hsa_8654 | D03657 | 0 | hsa_9475 | D04368 | 0 | hsa_4311 | D10365 |
| 1 | hsa_8654 | D08514 | 0 | hsa_9475 | D03786 | 0 | hsa_4311 | D07137 |
| 1 | hsa_8654 | D08668 | 0 | hsa_9475 | D10076 | 0 | hsa_4311 | D07516 |
| 1 | hsa_8654 | D09621 | 0 | hsa_9475 | D10308 | 0 | hsa_4311 | D03217 |
| 1 | hsa_8654 | D09622 | 0 | hsa_9475 | D10560 | 0 | hsa_4311 | D09692 |
| 1 | hsa_8654 | D09989 | 0 | hsa_9475 | D01267 | 0 | hsa_4311 | D08374 |
| 1 | hsa_8654 | D10027 | 0 | hsa_9475 | D08870 | 0 | hsa_43 | D09786 |
| 1 | hsa_8622 | D00718 | 0 | hsa_9212 | D01841 | 0 | hsa_43 | D10635 |
| 1 | hsa_8622 | D01220 | 0 | hsa_9212 | D01846 | 0 | hsa_43 | D07144 |
| 1 | hsa_8622 | D01630 | 0 | hsa_9212 | D10255 | 0 | hsa_43 | D01846 |
| 1 | hsa_8622 | D02218 | 0 | hsa_9212 | D08878 | 0 | hsa_43 | D03718 |
| 1 | hsa_8622 | D06132 | 0 | hsa_9212 | D05400 | 0 | hsa_43 | D07974 |
| 1 | hsa_8622 | D07089 | 0 | hsa_9212 | D01630 | 0 | hsa_43 | D08917 |
| 1 | hsa_8622 | D07425 | 0 | hsa_9212 | D10543 | 0 | hsa_43 | D03747 |
| 1 | hsa_8622 | D07439 | 0 | hsa_9212 | D03115 | 0 | hsa_43 | D04529 |
| 1 | hsa_8622 | D07961 | 0 | hsa_9212 | D10481 | 0 | hsa_43 | D03650 |
| 1 | hsa_8622 | D08238 | 0 | hsa_9212 | D03061 | 0 | hsa_4233 | D09955 |
| 1 | hsa_84647 | D06283 | 0 | hsa_8974 | D10365 | 0 | hsa_4233 | D02729 |
| 1 | hsa_84647 | D08107 | 0 | hsa_8974 | D10064 | 0 | hsa_4233 | D03658 |
| 1 | hsa_84647 | D08221 | 0 | hsa_8974 | D03745 | 0 | hsa_4233 | D09730 |
| 1 | hsa_8399 | D06283 | 0 | hsa_8974 | D09760 | 0 | hsa_4233 | D09750 |
| 1 | hsa_8399 | D08107 | 0 | hsa_8974 | D00428 | 0 | hsa_4233 | D08556 |
| 1 | hsa_8399 | D08221 | 0 | hsa_8974 | D01581 | 0 | hsa_4233 | D10426 |
| 1 | hsa_83933 | D06637 | 0 | hsa_8974 | D06612 | 0 | hsa_4233 | D09881 |
| 1 | hsa_83933 | D08870 | 0 | hsa_8974 | D00564 | 0 | hsa_4129 | D10182 |
| 1 | hsa_83933 | D10019 | 0 | hsa_8972 | D08516 | 0 | hsa_4129 | D10399 |
| 1 | hsa_83933 | D10060 | 0 | hsa_8972 | D07969 | 0 | hsa_4129 | D07439 |
| 1 | hsa_83933 | D10084 | 0 | hsa_8972 | D06272 | 0 | hsa_4129 | D10437 |
| 1 | hsa_83933 | D10319 | 0 | hsa_8972 | D08529 | 0 | hsa_4129 | D10696 |
| 1 | hsa_834 | D08978 | 0 | hsa_8972 | D10019 | 0 | hsa_4129 | D08108 |
| 1 | hsa_834 | D10416 | 0 | hsa_8972 | D10481 | 0 | hsa_4129 | D06645 |
| 1 | hsa_81579 | D06283 | 0 | hsa_8972 | D10616 | 0 | hsa_4129 | D08469 |
| 1 | hsa_81579 | D08107 | 0 | hsa_8972 | D09333 | 0 | hsa_4129 | D01572 |
| 1 | hsa_81579 | D08221 | 0 | hsa_8841 | D05292 | 0 | hsa_4129 | D09919 |
| 1 | hsa_79644 | D00321 | 0 | hsa_8841 | D03828 | 0 | hsa_4129 | D08883 |
| 1 | hsa_79644 | D01134 | 0 | hsa_8841 | D04696 | 0 | hsa_4129 | D06412 |
| 1 | hsa_79644 | D04498 | 0 | hsa_8841 | D00939 | 0 | hsa_4129 | D10189 |
| 1 | hsa_7941 | D03650 | 0 | hsa_8841 | D03082 | 0 | hsa_4129 | D07338 |
| 1 | hsa_7941 | D04368 | 0 | hsa_8841 | D09920 | 0 | hsa_4129 | D10076 |
| 1 | hsa_7941 | D05728 | 0 | hsa_8841 | D06320 | 0 | hsa_4129 | D10262 |
| 1 | hsa_79001 | D00564 | 0 | hsa_8841 | D03747 | 0 | hsa_4128 | D01840 |
| 1 | hsa_79001 | D01280 | 0 | hsa_8841 | D07142 | 0 | hsa_4128 | D09959 |
| 1 | hsa_79001 | D05457 | 0 | hsa_8841 | D09572 | 0 | hsa_4128 | D07564 |
| 1 | hsa_79001 | D07064 | 0 | hsa_8694 | D09692 | 0 | hsa_4128 | D08102 |
| 1 | hsa_79001 | D07131 | 0 | hsa_8694 | D04793 | 0 | hsa_4128 | D08102 |
| 1 | hsa_79001 | D07134 | 0 | hsa_8694 | D01920 | 0 | hsa_4128 | D04681 |
| 1 | hsa_79001 | D07135 | 0 | hsa_8694 | D02755 | 0 | hsa_4128 | D03163 |
| 1 | hsa_79001 | D07136 | 0 | hsa_8694 | D10317 | 0 | hsa_4128 | D10140 |
| 1 | hsa_79001 | D07137 | 0 | hsa_8694 | D08878 | 0 | hsa_4128 | D10130 |
| 1 | hsa_79001 | D07969 | 0 | hsa_8694 | D09919 | 0 | hsa_412 | D08900 |
| 1 | hsa_79001 | D08354 | 0 | hsa_8654 | D10721 | 0 | hsa_412 | D08529 |
| 1 | hsa_79001 | D08682 | 0 | hsa_8654 | D10552 | 0 | hsa_412 | D03234 |
| 1 | hsa_79001 | D09676 | 0 | hsa_8654 | D07142 | 0 | hsa_412 | D08903 |
| 1 | hsa_79001 | D09677 | 0 | hsa_8654 | D07089 | 0 | hsa_412 | D10738 |
| 1 | hsa_771 | D01822 | 0 | hsa_8654 | D07917 | 0 | hsa_412 | D10202 |
| 1 | hsa_771 | D02441 | 0 | hsa_8654 | D05511 | 0 | hsa_412 | D10604 |
| 1 | hsa_771 | D03845 | 0 | hsa_8654 | D10386 | 0 | hsa_412 | D07581 |
| 1 | hsa_771 | D07871 | 0 | hsa_8654 | D04486 | 0 | hsa_412 | D02729 |
| 1 | hsa_768 | D01822 | 0 | hsa_8654 | D03409 | 0 | hsa_412 | D00718 |
| 1 | hsa_768 | D02441 | 0 | hsa_8654 | D09602 | 0 | hsa_412 | D09589 |
| 1 | hsa_768 | D03845 | 0 | hsa_8622 | D07123 | 0 | hsa_412 | D08913 |
| 1 | hsa_768 | D07871 | 0 | hsa_8622 | D09925 | 0 | hsa_412 | D10173 |
| 1 | hsa_767 | D01822 | 0 | hsa_8622 | D10432 | 0 | hsa_412 | D04537 |
| 1 | hsa_767 | D02441 | 0 | hsa_8622 | D10137 | 0 | hsa_4047 | D05819 |
| 1 | hsa_767 | D03845 | 0 | hsa_8622 | D09753 | 0 | hsa_4047 | D08556 |
| 1 | hsa_767 | D07871 | 0 | hsa_8622 | D08238 | 0 | hsa_4047 | D07425 |
| 1 | hsa_766 | D01822 | 0 | hsa_8622 | D07781 | 0 | hsa_4047 | D08073 |
| 1 | hsa_766 | D02441 | 0 | hsa_8622 | D09753 | 0 | hsa_4047 | D10103 |
| 1 | hsa_766 | D03845 | 0 | hsa_8622 | D01630 | 0 | hsa_4047 | D10596 |
| 1 | hsa_766 | D07871 | 0 | hsa_8622 | D09198 | 0 | hsa_4047 | D05589 |
| 1 | hsa_765 | D01822 | 0 | hsa_8622 | D08516 | 0 | hsa_4047 | D10715 |
| 1 | hsa_765 | D02441 | 0 | hsa_8622 | D10709 | 0 | hsa_4047 | D03234 |
| 1 | hsa_765 | D03845 | 0 | hsa_84647 | D09033 | 0 | hsa_4047 | D10085 |
| 1 | hsa_765 | D07871 | 0 | hsa_84647 | D10017 | 0 | hsa_4047 | D09925 |
| 1 | hsa_763 | D01822 | 0 | hsa_84647 | D03254 | 0 | hsa_4023 | D08225 |
| 1 | hsa_763 | D02441 | 0 | hsa_84647 | D04628 | 0 | hsa_4023 | D10664 |
| 1 | hsa_763 | D03845 | 0 | hsa_84647 | D05900 | 0 | hsa_4023 | D08004 |
| 1 | hsa_763 | D07871 | 0 | hsa_84647 | D08682 | 0 | hsa_4023 | D09604 |
| 1 | hsa_762 | D01822 | 0 | hsa_84647 | D03021 | 0 | hsa_391013 | D10189 |
| 1 | hsa_762 | D02441 | 0 | hsa_84647 | D07992 | 0 | hsa_391013 | D00718 |
| 1 | hsa_762 | D03845 | 0 | hsa_84647 | D07817 | 0 | hsa_391013 | D10155 |
| 1 | hsa_762 | D07871 | 0 | hsa_8399 | D04490 | 0 | hsa_391013 | D07134 |
| 1 | hsa_761 | D01822 | 0 | hsa_8399 | D09786 | 0 | hsa_391013 | D06612 |
| 1 | hsa_761 | D02441 | 0 | hsa_8399 | D10173 | 0 | hsa_391013 | D07961 |
| 1 | hsa_761 | D03845 | 0 | hsa_8399 | D09334 | 0 | hsa_391013 | D10653 |
| 1 | hsa_761 | D07871 | 0 | hsa_8399 | D10319 | 0 | hsa_391013 | D07260 |
| 1 | hsa_760 | D01822 | 0 | hsa_8399 | D08261 | 0 | hsa_391013 | D08085 |
| 1 | hsa_760 | D02441 | 0 | hsa_8399 | D04793 | 0 | hsa_391013 | D09756 |
| 1 | hsa_760 | D03845 | 0 | hsa_8399 | D09666 | 0 | hsa_391013 | D03652 |
| 1 | hsa_760 | D07871 | 0 | hsa_83933 | D03657 | 0 | hsa_3818 | D07888 |
| 1 | hsa_759 | D01822 | 0 | hsa_83933 | D03602 | 0 | hsa_3818 | D05181 |
| 1 | hsa_759 | D03845 | 0 | hsa_83933 | D08364 | 0 | hsa_3818 | D04791 |
| 1 | hsa_759 | D07871 | 0 | hsa_83933 | D06612 | 0 | hsa_3818 | D00941 |
| 1 | hsa_7498 | D01206 | 0 | hsa_83933 | D09635 | 0 | hsa_3818 | D00152 |
| 1 | hsa_7498 | D02365 | 0 | hsa_83933 | D07580 | 0 | hsa_3818 | D04628 |
| 1 | hsa_7498 | D07564 | 0 | hsa_83933 | D02729 | 0 | hsa_3818 | D10131 |
| 1 | hsa_7498 | D09786 | 0 | hsa_83933 | D09955 | 0 | hsa_3818 | D06387 |
| 1 | hsa_7357 | D05032 | 0 | hsa_83933 | D08503 | 0 | hsa_3815 | D07917 |
| 1 | hsa_7357 | D09893 | 0 | hsa_834 | D10172 | 0 | hsa_3815 | D09707 |
| 1 | hsa_7357 | D09894 | 0 | hsa_834 | D04822 | 0 | hsa_3815 | D09955 |
| 1 | hsa_7299 | D00073 | 0 | hsa_834 | D00420 | 0 | hsa_3815 | D07147 |
| 1 | hsa_7298 | D01244 | 0 | hsa_834 | D08094 | 0 | hsa_3815 | D09881 |
| 1 | hsa_7298 | D01309 | 0 | hsa_834 | D02365 | 0 | hsa_3791 | D04023 |
| 1 | hsa_7298 | D01784 | 0 | hsa_81579 | D03409 | 0 | hsa_3791 | D10593 |
| 1 | hsa_7298 | D04964 | 0 | hsa_81579 | D08392 | 0 | hsa_3791 | D10202 |
| 1 | hsa_7298 | D07974 | 0 | hsa_81579 | D08294 | 0 | hsa_3791 | D09667 |
| 1 | hsa_7173 | D07231 | 0 | hsa_81579 | D01675 | 0 | hsa_3791 | D10372 |
| 1 | hsa_7173 | D07519 | 0 | hsa_81579 | D08349 | 0 | hsa_377677 | D08860 |
| 1 | hsa_7155 | D01404 | 0 | hsa_81579 | D07818 | 0 | hsa_377677 | D05900 |
| 1 | hsa_7155 | D01747 | 0 | hsa_81579 | D00718 | 0 | hsa_377677 | D04107 |
| 1 | hsa_7155 | D03602 | 0 | hsa_81579 | D07580 | 0 | hsa_377677 | D08363 |
| 1 | hsa_7155 | D04107 | 0 | hsa_79644 | D00942 | 0 | hsa_377677 | D03021 |
| 1 | hsa_7155 | D04685 | 0 | hsa_79644 | D07100 | 0 | hsa_377677 | D09760 |
| 1 | hsa_7155 | D04791 | 0 | hsa_79644 | D10691 | 0 | hsa_377677 | D02365 |
| 1 | hsa_7155 | D07100 | 0 | hsa_79644 | D08688 | 0 | hsa_377677 | D00087 |
| 1 | hsa_7155 | D07776 | 0 | hsa_79644 | D07941 | 0 | hsa_377677 | D04498 |
| 1 | hsa_7155 | D07901 | 0 | hsa_79644 | D09780 | 0 | hsa_377677 | D01841 |
| 1 | hsa_7155 | D08024 | 0 | hsa_79644 | D10138 | 0 | hsa_377677 | D10658 |
| 1 | hsa_7155 | D08062 | 0 | hsa_79644 | D07231 | 0 | hsa_3718 | D10191 |
| 1 | hsa_7155 | D08224 | 0 | hsa_7941 | D06379 | 0 | hsa_3718 | D08682 |
| 1 | hsa_7155 | D08386 | 0 | hsa_7941 | D07556 | 0 | hsa_3718 | D09664 |
| 1 | hsa_7155 | D08854 | 0 | hsa_7941 | D04092 | 0 | hsa_3718 | D05512 |
| 1 | hsa_7155 | D08871 | 0 | hsa_7941 | D04863 | 0 | hsa_3718 | D01309 |
| 1 | hsa_7155 | D10090 | 0 | hsa_7941 | D08657 | 0 | hsa_3718 | D03213 |
| 1 | hsa_7155 | D10091 | 0 | hsa_7941 | D10721 | 0 | hsa_3718 | D07983 |
| 1 | hsa_7153 | D01404 | 0 | hsa_7941 | D04508 | 0 | hsa_3718 | D03657 |
| 1 | hsa_7153 | D01747 | 0 | hsa_7941 | D04770 | 0 | hsa_3718 | D10674 |
| 1 | hsa_7153 | D03602 | 0 | hsa_7941 | D07425 | 0 | hsa_3718 | D09750 |
| 1 | hsa_7153 | D04107 | 0 | hsa_7941 | D07142 | 0 | hsa_3718 | D08555 |
| 1 | hsa_7153 | D04685 | 0 | hsa_79001 | D05893 | 0 | hsa_3718 | D08544 |
| 1 | hsa_7153 | D04791 | 0 | hsa_79001 | D00939 | 0 | hsa_3718 | D08950 |
| 1 | hsa_7153 | D07100 | 0 | hsa_79001 | D01630 | 0 | hsa_3718 | D05590 |
| 1 | hsa_7153 | D07776 | 0 | hsa_79001 | D10585 | 0 | hsa_3718 | D05400 |
| 1 | hsa_7153 | D07901 | 0 | hsa_79001 | D10696 | 0 | hsa_3717 | D10086 |
| 1 | hsa_7153 | D08024 | 0 | hsa_79001 | D10190 | 0 | hsa_3717 | D09956 |
| 1 | hsa_7153 | D08062 | 0 | hsa_79001 | D09925 | 0 | hsa_3717 | D10062 |
| 1 | hsa_7153 | D08224 | 0 | hsa_771 | D09893 | 0 | hsa_3717 | D07699 |
| 1 | hsa_7153 | D08386 | 0 | hsa_771 | D01267 | 0 | hsa_3717 | D07941 |
| 1 | hsa_7153 | D08854 | 0 | hsa_771 | D10382 | 0 | hsa_3717 | D03998 |
| 1 | hsa_7153 | D08871 | 0 | hsa_771 | D07519 | 0 | hsa_3717 | D07260 |
| 1 | hsa_7153 | D10090 | 0 | hsa_771 | D04014 | 0 | hsa_3717 | D02365 |
| 1 | hsa_7153 | D10091 | 0 | hsa_771 | D10471 | 0 | hsa_3717 | D09572 |
| 1 | hsa_7150 | D03225 | 0 | hsa_771 | D04245 | 0 | hsa_3717 | D09198 |
| 1 | hsa_7150 | D03954 | 0 | hsa_771 | D01206 | 0 | hsa_3717 | D07556 |
| 1 | hsa_7150 | D04822 | 0 | hsa_771 | D07781 | 0 | hsa_3717 | D04751 |
| 1 | hsa_7150 | D08086 | 0 | hsa_768 | D10255 | 0 | hsa_3717 | D05513 |
| 1 | hsa_7150 | D08618 | 0 | hsa_768 | D10688 | 0 | hsa_3717 | D08131 |
| 1 | hsa_7150 | D09327 | 0 | hsa_768 | D03099 | 0 | hsa_3716 | D00123 |
| 1 | hsa_7150 | D09679 | 0 | hsa_768 | D08327 | 0 | hsa_3716 | D08427 |
| 1 | hsa_7046 | D10437 | 0 | hsa_768 | D01220 | 0 | hsa_3716 | D05511 |
| 1 | hsa_7010 | D10334 | 0 | hsa_768 | D00941 | 0 | hsa_3716 | D10737 |
| 1 | hsa_7010 | D10399 | 0 | hsa_768 | D06637 | 0 | hsa_3716 | D09920 |
| 1 | hsa_695 | D10223 | 0 | hsa_768 | D09817 | 0 | hsa_3716 | D10584 |
| 1 | hsa_695 | D10730 | 0 | hsa_767 | D10064 | 0 | hsa_3716 | D03663 |
| 1 | hsa_695 | D10731 | 0 | hsa_767 | D03793 | 0 | hsa_3716 | D10365 |
| 1 | hsa_6916 | D01683 | 0 | hsa_767 | D05095 | 0 | hsa_3716 | D10125 |
| 1 | hsa_6916 | D01684 | 0 | hsa_767 | D10635 | 0 | hsa_3716 | D10630 |
| 1 | hsa_6916 | D03661 | 0 | hsa_767 | D07269 | 0 | hsa_3716 | D08393 |
| 1 | hsa_6916 | D03663 | 0 | hsa_767 | D00564 | 0 | hsa_3716 | D06596 |
| 1 | hsa_6916 | D04276 | 0 | hsa_767 | D10308 | 0 | hsa_3716 | D00355 |
| 1 | hsa_6916 | D05502 | 0 | hsa_767 | D10616 | 0 | hsa_3716 | D08873 |
| 1 | hsa_6916 | D08327 | 0 | hsa_766 | D09690 | 0 | hsa_3716 | D10254 |
| 1 | hsa_6868 | D08859 | 0 | hsa_766 | D03239 | 0 | hsa_3620 | D06407 |
| 1 | hsa_6850 | D09347 | 0 | hsa_766 | D09970 | 0 | hsa_3620 | D07134 |
| 1 | hsa_6850 | D09348 | 0 | hsa_766 | D06637 | 0 | hsa_3620 | D01972 |
| 1 | hsa_6795 | D08279 | 0 | hsa_766 | D09728 | 0 | hsa_3620 | D08631 |
| 1 | hsa_6795 | D08344 | 0 | hsa_766 | D08996 | 0 | hsa_3620 | D10138 |
| 1 | hsa_6790 | D08279 | 0 | hsa_765 | D03745 | 0 | hsa_3620 | D05502 |
| 1 | hsa_6790 | D08344 | 0 | hsa_765 | D06553 | 0 | hsa_3615 | D09707 |
| 1 | hsa_6790 | D10085 | 0 | hsa_765 | D09718 | 0 | hsa_3615 | D08279 |
| 1 | hsa_6790 | D10086 | 0 | hsa_765 | D10674 | 0 | hsa_3615 | D07516 |
| 1 | hsa_673 | D06272 | 0 | hsa_765 | D02587 | 0 | hsa_3615 | D01404 |
| 1 | hsa_673 | D08524 | 0 | hsa_765 | D00718 | 0 | hsa_3615 | D07064 |
| 1 | hsa_673 | D09996 | 0 | hsa_765 | D10178 | 0 | hsa_3615 | D00156 |
| 1 | hsa_673 | D10064 | 0 | hsa_765 | D09750 | 0 | hsa_3615 | D10432 |
| 1 | hsa_673 | D10104 | 0 | hsa_765 | D03749 | 0 | hsa_3615 | D10585 |
| 1 | hsa_6716 | D00321 | 0 | hsa_765 | D00749 | 0 | hsa_3614 | D09718 |
| 1 | hsa_6716 | D01134 | 0 | hsa_763 | D09572 | 0 | hsa_3614 | D04681 |
| 1 | hsa_6716 | D04498 | 0 | hsa_763 | D08616 | 0 | hsa_3614 | D04368 |
| 1 | hsa_6715 | D00321 | 0 | hsa_763 | D03990 | 0 | hsa_3614 | D04863 |
| 1 | hsa_6715 | D01134 | 0 | hsa_762 | D09033 | 0 | hsa_3614 | D10157 |
| 1 | hsa_6715 | D03107 | 0 | hsa_762 | D10175 | 0 | hsa_3614 | D07907 |
| 1 | hsa_6715 | D03820 | 0 | hsa_762 | D10315 | 0 | hsa_3480 | D01581 |
| 1 | hsa_6715 | D04498 | 0 | hsa_762 | D10543 | 0 | hsa_3480 | D08354 |
| 1 | hsa_64816 | D09881 | 0 | hsa_762 | D06596 | 0 | hsa_3480 | D07141 |
| 1 | hsa_64600 | D06283 | 0 | hsa_762 | D09707 | 0 | hsa_3480 | D02085 |
| 1 | hsa_64600 | D08107 | 0 | hsa_762 | D08279 | 0 | hsa_3480 | D09679 |
| 1 | hsa_64600 | D08221 | 0 | hsa_762 | D08363 | 0 | hsa_3480 | D00939 |
| 1 | hsa_63928 | D00107 | 0 | hsa_762 | D10062 | 0 | hsa_3480 | D06378 |
| 1 | hsa_63928 | D00184 | 0 | hsa_762 | D03217 | 0 | hsa_3480 | D07269 |
| 1 | hsa_63928 | D05480 | 0 | hsa_762 | D07781 | 0 | hsa_3480 | D04863 |
| 1 | hsa_63928 | D08556 | 0 | hsa_762 | D02565 | 0 | hsa_3480 | D09750 |
| 1 | hsa_63928 | D09033 | 0 | hsa_762 | D10596 | 0 | hsa_3290 | D09920 |
| 1 | hsa_6300 | D09602 | 0 | hsa_762 | D08878 | 0 | hsa_3290 | D04685 |
| 1 | hsa_6300 | D09603 | 0 | hsa_762 | D10062 | 0 | hsa_3290 | D03150 |
| 1 | hsa_6300 | D09639 | 0 | hsa_761 | D01899 | 0 | hsa_3290 | D09728 |
| 1 | hsa_6300 | D10658 | 0 | hsa_761 | D10140 | 0 | hsa_3290 | D08484 |
| 1 | hsa_6300 | D10659 | 0 | hsa_761 | D10222 | 0 | hsa_3290 | D06634 |
| 1 | hsa_6241 | D00341 | 0 | hsa_761 | D01630 | 0 | hsa_3290 | D09913 |
| 1 | hsa_6241 | D03546 | 0 | hsa_761 | D04368 | 0 | hsa_3242 | D01206 |
| 1 | hsa_6240 | D01155 | 0 | hsa_761 | D07089 | 0 | hsa_3242 | D01206 |
| 1 | hsa_6240 | D03378 | 0 | hsa_761 | D09690 | 0 | hsa_3242 | D08883 |
| 1 | hsa_6240 | D03546 | 0 | hsa_761 | D08668 | 0 | hsa_3242 | D06378 |
| 1 | hsa_6240 | D10222 | 0 | hsa_761 | D03745 | 0 | hsa_3242 | D08225 |
| 1 | hsa_6093 | D01840 | 0 | hsa_761 | D03252 | 0 | hsa_3242 | D03517 |
| 1 | hsa_6093 | D03115 | 0 | hsa_760 | D07144 | 0 | hsa_3242 | D03115 |
| 1 | hsa_6093 | D07941 | 0 | hsa_760 | D07136 | 0 | hsa_3156 | D10657 |
| 1 | hsa_6093 | D10737 | 0 | hsa_760 | D08392 | 0 | hsa_3156 | D10229 |
| 1 | hsa_6093 | D10738 | 0 | hsa_760 | D08913 | 0 | hsa_3156 | D07064 |
| 1 | hsa_6051 | D00087 | 0 | hsa_760 | D10179 | 0 | hsa_3156 | D08094 |
| 1 | hsa_5972 | D03208 | 0 | hsa_760 | D10515 | 0 | hsa_3156 | D10182 |
| 1 | hsa_5972 | D03745 | 0 | hsa_760 | D10437 | 0 | hsa_3156 | D10382 |
| 1 | hsa_5972 | D06412 | 0 | hsa_760 | D10465 | 0 | hsa_3156 | D10026 |
| 1 | hsa_5972 | D09038 | 0 | hsa_760 | D09618 | 0 | hsa_3156 | D09664 |
| 1 | hsa_5837 | D04537 | 0 | hsa_760 | D08469 | 0 | hsa_3156 | D06596 |
| 1 | hsa_5836 | D04537 | 0 | hsa_759 | D09728 | 0 | hsa_3156 | D10585 |
| 1 | hsa_5834 | D04537 | 0 | hsa_759 | D10471 | 0 | hsa_3156 | D10191 |
| 1 | hsa_5770 | D04050 | 0 | hsa_759 | D05451 | 0 | hsa_3156 | D09955 |
| 1 | hsa_5743 | D01049 | 0 | hsa_759 | D08516 | 0 | hsa_3156 | D01784 |
| 1 | hsa_5743 | D01267 | 0 | hsa_759 | D08873 | 0 | hsa_30814 | D02729 |
| 1 | hsa_5743 | D01545 | 0 | hsa_759 | D10064 | 0 | hsa_30814 | D09894 |
| 1 | hsa_5743 | D03163 | 0 | hsa_759 | D07983 | 0 | hsa_30814 | D01840 |
| 1 | hsa_5743 | D04863 | 0 | hsa_759 | D08870 | 0 | hsa_30814 | D03661 |
| 1 | hsa_5743 | D07541 | 0 | hsa_759 | D08860 | 0 | hsa_30814 | D09731 |
| 1 | hsa_5743 | D07888 | 0 | hsa_759 | D07961 | 0 | hsa_30814 | D07134 |
| 1 | hsa_5743 | D08657 | 0 | hsa_759 | D07917 | 0 | hsa_30814 | D02218 |
| 1 | hsa_5743 | D10656 | 0 | hsa_7498 | D06609 | 0 | hsa_30814 | D10560 |
| 1 | hsa_5742 | D00151 | 0 | hsa_7498 | D08552 | 0 | hsa_3067 | D10104 |
| 1 | hsa_5742 | D00428 | 0 | hsa_7498 | D08066 | 0 | hsa_3067 | D10419 |
| 1 | hsa_5742 | D01090 | 0 | hsa_7498 | D08453 | 0 | hsa_3067 | D07082 |
| 1 | hsa_5742 | D01410 | 0 | hsa_7498 | D03745 | 0 | hsa_3067 | D09038 |
| 1 | hsa_5742 | D01581 | 0 | hsa_7498 | D10733 | 0 | hsa_3067 | D03208 |
| 1 | hsa_5742 | D01675 | 0 | hsa_7498 | D08374 | 0 | hsa_3067 | D02729 |
| 1 | hsa_5742 | D01788 | 0 | hsa_7498 | D05590 | 0 | hsa_3067 | D08917 |
| 1 | hsa_5742 | D01823 | 0 | hsa_7498 | D05906 | 0 | hsa_3067 | D03208 |
| 1 | hsa_5742 | D01841 | 0 | hsa_7498 | D10552 | 0 | hsa_3067 | D06678 |
| 1 | hsa_5742 | D03254 | 0 | hsa_7357 | D08668 | 0 | hsa_3067 | D09326 |
| 1 | hsa_5742 | D03715 | 0 | hsa_7357 | D00939 | 0 | hsa_3067 | D03658 |
| 1 | hsa_5742 | D03718 | 0 | hsa_7357 | D00718 | 0 | hsa_3067 | D10137 |
| 1 | hsa_5742 | D04490 | 0 | hsa_7357 | D08059 | 0 | hsa_3067 | D03802 |
| 1 | hsa_5742 | D05143 | 0 | hsa_7357 | D10529 | 0 | hsa_3067 | D10024 |
| 1 | hsa_5742 | D05181 | 0 | hsa_7357 | D04508 | 0 | hsa_3066 | D10431 |
| 1 | hsa_5742 | D05319 | 0 | hsa_7357 | D08492 | 0 | hsa_3066 | D07541 |
| 1 | hsa_5742 | D05451 | 0 | hsa_7357 | D09334 | 0 | hsa_3066 | D08631 |
| 1 | hsa_5742 | D05511 | 0 | hsa_7357 | D08149 | 0 | hsa_3066 | D08073 |
| 1 | hsa_5742 | D05512 | 0 | hsa_7299 | D08616 | 0 | hsa_3066 | D05513 |
| 1 | hsa_5742 | D05513 | 0 | hsa_7299 | D09635 | 0 | hsa_3066 | D07579 |
| 1 | hsa_5742 | D05598 | 0 | hsa_7299 | D03602 | 0 | hsa_3065 | D08524 |
| 1 | hsa_5742 | D06606 | 0 | hsa_7299 | D01896 | 0 | hsa_3065 | D10183 |
| 1 | hsa_5742 | D07141 | 0 | hsa_7299 | D08625 | 0 | hsa_3065 | D06553 |
| 1 | hsa_5742 | D07142 | 0 | hsa_7299 | D10318 | 0 | hsa_3065 | D07136 |
| 1 | hsa_5742 | D07269 | 0 | hsa_7299 | D09780 | 0 | hsa_3065 | D01788 |
| 1 | hsa_5742 | D07443 | 0 | hsa_7299 | D10358 | 0 | hsa_3065 | D00073 |
| 1 | hsa_5742 | D07516 | 0 | hsa_7298 | D00939 | 0 | hsa_3065 | D00321 |
| 1 | hsa_5742 | D07517 | 0 | hsa_7298 | D07516 | 0 | hsa_3065 | D10317 |
| 1 | hsa_5742 | D07579 | 0 | hsa_7298 | D08349 | 0 | hsa_3065 | D10154 |
| 1 | hsa_5742 | D07580 | 0 | hsa_7298 | D10585 | 0 | hsa_3065 | D06578 |
| 1 | hsa_5742 | D07581 | 0 | hsa_7298 | D08907 | 0 | hsa_3065 | D10138 |
| 1 | hsa_5742 | D07582 | 0 | hsa_7298 | D00355 | 0 | hsa_3065 | D10183 |
| 1 | hsa_5742 | D07816 | 0 | hsa_7298 | D08689 | 0 | hsa_3065 | D08529 |
| 1 | hsa_5742 | D07817 | 0 | hsa_7298 | D01920 | 0 | hsa_3065 | D10630 |
| 1 | hsa_5742 | D07818 | 0 | hsa_7298 | D10334 | 0 | hsa_3065 | D09690 |
| 1 | hsa_5742 | D07819 | 0 | hsa_7298 | D07879 | 0 | hsa_29947 | D10254 |
| 1 | hsa_5742 | D08058 | 0 | hsa_7298 | D04863 | 0 | hsa_29947 | D00152 |
| 1 | hsa_5742 | D08059 | 0 | hsa_7173 | D06578 | 0 | hsa_29947 | D05094 |
| 1 | hsa_5742 | D08102 | 0 | hsa_7173 | D00156 | 0 | hsa_29947 | D04092 |
| 1 | hsa_5742 | D08103 | 0 | hsa_7173 | D06609 | 0 | hsa_29947 | D10104 |
| 1 | hsa_5742 | D08104 | 0 | hsa_7173 | D09641 | 0 | hsa_2984 | D05590 |
| 1 | hsa_5742 | D08149 | 0 | hsa_7173 | D03252 | 0 | hsa_2984 | D01672 |
| 1 | hsa_5742 | D08162 | 0 | hsa_7173 | D10183 | 0 | hsa_2984 | D07699 |
| 1 | hsa_5742 | D08324 | 0 | hsa_7173 | D10419 | 0 | hsa_2983 | D06387 |
| 1 | hsa_5742 | D08363 | 0 | hsa_7173 | D10062 | 0 | hsa_2983 | D08261 |
| 1 | hsa_5742 | D08364 | 0 | hsa_7155 | D09960 | 0 | hsa_2983 | D03517 |
| 1 | hsa_5742 | D08374 | 0 | hsa_7155 | D09920 | 0 | hsa_2983 | D04368 |
| 1 | hsa_5742 | D08375 | 0 | hsa_7155 | D08149 | 0 | hsa_2983 | D03942 |
| 1 | hsa_5742 | D08427 | 0 | hsa_7155 | D08917 | 0 | hsa_2983 | D07818 |
| 1 | hsa_5742 | D09760 | 0 | hsa_7155 | D09718 | 0 | hsa_2983 | D07141 |
| 1 | hsa_5742 | D10017 | 0 | hsa_7155 | D09572 | 0 | hsa_2983 | D03517 |
| 1 | hsa_5742 | D10254 | 0 | hsa_7155 | D08917 | 0 | hsa_2983 | D10585 |
| 1 | hsa_5693 | D08880 | 0 | hsa_7155 | D07817 | 0 | hsa_2982 | D06645 |
| 1 | hsa_5693 | D10130 | 0 | hsa_7155 | D09959 | 0 | hsa_2982 | D08688 |
| 1 | hsa_5693 | D10131 | 0 | hsa_7155 | D09893 | 0 | hsa_2982 | D07974 |
| 1 | hsa_5693 | D10318 | 0 | hsa_7155 | D08324 | 0 | hsa_2982 | D10405 |
| 1 | hsa_5689 | D03150 | 0 | hsa_7153 | D09667 | 0 | hsa_2982 | D07064 |
| 1 | hsa_5689 | D09640 | 0 | hsa_7153 | D09718 | 0 | hsa_2982 | D06637 |
| 1 | hsa_5689 | D10110 | 0 | hsa_7153 | D08104 | 0 | hsa_2982 | D09326 |
| 1 | hsa_5605 | D09666 | 0 | hsa_7153 | D10450 | 0 | hsa_2982 | D03107 |
| 1 | hsa_5605 | D10024 | 0 | hsa_7153 | D10737 | 0 | hsa_2977 | D06387 |
| 1 | hsa_5605 | D10175 | 0 | hsa_7153 | D09635 | 0 | hsa_2977 | D01672 |
| 1 | hsa_5605 | D10176 | 0 | hsa_7153 | D08953 | 0 | hsa_2977 | D09915 |
| 1 | hsa_5605 | D10426 | 0 | hsa_7153 | D09665 | 0 | hsa_2977 | D09572 |
| 1 | hsa_5605 | D10486 | 0 | hsa_7153 | D06407 | 0 | hsa_2977 | D07983 |
| 1 | hsa_5605 | D10604 | 0 | hsa_7150 | D06634 | 0 | hsa_2977 | D02587 |
| 1 | hsa_5604 | D09666 | 0 | hsa_7150 | D07260 | 0 | hsa_2950 | D04768 |
| 1 | hsa_5604 | D10024 | 0 | hsa_7150 | D10138 | 0 | hsa_2950 | D08529 |
| 1 | hsa_5604 | D10175 | 0 | hsa_7150 | D01784 | 0 | hsa_2950 | D07577 |
| 1 | hsa_5604 | D10176 | 0 | hsa_7150 | D01134 | 0 | hsa_2950 | D03715 |
| 1 | hsa_5604 | D10426 | 0 | hsa_7150 | D08453 | 0 | hsa_2950 | D10173 |
| 1 | hsa_5604 | D10486 | 0 | hsa_7150 | D01788 | 0 | hsa_2950 | D05094 |
| 1 | hsa_5604 | D10604 | 0 | hsa_7046 | D03546 | 0 | hsa_2950 | D07499 |
| 1 | hsa_5603 | D09602 | 0 | hsa_7046 | D00944 | 0 | hsa_2950 | D10317 |
| 1 | hsa_5603 | D09603 | 0 | hsa_7046 | D08492 | 0 | hsa_2950 | D08907 |
| 1 | hsa_5603 | D09639 | 0 | hsa_7046 | D08086 | 0 | hsa_2950 | D05906 |
| 1 | hsa_5603 | D10658 | 0 | hsa_7046 | D10062 | 0 | hsa_2950 | D01899 |
| 1 | hsa_5603 | D10659 | 0 | hsa_7046 | D09710 | 0 | hsa_290 | D03929 |
| 1 | hsa_5602 | D10168 | 0 | hsa_7046 | D10733 | 0 | hsa_290 | D10615 |
| 1 | hsa_5601 | D10168 | 0 | hsa_7046 | D03845 | 0 | hsa_290 | D07817 |
| 1 | hsa_5600 | D09602 | 0 | hsa_7046 | D07580 | 0 | hsa_290 | D09625 |
| 1 | hsa_5600 | D09603 | 0 | hsa_7046 | D08555 | 0 | hsa_290 | D07517 |
| 1 | hsa_5600 | D09639 | 0 | hsa_7046 | D10615 | 0 | hsa_290 | D07337 |
| 1 | hsa_5600 | D10658 | 0 | hsa_7010 | D00428 | 0 | hsa_283208 | D04696 |
| 1 | hsa_5600 | D10659 | 0 | hsa_7010 | D07142 | 0 | hsa_283208 | D08516 |
| 1 | hsa_5599 | D10168 | 0 | hsa_7010 | D08375 | 0 | hsa_283208 | D04685 |
| 1 | hsa_5595 | D10405 | 0 | hsa_7010 | D10543 | 0 | hsa_283208 | D07555 |
| 1 | hsa_5595 | D10615 | 0 | hsa_7010 | D09753 | 0 | hsa_283208 | D06606 |
| 1 | hsa_5594 | D10405 | 0 | hsa_7010 | D03213 | 0 | hsa_283208 | D08688 |
| 1 | hsa_5594 | D10615 | 0 | hsa_7010 | D06320 | 0 | hsa_283208 | D01244 |
| 1 | hsa_5588 | D09671 | 0 | hsa_7010 | D08854 | 0 | hsa_283208 | D09881 |
| 1 | hsa_5588 | D09718 | 0 | hsa_7010 | D07818 | 0 | hsa_283208 | D01846 |
| 1 | hsa_55869 | D06637 | 0 | hsa_7010 | D06378 | 0 | hsa_283208 | D07901 |
| 1 | hsa_55869 | D08870 | 0 | hsa_695 | D07144 | 0 | hsa_283208 | D09817 |
| 1 | hsa_55869 | D10019 | 0 | hsa_695 | D02084 | 0 | hsa_27115 | D09546 |
| 1 | hsa_55869 | D10060 | 0 | hsa_695 | D08860 | 0 | hsa_27115 | D07541 |
| 1 | hsa_55869 | D10084 | 0 | hsa_695 | D06606 | 0 | hsa_27115 | D07260 |
| 1 | hsa_55869 | D10319 | 0 | hsa_695 | D09602 | 0 | hsa_27115 | D10674 |
| 1 | hsa_5583 | D09671 | 0 | hsa_695 | D08162 | 0 | hsa_27115 | D08354 |
| 1 | hsa_5583 | D09718 | 0 | hsa_695 | D10308 | 0 | hsa_27115 | D10222 |
| 1 | hsa_55811 | D01697 | 0 | hsa_6916 | D04685 | 0 | hsa_27115 | D00073 |
| 1 | hsa_5581 | D09671 | 0 | hsa_6916 | D10318 | 0 | hsa_2687 | D10609 |
| 1 | hsa_5581 | D09718 | 0 | hsa_6916 | D07818 | 0 | hsa_2687 | D08364 |
| 1 | hsa_5580 | D09671 | 0 | hsa_6916 | D10696 | 0 | hsa_2687 | D07941 |
| 1 | hsa_5580 | D09718 | 0 | hsa_6868 | D00939 | 0 | hsa_2687 | D03010 |
| 1 | hsa_5579 | D04014 | 0 | hsa_6868 | D08996 | 0 | hsa_2687 | D09718 |
| 1 | hsa_5563 | D00595 | 0 | hsa_6868 | D09847 | 0 | hsa_2687 | D06553 |
| 1 | hsa_5563 | D00944 | 0 | hsa_6868 | D09915 | 0 | hsa_2687 | D03021 |
| 1 | hsa_5563 | D02206 | 0 | hsa_6868 | D08903 | 0 | hsa_2687 | D08917 |
| 1 | hsa_5563 | D04966 | 0 | hsa_6868 | D03010 | 0 | hsa_2687 | D08369 |
| 1 | hsa_5563 | D08351 | 0 | hsa_6868 | D00564 | 0 | hsa_2687 | D10086 |
| 1 | hsa_5563 | D08352 | 0 | hsa_6868 | D09710 | 0 | hsa_2687 | D03021 |
| 1 | hsa_5562 | D00595 | 0 | hsa_6868 | D09033 | 0 | hsa_2686 | D08327 |
| 1 | hsa_5562 | D00944 | 0 | hsa_6850 | D06407 | 0 | hsa_2686 | D03942 |
| 1 | hsa_5562 | D02206 | 0 | hsa_6850 | D09996 | 0 | hsa_2686 | D03349 |
| 1 | hsa_5562 | D04966 | 0 | hsa_6850 | D03797 | 0 | hsa_2686 | D10104 |
| 1 | hsa_5562 | D08351 | 0 | hsa_6850 | D10102 | 0 | hsa_2686 | D08689 |
| 1 | hsa_5562 | D08352 | 0 | hsa_6850 | D02714 | 0 | hsa_2686 | D04681 |
| 1 | hsa_5535 | D00107 | 0 | hsa_6850 | D09357 | 0 | hsa_2686 | D05819 |
| 1 | hsa_5535 | D00184 | 0 | hsa_6850 | D09667 | 0 | hsa_2686 | D10137 |
| 1 | hsa_5535 | D05480 | 0 | hsa_6850 | D00181 | 0 | hsa_2686 | D07144 |
| 1 | hsa_5535 | D08556 | 0 | hsa_6850 | D05399 | 0 | hsa_2686 | D08688 |
| 1 | hsa_5535 | D09033 | 0 | hsa_6850 | D08085 | 0 | hsa_2686 | D06637 |
| 1 | hsa_5534 | D00107 | 0 | hsa_6850 | D10365 | 0 | hsa_2686 | D07269 |
| 1 | hsa_5534 | D00184 | 0 | hsa_6850 | D05019 | 0 | hsa_2686 | D10026 |
| 1 | hsa_5534 | D05480 | 0 | hsa_6850 | D09355 | 0 | hsa_2678 | D08514 |
| 1 | hsa_5534 | D08556 | 0 | hsa_6795 | D00753 | 0 | hsa_2678 | D08544 |
| 1 | hsa_5534 | D09033 | 0 | hsa_6795 | D10437 | 0 | hsa_2678 | D03248 |
| 1 | hsa_5533 | D05480 | 0 | hsa_6795 | D10529 | 0 | hsa_2678 | D09955 |
| 1 | hsa_5533 | D08556 | 0 | hsa_6795 | D03929 | 0 | hsa_2678 | D08529 |
| 1 | hsa_5533 | D09033 | 0 | hsa_6795 | D07143 | 0 | hsa_2678 | D10365 |
| 1 | hsa_5532 | D05480 | 0 | hsa_6795 | D10560 | 0 | hsa_2678 | D09679 |
| 1 | hsa_5532 | D08556 | 0 | hsa_6795 | D02985 | 0 | hsa_2678 | D10110 |
| 1 | hsa_5532 | D09033 | 0 | hsa_6795 | D10659 | 0 | hsa_26279 | D01697 |
| 1 | hsa_5530 | D05480 | 0 | hsa_6790 | D01846 | 0 | hsa_26279 | D09677 |
| 1 | hsa_5530 | D08556 | 0 | hsa_6790 | D05292 | 0 | hsa_26279 | D10104 |
| 1 | hsa_5530 | D09033 | 0 | hsa_6790 | D04023 | 0 | hsa_26279 | D03942 |
| 1 | hsa_54583 | D10593 | 0 | hsa_6790 | D10255 | 0 | hsa_26279 | D09333 |
| 1 | hsa_5347 | D10154 | 0 | hsa_6790 | D01090 | 0 | hsa_26279 | D01572 |
| 1 | hsa_5347 | D10155 | 0 | hsa_6790 | D06413 | 0 | hsa_26279 | D06637 |
| 1 | hsa_5347 | D10182 | 0 | hsa_6790 | D01049 | 0 | hsa_2618 | D10481 |
| 1 | hsa_5347 | D10183 | 0 | hsa_673 | D07969 | 0 | hsa_2618 | D09327 |
| 1 | hsa_5322 | D06283 | 0 | hsa_673 | D10102 | 0 | hsa_2618 | D10584 |
| 1 | hsa_5322 | D08107 | 0 | hsa_673 | D01244 | 0 | hsa_2618 | D10372 |
| 1 | hsa_5322 | D08221 | 0 | hsa_673 | D03747 | 0 | hsa_2618 | D07089 |
| 1 | hsa_5321 | D03938 | 0 | hsa_673 | D09925 | 0 | hsa_2618 | D01675 |
| 1 | hsa_5321 | D06600 | 0 | hsa_673 | D05512 | 0 | hsa_2618 | D09956 |
| 1 | hsa_5321 | D06674 | 0 | hsa_673 | D00355 | 0 | hsa_2595 | D10172 |
| 1 | hsa_5320 | D06283 | 0 | hsa_673 | D09621 | 0 | hsa_2595 | D06612 |
| 1 | hsa_5320 | D08107 | 0 | hsa_673 | D03661 | 0 | hsa_2595 | D04768 |
| 1 | hsa_5320 | D08221 | 0 | hsa_673 | D08094 | 0 | hsa_2595 | D05096 |
| 1 | hsa_5319 | D06283 | 0 | hsa_6716 | D02714 | 0 | hsa_2595 | D01896 |
| 1 | hsa_5319 | D08107 | 0 | hsa_6716 | D08907 | 0 | hsa_2595 | D08162 |
| 1 | hsa_5319 | D08221 | 0 | hsa_6716 | D10358 | 0 | hsa_2595 | D10652 |
| 1 | hsa_5294 | D10189 | 0 | hsa_6716 | D04822 | 0 | hsa_2548 | D05095 |
| 1 | hsa_5294 | D10552 | 0 | hsa_6716 | D10584 | 0 | hsa_2548 | D03213 |
| 1 | hsa_5294 | D10616 | 0 | hsa_6716 | D07871 | 0 | hsa_2548 | D09760 |
| 1 | hsa_5294 | D10635 | 0 | hsa_6716 | D10140 | 0 | hsa_2548 | D08907 |
| 1 | hsa_5293 | D10189 | 0 | hsa_6716 | D07136 | 0 | hsa_2548 | D00595 |
| 1 | hsa_5293 | D10552 | 0 | hsa_6715 | D09718 | 0 | hsa_2548 | D09333 |
| 1 | hsa_5293 | D10616 | 0 | hsa_6715 | D06068 | 0 | hsa_2548 | D09959 |
| 1 | hsa_5293 | D10635 | 0 | hsa_6715 | D07907 | 0 | hsa_2548 | D08529 |
| 1 | hsa_5291 | D10189 | 0 | hsa_6715 | D09692 | 0 | hsa_2548 | D03217 |
| 1 | hsa_5291 | D10552 | 0 | hsa_6715 | D03107 | 0 | hsa_2548 | D04751 |
| 1 | hsa_5291 | D10616 | 0 | hsa_6715 | D07080 | 0 | hsa_2548 | D08469 |
| 1 | hsa_5291 | D10635 | 0 | hsa_6715 | D08963 | 0 | hsa_25 | D08004 |
| 1 | hsa_5290 | D10189 | 0 | hsa_6715 | D04696 | 0 | hsa_25 | D09925 |
| 1 | hsa_5290 | D10543 | 0 | hsa_64816 | D07577 | 0 | hsa_25 | D09883 |
| 1 | hsa_5290 | D10552 | 0 | hsa_64816 | D09326 | 0 | hsa_25 | D08860 |
| 1 | hsa_5290 | D10616 | 0 | hsa_64816 | D10154 | 0 | hsa_25 | D05399 |
| 1 | hsa_5290 | D10635 | 0 | hsa_64816 | D09956 | 0 | hsa_25 | D01070 |
| 1 | hsa_5289 | D10560 | 0 | hsa_64816 | D04696 | 0 | hsa_25 | D05096 |
| 1 | hsa_5289 | D10584 | 0 | hsa_64816 | D08953 | 0 | hsa_2475 | D00321 |
| 1 | hsa_5289 | D10609 | 0 | hsa_64816 | D06068 | 0 | hsa_2475 | D01840 |
| 1 | hsa_5243 | D06008 | 0 | hsa_64816 | D10155 | 0 | hsa_2475 | D01070 |
| 1 | hsa_5243 | D06387 | 0 | hsa_64816 | D08427 | 0 | hsa_2475 | D03657 |
| 1 | hsa_5159 | D06285 | 0 | hsa_64816 | D06503 | 0 | hsa_240 | D07425 |
| 1 | hsa_5159 | D10102 | 0 | hsa_64816 | D10585 | 0 | hsa_240 | D06283 |
| 1 | hsa_5159 | D10103 | 0 | hsa_64816 | D00420 | 0 | hsa_240 | D03239 |
| 1 | hsa_51564 | D06637 | 0 | hsa_64816 | D05906 | 0 | hsa_240 | D09679 |
| 1 | hsa_51564 | D08870 | 0 | hsa_64816 | D10652 | 0 | hsa_240 | D10155 |
| 1 | hsa_51564 | D10019 | 0 | hsa_64816 | D07940 | 0 | hsa_240 | D10017 |
| 1 | hsa_51564 | D10060 | 0 | hsa_64600 | D09327 | 0 | hsa_240 | D06606 |
| 1 | hsa_51564 | D10084 | 0 | hsa_64600 | D08878 | 0 | hsa_240 | D05029 |
| 1 | hsa_51564 | D10319 | 0 | hsa_64600 | D08556 | 0 | hsa_240 | D10173 |
| 1 | hsa_5156 | D05380 | 0 | hsa_64600 | D02714 | 0 | hsa_240 | D05511 |
| 1 | hsa_5156 | D06678 | 0 | hsa_64600 | D08907 | 0 | hsa_240 | D01259 |
| 1 | hsa_5156 | D08947 | 0 | hsa_64600 | D05598 | 0 | hsa_240 | D08073 |
| 1 | hsa_5156 | D10062 | 0 | hsa_64600 | D09667 | 0 | hsa_238 | D06575 |
| 1 | hsa_5156 | D10102 | 0 | hsa_64600 | D08871 | 0 | hsa_238 | D03208 |
| 1 | hsa_5156 | D10103 | 0 | hsa_64600 | D00749 | 0 | hsa_238 | D07064 |
| 1 | hsa_5153 | D00718 | 0 | hsa_64600 | D07983 | 0 | hsa_238 | D10157 |
| 1 | hsa_5153 | D01220 | 0 | hsa_64600 | D07141 | 0 | hsa_238 | D05480 |
| 1 | hsa_5153 | D01630 | 0 | hsa_63928 | D10091 | 0 | hsa_238 | D10176 |
| 1 | hsa_5153 | D02218 | 0 | hsa_63928 | D09919 | 0 | hsa_238 | D10657 |
| 1 | hsa_5153 | D06132 | 0 | hsa_63928 | D10543 | 0 | hsa_238 | D10222 |
| 1 | hsa_5153 | D07089 | 0 | hsa_63928 | D09546 | 0 | hsa_238 | D10674 |
| 1 | hsa_5153 | D07425 | 0 | hsa_63928 | D04092 | 0 | hsa_238 | D10315 |
| 1 | hsa_5153 | D07439 | 0 | hsa_63928 | D04791 | 0 | hsa_23632 | D10450 |
| 1 | hsa_5153 | D07961 | 0 | hsa_6300 | D09690 | 0 | hsa_23632 | D09925 |
| 1 | hsa_5153 | D08238 | 0 | hsa_6300 | D09923 | 0 | hsa_23632 | D03929 |
| 1 | hsa_5151 | D00718 | 0 | hsa_6300 | D00073 | 0 | hsa_23632 | D06645 |
| 1 | hsa_5151 | D01220 | 0 | hsa_6300 | D07818 | 0 | hsa_23632 | D09707 |
| 1 | hsa_5151 | D01630 | 0 | hsa_6300 | D04936 | 0 | hsa_23632 | D08062 |
| 1 | hsa_5151 | D02218 | 0 | hsa_6300 | D10515 | 0 | hsa_23632 | D03802 |
| 1 | hsa_5151 | D06132 | 0 | hsa_6300 | D09665 | 0 | hsa_23632 | D10656 |
| 1 | hsa_5151 | D07089 | 0 | hsa_6300 | D08516 | 0 | hsa_23632 | D01155 |
| 1 | hsa_5151 | D07425 | 0 | hsa_6300 | D08024 | 0 | hsa_23632 | D07819 |
| 1 | hsa_5151 | D07439 | 0 | hsa_6241 | D10486 | 0 | hsa_23632 | D10076 |
| 1 | hsa_5151 | D07961 | 0 | hsa_6241 | D05906 | 0 | hsa_23621 | D04185 |
| 1 | hsa_5151 | D08238 | 0 | hsa_6241 | D10131 | 0 | hsa_23621 | D09689 |
| 1 | hsa_5150 | D00718 | 0 | hsa_6241 | D05589 | 0 | hsa_23621 | D10173 |
| 1 | hsa_5150 | D01220 | 0 | hsa_6241 | D08073 | 0 | hsa_23621 | D03749 |
| 1 | hsa_5150 | D01630 | 0 | hsa_6241 | D03150 | 0 | hsa_23621 | D03650 |
| 1 | hsa_5150 | D02218 | 0 | hsa_6240 | D04720 | 0 | hsa_23621 | D10653 |
| 1 | hsa_5150 | D06132 | 0 | hsa_6240 | D10024 | 0 | hsa_23621 | D07443 |
| 1 | hsa_5150 | D07089 | 0 | hsa_6240 | D08463 | 0 | hsa_23621 | D07969 |
| 1 | hsa_5150 | D07425 | 0 | hsa_6240 | D10657 | 0 | hsa_23621 | D08162 |
| 1 | hsa_5150 | D07439 | 0 | hsa_6240 | D03715 | 0 | hsa_23621 | D06503 |
| 1 | hsa_5150 | D07961 | 0 | hsa_6240 | D03107 | 0 | hsa_23621 | D04014 |
| 1 | hsa_5150 | D08238 | 0 | hsa_6240 | D08880 | 0 | hsa_23621 | D07143 |
| 1 | hsa_5144 | D00718 | 0 | hsa_6240 | D08324 | 0 | hsa_23439 | D04498 |
| 1 | hsa_5144 | D01220 | 0 | hsa_6093 | D10176 | 0 | hsa_23439 | D08689 |
| 1 | hsa_5144 | D01630 | 0 | hsa_6093 | D03718 | 0 | hsa_23439 | D10138 |
| 1 | hsa_5144 | D02218 | 0 | hsa_6093 | D07082 | 0 | hsa_23439 | D10543 |
| 1 | hsa_5144 | D02985 | 0 | hsa_6093 | D04936 | 0 | hsa_23439 | D10137 |
| 1 | hsa_5144 | D04185 | 0 | hsa_6093 | D10062 | 0 | hsa_23439 | D08880 |
| 1 | hsa_5144 | D05474 | 0 | hsa_6051 | D04245 | 0 | hsa_23439 | D01675 |
| 1 | hsa_5144 | D05744 | 0 | hsa_6051 | D05511 | 0 | hsa_2342 | D08085 |
| 1 | hsa_5144 | D06132 | 0 | hsa_6051 | D05480 | 0 | hsa_2342 | D09683 |
| 1 | hsa_5144 | D06575 | 0 | hsa_6051 | D07564 | 0 | hsa_2342 | D06320 |
| 1 | hsa_5144 | D07088 | 0 | hsa_6051 | D03409 | 0 | hsa_2342 | D03938 |
| 1 | hsa_5144 | D07089 | 0 | hsa_6051 | D05096 | 0 | hsa_2342 | D09198 |
| 1 | hsa_5144 | D07425 | 0 | hsa_6051 | D03061 | 0 | hsa_2342 | D00087 |
| 1 | hsa_5144 | D07439 | 0 | hsa_6051 | D08107 | 0 | hsa_2342 | D08068 |
| 1 | hsa_5144 | D07879 | 0 | hsa_6051 | D10696 | 0 | hsa_2342 | D05589 |
| 1 | hsa_5144 | D07961 | 0 | hsa_6051 | D10334 | 0 | hsa_2342 | D09677 |
| 1 | hsa_5144 | D08238 | 0 | hsa_6051 | D00355 | 0 | hsa_2342 | D07064 |
| 1 | hsa_5144 | D08860 | 0 | hsa_6051 | D01404 | 0 | hsa_2342 | D04791 |
| 1 | hsa_5144 | D09020 | 0 | hsa_6051 | D00152 | 0 | hsa_2339 | D05143 |
| 1 | hsa_5143 | D00718 | 0 | hsa_6051 | D10426 | 0 | hsa_2339 | D10060 |
| 1 | hsa_5143 | D01220 | 0 | hsa_6051 | D04245 | 0 | hsa_2339 | D08878 |
| 1 | hsa_5143 | D01630 | 0 | hsa_5972 | D10738 | 0 | hsa_2339 | D05744 |
| 1 | hsa_5143 | D02218 | 0 | hsa_5972 | D09960 | 0 | hsa_2339 | D09338 |
| 1 | hsa_5143 | D02985 | 0 | hsa_5972 | D10110 | 0 | hsa_2339 | D02985 |
| 1 | hsa_5143 | D04185 | 0 | hsa_5972 | D03225 | 0 | hsa_2339 | D04964 |
| 1 | hsa_5143 | D05474 | 0 | hsa_5972 | D07580 | 0 | hsa_2339 | D06634 |
| 1 | hsa_5143 | D05744 | 0 | hsa_5972 | D10465 | 0 | hsa_2339 | D10027 |
| 1 | hsa_5143 | D06132 | 0 | hsa_5972 | D01581 | 0 | hsa_2339 | D09689 |
| 1 | hsa_5143 | D06575 | 0 | hsa_5972 | D07556 | 0 | hsa_2339 | D03954 |
| 1 | hsa_5143 | D07088 | 0 | hsa_5972 | D10017 | 0 | hsa_2339 | D10183 |
| 1 | hsa_5143 | D07089 | 0 | hsa_5972 | D09689 | 0 | hsa_2339 | D10317 |
| 1 | hsa_5143 | D07425 | 0 | hsa_5972 | D00707 | 0 | hsa_2322 | D04793 |
| 1 | hsa_5143 | D07439 | 0 | hsa_5972 | D10721 | 0 | hsa_2322 | D09618 |
| 1 | hsa_5143 | D07879 | 0 | hsa_5972 | D03099 | 0 | hsa_2322 | D03661 |
| 1 | hsa_5143 | D07961 | 0 | hsa_5837 | D10674 | 0 | hsa_2321 | D09883 |
| 1 | hsa_5143 | D08238 | 0 | hsa_5837 | D07871 | 0 | hsa_2321 | D07260 |
| 1 | hsa_5143 | D08860 | 0 | hsa_5837 | D09676 | 0 | hsa_2321 | D10202 |
| 1 | hsa_5143 | D09020 | 0 | hsa_5837 | D03747 | 0 | hsa_2321 | D08854 |
| 1 | hsa_5142 | D00718 | 0 | hsa_5837 | D03241 | 0 | hsa_2321 | D08492 |
| 1 | hsa_5142 | D01220 | 0 | hsa_5837 | D09730 | 0 | hsa_2321 | D03378 |
| 1 | hsa_5142 | D01630 | 0 | hsa_5837 | D05598 | 0 | hsa_2321 | D09355 |
| 1 | hsa_5142 | D02218 | 0 | hsa_5836 | D06645 | 0 | hsa_2321 | D01581 |
| 1 | hsa_5142 | D02985 | 0 | hsa_5836 | D08085 | 0 | hsa_2321 | D03107 |
| 1 | hsa_5142 | D04185 | 0 | hsa_5836 | D10529 | 0 | hsa_2321 | D00469 |
| 1 | hsa_5142 | D05474 | 0 | hsa_5836 | D03260 | 0 | hsa_231 | D10619 |
| 1 | hsa_5142 | D05744 | 0 | hsa_5836 | D07992 | 0 | hsa_231 | D04685 |
| 1 | hsa_5142 | D06132 | 0 | hsa_5836 | D08363 | 0 | hsa_231 | D10189 |
| 1 | hsa_5142 | D06575 | 0 | hsa_5836 | D10086 | 0 | hsa_231 | D10138 |
| 1 | hsa_5142 | D07088 | 0 | hsa_5836 | D08854 | 0 | hsa_231 | D00939 |
| 1 | hsa_5142 | D07089 | 0 | hsa_5836 | D10137 | 0 | hsa_231 | D00428 |
| 1 | hsa_5142 | D07425 | 0 | hsa_5836 | D09783 | 0 | hsa_231 | D06881 |
| 1 | hsa_5142 | D07439 | 0 | hsa_5836 | D05502 | 0 | hsa_231 | D09347 |
| 1 | hsa_5142 | D07879 | 0 | hsa_5834 | D10317 | 0 | hsa_224 | D10178 |
| 1 | hsa_5142 | D07961 | 0 | hsa_5834 | D07698 | 0 | hsa_224 | D10154 |
| 1 | hsa_5142 | D08238 | 0 | hsa_5834 | D06881 | 0 | hsa_224 | D06413 |
| 1 | hsa_5142 | D08860 | 0 | hsa_5834 | D07941 | 0 | hsa_224 | D08880 |
| 1 | hsa_5142 | D09020 | 0 | hsa_5834 | D09622 | 0 | hsa_224 | D04014 |
| 1 | hsa_5141 | D00718 | 0 | hsa_5834 | D00749 | 0 | hsa_224 | D07781 |
| 1 | hsa_5141 | D01220 | 0 | hsa_5834 | D08354 | 0 | hsa_2224 | D10317 |
| 1 | hsa_5141 | D01630 | 0 | hsa_5834 | D10255 | 0 | hsa_2224 | D06503 |
| 1 | hsa_5141 | D02218 | 0 | hsa_5770 | D00321 | 0 | hsa_2224 | D10640 |
| 1 | hsa_5141 | D02985 | 0 | hsa_5770 | D08870 | 0 | hsa_2224 | D08469 |
| 1 | hsa_5141 | D04185 | 0 | hsa_5770 | D09756 | 0 | hsa_2224 | D08469 |
| 1 | hsa_5141 | D05474 | 0 | hsa_5770 | D08668 | 0 | hsa_2224 | D08149 |
| 1 | hsa_5141 | D05744 | 0 | hsa_5770 | D08953 | 0 | hsa_2224 | D10255 |
| 1 | hsa_5141 | D06132 | 0 | hsa_5770 | D05451 | 0 | hsa_219 | D07879 |
| 1 | hsa_5141 | D06575 | 0 | hsa_5743 | D06645 | 0 | hsa_219 | D06596 |
| 1 | hsa_5141 | D07088 | 0 | hsa_5743 | D08854 | 0 | hsa_219 | D06553 |
| 1 | hsa_5141 | D07089 | 0 | hsa_5743 | D10738 | 0 | hsa_219 | D07992 |
| 1 | hsa_5141 | D07425 | 0 | hsa_5743 | D01304 | 0 | hsa_219 | D09951 |
| 1 | hsa_5141 | D07439 | 0 | hsa_5743 | D10140 | 0 | hsa_219 | D07869 |
| 1 | hsa_5141 | D07879 | 0 | hsa_5743 | D08393 | 0 | hsa_219 | D10172 |
| 1 | hsa_5141 | D07961 | 0 | hsa_5743 | D09955 | 0 | hsa_219 | D10593 |
| 1 | hsa_5141 | D08238 | 0 | hsa_5743 | D01280 | 0 | hsa_219 | D05033 |
| 1 | hsa_5141 | D08860 | 0 | hsa_5743 | D08657 | 0 | hsa_219 | D03718 |
| 1 | hsa_5141 | D09020 | 0 | hsa_5743 | D01379 | 0 | hsa_219 | D10658 |
| 1 | hsa_5140 | D00718 | 0 | hsa_5742 | D08294 | 0 | hsa_219 | D09950 |
| 1 | hsa_5140 | D01220 | 0 | hsa_5742 | D07443 | 0 | hsa_219 | D09718 |
| 1 | hsa_5140 | D01630 | 0 | hsa_5742 | D06503 | 0 | hsa_2185 | D07144 |
| 1 | hsa_5140 | D01896 | 0 | hsa_5742 | D09955 | 0 | hsa_2185 | D07338 |
| 1 | hsa_5140 | D02084 | 0 | hsa_5742 | D07969 | 0 | hsa_2185 | D08913 |
| 1 | hsa_5140 | D02085 | 0 | hsa_5742 | D05744 | 0 | hsa_2185 | D10514 |
| 1 | hsa_5140 | D02218 | 0 | hsa_5742 | D10732 | 0 | hsa_2185 | D04185 |
| 1 | hsa_5140 | D04004 | 0 | hsa_5693 | D10138 | 0 | hsa_2185 | D01259 |
| 1 | hsa_5140 | D04508 | 0 | hsa_5693 | D09386 | 0 | hsa_2185 | D09639 |
| 1 | hsa_5140 | D04529 | 0 | hsa_5693 | D10551 | 0 | hsa_2185 | D08327 |
| 1 | hsa_5140 | D04628 | 0 | hsa_5693 | D09348 | 0 | hsa_2185 | D00564 |
| 1 | hsa_5140 | D04720 | 0 | hsa_5693 | D09338 | 0 | hsa_2185 | D03082 |
| 1 | hsa_5140 | D04751 | 0 | hsa_5693 | D03239 | 0 | hsa_2185 | D10481 |
| 1 | hsa_5140 | D06132 | 0 | hsa_5689 | D03021 | 0 | hsa_2185 | D08058 |
| 1 | hsa_5140 | D07089 | 0 | hsa_5689 | D10084 | 0 | hsa_217 | D08261 |
| 1 | hsa_5140 | D07425 | 0 | hsa_5689 | D01697 | 0 | hsa_217 | D09760 |
| 1 | hsa_5140 | D07439 | 0 | hsa_5689 | D09750 | 0 | hsa_217 | D09676 |
| 1 | hsa_5140 | D07961 | 0 | hsa_5689 | D08066 | 0 | hsa_217 | D08149 |
| 1 | hsa_5140 | D08238 | 0 | hsa_5689 | D08351 | 0 | hsa_217 | D08854 |
| 1 | hsa_5140 | D08294 | 0 | hsa_5689 | D00123 | 0 | hsa_217 | D07698 |
| 1 | hsa_5140 | D10255 | 0 | hsa_5689 | D00941 | 0 | hsa_217 | D06379 |
| 1 | hsa_5139 | D00231 | 0 | hsa_5689 | D10026 | 0 | hsa_217 | D02714 |
| 1 | hsa_5139 | D00417 | 0 | hsa_5689 | D10125 | 0 | hsa_217 | D03828 |
| 1 | hsa_5139 | D00718 | 0 | hsa_5689 | D04014 | 0 | hsa_217 | D08469 |
| 1 | hsa_5139 | D01133 | 0 | hsa_5689 | D07086 | 0 | hsa_217 | D10158 |
| 1 | hsa_5139 | D01198 | 0 | hsa_5689 | D08631 | 0 | hsa_217 | D10405 |
| 1 | hsa_5139 | D01220 | 0 | hsa_5689 | D10738 | 0 | hsa_217 | D09671 |
| 1 | hsa_5139 | D01630 | 0 | hsa_5689 | D03828 | 0 | hsa_2159 | D01572 |
| 1 | hsa_5139 | D01690 | 0 | hsa_5605 | D06596 | 0 | hsa_2159 | D09925 |
| 1 | hsa_5139 | D01896 | 0 | hsa_5605 | D07969 | 0 | hsa_2159 | D09923 |
| 1 | hsa_5139 | D02042 | 0 | hsa_5605 | D03954 | 0 | hsa_2159 | D06076 |
| 1 | hsa_5139 | D02084 | 0 | hsa_5605 | D05380 | 0 | hsa_2159 | D00341 |
| 1 | hsa_5139 | D02085 | 0 | hsa_5605 | D01379 | 0 | hsa_2159 | D09676 |
| 1 | hsa_5139 | D02218 | 0 | hsa_5605 | D10255 | 0 | hsa_2159 | D10183 |
| 1 | hsa_5139 | D04004 | 0 | hsa_5605 | D05589 | 0 | hsa_2159 | D08086 |
| 1 | hsa_5139 | D04508 | 0 | hsa_5604 | D06402 | 0 | hsa_2147 | D01972 |
| 1 | hsa_5139 | D04529 | 0 | hsa_5604 | D10688 | 0 | hsa_2147 | D03239 |
| 1 | hsa_5139 | D04628 | 0 | hsa_5604 | D08913 | 0 | hsa_2147 | D10656 |
| 1 | hsa_5139 | D04720 | 0 | hsa_5604 | D01090 | 0 | hsa_2147 | D09641 |
| 1 | hsa_5139 | D04751 | 0 | hsa_5604 | D05353 | 0 | hsa_2147 | D09893 |
| 1 | hsa_5139 | D06132 | 0 | hsa_5604 | D06578 | 0 | hsa_2147 | D07776 |
| 1 | hsa_5139 | D07089 | 0 | hsa_5604 | D09780 | 0 | hsa_2147 | D08953 |
| 1 | hsa_5139 | D07425 | 0 | hsa_5604 | D10079 | 0 | hsa_2147 | D04245 |
| 1 | hsa_5139 | D07439 | 0 | hsa_5604 | D09780 | 0 | hsa_2147 | D01840 |
| 1 | hsa_5139 | D07961 | 0 | hsa_5604 | D04486 | 0 | hsa_2147 | D07080 |
| 1 | hsa_5139 | D08238 | 0 | hsa_5604 | D09198 | 0 | hsa_2147 | D06575 |
| 1 | hsa_5139 | D08294 | 0 | hsa_5604 | D07119 | 0 | hsa_2147 | D09893 |
| 1 | hsa_5139 | D10255 | 0 | hsa_5604 | D04768 | 0 | hsa_208 | D07892 |
| 1 | hsa_5138 | D00718 | 0 | hsa_5603 | D09925 | 0 | hsa_208 | D01896 |
| 1 | hsa_5138 | D01220 | 0 | hsa_5603 | D01259 | 0 | hsa_208 | D06645 |
| 1 | hsa_5138 | D01630 | 0 | hsa_5603 | D00156 | 0 | hsa_208 | D09603 |
| 1 | hsa_5138 | D02218 | 0 | hsa_5603 | D07818 | 0 | hsa_208 | D10137 |
| 1 | hsa_5138 | D06132 | 0 | hsa_5602 | D03942 | 0 | hsa_208 | D10514 |
| 1 | hsa_5138 | D07089 | 0 | hsa_5602 | D07134 | 0 | hsa_208 | D04490 |
| 1 | hsa_5138 | D07425 | 0 | hsa_5602 | D08104 | 0 | hsa_208 | D09779 |
| 1 | hsa_5138 | D07439 | 0 | hsa_5602 | D07776 | 0 | hsa_208 | D10179 |
| 1 | hsa_5138 | D07961 | 0 | hsa_5602 | D09955 | 0 | hsa_208 | D08427 |
| 1 | hsa_5138 | D08238 | 0 | hsa_5602 | D04004 | 0 | hsa_208 | D10084 |
| 1 | hsa_5137 | D00718 | 0 | hsa_5602 | D04770 | 0 | hsa_207 | D08221 |
| 1 | hsa_5137 | D01220 | 0 | hsa_5602 | D06645 | 0 | hsa_207 | D10175 |
| 1 | hsa_5137 | D01630 | 0 | hsa_5602 | D08953 | 0 | hsa_207 | D09622 |
| 1 | hsa_5137 | D02218 | 0 | hsa_5602 | D03107 | 0 | hsa_207 | D05906 |
| 1 | hsa_5137 | D06132 | 0 | hsa_5601 | D06575 | 0 | hsa_207 | D01784 |
| 1 | hsa_5137 | D07089 | 0 | hsa_5601 | D09970 | 0 | hsa_207 | D09566 |
| 1 | hsa_5137 | D07425 | 0 | hsa_5601 | D10102 | 0 | hsa_2064 | D01545 |
| 1 | hsa_5137 | D07439 | 0 | hsa_5601 | D08463 | 0 | hsa_2064 | D07144 |
| 1 | hsa_5137 | D07961 | 0 | hsa_5601 | D10471 | 0 | hsa_2064 | D03225 |
| 1 | hsa_5137 | D08238 | 0 | hsa_5600 | D08689 | 0 | hsa_2064 | D01309 |
| 1 | hsa_5136 | D00718 | 0 | hsa_5600 | D09923 | 0 | hsa_2064 | D10437 |
| 1 | hsa_5136 | D01220 | 0 | hsa_5600 | D00355 | 0 | hsa_2064 | D01823 |
| 1 | hsa_5136 | D01630 | 0 | hsa_5600 | D00087 | 0 | hsa_2064 | D05033 |
| 1 | hsa_5136 | D02218 | 0 | hsa_5600 | D10431 | 0 | hsa_2064 | D10543 |
| 1 | hsa_5136 | D06132 | 0 | hsa_5599 | D07580 | 0 | hsa_200895 | D10222 |
| 1 | hsa_5136 | D07089 | 0 | hsa_5599 | D00944 | 0 | hsa_200895 | D10471 |
| 1 | hsa_5136 | D07425 | 0 | hsa_5599 | D03010 | 0 | hsa_200895 | D07141 |
| 1 | hsa_5136 | D07439 | 0 | hsa_5599 | D09689 | 0 | hsa_200895 | D03234 |
| 1 | hsa_5136 | D07961 | 0 | hsa_5599 | D05511 | 0 | hsa_200895 | D10137 |
| 1 | hsa_5136 | D08238 | 0 | hsa_5599 | D07499 | 0 | hsa_200895 | D10585 |
| 1 | hsa_50940 | D00718 | 0 | hsa_5599 | D10125 | 0 | hsa_200895 | D08102 |
| 1 | hsa_50940 | D01220 | 0 | hsa_5595 | D06503 | 0 | hsa_200895 | D04791 |
| 1 | hsa_50940 | D01630 | 0 | hsa_5595 | D00749 | 0 | hsa_200895 | D07472 |
| 1 | hsa_50940 | D02218 | 0 | hsa_5595 | D01304 | 0 | hsa_196883 | D09915 |
| 1 | hsa_50940 | D06132 | 0 | hsa_5595 | D06413 | 0 | hsa_196883 | D08516 |
| 1 | hsa_50940 | D07089 | 0 | hsa_5595 | D10515 | 0 | hsa_196883 | D04936 |
| 1 | hsa_50940 | D07425 | 0 | hsa_5594 | D10140 | 0 | hsa_196883 | D03241 |
| 1 | hsa_50940 | D07439 | 0 | hsa_5594 | D06005 | 0 | hsa_1956 | D04508 |
| 1 | hsa_50940 | D07961 | 0 | hsa_5594 | D05474 | 0 | hsa_1956 | D04685 |
| 1 | hsa_50940 | D08238 | 0 | hsa_5594 | D08552 | 0 | hsa_1956 | D04768 |
| 1 | hsa_50487 | D06283 | 0 | hsa_5594 | D03349 | 0 | hsa_1956 | D03234 |
| 1 | hsa_50487 | D08107 | 0 | hsa_5588 | D08131 | 0 | hsa_1956 | D05744 |
| 1 | hsa_50487 | D08221 | 0 | hsa_5588 | D09355 | 0 | hsa_1956 | D03248 |
| 1 | hsa_50484 | D03546 | 0 | hsa_5588 | D10728 | 0 | hsa_1956 | D09689 |
| 1 | hsa_5033 | D04793 | 0 | hsa_5588 | D05893 | 0 | hsa_1956 | D01309 |
| 1 | hsa_495 | D00355 | 0 | hsa_5588 | D10641 | 0 | hsa_1813 | D10733 |
| 1 | hsa_495 | D01920 | 0 | hsa_5588 | D08004 | 0 | hsa_1813 | D08102 |
| 1 | hsa_495 | D05353 | 0 | hsa_5588 | D01823 | 0 | hsa_1813 | D00152 |
| 1 | hsa_495 | D05900 | 0 | hsa_5588 | D10137 | 0 | hsa_1813 | D04936 |
| 1 | hsa_495 | D05901 | 0 | hsa_5588 | D06285 | 0 | hsa_1813 | D06413 |
| 1 | hsa_495 | D05906 | 0 | hsa_5588 | D03942 | 0 | hsa_1813 | D04276 |
| 1 | hsa_495 | D07917 | 0 | hsa_5588 | D09881 | 0 | hsa_1813 | D08354 |
| 1 | hsa_495 | D08463 | 0 | hsa_5588 | D08657 | 0 | hsa_1813 | D05901 |
| 1 | hsa_495 | D08903 | 0 | hsa_5588 | D10630 | 0 | hsa_1813 | D02755 |
| 1 | hsa_489 | D10715 | 0 | hsa_55869 | D03234 | 0 | hsa_1813 | D05598 |
| 1 | hsa_488 | D10715 | 0 | hsa_55869 | D09959 | 0 | hsa_1813 | D10568 |
| 1 | hsa_487 | D10715 | 0 | hsa_55869 | D08963 | 0 | hsa_1813 | D01070 |
| 1 | hsa_4860 | D04245 | 0 | hsa_55869 | D03602 | 0 | hsa_1813 | D02365 |
| 1 | hsa_4860 | D06596 | 0 | hsa_55869 | D10179 | 0 | hsa_1813 | D04508 |
| 1 | hsa_4860 | D10431 | 0 | hsa_55869 | D10318 | 0 | hsa_1813 | D10365 |
| 1 | hsa_4860 | D10432 | 0 | hsa_55869 | D10659 | 0 | hsa_1806 | D05177 |
| 1 | hsa_4846 | D09018 | 0 | hsa_55869 | D10405 | 0 | hsa_1806 | D04822 |
| 1 | hsa_4843 | D09018 | 0 | hsa_55869 | D01404 | 0 | hsa_1806 | D01823 |
| 1 | hsa_4843 | D10386 | 0 | hsa_55869 | D03546 | 0 | hsa_1806 | D00123 |
| 1 | hsa_4843 | D10419 | 0 | hsa_55869 | D05893 | 0 | hsa_1806 | D08883 |
| 1 | hsa_4842 | D09018 | 0 | hsa_5583 | D09728 | 0 | hsa_1806 | D07661 |
| 1 | hsa_483 | D00112 | 0 | hsa_5583 | D05177 | 0 | hsa_1806 | D10657 |
| 1 | hsa_483 | D00297 | 0 | hsa_5583 | D10432 | 0 | hsa_1806 | D03745 |
| 1 | hsa_483 | D00298 | 0 | hsa_5583 | D09386 | 0 | hsa_1806 | D06637 |
| 1 | hsa_483 | D01240 | 0 | hsa_5583 | D05032 | 0 | hsa_1806 | D04696 |
| 1 | hsa_483 | D01379 | 0 | hsa_5583 | D09622 | 0 | hsa_1806 | D07499 |
| 1 | hsa_483 | D01972 | 0 | hsa_55811 | D01572 | 0 | hsa_1806 | D10551 |
| 1 | hsa_483 | D02587 | 0 | hsa_55811 | D07281 | 0 | hsa_1803 | D08903 |
| 1 | hsa_483 | D06881 | 0 | hsa_55811 | D00181 | 0 | hsa_1803 | D03786 |
| 1 | hsa_483 | D07147 | 0 | hsa_55811 | D02206 | 0 | hsa_1803 | D03217 |
| 1 | hsa_483 | D07555 | 0 | hsa_55811 | D09730 | 0 | hsa_1803 | D05353 |
| 1 | hsa_483 | D07556 | 0 | hsa_55811 | D05893 | 0 | hsa_1803 | D07879 |
| 1 | hsa_483 | D09847 | 0 | hsa_55811 | D01823 | 0 | hsa_1803 | D10202 |
| 1 | hsa_482 | D00112 | 0 | hsa_5581 | D05181 | 0 | hsa_1800 | D00152 |
| 1 | hsa_482 | D00297 | 0 | hsa_5581 | D03665 | 0 | hsa_1800 | D07941 |
| 1 | hsa_482 | D00298 | 0 | hsa_5581 | D06596 | 0 | hsa_1800 | D10372 |
| 1 | hsa_482 | D01240 | 0 | hsa_5581 | D10381 | 0 | hsa_1800 | D07940 |
| 1 | hsa_482 | D01379 | 0 | hsa_5581 | D09604 | 0 | hsa_1800 | D04964 |
| 1 | hsa_482 | D01972 | 0 | hsa_5581 | D06285 | 0 | hsa_1800 | D07144 |
| 1 | hsa_482 | D02587 | 0 | hsa_5581 | D10085 | 0 | hsa_1800 | D09348 |
| 1 | hsa_482 | D06881 | 0 | hsa_5581 | D05181 | 0 | hsa_1789 | D10691 |
| 1 | hsa_482 | D07147 | 0 | hsa_5580 | D07917 | 0 | hsa_1789 | D03409 |
| 1 | hsa_482 | D07555 | 0 | hsa_5580 | D09960 | 0 | hsa_1789 | D03793 |
| 1 | hsa_482 | D07556 | 0 | hsa_5580 | D07974 | 0 | hsa_1789 | D07136 |
| 1 | hsa_482 | D09847 | 0 | hsa_5580 | D10146 | 0 | hsa_1789 | D07499 |
| 1 | hsa_481 | D00112 | 0 | hsa_5580 | D09951 | 0 | hsa_1789 | D06320 |
| 1 | hsa_481 | D00297 | 0 | hsa_5580 | D10730 | 0 | hsa_1789 | D06379 |
| 1 | hsa_481 | D00298 | 0 | hsa_5580 | D00753 | 0 | hsa_1789 | D01972 |
| 1 | hsa_481 | D01240 | 0 | hsa_5580 | D08107 | 0 | hsa_1789 | D10154 |
| 1 | hsa_481 | D01379 | 0 | hsa_5580 | D01155 | 0 | hsa_1789 | D01134 |
| 1 | hsa_481 | D01972 | 0 | hsa_5580 | D05094 | 0 | hsa_1788 | D05019 |
| 1 | hsa_481 | D02587 | 0 | hsa_5579 | D00355 | 0 | hsa_1788 | D10223 |
| 1 | hsa_481 | D06881 | 0 | hsa_5579 | D08108 | 0 | hsa_1788 | D00123 |
| 1 | hsa_481 | D07147 | 0 | hsa_5579 | D09639 | 0 | hsa_1788 | D09666 |
| 1 | hsa_481 | D07555 | 0 | hsa_5579 | D10224 | 0 | hsa_1788 | D04014 |
| 1 | hsa_481 | D07556 | 0 | hsa_5579 | D04793 | 0 | hsa_1788 | D06881 |
| 1 | hsa_481 | D09847 | 0 | hsa_5563 | D06239 | 0 | hsa_1788 | D07871 |
| 1 | hsa_480 | D00112 | 0 | hsa_5563 | D09671 | 0 | hsa_1788 | D06378 |
| 1 | hsa_480 | D00297 | 0 | hsa_5563 | D10024 | 0 | hsa_1788 | D10076 |
| 1 | hsa_480 | D00298 | 0 | hsa_5563 | D10386 | 0 | hsa_1788 | D09355 |
| 1 | hsa_480 | D01240 | 0 | hsa_5563 | D00355 | 0 | hsa_1788 | D06132 |
| 1 | hsa_480 | D01379 | 0 | hsa_5563 | D10641 | 0 | hsa_1786 | D03239 |
| 1 | hsa_480 | D01972 | 0 | hsa_5563 | D08238 | 0 | hsa_1786 | D05590 |
| 1 | hsa_480 | D02587 | 0 | hsa_5563 | D05380 | 0 | hsa_1786 | D02755 |
| 1 | hsa_480 | D06881 | 0 | hsa_5562 | D10619 | 0 | hsa_1786 | D08917 |
| 1 | hsa_480 | D07147 | 0 | hsa_5562 | D05480 | 0 | hsa_1786 | D10715 |
| 1 | hsa_480 | D07555 | 0 | hsa_5562 | D03845 | 0 | hsa_1723 | D03252 |
| 1 | hsa_480 | D07556 | 0 | hsa_5562 | D00428 | 0 | hsa_1723 | D03260 |
| 1 | hsa_480 | D09847 | 0 | hsa_5562 | D03661 | 0 | hsa_1723 | D01846 |
| 1 | hsa_478 | D00112 | 0 | hsa_5562 | D10191 | 0 | hsa_1723 | D06387 |
| 1 | hsa_478 | D00297 | 0 | hsa_5562 | D05819 | 0 | hsa_1723 | D09327 |
| 1 | hsa_478 | D00298 | 0 | hsa_5562 | D03820 | 0 | hsa_1723 | D01841 |
| 1 | hsa_478 | D01240 | 0 | hsa_5535 | D10255 | 0 | hsa_1723 | D07581 |
| 1 | hsa_478 | D01379 | 0 | hsa_5535 | D03517 | 0 | hsa_1723 | D07088 |
| 1 | hsa_478 | D01972 | 0 | hsa_5535 | D05019 | 0 | hsa_1723 | D03107 |
| 1 | hsa_478 | D02587 | 0 | hsa_5535 | D05033 | 0 | hsa_1723 | D10641 |
| 1 | hsa_478 | D06881 | 0 | hsa_5535 | D04936 | 0 | hsa_1719 | D05143 |
| 1 | hsa_478 | D07147 | 0 | hsa_5535 | D08294 | 0 | hsa_1719 | D05143 |
| 1 | hsa_478 | D07555 | 0 | hsa_5535 | D07698 | 0 | hsa_1719 | D05143 |
| 1 | hsa_478 | D07556 | 0 | hsa_5535 | D10732 | 0 | hsa_1719 | D08349 |
| 1 | hsa_478 | D09847 | 0 | hsa_5535 | D09817 | 0 | hsa_1719 | D07901 |
| 1 | hsa_477 | D00112 | 0 | hsa_5535 | D07879 | 0 | hsa_1719 | D07555 |
| 1 | hsa_477 | D00297 | 0 | hsa_5535 | D03158 | 0 | hsa_1719 | D07992 |
| 1 | hsa_477 | D00298 | 0 | hsa_5535 | D01155 | 0 | hsa_1719 | D08996 |
| 1 | hsa_477 | D01240 | 0 | hsa_5535 | D06387 | 0 | hsa_1719 | D03546 |
| 1 | hsa_477 | D01379 | 0 | hsa_5535 | D09347 | 0 | hsa_169355 | D09724 |
| 1 | hsa_477 | D01972 | 0 | hsa_5535 | D08085 | 0 | hsa_169355 | D08094 |
| 1 | hsa_477 | D02587 | 0 | hsa_5535 | D08062 | 0 | hsa_169355 | D00341 |
| 1 | hsa_477 | D06881 | 0 | hsa_5535 | D03208 | 0 | hsa_169355 | D08880 |
| 1 | hsa_477 | D07147 | 0 | hsa_5535 | D01070 | 0 | hsa_169355 | D06068 |
| 1 | hsa_477 | D07555 | 0 | hsa_5534 | D01392 | 0 | hsa_169355 | D04768 |
| 1 | hsa_477 | D07556 | 0 | hsa_5534 | D03217 | 0 | hsa_169355 | D09718 |
| 1 | hsa_477 | D09847 | 0 | hsa_5534 | D09357 | 0 | hsa_169355 | D09678 |
| 1 | hsa_476 | D00112 | 0 | hsa_5534 | D08085 | 0 | hsa_169355 | D09602 |
| 1 | hsa_476 | D00297 | 0 | hsa_5534 | D10696 | 0 | hsa_169355 | D10688 |
| 1 | hsa_476 | D01379 | 0 | hsa_5534 | D01267 | 0 | hsa_169355 | D02755 |
| 1 | hsa_476 | D01972 | 0 | hsa_5534 | D03158 | 0 | hsa_169355 | D00073 |
| 1 | hsa_476 | D02587 | 0 | hsa_5533 | D07940 | 0 | hsa_1644 | D10090 |
| 1 | hsa_476 | D06881 | 0 | hsa_5533 | D09950 | 0 | hsa_1644 | D03929 |
| 1 | hsa_476 | D07147 | 0 | hsa_5533 | D08349 | 0 | hsa_1644 | D01896 |
| 1 | hsa_476 | D07555 | 0 | hsa_5533 | D08392 | 0 | hsa_1644 | D06414 |
| 1 | hsa_476 | D07556 | 0 | hsa_5533 | D04185 | 0 | hsa_1644 | D10334 |
| 1 | hsa_476 | D09847 | 0 | hsa_5533 | D08556 | 0 | hsa_1644 | D10060 |
| 1 | hsa_47 | D10691 | 0 | hsa_5532 | D10104 | 0 | hsa_1644 | D10076 |
| 1 | hsa_4322 | D03797 | 0 | hsa_5532 | D10229 | 0 | hsa_1644 | D06609 |
| 1 | hsa_4313 | D03061 | 0 | hsa_5532 | D07941 | 0 | hsa_1644 | D07577 |
| 1 | hsa_4313 | D03793 | 0 | hsa_5532 | D09756 | 0 | hsa_1644 | D07817 |
| 1 | hsa_4313 | D03802 | 0 | hsa_5532 | D05096 | 0 | hsa_1644 | D03715 |
| 1 | hsa_4312 | D03517 | 0 | hsa_5532 | D10223 | 0 | hsa_1644 | D03828 |
| 1 | hsa_4311 | D01070 | 0 | hsa_5532 | D09756 | 0 | hsa_1636 | D08393 |
| 1 | hsa_4311 | D03349 | 0 | hsa_5532 | D09566 | 0 | hsa_1636 | D10709 |
| 1 | hsa_4311 | D03929 | 0 | hsa_5532 | D08344 | 0 | hsa_1636 | D00942 |
| 1 | hsa_43 | D00469 | 0 | hsa_5532 | D02985 | 0 | hsa_1636 | D03021 |
| 1 | hsa_43 | D01572 | 0 | hsa_5530 | D05399 | 0 | hsa_1636 | D04751 |
| 1 | hsa_43 | D02565 | 0 | hsa_5530 | D08913 | 0 | hsa_1636 | D10076 |
| 1 | hsa_43 | D03099 | 0 | hsa_5530 | D10060 | 0 | hsa_1636 | D10568 |
| 1 | hsa_43 | D03239 | 0 | hsa_5530 | D10399 | 0 | hsa_1636 | D02084 |
| 1 | hsa_43 | D05215 | 0 | hsa_5530 | D06606 | 0 | hsa_1636 | D03802 |
| 1 | hsa_43 | D05590 | 0 | hsa_5530 | D10658 | 0 | hsa_1588 | D07888 |
| 1 | hsa_43 | D06288 | 0 | hsa_5530 | D08024 | 0 | hsa_1588 | D07136 |
| 1 | hsa_43 | D07869 | 0 | hsa_5530 | D01672 | 0 | hsa_1588 | D07892 |
| 1 | hsa_43 | D08261 | 0 | hsa_5530 | D10661 | 0 | hsa_1588 | D05819 |
| 1 | hsa_43 | D08555 | 0 | hsa_5530 | D10223 | 0 | hsa_1588 | D07541 |
| 1 | hsa_43 | D09750 | 0 | hsa_54583 | D06132 | 0 | hsa_1588 | D03217 |
| 1 | hsa_43 | D10529 | 0 | hsa_54583 | D09333 | 0 | hsa_1588 | D07499 |
| 1 | hsa_4233 | D10173 | 0 | hsa_54583 | D04964 | 0 | hsa_1588 | D00564 |
| 1 | hsa_4233 | D10224 | 0 | hsa_54583 | D08900 | 0 | hsa_1588 | D07698 |
| 1 | hsa_4233 | D10465 | 0 | hsa_54583 | D06283 | 0 | hsa_1586 | D09678 |
| 1 | hsa_4233 | D10696 | 0 | hsa_54583 | D08618 | 0 | hsa_1586 | D05029 |
| 1 | hsa_4129 | D01304 | 0 | hsa_54583 | D08364 | 0 | hsa_1586 | D09664 |
| 1 | hsa_4129 | D03409 | 0 | hsa_54583 | D08616 | 0 | hsa_1586 | D10157 |
| 1 | hsa_4129 | D04092 | 0 | hsa_54583 | D03241 | 0 | hsa_1586 | D05502 |
| 1 | hsa_4129 | D04681 | 0 | hsa_54583 | D01379 | 0 | hsa_1586 | D04498 |
| 1 | hsa_4129 | D05033 | 0 | hsa_54583 | D07082 | 0 | hsa_1586 | D09955 |
| 1 | hsa_4129 | D07337 | 0 | hsa_54583 | D06132 | 0 | hsa_1584 | D08529 |
| 1 | hsa_4129 | D07338 | 0 | hsa_54583 | D09666 | 0 | hsa_1584 | D08351 |
| 1 | hsa_4129 | D08085 | 0 | hsa_54583 | D08917 | 0 | hsa_1584 | D06283 |
| 1 | hsa_4129 | D08349 | 0 | hsa_54583 | D10674 | 0 | hsa_1584 | D05033 |
| 1 | hsa_4129 | D08453 | 0 | hsa_54583 | D02565 | 0 | hsa_1577 | D10551 |
| 1 | hsa_4129 | D08469 | 0 | hsa_54583 | D09020 | 0 | hsa_1577 | D09756 |
| 1 | hsa_4129 | D08625 | 0 | hsa_5347 | D05511 | 0 | hsa_1577 | D09989 |
| 1 | hsa_4129 | D10158 | 0 | hsa_5347 | D05319 | 0 | hsa_1577 | D08552 |
| 1 | hsa_4129 | D10191 | 0 | hsa_5347 | D05502 | 0 | hsa_1577 | D03154 |
| 1 | hsa_4128 | D01304 | 0 | hsa_5347 | D05511 | 0 | hsa_1577 | D06606 |
| 1 | hsa_4128 | D03248 | 0 | hsa_5347 | D03786 | 0 | hsa_1577 | D03929 |
| 1 | hsa_4128 | D03409 | 0 | hsa_5347 | D10619 | 0 | hsa_1577 | D04023 |
| 1 | hsa_4128 | D04092 | 0 | hsa_5347 | D10076 | 0 | hsa_1577 | D06272 |
| 1 | hsa_4128 | D07337 | 0 | hsa_5347 | D04004 | 0 | hsa_1576 | D09707 |
| 1 | hsa_4128 | D07338 | 0 | hsa_5347 | D08094 | 0 | hsa_1576 | D07776 |
| 1 | hsa_4128 | D08085 | 0 | hsa_5347 | D07892 | 0 | hsa_1576 | D03990 |
| 1 | hsa_4128 | D08349 | 0 | hsa_5347 | D08668 | 0 | hsa_1576 | D07131 |
| 1 | hsa_4128 | D08392 | 0 | hsa_5347 | D03652 | 0 | hsa_1576 | D09666 |
| 1 | hsa_4128 | D08393 | 0 | hsa_5347 | D03248 | 0 | hsa_1576 | D07131 |
| 1 | hsa_4128 | D08453 | 0 | hsa_5322 | D05474 | 0 | hsa_1576 | D06076 |
| 1 | hsa_4128 | D08625 | 0 | hsa_5322 | D04685 | 0 | hsa_1576 | D08503 |
| 1 | hsa_412 | D09915 | 0 | hsa_5322 | D07142 | 0 | hsa_1576 | D10688 |
| 1 | hsa_4047 | D06609 | 0 | hsa_5322 | D09033 | 0 | hsa_1576 | D03150 |
| 1 | hsa_4023 | D03747 | 0 | hsa_5322 | D06379 | 0 | hsa_1576 | D01309 |
| 1 | hsa_391013 | D06283 | 0 | hsa_5322 | D01630 | 0 | hsa_1551 | D00420 |
| 1 | hsa_391013 | D08107 | 0 | hsa_5322 | D07338 | 0 | hsa_1551 | D10224 |
| 1 | hsa_391013 | D08221 | 0 | hsa_5322 | D08688 | 0 | hsa_1551 | D09920 |
| 1 | hsa_3818 | D01672 | 0 | hsa_5322 | D05744 | 0 | hsa_1551 | D07869 |
| 1 | hsa_3818 | D08004 | 0 | hsa_5321 | D10102 | 0 | hsa_1551 | D09667 |
| 1 | hsa_3815 | D08503 | 0 | hsa_5321 | D08556 | 0 | hsa_1551 | D10652 |
| 1 | hsa_3815 | D08544 | 0 | hsa_5321 | D04245 | 0 | hsa_1551 | D08104 |
| 1 | hsa_3815 | D10229 | 0 | hsa_5321 | D01896 | 0 | hsa_1551 | D10024 |
| 1 | hsa_3791 | D08878 | 0 | hsa_5321 | D09753 | 0 | hsa_1551 | D10027 |
| 1 | hsa_3791 | D09589 | 0 | hsa_5321 | D09893 | 0 | hsa_1551 | D07337 |
| 1 | hsa_3791 | D09618 | 0 | hsa_5321 | D07888 | 0 | hsa_1551 | D02714 |
| 1 | hsa_377677 | D01822 | 0 | hsa_5321 | D06402 | 0 | hsa_1551 | D03749 |
| 1 | hsa_377677 | D02441 | 0 | hsa_5321 | D10176 | 0 | hsa_1551 | D09817 |
| 1 | hsa_377677 | D03845 | 0 | hsa_5321 | D03954 | 0 | hsa_1513 | D09956 |
| 1 | hsa_377677 | D07871 | 0 | hsa_5321 | D07519 | 0 | hsa_1513 | D03248 |
| 1 | hsa_3718 | D09783 | 0 | hsa_5321 | D07516 | 0 | hsa_1513 | D09920 |
| 1 | hsa_3718 | D09970 | 0 | hsa_5321 | D10405 | 0 | hsa_1513 | D10191 |
| 1 | hsa_3718 | D10308 | 0 | hsa_5320 | D07888 | 0 | hsa_1513 | D00073 |
| 1 | hsa_3718 | D10585 | 0 | hsa_5320 | D01630 | 0 | hsa_1513 | D00564 |
| 1 | hsa_3718 | D10653 | 0 | hsa_5320 | D09728 | 0 | hsa_1513 | D09679 |
| 1 | hsa_3718 | D10721 | 0 | hsa_5320 | D10674 | 0 | hsa_1513 | D10223 |
| 1 | hsa_3717 | D10308 | 0 | hsa_5320 | D10154 | 0 | hsa_1513 | D10696 |
| 1 | hsa_3717 | D10365 | 0 | hsa_5320 | D04966 | 0 | hsa_1513 | D10560 |
| 1 | hsa_3717 | D10630 | 0 | hsa_5319 | D10146 | 0 | hsa_1513 | D03828 |
| 1 | hsa_3717 | D10653 | 0 | hsa_5319 | D08871 | 0 | hsa_1445 | D10179 |
| 1 | hsa_3717 | D10721 | 0 | hsa_5319 | D09970 | 0 | hsa_1445 | D00123 |
| 1 | hsa_3716 | D09959 | 0 | hsa_5319 | D00073 | 0 | hsa_1445 | D03225 |
| 1 | hsa_3716 | D09960 | 0 | hsa_5319 | D03938 | 0 | hsa_1445 | D04751 |
| 1 | hsa_3716 | D10308 | 0 | hsa_5319 | D01304 | 0 | hsa_1445 | D05215 |
| 1 | hsa_3716 | D10315 | 0 | hsa_5319 | D07135 | 0 | hsa_1445 | D09783 |
| 1 | hsa_3716 | D10358 | 0 | hsa_5319 | D04050 | 0 | hsa_1445 | D04092 |
| 1 | hsa_3716 | D10653 | 0 | hsa_5319 | D08349 | 0 | hsa_1435 | D07577 |
| 1 | hsa_3716 | D10721 | 0 | hsa_5319 | D07917 | 0 | hsa_1435 | D06596 |
| 1 | hsa_3716 | D10728 | 0 | hsa_5319 | D09925 | 0 | hsa_1435 | D08068 |
| 1 | hsa_3620 | D10640 | 0 | hsa_5319 | D09707 | 0 | hsa_1435 | D03239 |
| 1 | hsa_3615 | D01392 | 0 | hsa_5294 | D03786 | 0 | hsa_1435 | D09756 |
| 1 | hsa_3615 | D04936 | 0 | hsa_5294 | D08162 | 0 | hsa_1435 | D07135 |
| 1 | hsa_3615 | D05094 | 0 | hsa_5294 | D08631 | 0 | hsa_1435 | D10715 |
| 1 | hsa_3615 | D05095 | 0 | hsa_5294 | D07425 | 0 | hsa_1435 | D02218 |
| 1 | hsa_3615 | D05096 | 0 | hsa_5294 | D00558 | 0 | hsa_1435 | D07901 |
| 1 | hsa_3614 | D01392 | 0 | hsa_5294 | D01244 | 0 | hsa_1435 | D02729 |
| 1 | hsa_3614 | D04936 | 0 | hsa_5294 | D07661 | 0 | hsa_1432 | D07969 |
| 1 | hsa_3614 | D05094 | 0 | hsa_5294 | D03658 | 0 | hsa_1432 | D08616 |
| 1 | hsa_3614 | D05095 | 0 | hsa_5294 | D09679 | 0 | hsa_1432 | D07425 |
| 1 | hsa_3614 | D05096 | 0 | hsa_5294 | D06402 | 0 | hsa_1432 | D07615 |
| 1 | hsa_3480 | D09925 | 0 | hsa_5294 | D08224 | 0 | hsa_1432 | D10652 |
| 1 | hsa_3290 | D00156 | 0 | hsa_5293 | D04720 | 0 | hsa_1432 | D08352 |
| 1 | hsa_3290 | D01899 | 0 | hsa_5293 | D06678 | 0 | hsa_1432 | D04681 |
| 1 | hsa_3290 | D07615 | 0 | hsa_5293 | D09033 | 0 | hsa_143 | D03099 |
| 1 | hsa_3242 | D05177 | 0 | hsa_5293 | D06132 | 0 | hsa_143 | D09780 |
| 1 | hsa_3156 | D07474 | 0 | hsa_5293 | D00558 | 0 | hsa_143 | D10656 |
| 1 | hsa_3156 | D07661 | 0 | hsa_5293 | D01630 | 0 | hsa_143 | D08616 |
| 1 | hsa_3156 | D07983 | 0 | hsa_5293 | D06068 | 0 | hsa_143 | D09779 |
| 1 | hsa_3156 | D08410 | 0 | hsa_5293 | D08668 | 0 | hsa_143 | D00718 |
| 1 | hsa_3156 | D08492 | 0 | hsa_5293 | D08913 | 0 | hsa_143 | D06553 |
| 1 | hsa_3156 | D10568 | 0 | hsa_5293 | D10381 | 0 | hsa_143 | D06575 |
| 1 | hsa_30814 | D06283 | 0 | hsa_5291 | D10102 | 0 | hsa_143 | D03990 |
| 1 | hsa_30814 | D08107 | 0 | hsa_5291 | D05353 | 0 | hsa_143 | D05900 |
| 1 | hsa_30814 | D08221 | 0 | hsa_5291 | D04696 | 0 | hsa_142 | D08073 |
| 1 | hsa_3067 | D03158 | 0 | hsa_5291 | D10399 | 0 | hsa_142 | D09326 |
| 1 | hsa_3066 | D06637 | 0 | hsa_5291 | D08344 | 0 | hsa_142 | D07123 |
| 1 | hsa_3066 | D08870 | 0 | hsa_5290 | D10223 | 0 | hsa_142 | D10738 |
| 1 | hsa_3066 | D10019 | 0 | hsa_5290 | D03954 | 0 | hsa_142 | D09883 |
| 1 | hsa_3066 | D10060 | 0 | hsa_5290 | D09913 | 0 | hsa_142 | D01653 |
| 1 | hsa_3066 | D10084 | 0 | hsa_5290 | D07582 | 0 | hsa_142 | D00073 |
| 1 | hsa_3066 | D10319 | 0 | hsa_5290 | D10721 | 0 | hsa_142 | D09970 |
| 1 | hsa_3065 | D06320 | 0 | hsa_5290 | D04964 | 0 | hsa_1375 | D03409 |
| 1 | hsa_3065 | D06637 | 0 | hsa_5290 | D00123 | 0 | hsa_1375 | D05400 |
| 1 | hsa_3065 | D08870 | 0 | hsa_5290 | D10515 | 0 | hsa_1375 | D10399 |
| 1 | hsa_3065 | D09338 | 0 | hsa_5290 | D09618 | 0 | hsa_1375 | D07698 |
| 1 | hsa_3065 | D09357 | 0 | hsa_5290 | D03661 | 0 | hsa_1375 | D09348 |
| 1 | hsa_3065 | D09641 | 0 | hsa_5290 | D01134 | 0 | hsa_1375 | D07582 |
| 1 | hsa_3065 | D10019 | 0 | hsa_5290 | D07137 | 0 | hsa_1375 | D04014 |
| 1 | hsa_3065 | D10060 | 0 | hsa_5290 | D08996 | 0 | hsa_1375 | D04529 |
| 1 | hsa_3065 | D10084 | 0 | hsa_5290 | D10229 | 0 | hsa_1375 | D04793 |
| 1 | hsa_3065 | D10319 | 0 | hsa_5290 | D10365 | 0 | hsa_1375 | D10130 |
| 1 | hsa_29947 | D03021 | 0 | hsa_5289 | D07579 | 0 | hsa_1374 | D09955 |
| 1 | hsa_29947 | D03665 | 0 | hsa_5289 | D08327 | 0 | hsa_1374 | D08056 |
| 1 | hsa_2984 | D06612 | 0 | hsa_5289 | D07819 | 0 | hsa_1374 | D02084 |
| 1 | hsa_2984 | D09355 | 0 | hsa_5289 | D08073 | 0 | hsa_1374 | D01972 |
| 1 | hsa_2983 | D07577 | 0 | hsa_5289 | D00944 | 0 | hsa_1374 | D00941 |
| 1 | hsa_2983 | D09572 | 0 | hsa_5289 | D09664 | 0 | hsa_1374 | D03239 |
| 1 | hsa_2982 | D07577 | 0 | hsa_5289 | D09707 | 0 | hsa_1374 | D03213 |
| 1 | hsa_2982 | D09572 | 0 | hsa_5243 | D09893 | 0 | hsa_1374 | D10515 |
| 1 | hsa_2977 | D07577 | 0 | hsa_5243 | D04696 | 0 | hsa_1374 | D10076 |
| 1 | hsa_2977 | D09572 | 0 | hsa_5243 | D10661 | 0 | hsa_1374 | D08963 |
| 1 | hsa_2950 | D08917 | 0 | hsa_5243 | D08524 | 0 | hsa_1374 | D08453 |
| 1 | hsa_290 | D10026 | 0 | hsa_5243 | D06283 | 0 | hsa_1312 | D05893 |
| 1 | hsa_283208 | D04793 | 0 | hsa_5243 | D01630 | 0 | hsa_1312 | D07699 |
| 1 | hsa_27115 | D00718 | 0 | hsa_5243 | D00428 | 0 | hsa_1312 | D06402 |
| 1 | hsa_27115 | D01220 | 0 | hsa_5243 | D10168 | 0 | hsa_1312 | D10738 |
| 1 | hsa_27115 | D01630 | 0 | hsa_5243 | D07086 | 0 | hsa_1312 | D04964 |
| 1 | hsa_27115 | D02218 | 0 | hsa_5243 | D04245 | 0 | hsa_1312 | D09847 |
| 1 | hsa_27115 | D06132 | 0 | hsa_5243 | D09618 | 0 | hsa_1312 | D07439 |
| 1 | hsa_27115 | D07089 | 0 | hsa_5243 | D05095 | 0 | hsa_131 | D01259 |
| 1 | hsa_27115 | D07425 | 0 | hsa_5243 | D07269 | 0 | hsa_131 | D10103 |
| 1 | hsa_27115 | D07439 | 0 | hsa_5243 | D10155 | 0 | hsa_131 | D09676 |
| 1 | hsa_27115 | D07961 | 0 | hsa_5159 | D07541 | 0 | hsa_131 | D01155 |
| 1 | hsa_27115 | D08238 | 0 | hsa_5159 | D03845 | 0 | hsa_131 | D07269 |
| 1 | hsa_2687 | D02755 | 0 | hsa_5159 | D09677 | 0 | hsa_131 | D00355 |
| 1 | hsa_2686 | D02755 | 0 | hsa_5159 | D10223 | 0 | hsa_131 | D00151 |
| 1 | hsa_2678 | D02755 | 0 | hsa_5159 | D04964 | 0 | hsa_131 | D01392 |
| 1 | hsa_26279 | D06283 | 0 | hsa_5159 | D01972 | 0 | hsa_131 | D10691 |
| 1 | hsa_26279 | D08107 | 0 | hsa_51564 | D09678 | 0 | hsa_131 | D01280 |
| 1 | hsa_26279 | D08221 | 0 | hsa_51564 | D08953 | 0 | hsa_131 | D06634 |
| 1 | hsa_2618 | D03828 | 0 | hsa_51564 | D08555 | 0 | hsa_131 | D10358 |
| 1 | hsa_2618 | D05400 | 0 | hsa_51564 | D10609 | 0 | hsa_130 | D08393 |
| 1 | hsa_2618 | D06503 | 0 | hsa_5156 | D09683 | 0 | hsa_130 | D03163 |
| 1 | hsa_2618 | D07472 | 0 | hsa_5156 | D01972 | 0 | hsa_130 | D10640 |
| 1 | hsa_2618 | D10596 | 0 | hsa_5156 | D08374 | 0 | hsa_130 | D09327 |
| 1 | hsa_2595 | D09779 | 0 | hsa_5156 | D03163 | 0 | hsa_130 | D10091 |
| 1 | hsa_2548 | D09779 | 0 | hsa_5156 | D10658 | 0 | hsa_130 | D01304 |
| 1 | hsa_25 | D03252 | 0 | hsa_5156 | D01899 | 0 | hsa_130 | D09925 |
| 1 | hsa_25 | D03658 | 0 | hsa_5156 | D10432 | 0 | hsa_130 | D10731 |
| 1 | hsa_25 | D06413 | 0 | hsa_5156 | D10381 | 0 | hsa_130 | D08004 |
| 1 | hsa_25 | D06414 | 0 | hsa_5156 | D03745 | 0 | hsa_130 | D01788 |
| 1 | hsa_25 | D08066 | 0 | hsa_5156 | D01155 | 0 | hsa_130 | D10450 |
| 1 | hsa_25 | D08953 | 0 | hsa_5153 | D05094 | 0 | hsa_130 | D08453 |
| 1 | hsa_25 | D09664 | 0 | hsa_5153 | D05728 | 0 | hsa_130 | D08103 |
| 1 | hsa_25 | D09665 | 0 | hsa_5153 | D08393 | 0 | hsa_130 | D07983 |
| 1 | hsa_25 | D09728 | 0 | hsa_5153 | D09667 | 0 | hsa_130 | D10026 |
| 1 | hsa_25 | D10202 | 0 | hsa_5153 | D01572 | 0 | hsa_130 | D10405 |
| 1 | hsa_2475 | D00753 | 0 | hsa_5153 | D09733 | 0 | hsa_128 | D07064 |
| 1 | hsa_2475 | D02714 | 0 | hsa_5153 | D03786 | 0 | hsa_128 | D08492 |
| 1 | hsa_2475 | D06068 | 0 | hsa_5151 | D07969 | 0 | hsa_128 | D05353 |
| 1 | hsa_2475 | D08900 | 0 | hsa_5151 | D10696 | 0 | hsa_128 | D08393 |
| 1 | hsa_2475 | D10076 | 0 | hsa_5151 | D10738 | 0 | hsa_128 | D07579 |
| 1 | hsa_240 | D03010 | 0 | hsa_5151 | D08871 | 0 | hsa_128 | D04276 |
| 1 | hsa_240 | D03080 | 0 | hsa_5151 | D03217 | 0 | hsa_128 | D05589 |
| 1 | hsa_240 | D03652 | 0 | hsa_5151 | D10471 | 0 | hsa_128 | D08947 |
| 1 | hsa_240 | D03990 | 0 | hsa_5151 | D08625 | 0 | hsa_128 | D00341 |
| 1 | hsa_240 | D04151 | 0 | hsa_5151 | D05728 | 0 | hsa_128 | D10568 |
| 1 | hsa_240 | D04770 | 0 | hsa_5151 | D10737 | 0 | hsa_128 | D08238 |
| 1 | hsa_240 | D09667 | 0 | hsa_5151 | D08279 | 0 | hsa_128 | D01134 |
| 1 | hsa_238 | D09731 | 0 | hsa_5151 | D00941 | 0 | hsa_127 | D06503 |
| 1 | hsa_238 | D10450 | 0 | hsa_5151 | D01244 | 0 | hsa_127 | D09710 |
| 1 | hsa_238 | D10551 | 0 | hsa_5150 | D10224 | 0 | hsa_127 | D08631 |
| 1 | hsa_23632 | D01822 | 0 | hsa_5150 | D04498 | 0 | hsa_127 | D06285 |
| 1 | hsa_23632 | D02441 | 0 | hsa_5150 | D05292 | 0 | hsa_126129 | D09640 |
| 1 | hsa_23632 | D03845 | 0 | hsa_5150 | D08524 | 0 | hsa_126129 | D09760 |
| 1 | hsa_23632 | D07871 | 0 | hsa_5150 | D00152 | 0 | hsa_126129 | D07907 |
| 1 | hsa_23621 | D10739 | 0 | hsa_5150 | D04023 | 0 | hsa_126129 | D06379 |
| 1 | hsa_23439 | D00112 | 0 | hsa_5150 | D07147 | 0 | hsa_126129 | D07143 |
| 1 | hsa_23439 | D00297 | 0 | hsa_5150 | D05480 | 0 | hsa_126129 | D07144 |
| 1 | hsa_23439 | D00298 | 0 | hsa_5150 | D00152 | 0 | hsa_126129 | D00595 |
| 1 | hsa_23439 | D01240 | 0 | hsa_5150 | D08657 | 0 | hsa_126129 | D08073 |
| 1 | hsa_23439 | D01379 | 0 | hsa_5150 | D02206 | 0 | hsa_126129 | D03845 |
| 1 | hsa_23439 | D01972 | 0 | hsa_5144 | D10017 | 0 | hsa_126129 | D07131 |
| 1 | hsa_23439 | D02587 | 0 | hsa_5144 | D09038 | 0 | hsa_126 | D07776 |
| 1 | hsa_23439 | D06881 | 0 | hsa_5144 | D07615 | 0 | hsa_126 | D08261 |
| 1 | hsa_23439 | D07147 | 0 | hsa_5144 | D10696 | 0 | hsa_126 | D08410 |
| 1 | hsa_23439 | D07555 | 0 | hsa_5144 | D00156 | 0 | hsa_126 | D06645 |
| 1 | hsa_23439 | D07556 | 0 | hsa_5144 | D10223 | 0 | hsa_126 | D08102 |
| 1 | hsa_23439 | D09847 | 0 | hsa_5144 | D08871 | 0 | hsa_126 | D00087 |
| 1 | hsa_2342 | D04768 | 0 | hsa_5143 | D10064 | 0 | hsa_126 | D01090 |
| 1 | hsa_2339 | D04768 | 0 | hsa_5143 | D07269 | 0 | hsa_126 | D06387 |
| 1 | hsa_2322 | D04696 | 0 | hsa_5143 | D09923 | 0 | hsa_126 | D08566 |
| 1 | hsa_2322 | D05029 | 0 | hsa_5143 | D05019 | 0 | hsa_126 | D05598 |
| 1 | hsa_2322 | D05819 | 0 | hsa_5143 | D10137 | 0 | hsa_126 | D10202 |
| 1 | hsa_2322 | D06005 | 0 | hsa_5143 | D10154 | 0 | hsa_126 | D10372 |
| 1 | hsa_2322 | D09955 | 0 | hsa_5142 | D09618 | 0 | hsa_126 | D02587 |
| 1 | hsa_2322 | D09956 | 0 | hsa_5142 | D08883 | 0 | hsa_126 | D10584 |
| 1 | hsa_2322 | D10709 | 0 | hsa_5142 | D06634 | 0 | hsa_125 | D05380 |
| 1 | hsa_2321 | D08881 | 0 | hsa_5142 | D06606 | 0 | hsa_125 | D06678 |
| 1 | hsa_2321 | D08883 | 0 | hsa_5142 | D10386 | 0 | hsa_125 | D08324 |
| 1 | hsa_2321 | D08907 | 0 | hsa_5142 | D06600 | 0 | hsa_125 | D10688 |
| 1 | hsa_2321 | D09635 | 0 | hsa_5142 | D08529 | 0 | hsa_125 | D05177 |
| 1 | hsa_2321 | D09683 | 0 | hsa_5140 | D09707 | 0 | hsa_125 | D07142 |
| 1 | hsa_2321 | D09919 | 0 | hsa_5140 | D09639 | 0 | hsa_125 | D08870 |
| 1 | hsa_2321 | D09920 | 0 | hsa_5140 | D03248 | 0 | hsa_125 | D10176 |
| 1 | hsa_2321 | D10137 | 0 | hsa_5140 | D08854 | 0 | hsa_125 | D09919 |
| 1 | hsa_2321 | D10138 | 0 | hsa_5140 | D06285 | 0 | hsa_125 | D10358 |
| 1 | hsa_2321 | D10190 | 0 | hsa_5140 | D06285 | 0 | hsa_125 | D08618 |
| 1 | hsa_2321 | D10481 | 0 | hsa_5140 | D05511 | 0 | hsa_124975 | D08900 |
| 1 | hsa_231 | D05893 | 0 | hsa_5140 | D04004 | 0 | hsa_124975 | D07517 |
| 1 | hsa_224 | D00123 | 0 | hsa_5140 | D09728 | 0 | hsa_124975 | D05399 |
| 1 | hsa_2224 | D00939 | 0 | hsa_5139 | D10138 | 0 | hsa_124975 | D10182 |
| 1 | hsa_2224 | D00941 | 0 | hsa_5139 | D05744 | 0 | hsa_124975 | D00941 |
| 1 | hsa_2224 | D00942 | 0 | hsa_5139 | D09919 | 0 | hsa_124975 | D09923 |
| 1 | hsa_2224 | D03234 | 0 | hsa_5139 | D06612 | 0 | hsa_124975 | D06132 |
| 1 | hsa_2224 | D04486 | 0 | hsa_5138 | D04023 | 0 | hsa_124 | D04751 |
| 1 | hsa_2224 | D06378 | 0 | hsa_5138 | D10076 | 0 | hsa_124 | D06600 |
| 1 | hsa_2224 | D06379 | 0 | hsa_5138 | D10157 | 0 | hsa_124 | D07974 |
| 1 | hsa_2224 | D07119 | 0 | hsa_5138 | D09635 | 0 | hsa_124 | D10179 |
| 1 | hsa_2224 | D07123 | 0 | hsa_5138 | D10223 | 0 | hsa_124 | D03260 |
| 1 | hsa_2224 | D07281 | 0 | hsa_5138 | D09883 | 0 | hsa_124 | D08004 |
| 1 | hsa_2224 | D08056 | 0 | hsa_5138 | D03797 | 0 | hsa_115 | D07269 |
| 1 | hsa_2224 | D08073 | 0 | hsa_5138 | D05744 | 0 | hsa_115 | D08873 |
| 1 | hsa_2224 | D08484 | 0 | hsa_5138 | D03082 | 0 | hsa_115 | D10630 |
| 1 | hsa_2224 | D08689 | 0 | hsa_5138 | D03021 | 0 | hsa_115 | D07892 |
| 1 | hsa_2224 | D09198 | 0 | hsa_5138 | D10085 | 0 | hsa_115 | D09701 |
| 1 | hsa_2224 | D10515 | 0 | hsa_5138 | D08363 | 0 | hsa_115 | D07131 |
| 1 | hsa_219 | D00123 | 0 | hsa_5138 | D00595 | 0 | hsa_115 | D01899 |
| 1 | hsa_2185 | D10618 | 0 | hsa_5138 | D05095 | 0 | hsa_114 | D09692 |
| 1 | hsa_2185 | D10619 | 0 | hsa_5138 | D08514 | 0 | hsa_114 | D03793 |
| 1 | hsa_217 | D00123 | 0 | hsa_5138 | D09357 | 0 | hsa_114 | D01672 |
| 1 | hsa_2159 | D03213 | 0 | hsa_5137 | D02084 | 0 | hsa_114 | D03546 |
| 1 | hsa_2159 | D07086 | 0 | hsa_5137 | D09327 | 0 | hsa_114 | D09960 |
| 1 | hsa_2159 | D08873 | 0 | hsa_5137 | D01630 | 0 | hsa_114 | D10027 |
| 1 | hsa_2159 | D08913 | 0 | hsa_5137 | D10604 | 0 | hsa_114 | D09881 |
| 1 | hsa_2159 | D09546 | 0 | hsa_5137 | D08324 | 0 | hsa_114 | D03021 |
| 1 | hsa_2159 | D09710 | 0 | hsa_5136 | D10465 | 0 | hsa_114 | D10543 |
| 1 | hsa_2159 | D09817 | 0 | hsa_5136 | D06320 | 0 | hsa_114 | D04498 |
| 1 | hsa_2159 | D09923 | 0 | hsa_5136 | D00428 | 0 | hsa_113 | D03998 |
| 1 | hsa_2159 | D10471 | 0 | hsa_5136 | D03942 | 0 | hsa_113 | D10137 |
| 1 | hsa_2147 | D00181 | 0 | hsa_5136 | D09622 | 0 | hsa_113 | D07439 |
| 1 | hsa_2147 | D07082 | 0 | hsa_5136 | D10178 | 0 | hsa_113 | D07088 |
| 1 | hsa_2147 | D07143 | 0 | hsa_5136 | D03163 | 0 | hsa_113 | D10318 |
| 1 | hsa_2147 | D07144 | 0 | hsa_5136 | D08107 | 0 | hsa_113 | D10137 |
| 1 | hsa_2147 | D09707 | 0 | hsa_5136 | D10130 | 0 | hsa_113 | D03107 |
| 1 | hsa_208 | D10381 | 0 | hsa_5136 | D07474 | 0 | hsa_113 | D07541 |
| 1 | hsa_208 | D10382 | 0 | hsa_5136 | D08085 | 0 | hsa_113 | D03217 |
| 1 | hsa_208 | D10641 | 0 | hsa_5136 | D07699 | 0 | hsa_113 | D05589 |
| 1 | hsa_208 | D10674 | 0 | hsa_5136 | D07135 | 0 | hsa_113 | D09679 |
| 1 | hsa_207 | D10381 | 0 | hsa_5136 | D00420 | 0 | hsa_11261 | D08324 |
| 1 | hsa_207 | D10382 | 0 | hsa_5136 | D01823 | 0 | hsa_11261 | D10130 |
| 1 | hsa_207 | D10641 | 0 | hsa_50940 | D07580 | 0 | hsa_11261 | D08107 |
| 1 | hsa_207 | D10674 | 0 | hsa_50940 | D07615 | 0 | hsa_11261 | D08689 |
| 1 | hsa_2064 | D09689 | 0 | hsa_50940 | D07888 | 0 | hsa_11261 | D01379 |
| 1 | hsa_2064 | D09690 | 0 | hsa_50940 | D09690 | 0 | hsa_11261 | D04092 |
| 1 | hsa_200895 | D00142 | 0 | hsa_50940 | D01581 | 0 | hsa_11261 | D10721 |
| 1 | hsa_200895 | D02115 | 0 | hsa_50940 | D10551 | 0 | hsa_11261 | D07983 |
| 1 | hsa_200895 | D03942 | 0 | hsa_50940 | D08102 | 0 | hsa_11261 | D03786 |
| 1 | hsa_200895 | D05589 | 0 | hsa_50940 | D10732 | 0 | hsa_11261 | D03650 |
| 1 | hsa_200895 | D06238 | 0 | hsa_50940 | D09753 | 0 | hsa_11261 | D08544 |
| 1 | hsa_200895 | D06239 | 0 | hsa_50940 | D09707 | 0 | hsa_11261 | D03021 |
| 1 | hsa_196883 | D01697 | 0 | hsa_50487 | D10652 | 0 | hsa_11261 | D10641 |
| 1 | hsa_1956 | D04023 | 0 | hsa_50487 | D08375 | 0 | hsa_11261 | D09783 |
| 1 | hsa_1956 | D05399 | 0 | hsa_50487 | D09640 | 0 | hsa_11261 | D09753 |
| 1 | hsa_1956 | D06407 | 0 | hsa_50487 | D09731 | 0 | hsa_11261 | D07818 |
| 1 | hsa_1956 | D07907 | 0 | hsa_50487 | D03378 | 0 | hsa_11261 | D08327 |
| 1 | hsa_1956 | D08108 | 0 | hsa_50487 | D06005 | 0 | hsa_112399 | D10486 |
| 1 | hsa_1956 | D08950 | 0 | hsa_50487 | D04863 | 0 | hsa_112399 | D03099 |
| 1 | hsa_1956 | D09724 | 0 | hsa_50487 | D08514 | 0 | hsa_112399 | D07472 |
| 1 | hsa_1956 | D09733 | 0 | hsa_50487 | D00152 | 0 | hsa_112399 | D01840 |
| 1 | hsa_1956 | D09883 | 0 | hsa_50484 | D08492 | 0 | hsa_112399 | D10696 |
| 1 | hsa_1956 | D10514 | 0 | hsa_50484 | D10060 | 0 | hsa_112399 | D01840 |
| 1 | hsa_1813 | D02729 | 0 | hsa_50484 | D10730 | 0 | hsa_112399 | D05819 |
| 1 | hsa_1813 | D08094 | 0 | hsa_50484 | D10641 | 0 | hsa_112399 | D00469 |
| 1 | hsa_1806 | D01846 | 0 | hsa_50484 | D10060 | 0 | hsa_112399 | D10552 |
| 1 | hsa_1806 | D03998 | 0 | hsa_50484 | D01155 | 0 | hsa_112399 | D08056 |
| 1 | hsa_1803 | D06553 | 0 | hsa_50484 | D10182 | 0 | hsa_112399 | D03749 |
| 1 | hsa_1803 | D06578 | 0 | hsa_50484 | D05019 | 0 | hsa_112398 | D05292 |
| 1 | hsa_1803 | D06645 | 0 | hsa_50484 | D07088 | 0 | hsa_112398 | D05096 |
| 1 | hsa_1803 | D07080 | 0 | hsa_50484 | D08469 | 0 | hsa_112398 | D02084 |
| 1 | hsa_1803 | D08516 | 0 | hsa_5033 | D07137 | 0 | hsa_112398 | D08657 |
| 1 | hsa_1803 | D08616 | 0 | hsa_5033 | D06379 | 0 | hsa_112398 | D08913 |
| 1 | hsa_1803 | D08631 | 0 | hsa_5033 | D05029 | 0 | hsa_112398 | D09665 |
| 1 | hsa_1803 | D08996 | 0 | hsa_5033 | D06320 | 0 | hsa_112398 | D09919 |
| 1 | hsa_1803 | D09326 | 0 | hsa_5033 | D10019 | 0 | hsa_112398 | D10091 |
| 1 | hsa_1803 | D09333 | 0 | hsa_495 | D08913 | 0 | hsa_11238 | D03828 |
| 1 | hsa_1803 | D09334 | 0 | hsa_495 | D07141 | 0 | hsa_11238 | D09020 |
| 1 | hsa_1803 | D09566 | 0 | hsa_495 | D08492 | 0 | hsa_11238 | D03010 |
| 1 | hsa_1803 | D09625 | 0 | hsa_495 | D03786 | 0 | hsa_11238 | D08131 |
| 1 | hsa_1803 | D09753 | 0 | hsa_495 | D03661 | 0 | hsa_11238 | D09589 |
| 1 | hsa_1803 | D09756 | 0 | hsa_495 | D07131 | 0 | hsa_11238 | D08085 |
| 1 | hsa_1803 | D09780 | 0 | hsa_489 | D08854 | 0 | hsa_11238 | D08004 |
| 1 | hsa_1803 | D10178 | 0 | hsa_489 | D02714 | 0 | hsa_11238 | D03657 |
| 1 | hsa_1803 | D10179 | 0 | hsa_489 | D05728 | 0 | hsa_11238 | D09959 |
| 1 | hsa_1803 | D10262 | 0 | hsa_489 | D05901 | 0 | hsa_11238 | D06678 |
| 1 | hsa_1803 | D10317 | 0 | hsa_489 | D02085 | 0 | hsa_112 | D07135 |
| 1 | hsa_1800 | D07698 | 0 | hsa_489 | D02587 | 0 | hsa_112 | D09546 |
| 1 | hsa_1789 | D03021 | 0 | hsa_489 | D10728 | 0 | hsa_112 | D06596 |
| 1 | hsa_1789 | D03665 | 0 | hsa_489 | D08881 | 0 | hsa_112 | D08066 |
| 1 | hsa_1788 | D03021 | 0 | hsa_489 | D08349 | 0 | hsa_112 | D09639 |
| 1 | hsa_1788 | D03665 | 0 | hsa_489 | D07269 | 0 | hsa_112 | D01684 |
| 1 | hsa_1786 | D03021 | 0 | hsa_489 | D04529 | 0 | hsa_112 | D09640 |
| 1 | hsa_1786 | D03665 | 0 | hsa_488 | D10381 | 0 | hsa_112 | D10319 |
| 1 | hsa_1723 | D00749 | 0 | hsa_488 | D08524 | 0 | hsa_112 | D09589 |
| 1 | hsa_1723 | D03154 | 0 | hsa_488 | D07699 | 0 | hsa_112 | D07131 |
| 1 | hsa_1723 | D10172 | 0 | hsa_488 | D04050 | 0 | hsa_112 | D06288 |
| 1 | hsa_1719 | D03942 | 0 | hsa_488 | D03239 | 0 | hsa_111 | D10688 |
| 1 | hsa_1719 | D05589 | 0 | hsa_488 | D09707 | 0 | hsa_111 | D10103 |
| 1 | hsa_1719 | D06239 | 0 | hsa_488 | D07089 | 0 | hsa_111 | D09689 |
| 1 | hsa_169355 | D10640 | 0 | hsa_488 | D08363 | 0 | hsa_111 | D02985 |
| 1 | hsa_1644 | D00558 | 0 | hsa_488 | D06612 | 0 | hsa_111 | D08073 |
| 1 | hsa_1644 | D01653 | 0 | hsa_488 | D03021 | 0 | hsa_111 | D03802 |
| 1 | hsa_1644 | D03082 | 0 | hsa_488 | D02085 | 0 | hsa_111 | D05598 |
| 1 | hsa_1636 | D06076 | 0 | hsa_487 | D03828 | 0 | hsa_109 | D09780 |
| 1 | hsa_1636 | D07499 | 0 | hsa_487 | D10568 | 0 | hsa_109 | D03378 |
| 1 | hsa_1636 | D07699 | 0 | hsa_487 | D01823 | 0 | hsa_109 | D06674 |
| 1 | hsa_1636 | D07781 | 0 | hsa_487 | D10738 | 0 | hsa_109 | D03241 |
| 1 | hsa_1636 | D07892 | 0 | hsa_487 | D04245 | 0 | hsa_109 | D03718 |
| 1 | hsa_1636 | D07992 | 0 | hsa_487 | D09733 | 0 | hsa_109 | D08327 |
| 1 | hsa_1636 | D08068 | 0 | hsa_487 | D04023 | 0 | hsa_109 | D07969 |
| 1 | hsa_1636 | D08131 | 0 | hsa_487 | D03158 | 0 | hsa_109 | D01404 |
| 1 | hsa_1636 | D08225 | 0 | hsa_487 | D10437 | 0 | hsa_109 | D08688 |
| 1 | hsa_1636 | D08529 | 0 | hsa_4860 | D06285 | 0 | hsa_109 | D04107 |
| 1 | hsa_1636 | D08566 | 0 | hsa_4860 | D03239 | 0 | hsa_109 | D03938 |
| 1 | hsa_1636 | D08688 | 0 | hsa_4860 | D07474 | 0 | hsa_10846 | D08103 |
| 1 | hsa_1588 | D03749 | 0 | hsa_4860 | D01404 | 0 | hsa_10846 | D09996 |
| 1 | hsa_1588 | D03786 | 0 | hsa_4860 | D08878 | 0 | hsa_10846 | D09641 |
| 1 | hsa_1588 | D07260 | 0 | hsa_4860 | D02755 | 0 | hsa_10846 | D10543 |
| 1 | hsa_1588 | D07940 | 0 | hsa_4860 | D01840 | 0 | hsa_10846 | D09996 |
| 1 | hsa_1586 | D00420 | 0 | hsa_4860 | D09355 | 0 | hsa_10846 | D07699 |
| 1 | hsa_1586 | D09701 | 0 | hsa_4860 | D05480 | 0 | hsa_10846 | D09955 |
| 1 | hsa_1586 | D10125 | 0 | hsa_4846 | D01784 | 0 | hsa_10846 | D10358 |
| 1 | hsa_1586 | D10146 | 0 | hsa_4846 | D09951 | 0 | hsa_10846 | D03990 |
| 1 | hsa_1584 | D05019 | 0 | hsa_4846 | D08349 | 0 | hsa_10846 | D05029 |
| 1 | hsa_1577 | D09881 | 0 | hsa_4846 | D01070 | 0 | hsa_10846 | D06413 |
| 1 | hsa_1576 | D09881 | 0 | hsa_4846 | D08349 | 0 | hsa_108 | D02085 |
| 1 | hsa_1551 | D09881 | 0 | hsa_4846 | D01684 | 0 | hsa_108 | D07123 |
| 1 | hsa_1513 | D06634 | 0 | hsa_4846 | D01784 | 0 | hsa_108 | D08503 |
| 1 | hsa_1513 | D08955 | 0 | hsa_4846 | D10731 | 0 | hsa_108 | D08878 |
| 1 | hsa_1445 | D09950 | 0 | hsa_4846 | D08058 | 0 | hsa_108 | D00297 |
| 1 | hsa_1445 | D09951 | 0 | hsa_4846 | D08344 | 0 | hsa_108 | D10399 |
| 1 | hsa_1435 | D06402 | 0 | hsa_4846 | D10178 | 0 | hsa_108 | D08524 |
| 1 | hsa_1435 | D08552 | 0 | hsa_4846 | D07082 | 0 | hsa_108 | D08616 |
| 1 | hsa_1432 | D08963 | 0 | hsa_4843 | D01155 | 0 | hsa_108 | D08883 |
| 1 | hsa_1432 | D09386 | 0 | hsa_4843 | D06378 | 0 | hsa_107 | D09996 |
| 1 | hsa_1432 | D09602 | 0 | hsa_4843 | D03349 | 0 | hsa_107 | D10202 |
| 1 | hsa_1432 | D09603 | 0 | hsa_4843 | D03234 | 0 | hsa_107 | D10172 |
| 1 | hsa_1432 | D09639 | 0 | hsa_4843 | D08369 | 0 | hsa_107 | D03234 |
| 1 | hsa_1432 | D10658 | 0 | hsa_4843 | D00939 | 0 | hsa_107 | D00181 |
| 1 | hsa_1432 | D10659 | 0 | hsa_4843 | D09955 | 0 | hsa_107 | D01822 |
| 1 | hsa_143 | D10079 | 0 | hsa_4843 | D09386 | 0 | hsa_107 | D10688 |
| 1 | hsa_143 | D10157 | 0 | hsa_4843 | D03797 | 0 | hsa_107 | D08907 |
| 1 | hsa_142 | D09692 | 0 | hsa_4843 | D05177 | 0 | hsa_107 | D08024 |
| 1 | hsa_142 | D09730 | 0 | hsa_4843 | D01220 | 0 | hsa_1021 | D07519 |
| 1 | hsa_142 | D09913 | 0 | hsa_4843 | D10189 | 0 | hsa_1021 | D10450 |
| 1 | hsa_142 | D10079 | 0 | hsa_4842 | D08870 | 0 | hsa_1021 | D10737 |
| 1 | hsa_142 | D10140 | 0 | hsa_4842 | D10202 | 0 | hsa_1021 | D03158 |
| 1 | hsa_142 | D10157 | 0 | hsa_4842 | D07581 | 0 | hsa_1021 | D03546 |
| 1 | hsa_142 | D10732 | 0 | hsa_4842 | D10653 | 0 | hsa_1021 | D08524 |
| 1 | hsa_142 | D10733 | 0 | hsa_4842 | D08386 | 0 | hsa_1021 | D09707 |
| 1 | hsa_1375 | D05292 | 0 | hsa_4842 | D10405 | 0 | hsa_1021 | D05480 |
| 1 | hsa_1374 | D05292 | 0 | hsa_4842 | D10721 | 0 | hsa_1021 | D10229 |
| 1 | hsa_1312 | D00152 | 0 | hsa_483 | D08094 | 0 | hsa_1021 | D09020 |
| 1 | hsa_1312 | D01259 | 0 | hsa_483 | D01309 | 0 | hsa_1021 | D03158 |
| 1 | hsa_1312 | D03241 | 0 | hsa_483 | D09780 | 0 | hsa_1021 | D09920 |
| 1 | hsa_1312 | D08369 | 0 | hsa_483 | D01747 | 0 | hsa_1021 | D04107 |
| 1 | hsa_131 | D00707 | 0 | hsa_483 | D05353 | 0 | hsa_102 | D08351 |
| 1 | hsa_130 | D00707 | 0 | hsa_483 | D06645 | 0 | hsa_102 | D06272 |
| 1 | hsa_128 | D00707 | 0 | hsa_483 | D06272 | 0 | hsa_102 | D01747 |
| 1 | hsa_127 | D00707 | 0 | hsa_483 | D04014 | 0 | hsa_102 | D10604 |
| 1 | hsa_126129 | D05292 | 0 | hsa_483 | D10255 | 0 | hsa_102 | D10026 |
| 1 | hsa_126 | D00707 | 0 | hsa_483 | D08881 | 0 | hsa_1019 | D08085 |
| 1 | hsa_125 | D00707 | 0 | hsa_482 | D10191 | 0 | hsa_1019 | D05019 |
| 1 | hsa_124975 | D02755 | 0 | hsa_482 | D07338 | 0 | hsa_1019 | D03208 |
| 1 | hsa_124 | D00707 | 0 | hsa_482 | D08688 | 0 | hsa_1019 | D04628 |
| 1 | hsa_115 | D01697 | 0 | hsa_482 | D10319 | 0 | hsa_1019 | D08102 |
| 1 | hsa_114 | D01697 | 0 | hsa_482 | D10381 | 0 | hsa_1019 | D10179 |
| 1 | hsa_113 | D01697 | 0 | hsa_482 | D07581 | 0 | hsa_1019 | D08107 |
| 1 | hsa_11261 | D00107 | 0 | hsa_482 | D04770 | 0 | hsa_1019 | D03213 |
| 1 | hsa_11261 | D00184 | 0 | hsa_482 | D05032 | 0 | hsa_10135 | D07615 |
| 1 | hsa_11261 | D05480 | 0 | hsa_482 | D07541 | 0 | hsa_10135 | D10609 |
| 1 | hsa_11261 | D08556 | 0 | hsa_482 | D00123 | 0 | hsa_10135 | D10382 |
| 1 | hsa_11261 | D09033 | 0 | hsa_482 | D03225 | 0 | hsa_10135 | D09347 |
| 1 | hsa_112399 | D10593 | 0 | hsa_482 | D08668 | 0 | hsa_10135 | D10229 |
| 1 | hsa_112398 | D10593 | 0 | hsa_482 | D00112 | 0 | hsa_10135 | D10552 |
| 1 | hsa_11238 | D01822 | 0 | hsa_481 | D10026 | 0 | hsa_10135 | D10674 |
| 1 | hsa_11238 | D02441 | 0 | hsa_481 | D10688 | 0 | hsa_10135 | D08618 |
| 1 | hsa_11238 | D03845 | 0 | hsa_481 | D06637 | 0 | hsa_10135 | D08344 |
| 1 | hsa_11238 | D07871 | 0 | hsa_481 | D10173 | 0 | hsa_10135 | D04486 |
| 1 | hsa_112 | D01697 | 0 | hsa_481 | D07136 | 0 | hsa_10135 | D04768 |
| 1 | hsa_111 | D01697 | 0 | hsa_481 | D08085 | 0 | hsa_10135 | D03099 |
| 1 | hsa_109 | D01697 | 0 | hsa_481 | D03793 | 0 | hsa_10039 | D08392 |
| 1 | hsa_10846 | D00718 | 0 | hsa_481 | D10060 | 0 | hsa_10039 | D10224 |
| 1 | hsa_10846 | D01220 | 0 | hsa_481 | D10140 | 0 | hsa_10039 | D10155 |
| 1 | hsa_10846 | D01630 | 0 | hsa_481 | D08062 | 0 | hsa_10039 | D09731 |
| 1 | hsa_10846 | D02218 | 0 | hsa_481 | D06288 | 0 | hsa_10039 | D04681 |
| 1 | hsa_10846 | D06132 | 0 | hsa_480 | D07135 | 0 | hsa_10039 | D04151 |
| 1 | hsa_10846 | D07089 | 0 | hsa_480 | D03115 | 0 | hsa_10039 | D07817 |
| 1 | hsa_10846 | D07425 | 0 | hsa_480 | D03260 | 0 | hsa_10039 | D10155 |
| 1 | hsa_10846 | D07439 | 0 | hsa_480 | D07086 | 0 | hsa_10039 | D07940 |
| 1 | hsa_10846 | D07961 | 0 | hsa_480 | D05096 | 0 | hsa_10038 | D02755 |
| 1 | hsa_10846 | D08238 | 0 | hsa_480 | D10688 | 0 | hsa_10038 | D09348 |
| 1 | hsa_108 | D01697 | 0 | hsa_480 | D03021 | 0 | hsa_10038 | D08056 |
| 1 | hsa_107 | D01697 | 0 | hsa_480 | D03546 | 0 | hsa_10038 | D00151 |
| 1 | hsa_1021 | D10372 | 0 | hsa_480 | D04696 | 0 | hsa_10038 | D10432 |
| 1 | hsa_1021 | D10652 | 0 | hsa_478 | D09970 | 0 | hsa_10038 | D05502 |
| 1 | hsa_1021 | D10688 | 0 | hsa_478 | D04964 | 0 | hsa_10014 | D10319 |
| 1 | hsa_102 | D09320 | 0 | hsa_478 | D10103 | 0 | hsa_10014 | D08870 |
| 1 | hsa_1019 | D10372 | 0 | hsa_478 | D09589 | 0 | hsa_10014 | D04092 |
| 1 | hsa_1019 | D10652 | 0 | hsa_478 | D08349 | 0 | hsa_10014 | D10659 |
| 1 | hsa_1019 | D10688 | 0 | hsa_478 | D07119 | 0 | hsa_10014 | D10183 |
| 1 | hsa_10135 | D09678 | 0 | hsa_478 | D01220 | 0 | hsa_10014 | D00151 |
| 1 | hsa_10039 | D10079 | 0 | hsa_478 | D01683 | 0 | hsa_10014 | D10465 |
| 1 | hsa_10039 | D10157 | 0 | hsa_478 | D09951 | 0 | hsa_10014 | D04685 |
| 1 | hsa_10038 | D09692 | 0 | hsa_478 | D03099 | 0 | hsa_10013 | D10255 |
| 1 | hsa_10038 | D10079 | 0 | hsa_478 | D10514 | 0 | hsa_10013 | D10515 |
| 1 | hsa_10038 | D10140 | 0 | hsa_478 | D09020 | 0 | hsa_10013 | D05480 |
| 1 | hsa_10038 | D10157 | 0 | hsa_478 | D00749 | 0 | hsa_10013 | D09678 |
| 1 | hsa_10038 | D10732 | 0 | hsa_478 | D09566 | 0 | hsa_10013 | D05893 |
| 1 | hsa_10038 | D10733 | 0 | hsa_477 | D07974 | 0 | hsa_10013 | D10365 |
| 1 | hsa_10014 | D06637 | 0 | hsa_477 | D08344 | 0 | hsa_10013 | D07816 |
| 1 | hsa_10014 | D08870 | 0 | hsa_477 | D09756 | 0 | hsa_10013 | D00749 |
| 1 | hsa_10014 | D10019 | 0 | hsa_477 | D07516 | 0 | hsa_10013 | D06132 |
| 1 | hsa_10014 | D10060 | 0 | hsa_477 | D08393 | 0 | hsa_10013 | D08086 |
| 1 | hsa_10014 | D10084 | 0 | hsa_477 | D03602 | 0 | hsa_10013 | D01155 |
| 1 | hsa_10014 | D10319 | 0 | hsa_476 | D03241 | 0 | hsa_10013 | D07969 |
| 1 | hsa_10013 | D06637 | 0 | hsa_476 | D08503 | 0 | hsa_10013 | D06005 |
| 1 | hsa_10013 | D08870 | 0 | hsa_476 | D09920 | 0 | hsa_10000 | D06239 |
| 1 | hsa_10013 | D10019 | 0 | hsa_476 | D00939 | 0 | hsa_10000 | D05096 |
| 1 | hsa_10013 | D10060 | 0 | hsa_476 | D09347 | 0 | hsa_10000 | D09783 |
| 1 | hsa_10013 | D10084 | 0 | hsa_476 | D00944 | 0 | hsa_10000 | D10609 |
| 1 | hsa_10013 | D10319 | 0 | hsa_476 | D08881 | 0 | hsa_10000 | D07147 |
| 1 | hsa_10013 | D10661 | 0 | hsa_47 | D09718 | 0 | hsa_10000 | D10728 |
| 1 | hsa_10000 | D10381 | 0 | hsa_47 | D10585 | 0 | hsa_10000 | D06402 |
| 1 | hsa_10000 | D10382 | 0 | hsa_47 | D08689 | 0 | hsa_10000 | D02729 |
| 1 | hsa_10000 | D10641 | 0 | hsa_47 | D05033 | 0 | hsa_10000 | D10674 |
| 1 | hsa_10000 | D10674 | 0 | hsa_47 | D10640 | 0 | hsa_10000 | D05096 |

**Table II** The overall of drug-target interactions for ion channels class on *Dataset2*

| **class** | **Targets** | **drugs** | **class** | **Targets** | **drugs** | **class** | **Targets** | **drugs** |
| --- | --- | --- | --- | --- | --- | --- | --- | --- |
| 1 | hsa_1080 | D00808 | 0 | hsa_1080 | D04657 | 0 | hsa_3736 | D10626 |
| 1 | hsa_1134 | D00492 | 0 | hsa_1080 | D09368 | 0 | hsa_3736 | D00380 |
| 1 | hsa_1134 | D00612 | 0 | hsa_1080 | D01328 | 0 | hsa_3736 | D00764 |
| 1 | hsa_1134 | D00758 | 0 | hsa_1080 | D08459 | 0 | hsa_3736 | D00331 |
| 1 | hsa_1134 | D00759 | 0 | hsa_1080 | D03562 | 0 | hsa_3736 | D08892 |
| 1 | hsa_1134 | D00760 | 0 | hsa_1080 | D00764 | 0 | hsa_3736 | D00533 |
| 1 | hsa_1134 | D00763 | 0 | hsa_1080 | D08098 | 0 | hsa_3736 | D02969 |
| 1 | hsa_1134 | D00764 | 0 | hsa_1080 | D02780 | 0 | hsa_3736 | D00430 |
| 1 | hsa_1134 | D00765 | 0 | hsa_1080 | D07509 | 0 | hsa_3736 | D00336 |
| 1 | hsa_1134 | D00766 | 0 | hsa_1080 | D00549 | 0 | hsa_3736 | D08421 |
| 1 | hsa_1134 | D00767 | 0 | hsa_1080 | D00555 | 0 | hsa_3736 | D04257 |
| 1 | hsa_1134 | D02202 | 0 | hsa_1080 | D00700 | 0 | hsa_3736 | D08009 |
| 1 | hsa_1134 | D02275 | 0 | hsa_1080 | D07442 | 0 | hsa_3736 | D05077 |
| 1 | hsa_1134 | D02292 | 0 | hsa_1080 | D01279 | 0 | hsa_3736 | D04303 |
| 1 | hsa_1134 | D02364 | 0 | hsa_1080 | D08174 | 0 | hsa_3736 | D07595 |
| 1 | hsa_1134 | D04303 | 0 | hsa_1080 | D06652 | 0 | hsa_3736 | D00399 |
| 1 | hsa_1134 | D05703 | 0 | hsa_1080 | D02292 | 0 | hsa_3736 | D06653 |
| 1 | hsa_1134 | D07272 | 0 | hsa_1080 | D02347 | 0 | hsa_3736 | D02096 |
| 1 | hsa_1134 | D08138 | 0 | hsa_1080 | D07971 | 0 | hsa_3736 | D00848 |
| 1 | hsa_1134 | D08655 | 0 | hsa_1080 | D00763 | 0 | hsa_3736 | D03742 |
| 1 | hsa_1135 | D00612 | 0 | hsa_1080 | D01744 | 0 | hsa_3736 | D07442 |
| 1 | hsa_1135 | D02202 | 0 | hsa_1080 | D04657 | 0 | hsa_3736 | D03037 |
| 1 | hsa_1135 | D02364 | 0 | hsa_1080 | D01758 | 0 | hsa_3736 | D08448 |
| 1 | hsa_1135 | D08138 | 0 | hsa_1080 | D00643 | 0 | hsa_3736 | D07971 |
| 1 | hsa_1136 | D00612 | 0 | hsa_1080 | D02347 | 0 | hsa_3736 | D00760 |
| 1 | hsa_1136 | D02202 | 0 | hsa_1080 | D07442 | 0 | hsa_3736 | D02594 |
| 1 | hsa_1136 | D02364 | 0 | hsa_1080 | D07881 | 0 | hsa_3736 | D01593 |
| 1 | hsa_1136 | D08138 | 0 | hsa_1080 | D00557 | 0 | hsa_3736 | D01071 |
| 1 | hsa_1137 | D00612 | 0 | hsa_1080 | D01293 | 0 | hsa_3736 | D00387 |
| 1 | hsa_1137 | D02202 | 0 | hsa_1080 | D07845 | 0 | hsa_3736 | D00430 |
| 1 | hsa_1137 | D02364 | 0 | hsa_1080 | D08964 | 0 | hsa_3736 | D00331 |
| 1 | hsa_1137 | D06282 | 0 | hsa_1080 | D06204 | 0 | hsa_3741 | D08596 |
| 1 | hsa_1137 | D08138 | 0 | hsa_1080 | D10626 | 0 | hsa_3741 | D04226 |
| 1 | hsa_1137 | D08669 | 0 | hsa_1080 | D07993 | 0 | hsa_3741 | D07845 |
| 1 | hsa_1137 | D08935 | 0 | hsa_1080 | D01408 | 0 | hsa_3741 | D02410 |
| 1 | hsa_1137 | D08987 | 0 | hsa_1080 | D00199 | 0 | hsa_3741 | D07552 |
| 1 | hsa_1137 | D09367 | 0 | hsa_1080 | D00329 | 0 | hsa_3741 | D00700 |
| 1 | hsa_1137 | D09368 | 0 | hsa_1080 | D00280 | 0 | hsa_3741 | D00701 |
| 1 | hsa_1137 | D09382 | 0 | hsa_1080 | D00294 | 0 | hsa_3741 | D10613 |
| 1 | hsa_1137 | D09383 | 0 | hsa_1134 | D00555 | 0 | hsa_3741 | D09539 |
| 1 | hsa_1137 | D10111 | 0 | hsa_1134 | D08421 | 0 | hsa_3741 | D05714 |
| 1 | hsa_1137 | D10112 | 0 | hsa_1134 | D08964 | 0 | hsa_3741 | D00457 |
| 1 | hsa_1138 | D00612 | 0 | hsa_1134 | D04300 | 0 | hsa_3741 | D05703 |
| 1 | hsa_1138 | D02202 | 0 | hsa_1134 | D00550 | 0 | hsa_3741 | D02220 |
| 1 | hsa_1138 | D02364 | 0 | hsa_1134 | D07326 | 0 | hsa_3741 | D00506 |
| 1 | hsa_1138 | D08138 | 0 | hsa_1134 | D08669 | 0 | hsa_3741 | D00693 |
| 1 | hsa_1139 | D00612 | 0 | hsa_1134 | D04882 | 0 | hsa_3741 | D02910 |
| 1 | hsa_1139 | D02202 | 0 | hsa_1134 | D01514 | 0 | hsa_3741 | D08116 |
| 1 | hsa_1139 | D02364 | 0 | hsa_1134 | D03679 | 0 | hsa_3741 | D00537 |
| 1 | hsa_1139 | D08138 | 0 | hsa_1134 | D05028 | 0 | hsa_3741 | D01744 |
| 1 | hsa_1139 | D10613 | 0 | hsa_1134 | D08690 | 0 | hsa_3741 | D07999 |
| 1 | hsa_1139 | D10626 | 0 | hsa_1134 | D03492 | 0 | hsa_3741 | D09367 |
| 1 | hsa_1139 | D10693 | 0 | hsa_1134 | D07283 | 0 | hsa_3741 | D08421 |
| 1 | hsa_1139 | D10702 | 0 | hsa_1134 | D00637 | 0 | hsa_3741 | D06653 |
| 1 | hsa_1140 | D00492 | 0 | hsa_1134 | D00766 | 0 | hsa_3741 | D04849 |
| 1 | hsa_1140 | D00611 | 0 | hsa_1134 | D07447 | 0 | hsa_3741 | D04882 |
| 1 | hsa_1140 | D00612 | 0 | hsa_1134 | D02696 | 0 | hsa_3741 | D04048 |
| 1 | hsa_1140 | D00758 | 0 | hsa_1134 | D00631 | 0 | hsa_3741 | D08215 |
| 1 | hsa_1140 | D00759 | 0 | hsa_1134 | D03655 | 0 | hsa_3741 | D07552 |
| 1 | hsa_1140 | D00760 | 0 | hsa_1134 | D08116 | 0 | hsa_3741 | D00499 |
| 1 | hsa_1140 | D00763 | 0 | hsa_1134 | D00375 | 0 | hsa_3741 | D05714 |
| 1 | hsa_1140 | D00764 | 0 | hsa_1134 | D04282 | 0 | hsa_3741 | D00552 |
| 1 | hsa_1140 | D00765 | 0 | hsa_1134 | D10370 | 0 | hsa_3741 | D08282 |
| 1 | hsa_1140 | D00766 | 0 | hsa_1134 | D00636 | 0 | hsa_3741 | D08356 |
| 1 | hsa_1140 | D00767 | 0 | hsa_1134 | D08009 | 0 | hsa_3741 | D01278 |
| 1 | hsa_1140 | D02202 | 0 | hsa_1134 | D05453 | 0 | hsa_3741 | D01104 |
| 1 | hsa_1140 | D02207 | 0 | hsa_1134 | D00766 | 0 | hsa_3741 | D00848 |
| 1 | hsa_1140 | D02275 | 0 | hsa_1134 | D00438 | 0 | hsa_3741 | D05028 |
| 1 | hsa_1140 | D02292 | 0 | hsa_1134 | D01553 | 0 | hsa_3741 | D08001 |
| 1 | hsa_1140 | D02364 | 0 | hsa_1134 | D03100 | 0 | hsa_3741 | D00552 |
| 1 | hsa_1140 | D04303 | 0 | hsa_1134 | D00549 | 0 | hsa_3741 | D07784 |
| 1 | hsa_1140 | D05703 | 0 | hsa_1134 | D00280 | 0 | hsa_3741 | D10693 |
| 1 | hsa_1140 | D07272 | 0 | hsa_1134 | D00710 | 0 | hsa_3741 | D08217 |
| 1 | hsa_1140 | D08138 | 0 | hsa_1134 | D00375 | 0 | hsa_3741 | D04741 |
| 1 | hsa_1140 | D08655 | 0 | hsa_1134 | D01455 | 0 | hsa_3741 | D07058 |
| 1 | hsa_1141 | D00611 | 0 | hsa_1134 | D07845 | 0 | hsa_3741 | D01278 |
| 1 | hsa_1141 | D00612 | 0 | hsa_1134 | D02364 | 0 | hsa_3752 | D10111 |
| 1 | hsa_1141 | D02202 | 0 | hsa_1134 | D01279 | 0 | hsa_3752 | D09383 |
| 1 | hsa_1141 | D02204 | 0 | hsa_1134 | D08217 | 0 | hsa_3752 | D00551 |
| 1 | hsa_1141 | D02364 | 0 | hsa_1134 | D08098 | 0 | hsa_3752 | D01744 |
| 1 | hsa_1141 | D06282 | 0 | hsa_1134 | D01758 | 0 | hsa_3752 | D02283 |
| 1 | hsa_1141 | D08138 | 0 | hsa_1134 | D03914 | 0 | hsa_3752 | D03914 |
| 1 | hsa_1141 | D08669 | 0 | hsa_1134 | D02537 | 0 | hsa_3752 | D04728 |
| 1 | hsa_1141 | D08935 | 0 | hsa_1134 | D02594 | 0 | hsa_3752 | D07993 |
| 1 | hsa_1141 | D08987 | 0 | hsa_1134 | D02385 | 0 | hsa_3752 | D05453 |
| 1 | hsa_1141 | D09367 | 0 | hsa_1134 | D00706 | 0 | hsa_3752 | D09368 |
| 1 | hsa_1141 | D09368 | 0 | hsa_1134 | D08138 | 0 | hsa_3752 | D08100 |
| 1 | hsa_1141 | D09382 | 0 | hsa_1134 | D02410 | 0 | hsa_3752 | D04918 |
| 1 | hsa_1141 | D09383 | 0 | hsa_1134 | D04048 | 0 | hsa_3752 | D01245 |
| 1 | hsa_1141 | D10111 | 0 | hsa_1134 | D05714 | 0 | hsa_3752 | D07993 |
| 1 | hsa_1141 | D10112 | 0 | hsa_1134 | D00695 | 0 | hsa_3752 | D00380 |
| 1 | hsa_1142 | D00611 | 0 | hsa_1134 | D01293 | 0 | hsa_3752 | D02780 |
| 1 | hsa_1142 | D00612 | 0 | hsa_1134 | D08596 | 0 | hsa_3752 | D00470 |
| 1 | hsa_1142 | D02202 | 0 | hsa_1135 | D08283 | 0 | hsa_3752 | D08935 |
| 1 | hsa_1142 | D02204 | 0 | hsa_1135 | D04955 | 0 | hsa_3752 | D01245 |
| 1 | hsa_1142 | D02364 | 0 | hsa_1135 | D04257 | 0 | hsa_3752 | D08507 |
| 1 | hsa_1142 | D08138 | 0 | hsa_1135 | D08282 | 0 | hsa_3752 | D01278 |
| 1 | hsa_1143 | D00611 | 0 | hsa_1135 | D00365 | 0 | hsa_3752 | D06517 |
| 1 | hsa_1143 | D00612 | 0 | hsa_1135 | D01514 | 0 | hsa_3752 | D00700 |
| 1 | hsa_1143 | D02202 | 0 | hsa_1135 | D00532 | 0 | hsa_3752 | D00430 |
| 1 | hsa_1143 | D02204 | 0 | hsa_1135 | D00636 | 0 | hsa_3752 | D05714 |
| 1 | hsa_1143 | D02364 | 0 | hsa_1135 | D02202 | 0 | hsa_3752 | D00329 |
| 1 | hsa_1143 | D08138 | 0 | hsa_1135 | D08127 | 0 | hsa_3752 | D00304 |
| 1 | hsa_1144 | D00492 | 0 | hsa_1135 | D00693 | 0 | hsa_3752 | D03155 |
| 1 | hsa_1144 | D00611 | 0 | hsa_1135 | D00767 | 0 | hsa_3752 | D00376 |
| 1 | hsa_1144 | D00612 | 0 | hsa_1135 | D01254 | 0 | hsa_3752 | D07894 |
| 1 | hsa_1144 | D00758 | 0 | hsa_1135 | D09789 | 0 | hsa_3752 | D00531 |
| 1 | hsa_1144 | D00759 | 0 | hsa_1135 | D01303 | 0 | hsa_3752 | D03492 |
| 1 | hsa_1144 | D00760 | 0 | hsa_1135 | D09789 | 0 | hsa_3752 | D08655 |
| 1 | hsa_1144 | D00763 | 0 | hsa_1135 | D04226 | 0 | hsa_3752 | D00387 |
| 1 | hsa_1144 | D00764 | 0 | hsa_1135 | D08667 | 0 | hsa_3752 | D00701 |
| 1 | hsa_1144 | D00765 | 0 | hsa_1135 | D00331 | 0 | hsa_3752 | D07185 |
| 1 | hsa_1144 | D00766 | 0 | hsa_1135 | D09612 | 0 | hsa_3752 | D00250 |
| 1 | hsa_1144 | D00767 | 0 | hsa_1135 | D00549 | 0 | hsa_3752 | D07520 |
| 1 | hsa_1144 | D02202 | 0 | hsa_1135 | D08138 | 0 | hsa_3752 | D00636 |
| 1 | hsa_1144 | D02207 | 0 | hsa_1135 | D07185 | 0 | hsa_3752 | D02220 |
| 1 | hsa_1144 | D02275 | 0 | hsa_1135 | D05077 | 0 | hsa_3752 | D08690 |
| 1 | hsa_1144 | D02292 | 0 | hsa_1135 | D07993 | 0 | hsa_3752 | D00376 |
| 1 | hsa_1144 | D02364 | 0 | hsa_1135 | D02220 | 0 | hsa_3752 | D04882 |
| 1 | hsa_1144 | D04303 | 0 | hsa_1135 | D10627 | 0 | hsa_3752 | D00765 |
| 1 | hsa_1144 | D05703 | 0 | hsa_1135 | D07845 | 0 | hsa_3752 | D02385 |
| 1 | hsa_1144 | D07272 | 0 | hsa_1135 | D08892 | 0 | hsa_3752 | D08459 |
| 1 | hsa_1144 | D08138 | 0 | hsa_1135 | D07845 | 0 | hsa_3752 | D02969 |
| 1 | hsa_1144 | D08655 | 0 | hsa_1135 | D02347 | 0 | hsa_3757 | D01245 |
| 1 | hsa_1145 | D00492 | 0 | hsa_1135 | D01173 | 0 | hsa_3757 | D07978 |
| 1 | hsa_1145 | D00611 | 0 | hsa_1135 | D04882 | 0 | hsa_3757 | D01254 |
| 1 | hsa_1145 | D00612 | 0 | hsa_1135 | D00553 | 0 | hsa_3757 | D01810 |
| 1 | hsa_1145 | D00758 | 0 | hsa_1135 | D01243 | 0 | hsa_3757 | D01408 |
| 1 | hsa_1145 | D00759 | 0 | hsa_1135 | D06656 | 0 | hsa_3757 | D00643 |
| 1 | hsa_1145 | D00760 | 0 | hsa_1135 | D01657 | 0 | hsa_3757 | D06172 |
| 1 | hsa_1145 | D00763 | 0 | hsa_1136 | D01554 | 0 | hsa_3757 | D03742 |
| 1 | hsa_1145 | D00764 | 0 | hsa_1136 | D03492 | 0 | hsa_3757 | D00553 |
| 1 | hsa_1145 | D00765 | 0 | hsa_1136 | D00611 | 0 | hsa_3757 | D00631 |
| 1 | hsa_1145 | D00766 | 0 | hsa_1136 | D08667 | 0 | hsa_3757 | D01657 |
| 1 | hsa_1145 | D00767 | 0 | hsa_1136 | D00267 | 0 | hsa_3757 | D00470 |
| 1 | hsa_1145 | D02202 | 0 | hsa_1136 | D06517 | 0 | hsa_3757 | D00331 |
| 1 | hsa_1145 | D02207 | 0 | hsa_1136 | D07881 | 0 | hsa_3757 | D00293 |
| 1 | hsa_1145 | D02275 | 0 | hsa_1136 | D00765 | 0 | hsa_3757 | D00555 |
| 1 | hsa_1145 | D02292 | 0 | hsa_1136 | D03742 | 0 | hsa_3757 | D08101 |
| 1 | hsa_1145 | D02364 | 0 | hsa_1136 | D02220 | 0 | hsa_3757 | D02780 |
| 1 | hsa_1145 | D04303 | 0 | hsa_1136 | D05453 | 0 | hsa_3757 | D00531 |
| 1 | hsa_1145 | D05703 | 0 | hsa_1136 | D09789 | 0 | hsa_3757 | D07185 |
| 1 | hsa_1145 | D07272 | 0 | hsa_1136 | D02537 | 0 | hsa_3757 | D01303 |
| 1 | hsa_1145 | D08138 | 0 | hsa_1136 | D03746 | 0 | hsa_3757 | D02252 |
| 1 | hsa_1145 | D08655 | 0 | hsa_1136 | D02696 | 0 | hsa_3757 | D00611 |
| 1 | hsa_1146 | D00492 | 0 | hsa_1136 | D06282 | 0 | hsa_3757 | D01268 |
| 1 | hsa_1146 | D00611 | 0 | hsa_1136 | D10702 | 0 | hsa_3757 | D01372 |
| 1 | hsa_1146 | D00612 | 0 | hsa_1136 | D04257 | 0 | hsa_3757 | D10626 |
| 1 | hsa_1146 | D00758 | 0 | hsa_1136 | D07283 | 0 | hsa_3757 | D04728 |
| 1 | hsa_1146 | D00759 | 0 | hsa_1136 | D01316 | 0 | hsa_3757 | D08892 |
| 1 | hsa_1146 | D00760 | 0 | hsa_1136 | D08458 | 0 | hsa_3757 | D02202 |
| 1 | hsa_1146 | D00763 | 0 | hsa_1136 | D04048 | 0 | hsa_3757 | D07845 |
| 1 | hsa_1146 | D00764 | 0 | hsa_1136 | D00551 | 0 | hsa_3757 | D07962 |
| 1 | hsa_1146 | D00765 | 0 | hsa_1136 | D02969 | 0 | hsa_3757 | D04226 |
| 1 | hsa_1146 | D00766 | 0 | hsa_1136 | D03732 | 0 | hsa_3757 | D00418 |
| 1 | hsa_1146 | D00767 | 0 | hsa_1136 | D10702 | 0 | hsa_3757 | D07450 |
| 1 | hsa_1146 | D02202 | 0 | hsa_1136 | D00365 | 0 | hsa_3757 | D09215 |
| 1 | hsa_1146 | D02207 | 0 | hsa_1136 | D01785 | 0 | hsa_3757 | D04728 |
| 1 | hsa_1146 | D02275 | 0 | hsa_1136 | D10194 | 0 | hsa_3757 | D08145 |
| 1 | hsa_1146 | D02292 | 0 | hsa_1136 | D07447 | 0 | hsa_3757 | D00714 |
| 1 | hsa_1146 | D02364 | 0 | hsa_1136 | D02292 | 0 | hsa_3757 | D02274 |
| 1 | hsa_1146 | D04303 | 0 | hsa_1136 | D09367 | 0 | hsa_3757 | D01408 |
| 1 | hsa_1146 | D05703 | 0 | hsa_1136 | D00759 | 0 | hsa_3757 | D00499 |
| 1 | hsa_1146 | D07272 | 0 | hsa_1136 | D06204 | 0 | hsa_3757 | D10693 |
| 1 | hsa_1146 | D08138 | 0 | hsa_1136 | D02410 | 0 | hsa_3757 | D00399 |
| 1 | hsa_1146 | D08655 | 0 | hsa_1136 | D08840 | 0 | hsa_3757 | D04303 |
| 1 | hsa_18 | D00304 | 0 | hsa_1136 | D04918 | 0 | hsa_3757 | D08507 |
| 1 | hsa_18 | D00399 | 0 | hsa_1136 | D07552 | 0 | hsa_3757 | D02624 |
| 1 | hsa_18 | D00710 | 0 | hsa_1136 | D05453 | 0 | hsa_3757 | D04303 |
| 1 | hsa_18 | D08667 | 0 | hsa_1136 | D01293 | 0 | hsa_3757 | D08667 |
| 1 | hsa_2554 | D00267 | 0 | hsa_1136 | D00418 | 0 | hsa_3757 | D10194 |
| 1 | hsa_2554 | D00280 | 0 | hsa_1136 | D00329 | 0 | hsa_3757 | D00011 |
| 1 | hsa_2554 | D00311 | 0 | hsa_1136 | D04721 | 0 | hsa_3757 | D01908 |
| 1 | hsa_2554 | D00365 | 0 | hsa_1136 | D00550 | 0 | hsa_3757 | D02283 |
| 1 | hsa_2554 | D00370 | 0 | hsa_1136 | D09215 | 0 | hsa_3757 | D06656 |
| 1 | hsa_2554 | D00376 | 0 | hsa_1136 | D02283 | 0 | hsa_3757 | D08356 |
| 1 | hsa_2554 | D00387 | 0 | hsa_1137 | D03732 | 0 | hsa_3757 | D06517 |
| 1 | hsa_2554 | D00430 | 0 | hsa_1137 | D10627 | 0 | hsa_3757 | D08340 |
| 1 | hsa_2554 | D00457 | 0 | hsa_1137 | D04721 | 0 | hsa_3757 | D03562 |
| 1 | hsa_2554 | D00470 | 0 | hsa_1137 | D02973 | 0 | hsa_3757 | D07881 |
| 1 | hsa_2554 | D00500 | 0 | hsa_1137 | D07272 | 0 | hsa_3767 | D05703 |
| 1 | hsa_2554 | D00506 | 0 | hsa_1137 | D00763 | 0 | hsa_3767 | D04226 |
| 1 | hsa_2554 | D00531 | 0 | hsa_1137 | D07962 | 0 | hsa_3767 | D00399 |
| 1 | hsa_2554 | D00532 | 0 | hsa_1137 | D00537 | 0 | hsa_3767 | D08490 |
| 1 | hsa_2554 | D00555 | 0 | hsa_1137 | D02969 | 0 | hsa_3767 | D08100 |
| 1 | hsa_2554 | D00557 | 0 | hsa_1137 | D00387 | 0 | hsa_3767 | D01303 |
| 1 | hsa_2554 | D00693 | 0 | hsa_1137 | D02045 | 0 | hsa_3767 | D01785 |
| 1 | hsa_2554 | D00695 | 0 | hsa_1137 | D01810 | 0 | hsa_3767 | D00336 |
| 1 | hsa_2554 | D00696 | 0 | hsa_1137 | D08840 | 0 | hsa_3767 | D05442 |
| 1 | hsa_2554 | D00700 | 0 | hsa_1137 | D08935 | 0 | hsa_3767 | D00304 |
| 1 | hsa_2554 | D00701 | 0 | hsa_1137 | D04741 | 0 | hsa_3767 | D09539 |
| 1 | hsa_2554 | D00706 | 0 | hsa_1137 | D04918 | 0 | hsa_3767 | D08421 |
| 1 | hsa_2554 | D00713 | 0 | hsa_1137 | D01173 | 0 | hsa_3767 | D08217 |
| 1 | hsa_2554 | D00714 | 0 | hsa_1137 | D02780 | 0 | hsa_3767 | D02275 |
| 1 | hsa_2554 | D01230 | 0 | hsa_1137 | D08667 | 0 | hsa_3767 | D05442 |
| 1 | hsa_2554 | D01245 | 0 | hsa_1137 | D00537 | 0 | hsa_3767 | D08155 |
| 1 | hsa_2554 | D01254 | 0 | hsa_1137 | D10627 | 0 | hsa_3767 | D00643 |
| 1 | hsa_2554 | D01268 | 0 | hsa_1137 | D08669 | 0 | hsa_3767 | D09789 |
| 1 | hsa_2554 | D01278 | 0 | hsa_1137 | D04048 | 0 | hsa_3767 | D08111 |
| 1 | hsa_2554 | D01279 | 0 | hsa_1137 | D05028 | 0 | hsa_3767 | D07881 |
| 1 | hsa_2554 | D01286 | 0 | hsa_1137 | D00713 | 0 | hsa_3767 | D06172 |
| 1 | hsa_2554 | D01292 | 0 | hsa_1137 | D00293 | 0 | hsa_3767 | D08421 |
| 1 | hsa_2554 | D01293 | 0 | hsa_1137 | D08690 | 0 | hsa_3767 | D10111 |
| 1 | hsa_2554 | D01310 | 0 | hsa_1137 | D02220 | 0 | hsa_3767 | D00331 |
| 1 | hsa_2554 | D01316 | 0 | hsa_1137 | D02594 | 0 | hsa_3767 | D08116 |
| 1 | hsa_2554 | D01328 | 0 | hsa_1137 | D00252 | 0 | hsa_3767 | D00552 |
| 1 | hsa_2554 | D01354 | 0 | hsa_1137 | D07058 | 0 | hsa_3767 | D05077 |
| 1 | hsa_2554 | D01372 | 0 | hsa_1137 | D07326 | 0 | hsa_3767 | D03655 |
| 1 | hsa_2554 | D01408 | 0 | hsa_1137 | D10702 | 0 | hsa_3767 | D08448 |
| 1 | hsa_2554 | D01514 | 0 | hsa_1137 | D02973 | 0 | hsa_3767 | D05703 |
| 1 | hsa_2554 | D01564 | 0 | hsa_1137 | D02624 | 0 | hsa_3767 | D02969 |
| 1 | hsa_2554 | D01593 | 0 | hsa_1137 | D05028 | 0 | hsa_3767 | D08655 |
| 1 | hsa_2554 | D01657 | 0 | hsa_1137 | D08435 | 0 | hsa_3767 | D04048 |
| 1 | hsa_2554 | D01740 | 0 | hsa_1137 | D04741 | 0 | hsa_3767 | D09383 |
| 1 | hsa_2554 | D01744 | 0 | hsa_1137 | D07886 | 0 | hsa_3784 | D04882 |
| 1 | hsa_2554 | D01758 | 0 | hsa_1137 | D00512 | 0 | hsa_3784 | D04955 |
| 1 | hsa_2554 | D02252 | 0 | hsa_1137 | D10613 | 0 | hsa_3784 | D01908 |
| 1 | hsa_2554 | D02283 | 0 | hsa_1137 | D04300 | 0 | hsa_3784 | D00304 |
| 1 | hsa_2554 | D02594 | 0 | hsa_1137 | D08174 | 0 | hsa_3784 | D08282 |
| 1 | hsa_2554 | D02616 | 0 | hsa_1137 | D08422 | 0 | hsa_3784 | D03155 |
| 1 | hsa_2554 | D02617 | 0 | hsa_1137 | D02969 | 0 | hsa_3784 | D01254 |
| 1 | hsa_2554 | D02624 | 0 | hsa_1137 | D09918 | 0 | hsa_3784 | D01071 |
| 1 | hsa_2554 | D03155 | 0 | hsa_1137 | D06652 | 0 | hsa_3784 | D08667 |
| 1 | hsa_2554 | D03562 | 0 | hsa_1137 | D02220 | 0 | hsa_3784 | D08377 |
| 1 | hsa_2554 | D03737 | 0 | hsa_1137 | D00636 | 0 | hsa_3784 | D07509 |
| 1 | hsa_2554 | D04257 | 0 | hsa_1137 | D07999 | 0 | hsa_3784 | D08935 |
| 1 | hsa_2554 | D04282 | 0 | hsa_1137 | D05482 | 0 | hsa_3784 | D07894 |
| 1 | hsa_2554 | D04300 | 0 | hsa_1138 | D00331 | 0 | hsa_3784 | D02364 |
| 1 | hsa_2554 | D04721 | 0 | hsa_1138 | D00848 | 0 | hsa_3784 | D00553 |
| 1 | hsa_2554 | D04882 | 0 | hsa_1138 | D07845 | 0 | hsa_3784 | D08356 |
| 1 | hsa_2554 | D05028 | 0 | hsa_1138 | D03914 | 0 | hsa_3784 | D08987 |
| 1 | hsa_2554 | D07326 | 0 | hsa_1138 | D01908 | 0 | hsa_3784 | D00550 |
| 1 | hsa_2554 | D07409 | 0 | hsa_1138 | D08669 | 0 | hsa_3784 | D01316 |
| 1 | hsa_2554 | D07784 | 0 | hsa_1138 | D02973 | 0 | hsa_3784 | D10613 |
| 1 | hsa_2554 | D08145 | 0 | hsa_1138 | D04849 | 0 | hsa_3784 | D00418 |
| 1 | hsa_2554 | D08283 | 0 | hsa_1138 | D09918 | 0 | hsa_3784 | D00370 |
| 1 | hsa_2554 | D08356 | 0 | hsa_1138 | D04741 | 0 | hsa_3784 | D00759 |
| 1 | hsa_2554 | D08481 | 0 | hsa_1138 | D02594 | 0 | hsa_3784 | D08283 |
| 1 | hsa_2554 | D08507 | 0 | hsa_1138 | D07283 | 0 | hsa_3784 | D07450 |
| 1 | hsa_2554 | D08690 | 0 | hsa_1138 | D00733 | 0 | hsa_3784 | D00280 |
| 1 | hsa_2554 | D08840 | 0 | hsa_1138 | D08270 | 0 | hsa_3784 | D00848 |
| 1 | hsa_2554 | D10194 | 0 | hsa_1138 | D00767 | 0 | hsa_3784 | D01908 |
| 1 | hsa_2555 | D00267 | 0 | hsa_1138 | D01372 | 0 | hsa_3784 | D10693 |
| 1 | hsa_2555 | D00280 | 0 | hsa_1138 | D00848 | 0 | hsa_3784 | D00387 |
| 1 | hsa_2555 | D00311 | 0 | hsa_1138 | D00710 | 0 | hsa_3784 | D01104 |
| 1 | hsa_2555 | D00365 | 0 | hsa_1138 | D05028 | 0 | hsa_3784 | D03742 |
| 1 | hsa_2555 | D00370 | 0 | hsa_1138 | D01479 | 0 | hsa_3784 | D09612 |
| 1 | hsa_2555 | D00376 | 0 | hsa_1138 | D00267 | 0 | hsa_3784 | D02292 |
| 1 | hsa_2555 | D00387 | 0 | hsa_1138 | D00506 | 0 | hsa_3784 | D08138 |
| 1 | hsa_2555 | D00430 | 0 | hsa_1138 | D02973 | 0 | hsa_3784 | D01479 |
| 1 | hsa_2555 | D00457 | 0 | hsa_1138 | D04728 | 0 | hsa_3784 | D00531 |
| 1 | hsa_2555 | D00470 | 0 | hsa_1138 | D09789 | 0 | hsa_3784 | D00808 |
| 1 | hsa_2555 | D00500 | 0 | hsa_1138 | D02616 | 0 | hsa_3784 | D07326 |
| 1 | hsa_2555 | D00506 | 0 | hsa_1138 | D03655 | 0 | hsa_3784 | D03100 |
| 1 | hsa_2555 | D00531 | 0 | hsa_1138 | D08138 | 0 | hsa_3784 | D08282 |
| 1 | hsa_2555 | D00532 | 0 | hsa_1138 | D00553 | 0 | hsa_3784 | D01230 |
| 1 | hsa_2555 | D00555 | 0 | hsa_1138 | D04721 | 0 | hsa_3784 | D00695 |
| 1 | hsa_2555 | D00557 | 0 | hsa_1138 | D00766 | 0 | hsa_3784 | D01310 |
| 1 | hsa_2555 | D00693 | 0 | hsa_1138 | D00376 | 0 | hsa_3784 | D08892 |
| 1 | hsa_2555 | D00694 | 0 | hsa_1138 | D00499 | 0 | hsa_3784 | D02275 |
| 1 | hsa_2555 | D00695 | 0 | hsa_1138 | D00499 | 0 | hsa_3784 | D09757 |
| 1 | hsa_2555 | D00696 | 0 | hsa_1138 | D10370 | 0 | hsa_3784 | D04282 |
| 1 | hsa_2555 | D00697 | 0 | hsa_1138 | D08421 | 0 | hsa_3784 | D00506 |
| 1 | hsa_2555 | D00700 | 0 | hsa_1138 | D01479 | 0 | hsa_3784 | D01071 |
| 1 | hsa_2555 | D00701 | 0 | hsa_1138 | D00553 | 0 | hsa_3785 | D01785 |
| 1 | hsa_2555 | D00706 | 0 | hsa_1138 | D03492 | 0 | hsa_3785 | D08669 |
| 1 | hsa_2555 | D00713 | 0 | hsa_1138 | D08394 | 0 | hsa_3785 | D01514 |
| 1 | hsa_2555 | D00714 | 0 | hsa_1138 | D05482 | 0 | hsa_3785 | D09918 |
| 1 | hsa_2555 | D01071 | 0 | hsa_1138 | D00280 | 0 | hsa_3785 | D10613 |
| 1 | hsa_2555 | D01230 | 0 | hsa_1138 | D00267 | 0 | hsa_3785 | D00706 |
| 1 | hsa_2555 | D01245 | 0 | hsa_1138 | D00611 | 0 | hsa_3785 | D07494 |
| 1 | hsa_2555 | D01253 | 0 | hsa_1138 | D02045 | 0 | hsa_3785 | D00370 |
| 1 | hsa_2555 | D01254 | 0 | hsa_1138 | D03679 | 0 | hsa_3785 | D02616 |
| 1 | hsa_2555 | D01268 | 0 | hsa_1138 | D00512 | 0 | hsa_3785 | D05442 |
| 1 | hsa_2555 | D01278 | 0 | hsa_1138 | D08490 | 0 | hsa_3785 | D00506 |
| 1 | hsa_2555 | D01279 | 0 | hsa_1138 | D06653 | 0 | hsa_3785 | D00532 |
| 1 | hsa_2555 | D01286 | 0 | hsa_1138 | D06204 | 0 | hsa_3785 | D00380 |
| 1 | hsa_2555 | D01292 | 0 | hsa_1138 | D01849 | 0 | hsa_3785 | D07784 |
| 1 | hsa_2555 | D01293 | 0 | hsa_1138 | D00267 | 0 | hsa_3785 | D07058 |
| 1 | hsa_2555 | D01310 | 0 | hsa_1138 | D00760 | 0 | hsa_3785 | D08458 |
| 1 | hsa_2555 | D01316 | 0 | hsa_1138 | D00365 | 0 | hsa_3785 | D00700 |
| 1 | hsa_2555 | D01328 | 0 | hsa_1138 | D01310 | 0 | hsa_3785 | D03037 |
| 1 | hsa_2555 | D01354 | 0 | hsa_1138 | D07881 | 0 | hsa_3785 | D08060 |
| 1 | hsa_2555 | D01372 | 0 | hsa_1138 | D01372 | 0 | hsa_3785 | D00643 |
| 1 | hsa_2555 | D01408 | 0 | hsa_1139 | D02385 | 0 | hsa_3785 | D00531 |
| 1 | hsa_2555 | D01514 | 0 | hsa_1139 | D06282 | 0 | hsa_3785 | D04303 |
| 1 | hsa_2555 | D01564 | 0 | hsa_1139 | D10627 | 0 | hsa_3785 | D01254 |
| 1 | hsa_2555 | D01593 | 0 | hsa_1139 | D07678 | 0 | hsa_3785 | D01173 |
| 1 | hsa_2555 | D01657 | 0 | hsa_1139 | D07886 | 0 | hsa_3785 | D00763 |
| 1 | hsa_2555 | D01740 | 0 | hsa_1139 | D01455 | 0 | hsa_3785 | D00537 |
| 1 | hsa_2555 | D01744 | 0 | hsa_1139 | D00631 | 0 | hsa_3785 | D01372 |
| 1 | hsa_2555 | D01758 | 0 | hsa_1139 | D00637 | 0 | hsa_3785 | D08275 |
| 1 | hsa_2555 | D02252 | 0 | hsa_1139 | D00701 | 0 | hsa_3785 | D00710 |
| 1 | hsa_2555 | D02283 | 0 | hsa_1139 | D08270 | 0 | hsa_3785 | D05714 |
| 1 | hsa_2555 | D02594 | 0 | hsa_1139 | D03547 | 0 | hsa_3785 | D00303 |
| 1 | hsa_2555 | D02616 | 0 | hsa_1139 | D00695 | 0 | hsa_3785 | D08667 |
| 1 | hsa_2555 | D02617 | 0 | hsa_1139 | D00733 | 0 | hsa_3785 | D02045 |
| 1 | hsa_2555 | D02624 | 0 | hsa_1139 | D00611 | 0 | hsa_3785 | D10626 |
| 1 | hsa_2555 | D03155 | 0 | hsa_1139 | D00808 | 0 | hsa_3785 | D00267 |
| 1 | hsa_2555 | D03562 | 0 | hsa_1139 | D00537 | 0 | hsa_3785 | D00399 |
| 1 | hsa_2555 | D03737 | 0 | hsa_1139 | D03037 | 0 | hsa_3785 | D00380 |
| 1 | hsa_2555 | D04257 | 0 | hsa_1139 | D00763 | 0 | hsa_43 | D01279 |
| 1 | hsa_2555 | D04282 | 0 | hsa_1139 | D03155 | 0 | hsa_43 | D07442 |
| 1 | hsa_2555 | D04300 | 0 | hsa_1139 | D02910 | 0 | hsa_43 | D00199 |
| 1 | hsa_2555 | D04721 | 0 | hsa_1139 | D00713 | 0 | hsa_43 | D08669 |
| 1 | hsa_2555 | D04882 | 0 | hsa_1139 | D00552 | 0 | hsa_43 | D00760 |
| 1 | hsa_2555 | D05028 | 0 | hsa_1139 | D02292 | 0 | hsa_43 | D06392 |
| 1 | hsa_2555 | D06106 | 0 | hsa_1139 | D00280 | 0 | hsa_43 | D01908 |
| 1 | hsa_2555 | D07326 | 0 | hsa_1139 | D09215 | 0 | hsa_43 | D08009 |
| 1 | hsa_2555 | D07409 | 0 | hsa_1139 | D05453 | 0 | hsa_43 | D05482 |
| 1 | hsa_2555 | D07784 | 0 | hsa_1139 | D02347 | 0 | hsa_43 | D00765 |
| 1 | hsa_2555 | D08145 | 0 | hsa_1139 | D00760 | 0 | hsa_43 | D00499 |
| 1 | hsa_2555 | D08283 | 0 | hsa_1139 | D01328 | 0 | hsa_43 | D01408 |
| 1 | hsa_2555 | D08356 | 0 | hsa_1139 | D02616 | 0 | hsa_43 | D09382 |
| 1 | hsa_2555 | D08481 | 0 | hsa_1139 | D09612 | 0 | hsa_43 | D07326 |
| 1 | hsa_2555 | D08507 | 0 | hsa_1139 | D03155 | 0 | hsa_43 | D10194 |
| 1 | hsa_2555 | D08690 | 0 | hsa_1139 | D00557 | 0 | hsa_43 | D08596 |
| 1 | hsa_2555 | D08840 | 0 | hsa_1139 | D07409 | 0 | hsa_43 | D00710 |
| 1 | hsa_2555 | D10194 | 0 | hsa_1139 | D07520 | 0 | hsa_43 | D00611 |
| 1 | hsa_2556 | D00267 | 0 | hsa_1139 | D01514 | 0 | hsa_43 | D07283 |
| 1 | hsa_2556 | D00280 | 0 | hsa_1139 | D00710 | 0 | hsa_43 | D00064 |
| 1 | hsa_2556 | D00311 | 0 | hsa_1139 | D07894 | 0 | hsa_43 | D00225 |
| 1 | hsa_2556 | D00365 | 0 | hsa_1139 | D05714 | 0 | hsa_43 | D06282 |
| 1 | hsa_2556 | D00370 | 0 | hsa_1139 | D10627 | 0 | hsa_43 | D08283 |
| 1 | hsa_2556 | D00376 | 0 | hsa_1139 | D01553 | 0 | hsa_43 | D08667 |
| 1 | hsa_2556 | D00387 | 0 | hsa_1139 | D08283 | 0 | hsa_43 | D02220 |
| 1 | hsa_2556 | D00430 | 0 | hsa_1139 | D07450 | 0 | hsa_43 | D08655 |
| 1 | hsa_2556 | D00457 | 0 | hsa_1139 | D00457 | 0 | hsa_43 | D08060 |
| 1 | hsa_2556 | D00470 | 0 | hsa_1139 | D08127 | 0 | hsa_43 | D02410 |
| 1 | hsa_2556 | D00500 | 0 | hsa_1139 | D02220 | 0 | hsa_43 | D06392 |
| 1 | hsa_2556 | D00506 | 0 | hsa_1139 | D00011 | 0 | hsa_43 | D00848 |
| 1 | hsa_2556 | D00531 | 0 | hsa_1139 | D00199 | 0 | hsa_43 | D01292 |
| 1 | hsa_2556 | D00532 | 0 | hsa_1139 | D07283 | 0 | hsa_43 | D00759 |
| 1 | hsa_2556 | D00555 | 0 | hsa_1140 | D00506 | 0 | hsa_43 | D10626 |
| 1 | hsa_2556 | D00557 | 0 | hsa_1140 | D08448 | 0 | hsa_43 | D02910 |
| 1 | hsa_2556 | D00693 | 0 | hsa_1140 | D00531 | 0 | hsa_43 | D08421 |
| 1 | hsa_2556 | D00694 | 0 | hsa_1140 | D02617 | 0 | hsa_43 | D01145 |
| 1 | hsa_2556 | D00695 | 0 | hsa_1140 | D08145 | 0 | hsa_43 | D07894 |
| 1 | hsa_2556 | D00696 | 0 | hsa_1140 | D09789 | 0 | hsa_43 | D09789 |
| 1 | hsa_2556 | D00697 | 0 | hsa_1140 | D00418 | 0 | hsa_43 | D00336 |
| 1 | hsa_2556 | D00700 | 0 | hsa_1140 | D00611 | 0 | hsa_43 | D00370 |
| 1 | hsa_2556 | D00701 | 0 | hsa_1140 | D00695 | 0 | hsa_43 | D08127 |
| 1 | hsa_2556 | D00706 | 0 | hsa_1140 | D00763 | 0 | hsa_43 | D00199 |
| 1 | hsa_2556 | D00713 | 0 | hsa_1140 | D06146 | 0 | hsa_43 | D00764 |
| 1 | hsa_2556 | D00714 | 0 | hsa_1140 | D00765 | 0 | hsa_43 | D00549 |
| 1 | hsa_2556 | D01071 | 0 | hsa_1140 | D04300 | 0 | hsa_43 | D02202 |
| 1 | hsa_2556 | D01230 | 0 | hsa_1140 | D02096 | 0 | hsa_43 | D03547 |
| 1 | hsa_2556 | D01245 | 0 | hsa_1140 | D10626 | 0 | hsa_43 | D00765 |
| 1 | hsa_2556 | D01253 | 0 | hsa_1140 | D03492 | 0 | hsa_43 | D05442 |
| 1 | hsa_2556 | D01254 | 0 | hsa_1140 | D00365 | 0 | hsa_43 | D01408 |
| 1 | hsa_2556 | D01268 | 0 | hsa_1140 | D00399 | 0 | hsa_43 | D10702 |
| 1 | hsa_2556 | D01278 | 0 | hsa_1140 | D00386 | 0 | hsa_43 | D10613 |
| 1 | hsa_2556 | D01279 | 0 | hsa_1140 | D07326 | 0 | hsa_43 | D07283 |
| 1 | hsa_2556 | D01286 | 0 | hsa_1140 | D00098 | 0 | hsa_43 | D01243 |
| 1 | hsa_2556 | D01292 | 0 | hsa_1140 | D00199 | 0 | hsa_43 | D01564 |
| 1 | hsa_2556 | D01293 | 0 | hsa_1140 | D09382 | 0 | hsa_43 | D08001 |
| 1 | hsa_2556 | D01310 | 0 | hsa_1140 | D07058 | 0 | hsa_55584 | D00304 |
| 1 | hsa_2556 | D01316 | 0 | hsa_1140 | D00470 | 0 | hsa_55584 | D02252 |
| 1 | hsa_2556 | D01328 | 0 | hsa_1140 | D08100 | 0 | hsa_55584 | D00380 |
| 1 | hsa_2556 | D01354 | 0 | hsa_1140 | D00064 | 0 | hsa_55584 | D00512 |
| 1 | hsa_2556 | D01372 | 0 | hsa_1140 | D00733 | 0 | hsa_55584 | D00294 |
| 1 | hsa_2556 | D01408 | 0 | hsa_1140 | D07971 | 0 | hsa_55584 | D03746 |
| 1 | hsa_2556 | D01514 | 0 | hsa_1140 | D08690 | 0 | hsa_55584 | D06652 |
| 1 | hsa_2556 | D01564 | 0 | hsa_1140 | D08507 | 0 | hsa_55584 | D06517 |
| 1 | hsa_2556 | D01593 | 0 | hsa_1140 | D03679 | 0 | hsa_55584 | D07886 |
| 1 | hsa_2556 | D01657 | 0 | hsa_1140 | D08490 | 0 | hsa_55584 | D01245 |
| 1 | hsa_2556 | D01740 | 0 | hsa_1140 | D00098 | 0 | hsa_55584 | D08448 |
| 1 | hsa_2556 | D01744 | 0 | hsa_1140 | D00303 | 0 | hsa_55584 | D07326 |
| 1 | hsa_2556 | D01758 | 0 | hsa_1140 | D08101 | 0 | hsa_55584 | D02969 |
| 1 | hsa_2556 | D02252 | 0 | hsa_1140 | D04955 | 0 | hsa_55584 | D05482 |
| 1 | hsa_2556 | D02283 | 0 | hsa_1140 | D02624 | 0 | hsa_55584 | D06388 |
| 1 | hsa_2556 | D02594 | 0 | hsa_1140 | D04905 | 0 | hsa_55584 | D10370 |
| 1 | hsa_2556 | D02616 | 0 | hsa_1140 | D07509 | 0 | hsa_55584 | D05145 |
| 1 | hsa_2556 | D02617 | 0 | hsa_1140 | D00506 | 0 | hsa_55584 | D00611 |
| 1 | hsa_2556 | D02624 | 0 | hsa_1140 | D01758 | 0 | hsa_55584 | D10470 |
| 1 | hsa_2556 | D03155 | 0 | hsa_1140 | D02364 | 0 | hsa_55584 | D01071 |
| 1 | hsa_2556 | D03562 | 0 | hsa_1140 | D00713 | 0 | hsa_55584 | D03492 |
| 1 | hsa_2556 | D03737 | 0 | hsa_1140 | D08481 | 0 | hsa_55584 | D08270 |
| 1 | hsa_2556 | D04257 | 0 | hsa_1140 | D07283 | 0 | hsa_55584 | D00533 |
| 1 | hsa_2556 | D04282 | 0 | hsa_1140 | D02045 | 0 | hsa_55584 | D08596 |
| 1 | hsa_2556 | D04300 | 0 | hsa_1140 | D00631 | 0 | hsa_55584 | D00733 |
| 1 | hsa_2556 | D04721 | 0 | hsa_1140 | D01310 | 0 | hsa_55584 | D07886 |
| 1 | hsa_2556 | D04882 | 0 | hsa_1140 | D00294 | 0 | hsa_55584 | D03155 |
| 1 | hsa_2556 | D05028 | 0 | hsa_1140 | D05145 | 0 | hsa_55584 | D00375 |
| 1 | hsa_2556 | D06106 | 0 | hsa_1140 | D00611 | 0 | hsa_55584 | D02410 |
| 1 | hsa_2556 | D07326 | 0 | hsa_1140 | D10111 | 0 | hsa_55584 | D03914 |
| 1 | hsa_2556 | D07409 | 0 | hsa_1140 | D01254 | 0 | hsa_55584 | D00506 |
| 1 | hsa_2556 | D07784 | 0 | hsa_1140 | D08377 | 0 | hsa_55584 | D09368 |
| 1 | hsa_2556 | D08145 | 0 | hsa_1140 | D00537 | 0 | hsa_55584 | D09918 |
| 1 | hsa_2556 | D08283 | 0 | hsa_1141 | D01785 | 0 | hsa_55584 | D00311 |
| 1 | hsa_2556 | D08356 | 0 | hsa_1141 | D02252 | 0 | hsa_55584 | D01104 |
| 1 | hsa_2556 | D08481 | 0 | hsa_1141 | D00386 | 0 | hsa_55584 | D01479 |
| 1 | hsa_2556 | D08507 | 0 | hsa_1141 | D02973 | 0 | hsa_55584 | D01372 |
| 1 | hsa_2556 | D08690 | 0 | hsa_1141 | D00387 | 0 | hsa_55584 | D07943 |
| 1 | hsa_2556 | D08840 | 0 | hsa_1141 | D00549 | 0 | hsa_55584 | D00631 |
| 1 | hsa_2556 | D10194 | 0 | hsa_1141 | D06653 | 0 | hsa_55584 | D01785 |
| 1 | hsa_2557 | D00267 | 0 | hsa_1141 | D04657 | 0 | hsa_55584 | D00551 |
| 1 | hsa_2557 | D00280 | 0 | hsa_1141 | D00304 | 0 | hsa_55584 | D00550 |
| 1 | hsa_2557 | D00311 | 0 | hsa_1141 | D08111 | 0 | hsa_55584 | D06652 |
| 1 | hsa_2557 | D00365 | 0 | hsa_1141 | D10194 | 0 | hsa_55584 | D03746 |
| 1 | hsa_2557 | D00370 | 0 | hsa_1141 | D04257 | 0 | hsa_55584 | D01230 |
| 1 | hsa_2557 | D00376 | 0 | hsa_1141 | D00764 | 0 | hsa_55584 | D00280 |
| 1 | hsa_2557 | D00387 | 0 | hsa_1141 | D10693 | 0 | hsa_55584 | D00492 |
| 1 | hsa_2557 | D00430 | 0 | hsa_1141 | D00380 | 0 | hsa_55584 | D08174 |
| 1 | hsa_2557 | D00457 | 0 | hsa_1141 | D03155 | 0 | hsa_55584 | D02410 |
| 1 | hsa_2557 | D00470 | 0 | hsa_1141 | D00329 | 0 | hsa_55584 | D00399 |
| 1 | hsa_2557 | D00500 | 0 | hsa_1141 | D00418 | 0 | hsa_55584 | D08060 |
| 1 | hsa_2557 | D00506 | 0 | hsa_1141 | D00387 | 0 | hsa_55584 | D04955 |
| 1 | hsa_2557 | D00531 | 0 | hsa_1141 | D01071 | 0 | hsa_55584 | D05714 |
| 1 | hsa_2557 | D00532 | 0 | hsa_1141 | D01553 | 0 | hsa_55584 | D02364 |
| 1 | hsa_2557 | D00555 | 0 | hsa_1141 | D08282 | 0 | hsa_55799 | D08270 |
| 1 | hsa_2557 | D00557 | 0 | hsa_1141 | D09757 | 0 | hsa_55799 | D06656 |
| 1 | hsa_2557 | D00693 | 0 | hsa_1141 | D00399 | 0 | hsa_55799 | D10627 |
| 1 | hsa_2557 | D00694 | 0 | hsa_1141 | D07894 | 0 | hsa_55799 | D04882 |
| 1 | hsa_2557 | D00695 | 0 | hsa_1141 | D01268 | 0 | hsa_55799 | D08422 |
| 1 | hsa_2557 | D00696 | 0 | hsa_1141 | D07283 | 0 | hsa_55799 | D00537 |
| 1 | hsa_2557 | D00697 | 0 | hsa_1141 | D00370 | 0 | hsa_55799 | D00293 |
| 1 | hsa_2557 | D00700 | 0 | hsa_1141 | D04131 | 0 | hsa_55799 | D08215 |
| 1 | hsa_2557 | D00701 | 0 | hsa_1141 | D08459 | 0 | hsa_55799 | D00331 |
| 1 | hsa_2557 | D00706 | 0 | hsa_1141 | D01408 | 0 | hsa_55799 | D01303 |
| 1 | hsa_2557 | D00713 | 0 | hsa_1141 | D08127 | 0 | hsa_55799 | D08174 |
| 1 | hsa_2557 | D00714 | 0 | hsa_1141 | D07845 | 0 | hsa_55799 | D00764 |
| 1 | hsa_2557 | D01071 | 0 | hsa_1141 | D05442 | 0 | hsa_55799 | D01657 |
| 1 | hsa_2557 | D01230 | 0 | hsa_1141 | D07520 | 0 | hsa_55799 | D01243 |
| 1 | hsa_2557 | D01245 | 0 | hsa_1141 | D04918 | 0 | hsa_55799 | D02594 |
| 1 | hsa_2557 | D01253 | 0 | hsa_1141 | D02696 | 0 | hsa_55799 | D00612 |
| 1 | hsa_2557 | D01254 | 0 | hsa_1141 | D05447 | 0 | hsa_55799 | D01562 |
| 1 | hsa_2557 | D01268 | 0 | hsa_1141 | D00637 | 0 | hsa_55799 | D09917 |
| 1 | hsa_2557 | D01278 | 0 | hsa_1141 | D00532 | 0 | hsa_55799 | D02385 |
| 1 | hsa_2557 | D01279 | 0 | hsa_1141 | D00758 | 0 | hsa_55799 | D02537 |
| 1 | hsa_2557 | D01286 | 0 | hsa_1141 | D00695 | 0 | hsa_55799 | D08422 |
| 1 | hsa_2557 | D01292 | 0 | hsa_1141 | D04303 | 0 | hsa_55799 | D10470 |
| 1 | hsa_2557 | D01293 | 0 | hsa_1141 | D01316 | 0 | hsa_55799 | D08001 |
| 1 | hsa_2557 | D01310 | 0 | hsa_1141 | D08127 | 0 | hsa_55799 | D01354 |
| 1 | hsa_2557 | D01316 | 0 | hsa_1141 | D08138 | 0 | hsa_55799 | D00252 |
| 1 | hsa_2557 | D01328 | 0 | hsa_1141 | D00760 | 0 | hsa_55799 | D08669 |
| 1 | hsa_2557 | D01354 | 0 | hsa_1142 | D05714 | 0 | hsa_55799 | D07999 |
| 1 | hsa_2557 | D01372 | 0 | hsa_1142 | D01479 | 0 | hsa_55799 | D00612 |
| 1 | hsa_2557 | D01408 | 0 | hsa_1142 | D00733 | 0 | hsa_55799 | D10627 |
| 1 | hsa_2557 | D01514 | 0 | hsa_1142 | D00304 | 0 | hsa_55799 | D04131 |
| 1 | hsa_2557 | D01564 | 0 | hsa_1142 | D00531 | 0 | hsa_55799 | D00267 |
| 1 | hsa_2557 | D01593 | 0 | hsa_1142 | D03100 | 0 | hsa_55799 | D00612 |
| 1 | hsa_2557 | D01657 | 0 | hsa_1142 | D02617 | 0 | hsa_55799 | D00376 |
| 1 | hsa_2557 | D01740 | 0 | hsa_1142 | D00500 | 0 | hsa_55799 | D08009 |
| 1 | hsa_2557 | D01744 | 0 | hsa_1142 | D01479 | 0 | hsa_55799 | D05442 |
| 1 | hsa_2557 | D01758 | 0 | hsa_1142 | D09917 | 0 | hsa_55799 | D04741 |
| 1 | hsa_2557 | D02252 | 0 | hsa_1142 | D02292 | 0 | hsa_55799 | D08145 |
| 1 | hsa_2557 | D02283 | 0 | hsa_1142 | D00532 | 0 | hsa_55799 | D04905 |
| 1 | hsa_2557 | D02594 | 0 | hsa_1142 | D01479 | 0 | hsa_55799 | D09569 |
| 1 | hsa_2557 | D02616 | 0 | hsa_1142 | D08596 | 0 | hsa_55799 | D09383 |
| 1 | hsa_2557 | D02617 | 0 | hsa_1142 | D00733 | 0 | hsa_55799 | D09382 |
| 1 | hsa_2557 | D02624 | 0 | hsa_1142 | D00533 | 0 | hsa_55879 | D10693 |
| 1 | hsa_2557 | D03155 | 0 | hsa_1142 | D03037 | 0 | hsa_55879 | D06282 |
| 1 | hsa_2557 | D03562 | 0 | hsa_1142 | D08111 | 0 | hsa_55879 | D01554 |
| 1 | hsa_2557 | D03737 | 0 | hsa_1142 | D00492 | 0 | hsa_55879 | D00553 |
| 1 | hsa_2557 | D04257 | 0 | hsa_1142 | D07881 | 0 | hsa_55879 | D00767 |
| 1 | hsa_2557 | D04282 | 0 | hsa_1142 | D02364 | 0 | hsa_55879 | D04226 |
| 1 | hsa_2557 | D04300 | 0 | hsa_1142 | D01554 | 0 | hsa_55879 | D08145 |
| 1 | hsa_2557 | D04721 | 0 | hsa_1142 | D01071 | 0 | hsa_55879 | D01230 |
| 1 | hsa_2557 | D04882 | 0 | hsa_1142 | D01908 | 0 | hsa_55879 | D00701 |
| 1 | hsa_2557 | D05028 | 0 | hsa_1142 | D03155 | 0 | hsa_55879 | D00532 |
| 1 | hsa_2557 | D06106 | 0 | hsa_1142 | D03655 | 0 | hsa_55879 | D09539 |
| 1 | hsa_2557 | D07326 | 0 | hsa_1142 | D03746 | 0 | hsa_55879 | D01479 |
| 1 | hsa_2557 | D07409 | 0 | hsa_1142 | D00380 | 0 | hsa_55879 | D04849 |
| 1 | hsa_2557 | D07784 | 0 | hsa_1142 | D03732 | 0 | hsa_55879 | D04849 |
| 1 | hsa_2557 | D08145 | 0 | hsa_1142 | D01071 | 0 | hsa_55879 | D08100 |
| 1 | hsa_2557 | D08283 | 0 | hsa_1142 | D01354 | 0 | hsa_55879 | D00555 |
| 1 | hsa_2557 | D08356 | 0 | hsa_1142 | D00380 | 0 | hsa_55879 | D08935 |
| 1 | hsa_2557 | D08481 | 0 | hsa_1142 | D09382 | 0 | hsa_55879 | D02045 |
| 1 | hsa_2557 | D08507 | 0 | hsa_1142 | D05482 | 0 | hsa_55879 | D05145 |
| 1 | hsa_2557 | D08690 | 0 | hsa_1142 | D01292 | 0 | hsa_55879 | D00500 |
| 1 | hsa_2557 | D08840 | 0 | hsa_1142 | D07447 | 0 | hsa_55879 | D00499 |
| 1 | hsa_2557 | D10194 | 0 | hsa_1142 | D08001 | 0 | hsa_55879 | D09918 |
| 1 | hsa_2558 | D00267 | 0 | hsa_1142 | D04849 | 0 | hsa_55879 | D01593 |
| 1 | hsa_2558 | D00280 | 0 | hsa_1142 | D08356 | 0 | hsa_55879 | D06204 |
| 1 | hsa_2558 | D00311 | 0 | hsa_1142 | D08840 | 0 | hsa_55879 | D00098 |
| 1 | hsa_2558 | D00365 | 0 | hsa_1142 | D10111 | 0 | hsa_55879 | D00294 |
| 1 | hsa_2558 | D00370 | 0 | hsa_1142 | D00733 | 0 | hsa_55879 | D08217 |
| 1 | hsa_2558 | D00376 | 0 | hsa_1142 | D01758 | 0 | hsa_55879 | D06656 |
| 1 | hsa_2558 | D00387 | 0 | hsa_1142 | D00331 | 0 | hsa_55879 | D03655 |
| 1 | hsa_2558 | D00430 | 0 | hsa_1143 | D00767 | 0 | hsa_55879 | D08282 |
| 1 | hsa_2558 | D00457 | 0 | hsa_1143 | D06204 | 0 | hsa_55879 | D07881 |
| 1 | hsa_2558 | D00470 | 0 | hsa_1143 | D00700 | 0 | hsa_55879 | D06652 |
| 1 | hsa_2558 | D00500 | 0 | hsa_1143 | D00430 | 0 | hsa_55879 | D00637 |
| 1 | hsa_2558 | D00506 | 0 | hsa_1143 | D02283 | 0 | hsa_55879 | D03155 |
| 1 | hsa_2558 | D00531 | 0 | hsa_1143 | D01455 | 0 | hsa_55879 | D00531 |
| 1 | hsa_2558 | D00532 | 0 | hsa_1143 | D02910 | 0 | hsa_55879 | D02252 |
| 1 | hsa_2558 | D00555 | 0 | hsa_1143 | D00549 | 0 | hsa_55879 | D01785 |
| 1 | hsa_2558 | D00557 | 0 | hsa_1143 | D02202 | 0 | hsa_55879 | D00848 |
| 1 | hsa_2558 | D00693 | 0 | hsa_1143 | D00636 | 0 | hsa_55879 | D04721 |
| 1 | hsa_2558 | D00694 | 0 | hsa_1143 | D00808 | 0 | hsa_55879 | D07962 |
| 1 | hsa_2558 | D00695 | 0 | hsa_1143 | D01243 | 0 | hsa_55879 | D01849 |
| 1 | hsa_2558 | D00696 | 0 | hsa_1143 | D03742 | 0 | hsa_55879 | D00848 |
| 1 | hsa_2558 | D00697 | 0 | hsa_1143 | D02252 | 0 | hsa_55879 | D00537 |
| 1 | hsa_2558 | D00700 | 0 | hsa_1143 | D00695 | 0 | hsa_55879 | D04728 |
| 1 | hsa_2558 | D00701 | 0 | hsa_1143 | D10702 | 0 | hsa_57053 | D00370 |
| 1 | hsa_2558 | D00706 | 0 | hsa_1143 | D07894 | 0 | hsa_57053 | D08435 |
| 1 | hsa_2558 | D00713 | 0 | hsa_1143 | D08459 | 0 | hsa_57053 | D07978 |
| 1 | hsa_2558 | D00714 | 0 | hsa_1143 | D01230 | 0 | hsa_57053 | D08459 |
| 1 | hsa_2558 | D01071 | 0 | hsa_1143 | D08127 | 0 | hsa_57053 | D10470 |
| 1 | hsa_2558 | D01230 | 0 | hsa_1143 | D00764 | 0 | hsa_57053 | D07409 |
| 1 | hsa_2558 | D01245 | 0 | hsa_1143 | D00848 | 0 | hsa_57053 | D05442 |
| 1 | hsa_2558 | D01253 | 0 | hsa_1143 | D03037 | 0 | hsa_57053 | D00430 |
| 1 | hsa_2558 | D01254 | 0 | hsa_1143 | D01071 | 0 | hsa_57053 | D09539 |
| 1 | hsa_2558 | D01268 | 0 | hsa_1143 | D09569 | 0 | hsa_57053 | D08690 |
| 1 | hsa_2558 | D01278 | 0 | hsa_1143 | D07999 | 0 | hsa_57053 | D00418 |
| 1 | hsa_2558 | D01279 | 0 | hsa_1143 | D10627 | 0 | hsa_57053 | D01293 |
| 1 | hsa_2558 | D01286 | 0 | hsa_1143 | D02385 | 0 | hsa_57053 | D00733 |
| 1 | hsa_2558 | D01292 | 0 | hsa_1143 | D02347 | 0 | hsa_57053 | D08116 |
| 1 | hsa_2558 | D01293 | 0 | hsa_1143 | D07185 | 0 | hsa_57053 | D07520 |
| 1 | hsa_2558 | D01310 | 0 | hsa_1143 | D04741 | 0 | hsa_57053 | D04048 |
| 1 | hsa_2558 | D01316 | 0 | hsa_1143 | D09367 | 0 | hsa_57053 | D01326 |
| 1 | hsa_2558 | D01328 | 0 | hsa_1143 | D08987 | 0 | hsa_57053 | D00499 |
| 1 | hsa_2558 | D01354 | 0 | hsa_1143 | D02594 | 0 | hsa_57053 | D04849 |
| 1 | hsa_2558 | D01372 | 0 | hsa_1143 | D00430 | 0 | hsa_57053 | D09612 |
| 1 | hsa_2558 | D01408 | 0 | hsa_1143 | D10112 | 0 | hsa_57053 | D08101 |
| 1 | hsa_2558 | D01514 | 0 | hsa_1143 | D08155 | 0 | hsa_57053 | D01278 |
| 1 | hsa_2558 | D01564 | 0 | hsa_1143 | D03562 | 0 | hsa_57053 | D04741 |
| 1 | hsa_2558 | D01593 | 0 | hsa_1143 | D08667 | 0 | hsa_57053 | D01316 |
| 1 | hsa_2558 | D01657 | 0 | hsa_1143 | D00331 | 0 | hsa_57053 | D01604 |
| 1 | hsa_2558 | D01740 | 0 | hsa_1143 | D01145 | 0 | hsa_57053 | D00375 |
| 1 | hsa_2558 | D01744 | 0 | hsa_1143 | D02696 | 0 | hsa_57053 | D07272 |
| 1 | hsa_2558 | D01758 | 0 | hsa_1143 | D01564 | 0 | hsa_57053 | D08448 |
| 1 | hsa_2558 | D02252 | 0 | hsa_1143 | D07450 | 0 | hsa_57053 | D03655 |
| 1 | hsa_2558 | D02283 | 0 | hsa_1143 | D00710 | 0 | hsa_57053 | D00531 |
| 1 | hsa_2558 | D02594 | 0 | hsa_1143 | D03547 | 0 | hsa_57053 | D10194 |
| 1 | hsa_2558 | D02616 | 0 | hsa_1143 | D02045 | 0 | hsa_57053 | D09035 |
| 1 | hsa_2558 | D02617 | 0 | hsa_1143 | D02274 | 0 | hsa_57053 | D01408 |
| 1 | hsa_2558 | D02624 | 0 | hsa_1143 | D08340 | 0 | hsa_57053 | D08145 |
| 1 | hsa_2558 | D03155 | 0 | hsa_1143 | D08435 | 0 | hsa_57053 | D07326 |
| 1 | hsa_2558 | D03562 | 0 | hsa_1143 | D04308 | 0 | hsa_57053 | D00500 |
| 1 | hsa_2558 | D03737 | 0 | hsa_1144 | D03679 | 0 | hsa_57053 | D07283 |
| 1 | hsa_2558 | D04257 | 0 | hsa_1144 | D05077 | 0 | hsa_57053 | D01303 |
| 1 | hsa_2558 | D04282 | 0 | hsa_1144 | D07943 | 0 | hsa_57053 | D09612 |
| 1 | hsa_2558 | D04300 | 0 | hsa_1144 | D08840 | 0 | hsa_57053 | D08145 |
| 1 | hsa_2558 | D04721 | 0 | hsa_1144 | D00370 | 0 | hsa_57053 | D00311 |
| 1 | hsa_2558 | D04882 | 0 | hsa_1144 | D00713 | 0 | hsa_57053 | D00329 |
| 1 | hsa_2558 | D05028 | 0 | hsa_1144 | D03742 | 0 | hsa_57053 | D02220 |
| 1 | hsa_2558 | D06106 | 0 | hsa_1144 | D07520 | 0 | hsa_57053 | D00713 |
| 1 | hsa_2558 | D07326 | 0 | hsa_1144 | D06653 | 0 | hsa_57053 | D04300 |
| 1 | hsa_2558 | D07409 | 0 | hsa_1144 | D00765 | 0 | hsa_57053 | D04657 |
| 1 | hsa_2558 | D07784 | 0 | hsa_1144 | D09918 | 0 | hsa_57053 | D01593 |
| 1 | hsa_2558 | D08145 | 0 | hsa_1144 | D00532 | 0 | hsa_57053 | D08964 |
| 1 | hsa_2558 | D08283 | 0 | hsa_1144 | D01286 | 0 | hsa_5743 | D04303 |
| 1 | hsa_2558 | D08356 | 0 | hsa_1144 | D00713 | 0 | hsa_5743 | D02780 |
| 1 | hsa_2558 | D08481 | 0 | hsa_1144 | D02910 | 0 | hsa_5743 | D03547 |
| 1 | hsa_2558 | D08507 | 0 | hsa_1144 | D00399 | 0 | hsa_5743 | D10370 |
| 1 | hsa_2558 | D08690 | 0 | hsa_1144 | D08690 | 0 | hsa_5743 | D05028 |
| 1 | hsa_2558 | D08840 | 0 | hsa_1144 | D00714 | 0 | hsa_5743 | D10470 |
| 1 | hsa_2558 | D10194 | 0 | hsa_1144 | D06652 | 0 | hsa_5743 | D07409 |
| 1 | hsa_2559 | D00267 | 0 | hsa_1144 | D03562 | 0 | hsa_5743 | D00280 |
| 1 | hsa_2559 | D00280 | 0 | hsa_1144 | D03155 | 0 | hsa_5743 | D00636 |
| 1 | hsa_2559 | D00311 | 0 | hsa_1144 | D08127 | 0 | hsa_5743 | D00763 |
| 1 | hsa_2559 | D00365 | 0 | hsa_1144 | D01593 | 0 | hsa_5743 | D04303 |
| 1 | hsa_2559 | D00370 | 0 | hsa_1144 | D01856 | 0 | hsa_5743 | D08215 |
| 1 | hsa_2559 | D00376 | 0 | hsa_1144 | D00764 | 0 | hsa_5743 | D06656 |
| 1 | hsa_2559 | D00387 | 0 | hsa_1144 | D06282 | 0 | hsa_5743 | D05442 |
| 1 | hsa_2559 | D00430 | 0 | hsa_1144 | D05028 | 0 | hsa_5743 | D10702 |
| 1 | hsa_2559 | D00457 | 0 | hsa_1144 | D01849 | 0 | hsa_5743 | D09383 |
| 1 | hsa_2559 | D00470 | 0 | hsa_1144 | D09917 | 0 | hsa_5743 | D07442 |
| 1 | hsa_2559 | D00500 | 0 | hsa_1144 | D00399 | 0 | hsa_5743 | D00418 |
| 1 | hsa_2559 | D00506 | 0 | hsa_1144 | D08421 | 0 | hsa_5743 | D01564 |
| 1 | hsa_2559 | D00531 | 0 | hsa_1144 | D09918 | 0 | hsa_5743 | D08283 |
| 1 | hsa_2559 | D00532 | 0 | hsa_1144 | D07283 | 0 | hsa_5743 | D08448 |
| 1 | hsa_2559 | D00555 | 0 | hsa_1144 | D08987 | 0 | hsa_5743 | D08283 |
| 1 | hsa_2559 | D00557 | 0 | hsa_1144 | D06146 | 0 | hsa_5743 | D08667 |
| 1 | hsa_2559 | D00693 | 0 | hsa_1144 | D00430 | 0 | hsa_5743 | D08282 |
| 1 | hsa_2559 | D00694 | 0 | hsa_1144 | D00551 | 0 | hsa_5743 | D08270 |
| 1 | hsa_2559 | D00695 | 0 | hsa_1144 | D06392 | 0 | hsa_5743 | D00765 |
| 1 | hsa_2559 | D00696 | 0 | hsa_1144 | D02252 | 0 | hsa_5743 | D07595 |
| 1 | hsa_2559 | D00697 | 0 | hsa_1144 | D00293 | 0 | hsa_5743 | D10613 |
| 1 | hsa_2559 | D00700 | 0 | hsa_1144 | D08987 | 0 | hsa_5743 | D02973 |
| 1 | hsa_2559 | D00701 | 0 | hsa_1144 | D04048 | 0 | hsa_5743 | D00329 |
| 1 | hsa_2559 | D00706 | 0 | hsa_1144 | D07552 | 0 | hsa_5743 | D00713 |
| 1 | hsa_2559 | D00713 | 0 | hsa_1144 | D04300 | 0 | hsa_5743 | D06392 |
| 1 | hsa_2559 | D00714 | 0 | hsa_1144 | D07999 | 0 | hsa_5743 | D00557 |
| 1 | hsa_2559 | D01071 | 0 | hsa_1144 | D04303 | 0 | hsa_5743 | D01593 |
| 1 | hsa_2559 | D01230 | 0 | hsa_1144 | D01145 | 0 | hsa_5743 | D10613 |
| 1 | hsa_2559 | D01245 | 0 | hsa_1144 | D01278 | 0 | hsa_5743 | D02910 |
| 1 | hsa_2559 | D01253 | 0 | hsa_1144 | D03737 | 0 | hsa_5743 | D07978 |
| 1 | hsa_2559 | D01254 | 0 | hsa_1145 | D10613 | 0 | hsa_5743 | D09035 |
| 1 | hsa_2559 | D01268 | 0 | hsa_1145 | D07971 | 0 | hsa_6261 | D04721 |
| 1 | hsa_2559 | D01278 | 0 | hsa_1145 | D06388 | 0 | hsa_6261 | D10626 |
| 1 | hsa_2559 | D01279 | 0 | hsa_1145 | D08435 | 0 | hsa_6261 | D00499 |
| 1 | hsa_2559 | D01286 | 0 | hsa_1145 | D02617 | 0 | hsa_6261 | D04131 |
| 1 | hsa_2559 | D01292 | 0 | hsa_1145 | D01293 | 0 | hsa_6261 | D07552 |
| 1 | hsa_2559 | D01293 | 0 | hsa_1145 | D02252 | 0 | hsa_6261 | D01293 |
| 1 | hsa_2559 | D01310 | 0 | hsa_1145 | D01286 | 0 | hsa_6261 | D05145 |
| 1 | hsa_2559 | D01316 | 0 | hsa_1145 | D08448 | 0 | hsa_6261 | D01230 |
| 1 | hsa_2559 | D01328 | 0 | hsa_1145 | D08422 | 0 | hsa_6261 | D10627 |
| 1 | hsa_2559 | D01354 | 0 | hsa_1145 | D07283 | 0 | hsa_6261 | D00470 |
| 1 | hsa_2559 | D01372 | 0 | hsa_1145 | D02275 | 0 | hsa_6261 | D09918 |
| 1 | hsa_2559 | D01408 | 0 | hsa_1145 | D07509 | 0 | hsa_6261 | D10702 |
| 1 | hsa_2559 | D01514 | 0 | hsa_1145 | D09367 | 0 | hsa_6261 | D09367 |
| 1 | hsa_2559 | D01564 | 0 | hsa_1145 | D00537 | 0 | hsa_6261 | D01316 |
| 1 | hsa_2559 | D01593 | 0 | hsa_1145 | D00557 | 0 | hsa_6261 | D08174 |
| 1 | hsa_2559 | D01657 | 0 | hsa_1145 | D07494 | 0 | hsa_6261 | D00763 |
| 1 | hsa_2559 | D01740 | 0 | hsa_1145 | D08283 | 0 | hsa_6261 | D07979 |
| 1 | hsa_2559 | D01744 | 0 | hsa_1145 | D02616 | 0 | hsa_6261 | D07962 |
| 1 | hsa_2559 | D01758 | 0 | hsa_1145 | D08116 | 0 | hsa_6261 | D00370 |
| 1 | hsa_2559 | D02252 | 0 | hsa_1145 | D06282 | 0 | hsa_6261 | D06172 |
| 1 | hsa_2559 | D02283 | 0 | hsa_1145 | D00848 | 0 | hsa_6261 | D06388 |
| 1 | hsa_2559 | D02594 | 0 | hsa_1145 | D02220 | 0 | hsa_6261 | D00365 |
| 1 | hsa_2559 | D02616 | 0 | hsa_1145 | D04282 | 0 | hsa_6261 | D07962 |
| 1 | hsa_2559 | D02617 | 0 | hsa_1145 | D10470 | 0 | hsa_6261 | D00537 |
| 1 | hsa_2559 | D02624 | 0 | hsa_1145 | D02202 | 0 | hsa_6261 | D06653 |
| 1 | hsa_2559 | D03155 | 0 | hsa_1145 | D08422 | 0 | hsa_6261 | D06656 |
| 1 | hsa_2559 | D03562 | 0 | hsa_1145 | D08282 | 0 | hsa_6261 | D06653 |
| 1 | hsa_2559 | D03737 | 0 | hsa_1145 | D08422 | 0 | hsa_6261 | D07971 |
| 1 | hsa_2559 | D04257 | 0 | hsa_1145 | D07283 | 0 | hsa_6261 | D09569 |
| 1 | hsa_2559 | D04282 | 0 | hsa_1145 | D01849 | 0 | hsa_6261 | D03732 |
| 1 | hsa_2559 | D04300 | 0 | hsa_1145 | D02696 | 0 | hsa_6261 | D01514 |
| 1 | hsa_2559 | D04721 | 0 | hsa_1145 | D00250 | 0 | hsa_6261 | D08060 |
| 1 | hsa_2559 | D04882 | 0 | hsa_1145 | D08270 | 0 | hsa_6261 | D04728 |
| 1 | hsa_2559 | D05028 | 0 | hsa_1145 | D02096 | 0 | hsa_6261 | D01758 |
| 1 | hsa_2559 | D06106 | 0 | hsa_1145 | D08340 | 0 | hsa_6261 | D10693 |
| 1 | hsa_2559 | D07326 | 0 | hsa_1145 | D04849 | 0 | hsa_6261 | D01554 |
| 1 | hsa_2559 | D07409 | 0 | hsa_1145 | D00549 | 0 | hsa_6261 | D01278 |
| 1 | hsa_2559 | D07784 | 0 | hsa_1145 | D00418 | 0 | hsa_6261 | D08596 |
| 1 | hsa_2559 | D08145 | 0 | hsa_1145 | D10470 | 0 | hsa_6261 | D08100 |
| 1 | hsa_2559 | D08283 | 0 | hsa_1145 | D04303 | 0 | hsa_6261 | D06146 |
| 1 | hsa_2559 | D08356 | 0 | hsa_1145 | D00808 | 0 | hsa_6261 | D09382 |
| 1 | hsa_2559 | D08481 | 0 | hsa_1145 | D00387 | 0 | hsa_6261 | D07962 |
| 1 | hsa_2559 | D08507 | 0 | hsa_1145 | D00387 | 0 | hsa_6261 | D01071 |
| 1 | hsa_2559 | D08690 | 0 | hsa_1145 | D08060 | 0 | hsa_6261 | D10111 |
| 1 | hsa_2559 | D08840 | 0 | hsa_1145 | D01604 | 0 | hsa_6261 | D03732 |
| 1 | hsa_2559 | D10194 | 0 | hsa_1145 | D02410 | 0 | hsa_6261 | D10702 |
| 1 | hsa_2560 | D00267 | 0 | hsa_1145 | D01303 | 0 | hsa_6261 | D04728 |
| 1 | hsa_2560 | D00280 | 0 | hsa_1145 | D06388 | 0 | hsa_6261 | D01408 |
| 1 | hsa_2560 | D00311 | 0 | hsa_1145 | D01104 | 0 | hsa_6261 | D00611 |
| 1 | hsa_2560 | D00365 | 0 | hsa_1145 | D00612 | 0 | hsa_6261 | D04728 |
| 1 | hsa_2560 | D00370 | 0 | hsa_1146 | D07409 | 0 | hsa_6261 | D00011 |
| 1 | hsa_2560 | D00376 | 0 | hsa_1146 | D08596 | 0 | hsa_6261 | D07447 |
| 1 | hsa_2560 | D00387 | 0 | hsa_1146 | D03914 | 0 | hsa_6261 | D04282 |
| 1 | hsa_2560 | D00430 | 0 | hsa_1146 | D07552 | 0 | hsa_6261 | D01326 |
| 1 | hsa_2560 | D00457 | 0 | hsa_1146 | D01455 | 0 | hsa_6261 | D00267 |
| 1 | hsa_2560 | D00470 | 0 | hsa_1146 | D03737 | 0 | hsa_6261 | D07442 |
| 1 | hsa_2560 | D00500 | 0 | hsa_1146 | D00552 | 0 | hsa_6261 | D02780 |
| 1 | hsa_2560 | D00506 | 0 | hsa_1146 | D02537 | 0 | hsa_6262 | D03492 |
| 1 | hsa_2560 | D00531 | 0 | hsa_1146 | D08215 | 0 | hsa_6262 | D02780 |
| 1 | hsa_2560 | D00532 | 0 | hsa_1146 | D02274 | 0 | hsa_6262 | D06517 |
| 1 | hsa_2560 | D00555 | 0 | hsa_1146 | D08667 | 0 | hsa_6262 | D08048 |
| 1 | hsa_2560 | D00557 | 0 | hsa_1146 | D01908 | 0 | hsa_6262 | D09789 |
| 1 | hsa_2560 | D00693 | 0 | hsa_1146 | D04300 | 0 | hsa_6262 | D09612 |
| 1 | hsa_2560 | D00694 | 0 | hsa_1146 | D09215 | 0 | hsa_6262 | D01604 |
| 1 | hsa_2560 | D00695 | 0 | hsa_1146 | D04048 | 0 | hsa_6262 | D00696 |
| 1 | hsa_2560 | D00696 | 0 | hsa_1146 | D01230 | 0 | hsa_6262 | D08448 |
| 1 | hsa_2560 | D00697 | 0 | hsa_1146 | D00252 | 0 | hsa_6262 | D00294 |
| 1 | hsa_2560 | D00700 | 0 | hsa_1146 | D00336 | 0 | hsa_6262 | D08145 |
| 1 | hsa_2560 | D00701 | 0 | hsa_1146 | D08669 | 0 | hsa_6262 | D00696 |
| 1 | hsa_2560 | D00706 | 0 | hsa_1146 | D07979 | 0 | hsa_6262 | D08422 |
| 1 | hsa_2560 | D00713 | 0 | hsa_1146 | D08596 | 0 | hsa_6262 | D01372 |
| 1 | hsa_2560 | D00714 | 0 | hsa_1146 | D01316 | 0 | hsa_6262 | D08145 |
| 1 | hsa_2560 | D01071 | 0 | hsa_1146 | D07595 | 0 | hsa_6262 | D00492 |
| 1 | hsa_2560 | D01230 | 0 | hsa_1146 | D01328 | 0 | hsa_6262 | D00766 |
| 1 | hsa_2560 | D01245 | 0 | hsa_1146 | D04308 | 0 | hsa_6262 | D02385 |
| 1 | hsa_2560 | D01253 | 0 | hsa_1146 | D02103 | 0 | hsa_6262 | D07979 |
| 1 | hsa_2560 | D01254 | 0 | hsa_1146 | D01856 | 0 | hsa_6262 | D06282 |
| 1 | hsa_2560 | D01268 | 0 | hsa_1146 | D00470 | 0 | hsa_6262 | D01785 |
| 1 | hsa_2560 | D01278 | 0 | hsa_1146 | D08987 | 0 | hsa_6262 | D00532 |
| 1 | hsa_2560 | D01279 | 0 | hsa_1146 | D00760 | 0 | hsa_6262 | D04048 |
| 1 | hsa_2560 | D01286 | 0 | hsa_1146 | D00701 | 0 | hsa_6262 | D10693 |
| 1 | hsa_2560 | D01292 | 0 | hsa_1146 | D08669 | 0 | hsa_6262 | D00303 |
| 1 | hsa_2560 | D01293 | 0 | hsa_1146 | D00011 | 0 | hsa_6262 | D03742 |
| 1 | hsa_2560 | D01310 | 0 | hsa_1146 | D01657 | 0 | hsa_6262 | D09367 |
| 1 | hsa_2560 | D01316 | 0 | hsa_1146 | D06392 | 0 | hsa_6262 | D09569 |
| 1 | hsa_2560 | D01328 | 0 | hsa_1146 | D05453 | 0 | hsa_6262 | D00706 |
| 1 | hsa_2560 | D01354 | 0 | hsa_1146 | D07283 | 0 | hsa_6262 | D02283 |
| 1 | hsa_2560 | D01372 | 0 | hsa_1146 | D01230 | 0 | hsa_6262 | D05442 |
| 1 | hsa_2560 | D01408 | 0 | hsa_1146 | D03547 | 0 | hsa_6262 | D01479 |
| 1 | hsa_2560 | D01514 | 0 | hsa_1146 | D04282 | 0 | hsa_6262 | D02103 |
| 1 | hsa_2560 | D01564 | 0 | hsa_1146 | D00551 | 0 | hsa_6262 | D00470 |
| 1 | hsa_2560 | D01593 | 0 | hsa_1146 | D02537 | 0 | hsa_6262 | D01104 |
| 1 | hsa_2560 | D01657 | 0 | hsa_1146 | D08690 | 0 | hsa_6262 | D08111 |
| 1 | hsa_2560 | D01740 | 0 | hsa_1146 | D01554 | 0 | hsa_6262 | D07520 |
| 1 | hsa_2560 | D01744 | 0 | hsa_1146 | D01268 | 0 | hsa_6262 | D09383 |
| 1 | hsa_2560 | D01758 | 0 | hsa_18 | D03742 | 0 | hsa_6262 | D08892 |
| 1 | hsa_2560 | D02252 | 0 | hsa_18 | D00701 | 0 | hsa_6262 | D04048 |
| 1 | hsa_2560 | D02253 | 0 | hsa_18 | D06282 | 0 | hsa_6262 | D00533 |
| 1 | hsa_2560 | D02283 | 0 | hsa_18 | D03746 | 0 | hsa_6262 | D09382 |
| 1 | hsa_2560 | D02594 | 0 | hsa_18 | D00499 | 0 | hsa_6262 | D00331 |
| 1 | hsa_2560 | D02616 | 0 | hsa_18 | D08935 | 0 | hsa_6262 | D00760 |
| 1 | hsa_2560 | D02617 | 0 | hsa_18 | D03155 | 0 | hsa_6262 | D03746 |
| 1 | hsa_2560 | D02624 | 0 | hsa_18 | D00549 | 0 | hsa_6262 | D00767 |
| 1 | hsa_2560 | D03155 | 0 | hsa_18 | D04721 | 0 | hsa_6262 | D08155 |
| 1 | hsa_2560 | D03562 | 0 | hsa_18 | D00643 | 0 | hsa_6262 | D00555 |
| 1 | hsa_2560 | D03737 | 0 | hsa_18 | D07943 | 0 | hsa_6262 | D10111 |
| 1 | hsa_2560 | D04257 | 0 | hsa_18 | D07552 | 0 | hsa_6263 | D07450 |
| 1 | hsa_2560 | D04282 | 0 | hsa_18 | D01145 | 0 | hsa_6263 | D04955 |
| 1 | hsa_2560 | D04300 | 0 | hsa_18 | D01740 | 0 | hsa_6263 | D06172 |
| 1 | hsa_2560 | D04721 | 0 | hsa_18 | D01104 | 0 | hsa_6263 | D00700 |
| 1 | hsa_2560 | D04882 | 0 | hsa_18 | D07993 | 0 | hsa_6263 | D00250 |
| 1 | hsa_2560 | D05028 | 0 | hsa_18 | D00267 | 0 | hsa_6263 | D00532 |
| 1 | hsa_2560 | D06106 | 0 | hsa_18 | D01564 | 0 | hsa_6263 | D00533 |
| 1 | hsa_2560 | D07326 | 0 | hsa_18 | D09215 | 0 | hsa_6263 | D10627 |
| 1 | hsa_2560 | D07409 | 0 | hsa_18 | D00549 | 0 | hsa_6263 | D00303 |
| 1 | hsa_2560 | D07784 | 0 | hsa_18 | D07845 | 0 | hsa_6263 | D00365 |
| 1 | hsa_2560 | D08145 | 0 | hsa_18 | D10111 | 0 | hsa_6263 | D03737 |
| 1 | hsa_2560 | D08283 | 0 | hsa_18 | D00506 | 0 | hsa_6263 | D01455 |
| 1 | hsa_2560 | D08356 | 0 | hsa_18 | D03492 | 0 | hsa_6263 | D07678 |
| 1 | hsa_2560 | D08481 | 0 | hsa_18 | D08060 | 0 | hsa_6263 | D01243 |
| 1 | hsa_2560 | D08507 | 0 | hsa_18 | D04918 | 0 | hsa_6263 | D00555 |
| 1 | hsa_2560 | D08690 | 0 | hsa_18 | D01604 | 0 | hsa_6263 | D10702 |
| 1 | hsa_2560 | D08840 | 0 | hsa_18 | D00637 | 0 | hsa_6263 | D00643 |
| 1 | hsa_2560 | D10194 | 0 | hsa_18 | D10111 | 0 | hsa_6263 | D05482 |
| 1 | hsa_2561 | D00267 | 0 | hsa_18 | D02910 | 0 | hsa_6263 | D09918 |
| 1 | hsa_2561 | D00280 | 0 | hsa_18 | D09035 | 0 | hsa_6263 | D09368 |
| 1 | hsa_2561 | D00311 | 0 | hsa_18 | D08174 | 0 | hsa_6263 | D02716 |
| 1 | hsa_2561 | D00365 | 0 | hsa_18 | D00011 | 0 | hsa_6263 | D02973 |
| 1 | hsa_2561 | D00370 | 0 | hsa_18 | D08340 | 0 | hsa_6263 | D00250 |
| 1 | hsa_2561 | D00376 | 0 | hsa_18 | D02780 | 0 | hsa_6263 | D08935 |
| 1 | hsa_2561 | D00387 | 0 | hsa_18 | D00500 | 0 | hsa_6263 | D02347 |
| 1 | hsa_2561 | D00430 | 0 | hsa_18 | D07784 | 0 | hsa_6263 | D00252 |
| 1 | hsa_2561 | D00457 | 0 | hsa_18 | D03037 | 0 | hsa_6263 | D06282 |
| 1 | hsa_2561 | D00470 | 0 | hsa_18 | D08507 | 0 | hsa_6263 | D01564 |
| 1 | hsa_2561 | D00500 | 0 | hsa_18 | D01145 | 0 | hsa_6263 | D00267 |
| 1 | hsa_2561 | D00506 | 0 | hsa_18 | D02696 | 0 | hsa_6263 | D05028 |
| 1 | hsa_2561 | D00531 | 0 | hsa_2554 | D06204 | 0 | hsa_6263 | D00280 |
| 1 | hsa_2561 | D00532 | 0 | hsa_2554 | D04918 | 0 | hsa_6263 | D01326 |
| 1 | hsa_2561 | D00555 | 0 | hsa_2554 | D00760 | 0 | hsa_6263 | D03746 |
| 1 | hsa_2561 | D00557 | 0 | hsa_2554 | D06204 | 0 | hsa_6263 | D00336 |
| 1 | hsa_2561 | D00693 | 0 | hsa_2554 | D08275 | 0 | hsa_6263 | D02616 |
| 1 | hsa_2561 | D00694 | 0 | hsa_2554 | D01856 | 0 | hsa_6263 | D03562 |
| 1 | hsa_2561 | D00695 | 0 | hsa_2554 | D00293 | 0 | hsa_6263 | D00293 |
| 1 | hsa_2561 | D00696 | 0 | hsa_2554 | D07971 | 0 | hsa_6263 | D03037 |
| 1 | hsa_2561 | D00697 | 0 | hsa_2554 | D10702 | 0 | hsa_6263 | D07409 |
| 1 | hsa_2561 | D00700 | 0 | hsa_2554 | D00733 | 0 | hsa_6263 | D07520 |
| 1 | hsa_2561 | D00701 | 0 | hsa_2554 | D08217 | 0 | hsa_6263 | D00643 |
| 1 | hsa_2561 | D00706 | 0 | hsa_2554 | D02973 | 0 | hsa_6263 | D04303 |
| 1 | hsa_2561 | D00713 | 0 | hsa_2554 | D00766 | 0 | hsa_6263 | D06392 |
| 1 | hsa_2561 | D00714 | 0 | hsa_2554 | D00329 | 0 | hsa_6263 | D05453 |
| 1 | hsa_2561 | D01071 | 0 | hsa_2554 | D09367 | 0 | hsa_6263 | D04849 |
| 1 | hsa_2561 | D01230 | 0 | hsa_2554 | D00331 | 0 | hsa_6263 | D08215 |
| 1 | hsa_2561 | D01245 | 0 | hsa_2554 | D03746 | 0 | hsa_6263 | D04955 |
| 1 | hsa_2561 | D01253 | 0 | hsa_2554 | D02220 | 0 | hsa_6263 | D07326 |
| 1 | hsa_2561 | D01254 | 0 | hsa_2554 | D07845 | 0 | hsa_6263 | D00492 |
| 1 | hsa_2561 | D01268 | 0 | hsa_2554 | D07185 | 0 | hsa_6263 | D08356 |
| 1 | hsa_2561 | D01278 | 0 | hsa_2554 | D02696 | 0 | hsa_6323 | D07447 |
| 1 | hsa_2561 | D01279 | 0 | hsa_2554 | D07595 | 0 | hsa_6323 | D01326 |
| 1 | hsa_2561 | D01286 | 0 | hsa_2554 | D02696 | 0 | hsa_6323 | D06388 |
| 1 | hsa_2561 | D01292 | 0 | hsa_2554 | D08282 | 0 | hsa_6323 | D00499 |
| 1 | hsa_2561 | D01293 | 0 | hsa_2554 | D01553 | 0 | hsa_6323 | D08116 |
| 1 | hsa_2561 | D01310 | 0 | hsa_2554 | D03746 | 0 | hsa_6323 | D08275 |
| 1 | hsa_2561 | D01316 | 0 | hsa_2554 | D03492 | 0 | hsa_6323 | D09367 |
| 1 | hsa_2561 | D01328 | 0 | hsa_2554 | D00064 | 0 | hsa_6323 | D00380 |
| 1 | hsa_2561 | D01354 | 0 | hsa_2554 | D07272 | 0 | hsa_6323 | D10112 |
| 1 | hsa_2561 | D01372 | 0 | hsa_2554 | D05453 | 0 | hsa_6323 | D08174 |
| 1 | hsa_2561 | D01408 | 0 | hsa_2554 | D03742 | 0 | hsa_6323 | D03492 |
| 1 | hsa_2561 | D01514 | 0 | hsa_2554 | D00553 | 0 | hsa_6323 | D06204 |
| 1 | hsa_2561 | D01564 | 0 | hsa_2554 | D08490 | 0 | hsa_6323 | D00499 |
| 1 | hsa_2561 | D01593 | 0 | hsa_2554 | D07886 | 0 | hsa_6323 | D02220 |
| 1 | hsa_2561 | D01657 | 0 | hsa_2554 | D08458 | 0 | hsa_6323 | D07881 |
| 1 | hsa_2561 | D01740 | 0 | hsa_2554 | D00011 | 0 | hsa_6323 | D00331 |
| 1 | hsa_2561 | D01744 | 0 | hsa_2554 | D00766 | 0 | hsa_6323 | D09383 |
| 1 | hsa_2561 | D01758 | 0 | hsa_2554 | D07886 | 0 | hsa_6323 | D01908 |
| 1 | hsa_2561 | D02252 | 0 | hsa_2554 | D09612 | 0 | hsa_6323 | D08459 |
| 1 | hsa_2561 | D02253 | 0 | hsa_2554 | D04226 | 0 | hsa_6323 | D04131 |
| 1 | hsa_2561 | D02283 | 0 | hsa_2554 | D02410 | 0 | hsa_6323 | D08174 |
| 1 | hsa_2561 | D02594 | 0 | hsa_2555 | D03742 | 0 | hsa_6323 | D04728 |
| 1 | hsa_2561 | D02616 | 0 | hsa_2555 | D07999 | 0 | hsa_6323 | D09789 |
| 1 | hsa_2561 | D02617 | 0 | hsa_2555 | D00329 | 0 | hsa_6336 | D08127 |
| 1 | hsa_2561 | D02624 | 0 | hsa_2555 | D08892 | 0 | hsa_6336 | D01326 |
| 1 | hsa_2561 | D03155 | 0 | hsa_2555 | D01243 | 0 | hsa_6336 | D00531 |
| 1 | hsa_2561 | D03562 | 0 | hsa_2555 | D07509 | 0 | hsa_6336 | D00714 |
| 1 | hsa_2561 | D03737 | 0 | hsa_2555 | D08490 | 0 | hsa_6336 | D01856 |
| 1 | hsa_2561 | D04257 | 0 | hsa_2555 | D08282 | 0 | hsa_6336 | D02292 |
| 1 | hsa_2561 | D04282 | 0 | hsa_2555 | D06388 | 0 | hsa_6336 | D01243 |
| 1 | hsa_2561 | D04300 | 0 | hsa_2555 | D00303 | 0 | hsa_6336 | D01243 |
| 1 | hsa_2561 | D04721 | 0 | hsa_2555 | D08490 | 0 | hsa_6336 | D07272 |
| 1 | hsa_2561 | D04882 | 0 | hsa_2555 | D02220 | 0 | hsa_6336 | D00252 |
| 1 | hsa_2561 | D05028 | 0 | hsa_2555 | D07447 | 0 | hsa_6336 | D05145 |
| 1 | hsa_2561 | D06106 | 0 | hsa_2555 | D00250 | 0 | hsa_6336 | D00695 |
| 1 | hsa_2561 | D07326 | 0 | hsa_2555 | D02973 | 0 | hsa_6336 | D00331 |
| 1 | hsa_2561 | D07409 | 0 | hsa_2555 | D08394 | 0 | hsa_6336 | D00370 |
| 1 | hsa_2561 | D07784 | 0 | hsa_2555 | D07509 | 0 | hsa_6336 | D04905 |
| 1 | hsa_2561 | D08145 | 0 | hsa_2555 | D04728 | 0 | hsa_6336 | D07999 |
| 1 | hsa_2561 | D08283 | 0 | hsa_2555 | D00252 | 0 | hsa_6336 | D02910 |
| 1 | hsa_2561 | D08356 | 0 | hsa_2555 | D08422 | 0 | hsa_6336 | D08964 |
| 1 | hsa_2561 | D08481 | 0 | hsa_2555 | D10693 | 0 | hsa_6336 | D00532 |
| 1 | hsa_2561 | D08507 | 0 | hsa_2555 | D00759 | 0 | hsa_6336 | D06388 |
| 1 | hsa_2561 | D08690 | 0 | hsa_2555 | D08340 | 0 | hsa_6336 | D03562 |
| 1 | hsa_2561 | D08840 | 0 | hsa_2555 | D08669 | 0 | hsa_6336 | D00700 |
| 1 | hsa_2561 | D10194 | 0 | hsa_2555 | D01810 | 0 | hsa_6336 | D04048 |
| 1 | hsa_2562 | D00267 | 0 | hsa_2555 | D00199 | 0 | hsa_6336 | D01604 |
| 1 | hsa_2562 | D00280 | 0 | hsa_2555 | D08448 | 0 | hsa_6336 | D02103 |
| 1 | hsa_2562 | D00311 | 0 | hsa_2555 | D08275 | 0 | hsa_6336 | D00399 |
| 1 | hsa_2562 | D00365 | 0 | hsa_2555 | D07520 | 0 | hsa_6336 | D00293 |
| 1 | hsa_2562 | D00370 | 0 | hsa_2556 | D08421 | 0 | hsa_6336 | D08283 |
| 1 | hsa_2562 | D00376 | 0 | hsa_2556 | D02347 | 0 | hsa_6336 | D01408 |
| 1 | hsa_2562 | D00387 | 0 | hsa_2556 | D07881 | 0 | hsa_6336 | D01268 |
| 1 | hsa_2562 | D00430 | 0 | hsa_2556 | D00555 | 0 | hsa_6336 | D02973 |
| 1 | hsa_2562 | D00457 | 0 | hsa_2556 | D08507 | 0 | hsa_6336 | D07943 |
| 1 | hsa_2562 | D00470 | 0 | hsa_2556 | D09918 | 0 | hsa_6336 | D04955 |
| 1 | hsa_2562 | D00500 | 0 | hsa_2556 | D01740 | 0 | hsa_6336 | D04741 |
| 1 | hsa_2562 | D00506 | 0 | hsa_2556 | D00098 | 0 | hsa_6336 | D08892 |
| 1 | hsa_2562 | D00531 | 0 | hsa_2556 | D00418 | 0 | hsa_6336 | D01354 |
| 1 | hsa_2562 | D00532 | 0 | hsa_2556 | D06282 | 0 | hsa_6336 | D01372 |
| 1 | hsa_2562 | D00555 | 0 | hsa_2556 | D08377 | 0 | hsa_6336 | D06652 |
| 1 | hsa_2562 | D00557 | 0 | hsa_2556 | D00631 | 0 | hsa_6336 | D01604 |
| 1 | hsa_2562 | D00693 | 0 | hsa_2556 | D01293 | 0 | hsa_6336 | D08101 |
| 1 | hsa_2562 | D00694 | 0 | hsa_2556 | D00532 | 0 | hsa_6337 | D07678 |
| 1 | hsa_2562 | D00695 | 0 | hsa_2556 | D09035 | 0 | hsa_6337 | D02616 |
| 1 | hsa_2562 | D00696 | 0 | hsa_2556 | D06282 | 0 | hsa_6337 | D08669 |
| 1 | hsa_2562 | D00697 | 0 | hsa_2556 | D08422 | 0 | hsa_6337 | D04048 |
| 1 | hsa_2562 | D00700 | 0 | hsa_2556 | D10627 | 0 | hsa_6337 | D10194 |
| 1 | hsa_2562 | D00701 | 0 | hsa_2556 | D02537 | 0 | hsa_6337 | D00696 |
| 1 | hsa_2562 | D00706 | 0 | hsa_2556 | D04849 | 0 | hsa_6337 | D00199 |
| 1 | hsa_2562 | D00713 | 0 | hsa_2556 | D08155 | 0 | hsa_6337 | D07283 |
| 1 | hsa_2562 | D00714 | 0 | hsa_2556 | D00294 | 0 | hsa_6337 | D00250 |
| 1 | hsa_2562 | D01071 | 0 | hsa_2556 | D00848 | 0 | hsa_6337 | D02973 |
| 1 | hsa_2562 | D01230 | 0 | hsa_2556 | D00763 | 0 | hsa_6337 | D06388 |
| 1 | hsa_2562 | D01245 | 0 | hsa_2556 | D05077 | 0 | hsa_6337 | D02780 |
| 1 | hsa_2562 | D01253 | 0 | hsa_2556 | D00555 | 0 | hsa_6337 | D04905 |
| 1 | hsa_2562 | D01254 | 0 | hsa_2556 | D05703 | 0 | hsa_6337 | D05482 |
| 1 | hsa_2562 | D01268 | 0 | hsa_2556 | D00733 | 0 | hsa_6337 | D02096 |
| 1 | hsa_2562 | D01278 | 0 | hsa_2556 | D08394 | 0 | hsa_6337 | D01564 |
| 1 | hsa_2562 | D01279 | 0 | hsa_2556 | D08098 | 0 | hsa_6337 | D01292 |
| 1 | hsa_2562 | D01286 | 0 | hsa_2556 | D03547 | 0 | hsa_6337 | D08669 |
| 1 | hsa_2562 | D01292 | 0 | hsa_2556 | D04308 | 0 | hsa_6337 | D10613 |
| 1 | hsa_2562 | D01293 | 0 | hsa_2556 | D01071 | 0 | hsa_6337 | D04918 |
| 1 | hsa_2562 | D01310 | 0 | hsa_2556 | D00533 | 0 | hsa_6337 | D01810 |
| 1 | hsa_2562 | D01316 | 0 | hsa_2556 | D10626 | 0 | hsa_6337 | D01455 |
| 1 | hsa_2562 | D01328 | 0 | hsa_2556 | D06146 | 0 | hsa_6337 | D08101 |
| 1 | hsa_2562 | D01354 | 0 | hsa_2556 | D01245 | 0 | hsa_6337 | D02594 |
| 1 | hsa_2562 | D01372 | 0 | hsa_2556 | D08101 | 0 | hsa_6337 | D00693 |
| 1 | hsa_2562 | D01408 | 0 | hsa_2556 | D08490 | 0 | hsa_6337 | D09569 |
| 1 | hsa_2562 | D01514 | 0 | hsa_2556 | D06656 | 0 | hsa_6337 | D08101 |
| 1 | hsa_2562 | D01564 | 0 | hsa_2556 | D00418 | 0 | hsa_6337 | D01278 |
| 1 | hsa_2562 | D01593 | 0 | hsa_2556 | D04300 | 0 | hsa_6337 | D09612 |
| 1 | hsa_2562 | D01657 | 0 | hsa_2556 | D08448 | 0 | hsa_6337 | D00555 |
| 1 | hsa_2562 | D01740 | 0 | hsa_2556 | D09612 | 0 | hsa_6337 | D00304 |
| 1 | hsa_2562 | D01744 | 0 | hsa_2557 | D03746 | 0 | hsa_6337 | D00329 |
| 1 | hsa_2562 | D01758 | 0 | hsa_2557 | D08964 | 0 | hsa_6337 | D03914 |
| 1 | hsa_2562 | D02252 | 0 | hsa_2557 | D03100 | 0 | hsa_6337 | D08283 |
| 1 | hsa_2562 | D02253 | 0 | hsa_2557 | D01354 | 0 | hsa_6337 | D08490 |
| 1 | hsa_2562 | D02283 | 0 | hsa_2557 | D05442 | 0 | hsa_6337 | D04918 |
| 1 | hsa_2562 | D02594 | 0 | hsa_2557 | D00303 | 0 | hsa_6337 | D02347 |
| 1 | hsa_2562 | D02616 | 0 | hsa_2557 | D07272 | 0 | hsa_6337 | D01254 |
| 1 | hsa_2562 | D02617 | 0 | hsa_2557 | D07881 | 0 | hsa_6337 | D00329 |
| 1 | hsa_2562 | D02624 | 0 | hsa_2557 | D00199 | 0 | hsa_6337 | D00531 |
| 1 | hsa_2562 | D03155 | 0 | hsa_2557 | D01758 | 0 | hsa_6337 | D04918 |
| 1 | hsa_2562 | D03562 | 0 | hsa_2557 | D07058 | 0 | hsa_6337 | D07894 |
| 1 | hsa_2562 | D03737 | 0 | hsa_2557 | D00531 | 0 | hsa_6337 | D02202 |
| 1 | hsa_2562 | D04257 | 0 | hsa_2557 | D00376 | 0 | hsa_6337 | D00637 |
| 1 | hsa_2562 | D04282 | 0 | hsa_2557 | D02780 | 0 | hsa_6337 | D00430 |
| 1 | hsa_2562 | D04300 | 0 | hsa_2557 | D01173 | 0 | hsa_6337 | D10702 |
| 1 | hsa_2562 | D04721 | 0 | hsa_2557 | D02045 | 0 | hsa_6337 | D08340 |
| 1 | hsa_2562 | D04882 | 0 | hsa_2557 | D00376 | 0 | hsa_6337 | D10112 |
| 1 | hsa_2562 | D05028 | 0 | hsa_2557 | D01455 | 0 | hsa_6337 | D01071 |
| 1 | hsa_2562 | D06106 | 0 | hsa_2557 | D00387 | 0 | hsa_6337 | D02696 |
| 1 | hsa_2562 | D07326 | 0 | hsa_2557 | D06282 | 0 | hsa_6337 | D04849 |
| 1 | hsa_2562 | D07409 | 0 | hsa_2557 | D02220 | 0 | hsa_6337 | D05028 |
| 1 | hsa_2562 | D07784 | 0 | hsa_2557 | D02616 | 0 | hsa_6337 | D02252 |
| 1 | hsa_2562 | D08145 | 0 | hsa_2557 | D05145 | 0 | hsa_6337 | D01514 |
| 1 | hsa_2562 | D08283 | 0 | hsa_2557 | D00331 | 0 | hsa_6337 | D08667 |
| 1 | hsa_2562 | D08356 | 0 | hsa_2557 | D07447 | 0 | hsa_6337 | D06204 |
| 1 | hsa_2562 | D08481 | 0 | hsa_2557 | D00329 | 0 | hsa_6337 | D06392 |
| 1 | hsa_2562 | D08507 | 0 | hsa_2557 | D09382 | 0 | hsa_6337 | D10626 |
| 1 | hsa_2562 | D08690 | 0 | hsa_2557 | D00550 | 0 | hsa_6337 | D02252 |
| 1 | hsa_2562 | D08840 | 0 | hsa_2557 | D01593 | 0 | hsa_6337 | D08669 |
| 1 | hsa_2562 | D10194 | 0 | hsa_2557 | D07886 | 0 | hsa_6337 | D08127 |
| 1 | hsa_2563 | D00267 | 0 | hsa_2557 | D02385 | 0 | hsa_6337 | D01604 |
| 1 | hsa_2563 | D00280 | 0 | hsa_2557 | D05145 | 0 | hsa_6337 | D01810 |
| 1 | hsa_2563 | D00311 | 0 | hsa_2557 | D00380 | 0 | hsa_6338 | D00551 |
| 1 | hsa_2563 | D00365 | 0 | hsa_2557 | D04048 | 0 | hsa_6338 | D00376 |
| 1 | hsa_2563 | D00370 | 0 | hsa_2557 | D08127 | 0 | hsa_6338 | D07784 |
| 1 | hsa_2563 | D00376 | 0 | hsa_2557 | D02096 | 0 | hsa_6338 | D02910 |
| 1 | hsa_2563 | D00387 | 0 | hsa_2557 | D08048 | 0 | hsa_6338 | D08101 |
| 1 | hsa_2563 | D00430 | 0 | hsa_2557 | D10627 | 0 | hsa_6338 | D07894 |
| 1 | hsa_2563 | D00457 | 0 | hsa_2557 | D00760 | 0 | hsa_6338 | D01303 |
| 1 | hsa_2563 | D00470 | 0 | hsa_2557 | D00303 | 0 | hsa_6338 | D00533 |
| 1 | hsa_2563 | D00500 | 0 | hsa_2557 | D00011 | 0 | hsa_6338 | D09382 |
| 1 | hsa_2563 | D00506 | 0 | hsa_2557 | D08215 | 0 | hsa_6338 | D04308 |
| 1 | hsa_2563 | D00531 | 0 | hsa_2557 | D00549 | 0 | hsa_6338 | D07784 |
| 1 | hsa_2563 | D00532 | 0 | hsa_2557 | D09367 | 0 | hsa_6338 | D02410 |
| 1 | hsa_2563 | D00555 | 0 | hsa_2557 | D04918 | 0 | hsa_6338 | D10112 |
| 1 | hsa_2563 | D00557 | 0 | hsa_2557 | D08340 | 0 | hsa_6338 | D00098 |
| 1 | hsa_2563 | D00693 | 0 | hsa_2557 | D07971 | 0 | hsa_6338 | D07058 |
| 1 | hsa_2563 | D00694 | 0 | hsa_2557 | D10111 | 0 | hsa_6338 | D07494 |
| 1 | hsa_2563 | D00695 | 0 | hsa_2557 | D00252 | 0 | hsa_6338 | D09539 |
| 1 | hsa_2563 | D00696 | 0 | hsa_2557 | D08155 | 0 | hsa_6338 | D09368 |
| 1 | hsa_2563 | D00697 | 0 | hsa_2557 | D01849 | 0 | hsa_6338 | D08127 |
| 1 | hsa_2563 | D00700 | 0 | hsa_2557 | D05028 | 0 | hsa_6338 | D00710 |
| 1 | hsa_2563 | D00701 | 0 | hsa_2557 | D07979 | 0 | hsa_6338 | D10370 |
| 1 | hsa_2563 | D00706 | 0 | hsa_2557 | D04257 | 0 | hsa_6338 | D08111 |
| 1 | hsa_2563 | D00713 | 0 | hsa_2557 | D00470 | 0 | hsa_6338 | D03037 |
| 1 | hsa_2563 | D00714 | 0 | hsa_2558 | D10111 | 0 | hsa_6338 | D03547 |
| 1 | hsa_2563 | D01071 | 0 | hsa_2558 | D03679 | 0 | hsa_6338 | D01243 |
| 1 | hsa_2563 | D01230 | 0 | hsa_2558 | D01514 | 0 | hsa_6338 | D00555 |
| 1 | hsa_2563 | D01245 | 0 | hsa_2558 | D00250 | 0 | hsa_6338 | D08690 |
| 1 | hsa_2563 | D01253 | 0 | hsa_2558 | D07520 | 0 | hsa_6338 | D07886 |
| 1 | hsa_2563 | D01254 | 0 | hsa_2558 | D08377 | 0 | hsa_6338 | D00386 |
| 1 | hsa_2563 | D01268 | 0 | hsa_2558 | D00336 | 0 | hsa_6338 | D00252 |
| 1 | hsa_2563 | D01278 | 0 | hsa_2558 | D05453 | 0 | hsa_6338 | D05447 |
| 1 | hsa_2563 | D01279 | 0 | hsa_2558 | D09539 | 0 | hsa_6338 | D09367 |
| 1 | hsa_2563 | D01286 | 0 | hsa_2558 | D08964 | 0 | hsa_6338 | D00553 |
| 1 | hsa_2563 | D01292 | 0 | hsa_2558 | D02274 | 0 | hsa_6338 | D08356 |
| 1 | hsa_2563 | D01293 | 0 | hsa_2558 | D08275 | 0 | hsa_6338 | D08127 |
| 1 | hsa_2563 | D01310 | 0 | hsa_2558 | D07886 | 0 | hsa_6338 | D01316 |
| 1 | hsa_2563 | D01316 | 0 | hsa_2558 | D01104 | 0 | hsa_6338 | D00386 |
| 1 | hsa_2563 | D01328 | 0 | hsa_2558 | D03655 | 0 | hsa_6338 | D08935 |
| 1 | hsa_2563 | D01354 | 0 | hsa_2558 | D06517 | 0 | hsa_6338 | D08127 |
| 1 | hsa_2563 | D01372 | 0 | hsa_2558 | D00098 | 0 | hsa_6338 | D08340 |
| 1 | hsa_2563 | D01408 | 0 | hsa_2558 | D02385 | 0 | hsa_6340 | D10626 |
| 1 | hsa_2563 | D01514 | 0 | hsa_2558 | D03742 | 0 | hsa_6340 | D00252 |
| 1 | hsa_2563 | D01564 | 0 | hsa_2558 | D00303 | 0 | hsa_6340 | D04955 |
| 1 | hsa_2563 | D01593 | 0 | hsa_2558 | D01908 | 0 | hsa_6340 | D00418 |
| 1 | hsa_2563 | D01657 | 0 | hsa_2558 | D02220 | 0 | hsa_6340 | D07962 |
| 1 | hsa_2563 | D01740 | 0 | hsa_2558 | D08275 | 0 | hsa_6340 | D02220 |
| 1 | hsa_2563 | D01744 | 0 | hsa_2558 | D03746 | 0 | hsa_6340 | D08282 |
| 1 | hsa_2563 | D01758 | 0 | hsa_2558 | D01908 | 0 | hsa_6340 | D09918 |
| 1 | hsa_2563 | D02252 | 0 | hsa_2558 | D08596 | 0 | hsa_6340 | D08458 |
| 1 | hsa_2563 | D02253 | 0 | hsa_2558 | D00758 | 0 | hsa_6340 | D08155 |
| 1 | hsa_2563 | D02283 | 0 | hsa_2558 | D02103 | 0 | hsa_6340 | D00531 |
| 1 | hsa_2563 | D02594 | 0 | hsa_2558 | D00492 | 0 | hsa_6340 | D10112 |
| 1 | hsa_2563 | D02616 | 0 | hsa_2558 | D10112 | 0 | hsa_6340 | D01562 |
| 1 | hsa_2563 | D02617 | 0 | hsa_2558 | D00808 | 0 | hsa_6340 | D00376 |
| 1 | hsa_2563 | D02624 | 0 | hsa_2558 | D01145 | 0 | hsa_6340 | D01372 |
| 1 | hsa_2563 | D03155 | 0 | hsa_2558 | D09789 | 0 | hsa_6340 | D07450 |
| 1 | hsa_2563 | D03562 | 0 | hsa_2558 | D03655 | 0 | hsa_6340 | D08101 |
| 1 | hsa_2563 | D03737 | 0 | hsa_2558 | D09382 | 0 | hsa_6340 | D04849 |
| 1 | hsa_2563 | D04257 | 0 | hsa_2558 | D00365 | 0 | hsa_6340 | D01254 |
| 1 | hsa_2563 | D04282 | 0 | hsa_2558 | D10370 | 0 | hsa_6340 | D07678 |
| 1 | hsa_2563 | D04300 | 0 | hsa_2558 | D08481 | 0 | hsa_6340 | D01657 |
| 1 | hsa_2563 | D04721 | 0 | hsa_2558 | D01514 | 0 | hsa_6340 | D10370 |
| 1 | hsa_2563 | D04882 | 0 | hsa_2558 | D07326 | 0 | hsa_6340 | D05442 |
| 1 | hsa_2563 | D05028 | 0 | hsa_2558 | D04308 | 0 | hsa_6340 | D01740 |
| 1 | hsa_2563 | D06106 | 0 | hsa_2558 | D08596 | 0 | hsa_6340 | D02283 |
| 1 | hsa_2563 | D07326 | 0 | hsa_2558 | D00765 | 0 | hsa_6340 | D02202 |
| 1 | hsa_2563 | D07409 | 0 | hsa_2558 | D00701 | 0 | hsa_6340 | D02347 |
| 1 | hsa_2563 | D07784 | 0 | hsa_2558 | D00553 | 0 | hsa_6340 | D07595 |
| 1 | hsa_2563 | D08145 | 0 | hsa_2558 | D04728 | 0 | hsa_6340 | D07962 |
| 1 | hsa_2563 | D08283 | 0 | hsa_2558 | D04955 | 0 | hsa_6340 | D08356 |
| 1 | hsa_2563 | D08356 | 0 | hsa_2559 | D10626 | 0 | hsa_6340 | D00438 |
| 1 | hsa_2563 | D08481 | 0 | hsa_2559 | D03547 | 0 | hsa_6340 | D00506 |
| 1 | hsa_2563 | D08507 | 0 | hsa_2559 | D00365 | 0 | hsa_6340 | D07326 |
| 1 | hsa_2563 | D08690 | 0 | hsa_2559 | D08155 | 0 | hsa_6340 | D01849 |
| 1 | hsa_2563 | D08840 | 0 | hsa_2559 | D09035 | 0 | hsa_6340 | D00250 |
| 1 | hsa_2563 | D10194 | 0 | hsa_2559 | D00643 | 0 | hsa_6340 | D05077 |
| 1 | hsa_2564 | D00267 | 0 | hsa_2559 | D00199 | 0 | hsa_6340 | D08101 |
| 1 | hsa_2564 | D00280 | 0 | hsa_2559 | D04955 | 0 | hsa_6340 | D06172 |
| 1 | hsa_2564 | D00311 | 0 | hsa_2559 | D03562 | 0 | hsa_6340 | D04282 |
| 1 | hsa_2564 | D00365 | 0 | hsa_2559 | D01293 | 0 | hsa_6340 | D00250 |
| 1 | hsa_2564 | D00370 | 0 | hsa_2559 | D08138 | 0 | hsa_6340 | D00533 |
| 1 | hsa_2564 | D00376 | 0 | hsa_2559 | D02364 | 0 | hsa_6340 | D07979 |
| 1 | hsa_2564 | D00387 | 0 | hsa_2559 | D04048 | 0 | hsa_6340 | D08127 |
| 1 | hsa_2564 | D00430 | 0 | hsa_2559 | D02283 | 0 | hsa_6340 | D07185 |
| 1 | hsa_2564 | D00457 | 0 | hsa_2559 | D02969 | 0 | hsa_6340 | D00550 |
| 1 | hsa_2564 | D00470 | 0 | hsa_2559 | D02910 | 0 | hsa_6557 | D08356 |
| 1 | hsa_2564 | D00500 | 0 | hsa_2559 | D00376 | 0 | hsa_6557 | D07999 |
| 1 | hsa_2564 | D00506 | 0 | hsa_2559 | D00557 | 0 | hsa_6557 | D08935 |
| 1 | hsa_2564 | D00531 | 0 | hsa_2559 | D09367 | 0 | hsa_6557 | D07784 |
| 1 | hsa_2564 | D00532 | 0 | hsa_2559 | D01514 | 0 | hsa_6557 | D08596 |
| 1 | hsa_2564 | D00555 | 0 | hsa_2559 | D01326 | 0 | hsa_6557 | D02624 |
| 1 | hsa_2564 | D00557 | 0 | hsa_2559 | D08001 | 0 | hsa_6557 | D09215 |
| 1 | hsa_2564 | D00693 | 0 | hsa_2559 | D01292 | 0 | hsa_6557 | D05453 |
| 1 | hsa_2564 | D00694 | 0 | hsa_2559 | D00430 | 0 | hsa_6557 | D00512 |
| 1 | hsa_2564 | D00695 | 0 | hsa_2559 | D08282 | 0 | hsa_6557 | D08275 |
| 1 | hsa_2564 | D00696 | 0 | hsa_2559 | D01293 | 0 | hsa_6557 | D06392 |
| 1 | hsa_2564 | D00697 | 0 | hsa_2559 | D07979 | 0 | hsa_6557 | D07678 |
| 1 | hsa_2564 | D00700 | 0 | hsa_2559 | D00760 | 0 | hsa_6557 | D00370 |
| 1 | hsa_2564 | D00701 | 0 | hsa_2559 | D02220 | 0 | hsa_6557 | D00557 |
| 1 | hsa_2564 | D00706 | 0 | hsa_2559 | D05077 | 0 | hsa_6557 | D01145 |
| 1 | hsa_2564 | D00713 | 0 | hsa_2559 | D05028 | 0 | hsa_6557 | D00700 |
| 1 | hsa_2564 | D00714 | 0 | hsa_2559 | D03155 | 0 | hsa_6557 | D00267 |
| 1 | hsa_2564 | D01071 | 0 | hsa_2559 | D08217 | 0 | hsa_6557 | D09757 |
| 1 | hsa_2564 | D01230 | 0 | hsa_2559 | D08009 | 0 | hsa_6557 | D00331 |
| 1 | hsa_2564 | D01245 | 0 | hsa_2559 | D02347 | 0 | hsa_6557 | D02202 |
| 1 | hsa_2564 | D01253 | 0 | hsa_2559 | D00551 | 0 | hsa_6557 | D00612 |
| 1 | hsa_2564 | D01254 | 0 | hsa_2559 | D08690 | 0 | hsa_6557 | D07595 |
| 1 | hsa_2564 | D01268 | 0 | hsa_2559 | D01286 | 0 | hsa_6557 | D00311 |
| 1 | hsa_2564 | D01278 | 0 | hsa_2559 | D00555 | 0 | hsa_6557 | D07058 |
| 1 | hsa_2564 | D01279 | 0 | hsa_2559 | D00387 | 0 | hsa_6557 | D07409 |
| 1 | hsa_2564 | D01286 | 0 | hsa_2560 | D04728 | 0 | hsa_6557 | D01328 |
| 1 | hsa_2564 | D01292 | 0 | hsa_2560 | D02103 | 0 | hsa_6557 | D01354 |
| 1 | hsa_2564 | D01293 | 0 | hsa_2560 | D00555 | 0 | hsa_6557 | D00365 |
| 1 | hsa_2564 | D01310 | 0 | hsa_2560 | D01243 | 0 | hsa_6557 | D07894 |
| 1 | hsa_2564 | D01316 | 0 | hsa_2560 | D08100 | 0 | hsa_6557 | D00011 |
| 1 | hsa_2564 | D01328 | 0 | hsa_2560 | D01104 | 0 | hsa_6557 | D00370 |
| 1 | hsa_2564 | D01354 | 0 | hsa_2560 | D08935 | 0 | hsa_6557 | D02624 |
| 1 | hsa_2564 | D01372 | 0 | hsa_2560 | D00250 | 0 | hsa_6557 | D02594 |
| 1 | hsa_2564 | D01408 | 0 | hsa_2560 | D02103 | 0 | hsa_6557 | D04048 |
| 1 | hsa_2564 | D01514 | 0 | hsa_2560 | D07509 | 0 | hsa_6557 | D00506 |
| 1 | hsa_2564 | D01564 | 0 | hsa_2560 | D01408 | 0 | hsa_6557 | D01104 |
| 1 | hsa_2564 | D01593 | 0 | hsa_2560 | D00533 | 0 | hsa_6557 | D00399 |
| 1 | hsa_2564 | D01657 | 0 | hsa_2560 | D08111 | 0 | hsa_6557 | D07784 |
| 1 | hsa_2564 | D01740 | 0 | hsa_2560 | D03679 | 0 | hsa_6557 | D04657 |
| 1 | hsa_2564 | D01744 | 0 | hsa_2560 | D01758 | 0 | hsa_6557 | D03732 |
| 1 | hsa_2564 | D01758 | 0 | hsa_2560 | D10626 | 0 | hsa_6557 | D01657 |
| 1 | hsa_2564 | D02252 | 0 | hsa_2560 | D08048 | 0 | hsa_6557 | D04728 |
| 1 | hsa_2564 | D02253 | 0 | hsa_2560 | D01268 | 0 | hsa_6557 | D04226 |
| 1 | hsa_2564 | D02283 | 0 | hsa_2560 | D07978 | 0 | hsa_6557 | D01856 |
| 1 | hsa_2564 | D02594 | 0 | hsa_2560 | D00365 | 0 | hsa_6557 | D04905 |
| 1 | hsa_2564 | D02616 | 0 | hsa_2560 | D00848 | 0 | hsa_6557 | D07509 |
| 1 | hsa_2564 | D02617 | 0 | hsa_2560 | D00457 | 0 | hsa_6833 | D04308 |
| 1 | hsa_2564 | D02624 | 0 | hsa_2560 | D04955 | 0 | hsa_6833 | D02385 |
| 1 | hsa_2564 | D03155 | 0 | hsa_2560 | D00293 | 0 | hsa_6833 | D04905 |
| 1 | hsa_2564 | D03562 | 0 | hsa_2560 | D07442 | 0 | hsa_6833 | D00733 |
| 1 | hsa_2564 | D03737 | 0 | hsa_2560 | D01856 | 0 | hsa_6833 | D08394 |
| 1 | hsa_2564 | D04257 | 0 | hsa_2560 | D08935 | 0 | hsa_6833 | D08655 |
| 1 | hsa_2564 | D04282 | 0 | hsa_2560 | D06392 | 0 | hsa_6833 | D08155 |
| 1 | hsa_2564 | D04300 | 0 | hsa_2560 | D07978 | 0 | hsa_6833 | D08964 |
| 1 | hsa_2564 | D04721 | 0 | hsa_2560 | D00293 | 0 | hsa_6833 | D00293 |
| 1 | hsa_2564 | D04882 | 0 | hsa_2560 | D09789 | 0 | hsa_6833 | D01243 |
| 1 | hsa_2564 | D05028 | 0 | hsa_2560 | D02973 | 0 | hsa_6833 | D04303 |
| 1 | hsa_2564 | D06106 | 0 | hsa_2560 | D00370 | 0 | hsa_6833 | D08458 |
| 1 | hsa_2564 | D07326 | 0 | hsa_2560 | D08987 | 0 | hsa_6833 | D01145 |
| 1 | hsa_2564 | D07409 | 0 | hsa_2560 | D00700 | 0 | hsa_6833 | D02973 |
| 1 | hsa_2564 | D07784 | 0 | hsa_2560 | D01408 | 0 | hsa_6833 | D03746 |
| 1 | hsa_2564 | D08145 | 0 | hsa_2560 | D07058 | 0 | hsa_6833 | D08490 |
| 1 | hsa_2564 | D08283 | 0 | hsa_2560 | D07845 | 0 | hsa_6833 | D08892 |
| 1 | hsa_2564 | D08356 | 0 | hsa_2560 | D10693 | 0 | hsa_6833 | D00710 |
| 1 | hsa_2564 | D08481 | 0 | hsa_2560 | D02103 | 0 | hsa_6833 | D07962 |
| 1 | hsa_2564 | D08507 | 0 | hsa_2560 | D00329 | 0 | hsa_6833 | D08669 |
| 1 | hsa_2564 | D08690 | 0 | hsa_2560 | D10112 | 0 | hsa_6833 | D07979 |
| 1 | hsa_2564 | D08840 | 0 | hsa_2560 | D08111 | 0 | hsa_6833 | D08060 |
| 1 | hsa_2564 | D10194 | 0 | hsa_2560 | D00331 | 0 | hsa_6833 | D09382 |
| 1 | hsa_2565 | D00225 | 0 | hsa_2560 | D01564 | 0 | hsa_6833 | D07962 |
| 1 | hsa_2565 | D00267 | 0 | hsa_2560 | D01740 | 0 | hsa_6833 | D08116 |
| 1 | hsa_2565 | D00280 | 0 | hsa_2560 | D00701 | 0 | hsa_6833 | D00199 |
| 1 | hsa_2565 | D00293 | 0 | hsa_2560 | D01593 | 0 | hsa_6833 | D01856 |
| 1 | hsa_2565 | D00311 | 0 | hsa_2560 | D09368 | 0 | hsa_6833 | D07678 |
| 1 | hsa_2565 | D00329 | 0 | hsa_2560 | D01310 | 0 | hsa_7442 | D01408 |
| 1 | hsa_2565 | D00365 | 0 | hsa_2560 | D04303 | 0 | hsa_7442 | D00386 |
| 1 | hsa_2565 | D00370 | 0 | hsa_2561 | D00637 | 0 | hsa_7442 | D08655 |
| 1 | hsa_2565 | D00376 | 0 | hsa_2561 | D00765 | 0 | hsa_7442 | D08394 |
| 1 | hsa_2565 | D00387 | 0 | hsa_2561 | D08048 | 0 | hsa_7442 | D08215 |
| 1 | hsa_2565 | D00430 | 0 | hsa_2561 | D01254 | 0 | hsa_7442 | D02252 |
| 1 | hsa_2565 | D00457 | 0 | hsa_2561 | D07971 | 0 | hsa_7442 | D00380 |
| 1 | hsa_2565 | D00470 | 0 | hsa_2561 | D05453 | 0 | hsa_7442 | D00365 |
| 1 | hsa_2565 | D00499 | 0 | hsa_2561 | D06146 | 0 | hsa_7442 | D08283 |
| 1 | hsa_2565 | D00500 | 0 | hsa_2561 | D08935 | 0 | hsa_7442 | D09367 |
| 1 | hsa_2565 | D00506 | 0 | hsa_2561 | D01310 | 0 | hsa_7442 | D00457 |
| 1 | hsa_2565 | D00531 | 0 | hsa_2561 | D00760 | 0 | hsa_7442 | D04728 |
| 1 | hsa_2565 | D00532 | 0 | hsa_2561 | D01810 | 0 | hsa_7442 | D08100 |
| 1 | hsa_2565 | D00549 | 0 | hsa_2561 | D07962 | 0 | hsa_7442 | D08009 |
| 1 | hsa_2565 | D00550 | 0 | hsa_2561 | D00808 | 0 | hsa_7442 | D08174 |
| 1 | hsa_2565 | D00555 | 0 | hsa_2561 | D00387 | 0 | hsa_7442 | D00199 |
| 1 | hsa_2565 | D00557 | 0 | hsa_2561 | D03914 | 0 | hsa_7442 | D01173 |
| 1 | hsa_2565 | D00693 | 0 | hsa_2561 | D07552 | 0 | hsa_7442 | D01514 |
| 1 | hsa_2565 | D00694 | 0 | hsa_2561 | D01758 | 0 | hsa_7442 | D02410 |
| 1 | hsa_2565 | D00695 | 0 | hsa_2561 | D08596 | 0 | hsa_7442 | D00499 |
| 1 | hsa_2565 | D00696 | 0 | hsa_2561 | D00531 | 0 | hsa_7442 | D00294 |
| 1 | hsa_2565 | D00697 | 0 | hsa_2561 | D02910 | 0 | hsa_7442 | D09789 |
| 1 | hsa_2565 | D00700 | 0 | hsa_2561 | D09917 | 0 | hsa_7442 | D08667 |
| 1 | hsa_2565 | D00701 | 0 | hsa_2561 | D00714 | 0 | hsa_7442 | D08422 |
| 1 | hsa_2565 | D00706 | 0 | hsa_2561 | D08596 | 0 | hsa_7442 | D06388 |
| 1 | hsa_2565 | D00713 | 0 | hsa_2561 | D08001 | 0 | hsa_7442 | D02617 |
| 1 | hsa_2565 | D00714 | 0 | hsa_2561 | D09215 | 0 | hsa_7442 | D09757 |
| 1 | hsa_2565 | D01071 | 0 | hsa_2561 | D01316 | 0 | hsa_7442 | D08111 |
| 1 | hsa_2565 | D01230 | 0 | hsa_2561 | D07979 | 0 | hsa_7442 | D07552 |
| 1 | hsa_2565 | D01245 | 0 | hsa_2561 | D06656 | 0 | hsa_7442 | D00329 |
| 1 | hsa_2565 | D01253 | 0 | hsa_2561 | D02252 | 0 | hsa_7442 | D07272 |
| 1 | hsa_2565 | D01254 | 0 | hsa_2561 | D01326 | 0 | hsa_7442 | D07447 |
| 1 | hsa_2565 | D01268 | 0 | hsa_2561 | D01328 | 0 | hsa_7442 | D04131 |
| 1 | hsa_2565 | D01278 | 0 | hsa_2561 | D00701 | 0 | hsa_7442 | D04741 |
| 1 | hsa_2565 | D01279 | 0 | hsa_2561 | D02616 | 0 | hsa_7442 | D10112 |
| 1 | hsa_2565 | D01286 | 0 | hsa_2561 | D00252 | 0 | hsa_7442 | D00500 |
| 1 | hsa_2565 | D01292 | 0 | hsa_2561 | D02385 | 0 | hsa_7442 | D01740 |
| 1 | hsa_2565 | D01293 | 0 | hsa_2561 | D00766 | 0 | hsa_7442 | D07442 |
| 1 | hsa_2565 | D01310 | 0 | hsa_2561 | D00764 | 0 | hsa_7442 | D02969 |
| 1 | hsa_2565 | D01316 | 0 | hsa_2561 | D00555 | 0 | hsa_7442 | D02385 |
| 1 | hsa_2565 | D01328 | 0 | hsa_2561 | D00701 | 0 | hsa_7442 | D01593 |
| 1 | hsa_2565 | D01354 | 0 | hsa_2561 | D00700 | 0 | hsa_7442 | D07962 |
| 1 | hsa_2565 | D01372 | 0 | hsa_2561 | D09612 | 0 | hsa_7442 | D01856 |
| 1 | hsa_2565 | D01408 | 0 | hsa_2561 | D01604 | 0 | hsa_7442 | D01104 |
| 1 | hsa_2565 | D01514 | 0 | hsa_2561 | D04905 | 0 | hsa_7442 | D08270 |
| 1 | hsa_2565 | D01564 | 0 | hsa_2561 | D01278 | 0 | hsa_7442 | D07978 |
| 1 | hsa_2565 | D01593 | 0 | hsa_2561 | D09035 | 0 | hsa_7442 | D01564 |
| 1 | hsa_2565 | D01657 | 0 | hsa_2561 | D04303 | 0 | hsa_7442 | D01268 |
| 1 | hsa_2565 | D01740 | 0 | hsa_2562 | D02973 | 0 | hsa_7442 | D01245 |
| 1 | hsa_2565 | D01744 | 0 | hsa_2562 | D00311 | 0 | hsa_7442 | D08669 |
| 1 | hsa_2565 | D01758 | 0 | hsa_2562 | D02973 | 0 | hsa_7442 | D04257 |
| 1 | hsa_2565 | D02252 | 0 | hsa_2562 | D08655 | 0 | hsa_7442 | D00303 |
| 1 | hsa_2565 | D02253 | 0 | hsa_2562 | D03155 | 0 | hsa_775 | D00693 |
| 1 | hsa_2565 | D02283 | 0 | hsa_2562 | D08270 | 0 | hsa_775 | D01254 |
| 1 | hsa_2565 | D02594 | 0 | hsa_2562 | D01286 | 0 | hsa_775 | D05028 |
| 1 | hsa_2565 | D02616 | 0 | hsa_2562 | D01254 | 0 | hsa_775 | D07450 |
| 1 | hsa_2565 | D02617 | 0 | hsa_2562 | D07978 | 0 | hsa_775 | D02969 |
| 1 | hsa_2565 | D02624 | 0 | hsa_2562 | D00531 | 0 | hsa_775 | D08596 |
| 1 | hsa_2565 | D03155 | 0 | hsa_2562 | D00252 | 0 | hsa_775 | D01071 |
| 1 | hsa_2565 | D03562 | 0 | hsa_2562 | D10111 | 0 | hsa_775 | D05028 |
| 1 | hsa_2565 | D03737 | 0 | hsa_2562 | D00225 | 0 | hsa_775 | D07993 |
| 1 | hsa_2565 | D04257 | 0 | hsa_2562 | D00225 | 0 | hsa_775 | D00636 |
| 1 | hsa_2565 | D04282 | 0 | hsa_2562 | D06392 | 0 | hsa_775 | D01071 |
| 1 | hsa_2565 | D04300 | 0 | hsa_2562 | D03100 | 0 | hsa_775 | D04657 |
| 1 | hsa_2565 | D04721 | 0 | hsa_2562 | D08987 | 0 | hsa_775 | D00386 |
| 1 | hsa_2565 | D04882 | 0 | hsa_2562 | D10613 | 0 | hsa_775 | D06204 |
| 1 | hsa_2565 | D05028 | 0 | hsa_2562 | D00552 | 0 | hsa_775 | D02969 |
| 1 | hsa_2565 | D06106 | 0 | hsa_2562 | D00532 | 0 | hsa_775 | D01279 |
| 1 | hsa_2565 | D07326 | 0 | hsa_2562 | D00499 | 0 | hsa_775 | D03732 |
| 1 | hsa_2565 | D07409 | 0 | hsa_2562 | D00250 | 0 | hsa_775 | D08217 |
| 1 | hsa_2565 | D07784 | 0 | hsa_2562 | D00533 | 0 | hsa_775 | D00700 |
| 1 | hsa_2565 | D08145 | 0 | hsa_2562 | D07520 | 0 | hsa_775 | D00549 |
| 1 | hsa_2565 | D08283 | 0 | hsa_2562 | D07520 | 0 | hsa_775 | D01604 |
| 1 | hsa_2565 | D08356 | 0 | hsa_2562 | D08098 | 0 | hsa_775 | D00370 |
| 1 | hsa_2565 | D08481 | 0 | hsa_2562 | D08001 | 0 | hsa_775 | D10111 |
| 1 | hsa_2565 | D08507 | 0 | hsa_2562 | D01354 | 0 | hsa_775 | D07978 |
| 1 | hsa_2565 | D08690 | 0 | hsa_2562 | D01479 | 0 | hsa_775 | D04849 |
| 1 | hsa_2565 | D08840 | 0 | hsa_2562 | D10370 | 0 | hsa_775 | D06388 |
| 1 | hsa_2565 | D10194 | 0 | hsa_2562 | D08009 | 0 | hsa_775 | D04226 |
| 1 | hsa_2566 | D00267 | 0 | hsa_2562 | D00418 | 0 | hsa_775 | D02910 |
| 1 | hsa_2566 | D00280 | 0 | hsa_2562 | D10702 | 0 | hsa_775 | D08155 |
| 1 | hsa_2566 | D00311 | 0 | hsa_2562 | D02780 | 0 | hsa_775 | D03492 |
| 1 | hsa_2566 | D00365 | 0 | hsa_2562 | D06388 | 0 | hsa_775 | D00848 |
| 1 | hsa_2566 | D00370 | 0 | hsa_2562 | D05028 | 0 | hsa_775 | D07447 |
| 1 | hsa_2566 | D00376 | 0 | hsa_2562 | D07979 | 0 | hsa_775 | D04905 |
| 1 | hsa_2566 | D00387 | 0 | hsa_2562 | D02696 | 0 | hsa_775 | D00631 |
| 1 | hsa_2566 | D00430 | 0 | hsa_2562 | D08394 | 0 | hsa_775 | D00701 |
| 1 | hsa_2566 | D00457 | 0 | hsa_2562 | D01562 | 0 | hsa_775 | D06653 |
| 1 | hsa_2566 | D00470 | 0 | hsa_2563 | D08655 | 0 | hsa_775 | D02969 |
| 1 | hsa_2566 | D00500 | 0 | hsa_2563 | D00336 | 0 | hsa_775 | D00553 |
| 1 | hsa_2566 | D00506 | 0 | hsa_2563 | D00375 | 0 | hsa_775 | D04282 |
| 1 | hsa_2566 | D00531 | 0 | hsa_2563 | D07326 | 0 | hsa_775 | D10626 |
| 1 | hsa_2566 | D00532 | 0 | hsa_2563 | D06172 | 0 | hsa_775 | D00636 |
| 1 | hsa_2566 | D00555 | 0 | hsa_2563 | D09368 | 0 | hsa_775 | D07520 |
| 1 | hsa_2566 | D00557 | 0 | hsa_2563 | D02096 | 0 | hsa_775 | D04048 |
| 1 | hsa_2566 | D00693 | 0 | hsa_2563 | D08987 | 0 | hsa_775 | D02283 |
| 1 | hsa_2566 | D00694 | 0 | hsa_2563 | D01657 | 0 | hsa_775 | D05482 |
| 1 | hsa_2566 | D00695 | 0 | hsa_2563 | D01310 | 0 | hsa_775 | D08667 |
| 1 | hsa_2566 | D00696 | 0 | hsa_2563 | D05453 | 0 | hsa_775 | D02347 |
| 1 | hsa_2566 | D00697 | 0 | hsa_2563 | D10626 | 0 | hsa_775 | D02716 |
| 1 | hsa_2566 | D00700 | 0 | hsa_2563 | D00303 | 0 | hsa_775 | D10370 |
| 1 | hsa_2566 | D00701 | 0 | hsa_2563 | D01479 | 0 | hsa_775 | D01554 |
| 1 | hsa_2566 | D00706 | 0 | hsa_2563 | D10693 | 0 | hsa_775 | D05703 |
| 1 | hsa_2566 | D00713 | 0 | hsa_2563 | D00293 | 0 | hsa_775 | D01593 |
| 1 | hsa_2566 | D00714 | 0 | hsa_2563 | D09918 | 0 | hsa_775 | D00706 |
| 1 | hsa_2566 | D01071 | 0 | hsa_2563 | D07845 | 0 | hsa_775 | D00331 |
| 1 | hsa_2566 | D01230 | 0 | hsa_2563 | D08507 | 0 | hsa_775 | D04741 |
| 1 | hsa_2566 | D01245 | 0 | hsa_2563 | D02385 | 0 | hsa_776 | D01785 |
| 1 | hsa_2566 | D01253 | 0 | hsa_2563 | D00386 | 0 | hsa_776 | D05453 |
| 1 | hsa_2566 | D01254 | 0 | hsa_2563 | D00695 | 0 | hsa_776 | D01604 |
| 1 | hsa_2566 | D01268 | 0 | hsa_2563 | D04048 | 0 | hsa_776 | D00758 |
| 1 | hsa_2566 | D01278 | 0 | hsa_2563 | D00808 | 0 | hsa_776 | D01316 |
| 1 | hsa_2566 | D01279 | 0 | hsa_2563 | D01071 | 0 | hsa_776 | D00418 |
| 1 | hsa_2566 | D01286 | 0 | hsa_2563 | D08275 | 0 | hsa_776 | D05482 |
| 1 | hsa_2566 | D01292 | 0 | hsa_2563 | D01740 | 0 | hsa_776 | D00532 |
| 1 | hsa_2566 | D01293 | 0 | hsa_2563 | D00767 | 0 | hsa_776 | D05442 |
| 1 | hsa_2566 | D01310 | 0 | hsa_2563 | D00252 | 0 | hsa_776 | D01593 |
| 1 | hsa_2566 | D01316 | 0 | hsa_2563 | D07979 | 0 | hsa_776 | D05028 |
| 1 | hsa_2566 | D01328 | 0 | hsa_2563 | D01326 | 0 | hsa_776 | D02780 |
| 1 | hsa_2566 | D01354 | 0 | hsa_2563 | D09382 | 0 | hsa_776 | D02780 |
| 1 | hsa_2566 | D01372 | 0 | hsa_2563 | D08270 | 0 | hsa_776 | D08596 |
| 1 | hsa_2566 | D01408 | 0 | hsa_2563 | D07409 | 0 | hsa_776 | D06388 |
| 1 | hsa_2566 | D01514 | 0 | hsa_2563 | D09612 | 0 | hsa_776 | D02275 |
| 1 | hsa_2566 | D01564 | 0 | hsa_2563 | D00555 | 0 | hsa_776 | D00763 |
| 1 | hsa_2566 | D01593 | 0 | hsa_2563 | D08282 | 0 | hsa_776 | D00252 |
| 1 | hsa_2566 | D01657 | 0 | hsa_2563 | D07784 | 0 | hsa_776 | D01254 |
| 1 | hsa_2566 | D01740 | 0 | hsa_2563 | D04741 | 0 | hsa_776 | D04721 |
| 1 | hsa_2566 | D01744 | 0 | hsa_2563 | D06172 | 0 | hsa_776 | D00636 |
| 1 | hsa_2566 | D01758 | 0 | hsa_2563 | D00011 | 0 | hsa_776 | D03562 |
| 1 | hsa_2566 | D02252 | 0 | hsa_2563 | D02283 | 0 | hsa_776 | D05703 |
| 1 | hsa_2566 | D02253 | 0 | hsa_2563 | D06204 | 0 | hsa_776 | D02594 |
| 1 | hsa_2566 | D02283 | 0 | hsa_2563 | D01268 | 0 | hsa_776 | D07552 |
| 1 | hsa_2566 | D02594 | 0 | hsa_2563 | D08283 | 0 | hsa_776 | D01303 |
| 1 | hsa_2566 | D02616 | 0 | hsa_2563 | D00387 | 0 | hsa_776 | D01514 |
| 1 | hsa_2566 | D02617 | 0 | hsa_2563 | D07979 | 0 | hsa_776 | D00637 |
| 1 | hsa_2566 | D02624 | 0 | hsa_2563 | D03492 | 0 | hsa_776 | D02103 |
| 1 | hsa_2566 | D03155 | 0 | hsa_2563 | D07326 | 0 | hsa_776 | D07185 |
| 1 | hsa_2566 | D03562 | 0 | hsa_2563 | D00710 | 0 | hsa_776 | D00706 |
| 1 | hsa_2566 | D03737 | 0 | hsa_2564 | D01372 | 0 | hsa_776 | D00760 |
| 1 | hsa_2566 | D04257 | 0 | hsa_2564 | D08155 | 0 | hsa_776 | D04905 |
| 1 | hsa_2566 | D04282 | 0 | hsa_2564 | D02274 | 0 | hsa_776 | D10627 |
| 1 | hsa_2566 | D04300 | 0 | hsa_2564 | D00380 | 0 | hsa_776 | D02910 |
| 1 | hsa_2566 | D04721 | 0 | hsa_2564 | D05714 | 0 | hsa_776 | D02103 |
| 1 | hsa_2566 | D04882 | 0 | hsa_2564 | D02973 | 0 | hsa_776 | D07509 |
| 1 | hsa_2566 | D05028 | 0 | hsa_2564 | D01744 | 0 | hsa_776 | D02385 |
| 1 | hsa_2566 | D06106 | 0 | hsa_2564 | D00549 | 0 | hsa_776 | D01810 |
| 1 | hsa_2566 | D07326 | 0 | hsa_2564 | D09757 | 0 | hsa_776 | D02716 |
| 1 | hsa_2566 | D07409 | 0 | hsa_2564 | D05482 | 0 | hsa_776 | D06392 |
| 1 | hsa_2566 | D07784 | 0 | hsa_2564 | D00636 | 0 | hsa_776 | D02275 |
| 1 | hsa_2566 | D08145 | 0 | hsa_2564 | D01104 | 0 | hsa_776 | D00759 |
| 1 | hsa_2566 | D08283 | 0 | hsa_2564 | D05077 | 0 | hsa_776 | D00553 |
| 1 | hsa_2566 | D08356 | 0 | hsa_2564 | D07552 | 0 | hsa_776 | D08964 |
| 1 | hsa_2566 | D08481 | 0 | hsa_2564 | D08422 | 0 | hsa_776 | D01145 |
| 1 | hsa_2566 | D08507 | 0 | hsa_2564 | D04303 | 0 | hsa_776 | D00304 |
| 1 | hsa_2566 | D08690 | 0 | hsa_2564 | D00701 | 0 | hsa_776 | D01657 |
| 1 | hsa_2566 | D08840 | 0 | hsa_2564 | D09215 | 0 | hsa_776 | D04303 |
| 1 | hsa_2566 | D10194 | 0 | hsa_2564 | D00710 | 0 | hsa_776 | D01230 |
| 1 | hsa_2567 | D00267 | 0 | hsa_2564 | D07886 | 0 | hsa_776 | D02292 |
| 1 | hsa_2567 | D00280 | 0 | hsa_2564 | D00764 | 0 | hsa_776 | D02716 |
| 1 | hsa_2567 | D00311 | 0 | hsa_2564 | D09789 | 0 | hsa_776 | D02252 |
| 1 | hsa_2567 | D00365 | 0 | hsa_2564 | D00532 | 0 | hsa_776 | D08282 |
| 1 | hsa_2567 | D00370 | 0 | hsa_2564 | D06392 | 0 | hsa_776 | D01856 |
| 1 | hsa_2567 | D00376 | 0 | hsa_2564 | D02347 | 0 | hsa_776 | D07894 |
| 1 | hsa_2567 | D00387 | 0 | hsa_2564 | D00399 | 0 | hsa_776 | D00764 |
| 1 | hsa_2567 | D00430 | 0 | hsa_2564 | D02410 | 0 | hsa_778 | D05482 |
| 1 | hsa_2567 | D00457 | 0 | hsa_2564 | D08458 | 0 | hsa_778 | D00531 |
| 1 | hsa_2567 | D00470 | 0 | hsa_2564 | D00696 | 0 | hsa_778 | D00808 |
| 1 | hsa_2567 | D00500 | 0 | hsa_2564 | D08174 | 0 | hsa_778 | D00612 |
| 1 | hsa_2567 | D00506 | 0 | hsa_2564 | D08987 | 0 | hsa_778 | D02220 |
| 1 | hsa_2567 | D00531 | 0 | hsa_2564 | D08490 | 0 | hsa_778 | D10626 |
| 1 | hsa_2567 | D00532 | 0 | hsa_2564 | D00380 | 0 | hsa_778 | D05447 |
| 1 | hsa_2567 | D00555 | 0 | hsa_2564 | D06517 | 0 | hsa_778 | D10111 |
| 1 | hsa_2567 | D00557 | 0 | hsa_2564 | D08458 | 0 | hsa_778 | D08422 |
| 1 | hsa_2567 | D00693 | 0 | hsa_2564 | D08009 | 0 | hsa_778 | D07978 |
| 1 | hsa_2567 | D00694 | 0 | hsa_2564 | D01514 | 0 | hsa_778 | D02617 |
| 1 | hsa_2567 | D00695 | 0 | hsa_2564 | D01758 | 0 | hsa_778 | D00706 |
| 1 | hsa_2567 | D00696 | 0 | hsa_2564 | D00808 | 0 | hsa_778 | D07886 |
| 1 | hsa_2567 | D00697 | 0 | hsa_2564 | D04282 | 0 | hsa_778 | D07979 |
| 1 | hsa_2567 | D00700 | 0 | hsa_2564 | D03742 | 0 | hsa_778 | D01254 |
| 1 | hsa_2567 | D00701 | 0 | hsa_2564 | D02364 | 0 | hsa_778 | D03742 |
| 1 | hsa_2567 | D00706 | 0 | hsa_2564 | D08458 | 0 | hsa_778 | D04282 |
| 1 | hsa_2567 | D00713 | 0 | hsa_2564 | D01564 | 0 | hsa_778 | D03914 |
| 1 | hsa_2567 | D00714 | 0 | hsa_2564 | D09917 | 0 | hsa_778 | D01071 |
| 1 | hsa_2567 | D01071 | 0 | hsa_2564 | D01278 | 0 | hsa_778 | D00399 |
| 1 | hsa_2567 | D01230 | 0 | hsa_2565 | D01562 | 0 | hsa_778 | D00064 |
| 1 | hsa_2567 | D01245 | 0 | hsa_2565 | D01554 | 0 | hsa_778 | D01408 |
| 1 | hsa_2567 | D01253 | 0 | hsa_2565 | D08111 | 0 | hsa_778 | D03742 |
| 1 | hsa_2567 | D01254 | 0 | hsa_2565 | D00733 | 0 | hsa_778 | D07886 |
| 1 | hsa_2567 | D01268 | 0 | hsa_2565 | D00280 | 0 | hsa_778 | D01908 |
| 1 | hsa_2567 | D01278 | 0 | hsa_2565 | D08892 | 0 | hsa_778 | D09612 |
| 1 | hsa_2567 | D01279 | 0 | hsa_2565 | D07442 | 0 | hsa_778 | D10702 |
| 1 | hsa_2567 | D01286 | 0 | hsa_2565 | D08435 | 0 | hsa_778 | D00199 |
| 1 | hsa_2567 | D01292 | 0 | hsa_2565 | D09918 | 0 | hsa_778 | D07450 |
| 1 | hsa_2567 | D01293 | 0 | hsa_2565 | D05714 | 0 | hsa_778 | D07494 |
| 1 | hsa_2567 | D01310 | 0 | hsa_2565 | D00713 | 0 | hsa_778 | D02220 |
| 1 | hsa_2567 | D01316 | 0 | hsa_2565 | D08435 | 0 | hsa_778 | D05453 |
| 1 | hsa_2567 | D01328 | 0 | hsa_2565 | D00250 | 0 | hsa_778 | D06282 |
| 1 | hsa_2567 | D01354 | 0 | hsa_2565 | D08275 | 0 | hsa_778 | D09539 |
| 1 | hsa_2567 | D01372 | 0 | hsa_2565 | D00700 | 0 | hsa_778 | D00766 |
| 1 | hsa_2567 | D01408 | 0 | hsa_2565 | D00387 | 0 | hsa_778 | D04849 |
| 1 | hsa_2567 | D01514 | 0 | hsa_2565 | D00700 | 0 | hsa_778 | D07971 |
| 1 | hsa_2567 | D01564 | 0 | hsa_2565 | D09612 | 0 | hsa_778 | D00759 |
| 1 | hsa_2567 | D01593 | 0 | hsa_2565 | D04905 | 0 | hsa_778 | D05145 |
| 1 | hsa_2567 | D01657 | 0 | hsa_2565 | D00506 | 0 | hsa_778 | D06146 |
| 1 | hsa_2567 | D01740 | 0 | hsa_2565 | D08060 | 0 | hsa_778 | D02780 |
| 1 | hsa_2567 | D01744 | 0 | hsa_2565 | D07978 | 0 | hsa_778 | D09368 |
| 1 | hsa_2567 | D01758 | 0 | hsa_2565 | D07442 | 0 | hsa_778 | D01758 |
| 1 | hsa_2567 | D02252 | 0 | hsa_2565 | D07978 | 0 | hsa_778 | D02969 |
| 1 | hsa_2567 | D02253 | 0 | hsa_2565 | D00532 | 0 | hsa_778 | D00808 |
| 1 | hsa_2567 | D02283 | 0 | hsa_2565 | D00011 | 0 | hsa_778 | D00304 |
| 1 | hsa_2567 | D02594 | 0 | hsa_2565 | D01514 | 0 | hsa_778 | D01553 |
| 1 | hsa_2567 | D02616 | 0 | hsa_2565 | D05453 | 0 | hsa_778 | D00636 |
| 1 | hsa_2567 | D02617 | 0 | hsa_2565 | D02624 | 0 | hsa_778 | D08100 |
| 1 | hsa_2567 | D02624 | 0 | hsa_2565 | D01554 | 0 | hsa_778 | D01278 |
| 1 | hsa_2567 | D03155 | 0 | hsa_2565 | D07494 | 0 | hsa_779 | D08098 |
| 1 | hsa_2567 | D03562 | 0 | hsa_2565 | D08421 | 0 | hsa_779 | D08098 |
| 1 | hsa_2567 | D03737 | 0 | hsa_2565 | D07552 | 0 | hsa_779 | D00693 |
| 1 | hsa_2567 | D04257 | 0 | hsa_2565 | D02364 | 0 | hsa_779 | D01455 |
| 1 | hsa_2567 | D04282 | 0 | hsa_2565 | D00331 | 0 | hsa_779 | D02969 |
| 1 | hsa_2567 | D04300 | 0 | hsa_2565 | D09789 | 0 | hsa_779 | D05077 |
| 1 | hsa_2567 | D04721 | 0 | hsa_2565 | D00499 | 0 | hsa_779 | D10626 |
| 1 | hsa_2567 | D04882 | 0 | hsa_2565 | D09035 | 0 | hsa_779 | D07881 |
| 1 | hsa_2567 | D05028 | 0 | hsa_2565 | D03914 | 0 | hsa_779 | D00549 |
| 1 | hsa_2567 | D06106 | 0 | hsa_2565 | D08111 | 0 | hsa_779 | D00763 |
| 1 | hsa_2567 | D07326 | 0 | hsa_2565 | D01455 | 0 | hsa_779 | D08377 |
| 1 | hsa_2567 | D07409 | 0 | hsa_2565 | D02969 | 0 | hsa_779 | D03914 |
| 1 | hsa_2567 | D07784 | 0 | hsa_2566 | D03562 | 0 | hsa_779 | D08377 |
| 1 | hsa_2567 | D08145 | 0 | hsa_2566 | D06392 | 0 | hsa_779 | D08394 |
| 1 | hsa_2567 | D08283 | 0 | hsa_2566 | D00552 | 0 | hsa_779 | D02385 |
| 1 | hsa_2567 | D08356 | 0 | hsa_2566 | D04282 | 0 | hsa_779 | D01593 |
| 1 | hsa_2567 | D08481 | 0 | hsa_2566 | D00250 | 0 | hsa_779 | D07962 |
| 1 | hsa_2567 | D08507 | 0 | hsa_2566 | D00765 | 0 | hsa_779 | D00760 |
| 1 | hsa_2567 | D08690 | 0 | hsa_2566 | D01279 | 0 | hsa_779 | D01758 |
| 1 | hsa_2567 | D08840 | 0 | hsa_2566 | D04308 | 0 | hsa_779 | D02969 |
| 1 | hsa_2567 | D10194 | 0 | hsa_2566 | D00848 | 0 | hsa_779 | D10470 |
| 1 | hsa_2568 | D00225 | 0 | hsa_2566 | D07971 | 0 | hsa_779 | D00011 |
| 1 | hsa_2568 | D00267 | 0 | hsa_2566 | D00457 | 0 | hsa_779 | D08840 |
| 1 | hsa_2568 | D00280 | 0 | hsa_2566 | D09569 | 0 | hsa_779 | D08421 |
| 1 | hsa_2568 | D00293 | 0 | hsa_2566 | D01564 | 0 | hsa_779 | D08145 |
| 1 | hsa_2568 | D00311 | 0 | hsa_2566 | D00637 | 0 | hsa_779 | D03562 |
| 1 | hsa_2568 | D00329 | 0 | hsa_2566 | D07886 | 0 | hsa_779 | D00710 |
| 1 | hsa_2568 | D00365 | 0 | hsa_2566 | D00225 | 0 | hsa_779 | D07509 |
| 1 | hsa_2568 | D00370 | 0 | hsa_2566 | D00531 | 0 | hsa_779 | D08145 |
| 1 | hsa_2568 | D00376 | 0 | hsa_2566 | D09917 | 0 | hsa_779 | D04741 |
| 1 | hsa_2568 | D00387 | 0 | hsa_2566 | D08174 | 0 | hsa_779 | D00553 |
| 1 | hsa_2568 | D00430 | 0 | hsa_2566 | D01554 | 0 | hsa_779 | D00386 |
| 1 | hsa_2568 | D00457 | 0 | hsa_2566 | D07409 | 0 | hsa_779 | D07881 |
| 1 | hsa_2568 | D00470 | 0 | hsa_2566 | D07058 | 0 | hsa_779 | D01254 |
| 1 | hsa_2568 | D00499 | 0 | hsa_2566 | D07595 | 0 | hsa_779 | D00064 |
| 1 | hsa_2568 | D00500 | 0 | hsa_2566 | D02616 | 0 | hsa_779 | D02274 |
| 1 | hsa_2568 | D00506 | 0 | hsa_2566 | D08448 | 0 | hsa_779 | D04882 |
| 1 | hsa_2568 | D00531 | 0 | hsa_2566 | D08340 | 0 | hsa_779 | D00336 |
| 1 | hsa_2568 | D00532 | 0 | hsa_2566 | D00706 | 0 | hsa_779 | D08001 |
| 1 | hsa_2568 | D00549 | 0 | hsa_2566 | D02616 | 0 | hsa_779 | D01316 |
| 1 | hsa_2568 | D00550 | 0 | hsa_2566 | D04131 | 0 | hsa_779 | D09789 |
| 1 | hsa_2568 | D00555 | 0 | hsa_2566 | D01268 | 0 | hsa_779 | D07272 |
| 1 | hsa_2568 | D00557 | 0 | hsa_2566 | D00064 | 0 | hsa_779 | D01230 |
| 1 | hsa_2568 | D00693 | 0 | hsa_2566 | D08048 | 0 | hsa_779 | D08596 |
| 1 | hsa_2568 | D00694 | 0 | hsa_2566 | D00550 | 0 | hsa_779 | D09917 |
| 1 | hsa_2568 | D00695 | 0 | hsa_2566 | D07494 | 0 | hsa_779 | D07409 |
| 1 | hsa_2568 | D00696 | 0 | hsa_2566 | D01230 | 0 | hsa_781 | D04728 |
| 1 | hsa_2568 | D00697 | 0 | hsa_2566 | D08138 | 0 | hsa_781 | D03679 |
| 1 | hsa_2568 | D00700 | 0 | hsa_2566 | D04728 | 0 | hsa_781 | D02385 |
| 1 | hsa_2568 | D00701 | 0 | hsa_2566 | D08422 | 0 | hsa_781 | D10627 |
| 1 | hsa_2568 | D00706 | 0 | hsa_2566 | D08101 | 0 | hsa_781 | D00438 |
| 1 | hsa_2568 | D00713 | 0 | hsa_2566 | D01856 | 0 | hsa_781 | D06172 |
| 1 | hsa_2568 | D00714 | 0 | hsa_2566 | D00500 | 0 | hsa_781 | D01310 |
| 1 | hsa_2568 | D01071 | 0 | hsa_2566 | D08377 | 0 | hsa_781 | D00710 |
| 1 | hsa_2568 | D01230 | 0 | hsa_2566 | D01562 | 0 | hsa_781 | D08283 |
| 1 | hsa_2568 | D01245 | 0 | hsa_2566 | D10370 | 0 | hsa_781 | D08840 |
| 1 | hsa_2568 | D01253 | 0 | hsa_2566 | D01740 | 0 | hsa_781 | D09612 |
| 1 | hsa_2568 | D01254 | 0 | hsa_2566 | D06146 | 0 | hsa_781 | D04905 |
| 1 | hsa_2568 | D01268 | 0 | hsa_2566 | D08138 | 0 | hsa_781 | D01408 |
| 1 | hsa_2568 | D01278 | 0 | hsa_2566 | D08596 | 0 | hsa_781 | D01479 |
| 1 | hsa_2568 | D01279 | 0 | hsa_2566 | D08048 | 0 | hsa_781 | D05442 |
| 1 | hsa_2568 | D01286 | 0 | hsa_2567 | D10112 | 0 | hsa_781 | D00376 |
| 1 | hsa_2568 | D01292 | 0 | hsa_2567 | D07979 | 0 | hsa_781 | D08840 |
| 1 | hsa_2568 | D01293 | 0 | hsa_2567 | D01243 | 0 | hsa_781 | D00733 |
| 1 | hsa_2568 | D01310 | 0 | hsa_2567 | D00552 | 0 | hsa_781 | D09368 |
| 1 | hsa_2568 | D01316 | 0 | hsa_2567 | D00636 | 0 | hsa_781 | D06392 |
| 1 | hsa_2568 | D01328 | 0 | hsa_2567 | D08481 | 0 | hsa_781 | D07894 |
| 1 | hsa_2568 | D01354 | 0 | hsa_2567 | D08275 | 0 | hsa_781 | D00537 |
| 1 | hsa_2568 | D01372 | 0 | hsa_2567 | D08507 | 0 | hsa_781 | D05482 |
| 1 | hsa_2568 | D01408 | 0 | hsa_2567 | D00380 | 0 | hsa_781 | D08377 |
| 1 | hsa_2568 | D01514 | 0 | hsa_2567 | D07784 | 0 | hsa_781 | D02696 |
| 1 | hsa_2568 | D01564 | 0 | hsa_2567 | D00733 | 0 | hsa_781 | D08448 |
| 1 | hsa_2568 | D01593 | 0 | hsa_2567 | D08060 | 0 | hsa_781 | D03492 |
| 1 | hsa_2568 | D01657 | 0 | hsa_2567 | D07409 | 0 | hsa_781 | D00693 |
| 1 | hsa_2568 | D01740 | 0 | hsa_2567 | D06656 | 0 | hsa_781 | D04918 |
| 1 | hsa_2568 | D01744 | 0 | hsa_2567 | D08100 | 0 | hsa_781 | D01553 |
| 1 | hsa_2568 | D01758 | 0 | hsa_2567 | D01604 | 0 | hsa_781 | D00225 |
| 1 | hsa_2568 | D02252 | 0 | hsa_2567 | D02973 | 0 | hsa_781 | D00552 |
| 1 | hsa_2568 | D02253 | 0 | hsa_2567 | D08127 | 0 | hsa_781 | D00252 |
| 1 | hsa_2568 | D02283 | 0 | hsa_2567 | D04905 | 0 | hsa_781 | D01245 |
| 1 | hsa_2568 | D02594 | 0 | hsa_2567 | D08507 | 0 | hsa_781 | D01316 |
| 1 | hsa_2568 | D02616 | 0 | hsa_2567 | D01554 | 0 | hsa_781 | D01908 |
| 1 | hsa_2568 | D02617 | 0 | hsa_2567 | D08009 | 0 | hsa_781 | D00329 |
| 1 | hsa_2568 | D02624 | 0 | hsa_2567 | D00760 | 0 | hsa_781 | D05714 |
| 1 | hsa_2568 | D03155 | 0 | hsa_2567 | D08507 | 0 | hsa_781 | D00758 |
| 1 | hsa_2568 | D03562 | 0 | hsa_2567 | D08490 | 0 | hsa_781 | D08138 |
| 1 | hsa_2568 | D03737 | 0 | hsa_2567 | D00457 | 0 | hsa_781 | D07520 |
| 1 | hsa_2568 | D04257 | 0 | hsa_2567 | D00267 | 0 | hsa_781 | D08377 |
| 1 | hsa_2568 | D04282 | 0 | hsa_2567 | D00252 | 0 | hsa_781 | D00760 |
| 1 | hsa_2568 | D04300 | 0 | hsa_2567 | D01479 | 0 | hsa_781 | D05145 |
| 1 | hsa_2568 | D04721 | 0 | hsa_2567 | D07881 | 0 | hsa_781 | D00438 |
| 1 | hsa_2568 | D04882 | 0 | hsa_2567 | D09215 | 0 | hsa_781 | D03732 |
| 1 | hsa_2568 | D05028 | 0 | hsa_2567 | D04905 | 0 | hsa_781 | D01071 |
| 1 | hsa_2568 | D06106 | 0 | hsa_2567 | D08215 | 0 | hsa_79054 | D01593 |
| 1 | hsa_2568 | D07326 | 0 | hsa_2567 | D01292 | 0 | hsa_79054 | D09367 |
| 1 | hsa_2568 | D07409 | 0 | hsa_2567 | D08138 | 0 | hsa_79054 | D08001 |
| 1 | hsa_2568 | D07784 | 0 | hsa_2567 | D01245 | 0 | hsa_79054 | D08481 |
| 1 | hsa_2568 | D08145 | 0 | hsa_2567 | D02537 | 0 | hsa_79054 | D00387 |
| 1 | hsa_2568 | D08283 | 0 | hsa_2567 | D00557 | 0 | hsa_79054 | D00533 |
| 1 | hsa_2568 | D08356 | 0 | hsa_2567 | D08098 | 0 | hsa_79054 | D00693 |
| 1 | hsa_2568 | D08481 | 0 | hsa_2568 | D08490 | 0 | hsa_79054 | D04282 |
| 1 | hsa_2568 | D08507 | 0 | hsa_2568 | D07326 | 0 | hsa_79054 | D05703 |
| 1 | hsa_2568 | D08690 | 0 | hsa_2568 | D03100 | 0 | hsa_79054 | D01849 |
| 1 | hsa_2568 | D08840 | 0 | hsa_2568 | D04257 | 0 | hsa_79054 | D02537 |
| 1 | hsa_2568 | D10194 | 0 | hsa_2568 | D06517 | 0 | hsa_79054 | D01408 |
| 1 | hsa_2741 | D00011 | 0 | hsa_2568 | D08596 | 0 | hsa_79054 | D00631 |
| 1 | hsa_2741 | D10470 | 0 | hsa_2568 | D04905 | 0 | hsa_79054 | D00418 |
| 1 | hsa_2742 | D00011 | 0 | hsa_2568 | D10613 | 0 | hsa_79054 | D04728 |
| 1 | hsa_2743 | D00011 | 0 | hsa_2568 | D01562 | 0 | hsa_79054 | D08422 |
| 1 | hsa_2890 | D00537 | 0 | hsa_2568 | D01372 | 0 | hsa_79054 | D00199 |
| 1 | hsa_2890 | D02696 | 0 | hsa_2568 | D00537 | 0 | hsa_79054 | D01230 |
| 1 | hsa_2890 | D04131 | 0 | hsa_2568 | D08356 | 0 | hsa_79054 | D01293 |
| 1 | hsa_2890 | D06656 | 0 | hsa_2568 | D08275 | 0 | hsa_79054 | D01514 |
| 1 | hsa_2890 | D08964 | 0 | hsa_2568 | D00303 | 0 | hsa_79054 | D02292 |
| 1 | hsa_2890 | D09035 | 0 | hsa_2568 | D08596 | 0 | hsa_79054 | D00763 |
| 1 | hsa_2891 | D00537 | 0 | hsa_2568 | D05703 | 0 | hsa_79054 | D06282 |
| 1 | hsa_2891 | D02696 | 0 | hsa_2568 | D02202 | 0 | hsa_79054 | D08458 |
| 1 | hsa_2891 | D04131 | 0 | hsa_2568 | D00370 | 0 | hsa_79054 | D00532 |
| 1 | hsa_2891 | D06656 | 0 | hsa_2568 | D02275 | 0 | hsa_79054 | D03562 |
| 1 | hsa_2891 | D08964 | 0 | hsa_2568 | D02347 | 0 | hsa_79054 | D01758 |
| 1 | hsa_2891 | D09035 | 0 | hsa_2568 | D08669 | 0 | hsa_79054 | D00267 |
| 1 | hsa_2892 | D00537 | 0 | hsa_2568 | D01279 | 0 | hsa_79054 | D00280 |
| 1 | hsa_2892 | D02696 | 0 | hsa_2568 | D00492 | 0 | hsa_79054 | D08448 |
| 1 | hsa_2892 | D04131 | 0 | hsa_2568 | D06517 | 0 | hsa_79054 | D07185 |
| 1 | hsa_2892 | D06656 | 0 | hsa_2568 | D08669 | 0 | hsa_79054 | D01104 |
| 1 | hsa_2892 | D08964 | 0 | hsa_2568 | D08009 | 0 | hsa_79054 | D09035 |
| 1 | hsa_2892 | D09035 | 0 | hsa_2568 | D04257 | 0 | hsa_79054 | D08138 |
| 1 | hsa_2893 | D00537 | 0 | hsa_2568 | D07881 | 0 | hsa_79054 | D00430 |
| 1 | hsa_2893 | D02696 | 0 | hsa_2568 | D05442 | 0 | hsa_79054 | D07678 |
| 1 | hsa_2893 | D04131 | 0 | hsa_2568 | D01744 | 0 | hsa_79054 | D08111 |
| 1 | hsa_2893 | D06656 | 0 | hsa_2568 | D08009 | 0 | hsa_79054 | D09757 |
| 1 | hsa_2893 | D08964 | 0 | hsa_2568 | D04226 | 0 | hsa_79054 | D04721 |
| 1 | hsa_2893 | D09035 | 0 | hsa_2568 | D01593 | 0 | hsa_79054 | D02252 |
| 1 | hsa_2902 | D00848 | 0 | hsa_2568 | D08987 | 0 | hsa_79054 | D02410 |
| 1 | hsa_2902 | D02410 | 0 | hsa_2568 | D10702 | 0 | hsa_79054 | D07272 |
| 1 | hsa_2902 | D02780 | 0 | hsa_2568 | D01254 | 0 | hsa_79054 | D08377 |
| 1 | hsa_2902 | D02973 | 0 | hsa_2568 | D01293 | 0 | hsa_8001 | D08101 |
| 1 | hsa_2902 | D03679 | 0 | hsa_2568 | D07993 | 0 | hsa_8001 | D09789 |
| 1 | hsa_2902 | D03742 | 0 | hsa_2568 | D01316 | 0 | hsa_8001 | D01292 |
| 1 | hsa_2902 | D03746 | 0 | hsa_2568 | D00492 | 0 | hsa_8001 | D00637 |
| 1 | hsa_2902 | D04226 | 0 | hsa_2568 | D00457 | 0 | hsa_8001 | D00375 |
| 1 | hsa_2902 | D04308 | 0 | hsa_2568 | D00250 | 0 | hsa_8001 | D03655 |
| 1 | hsa_2902 | D04728 | 0 | hsa_2568 | D06146 | 0 | hsa_8001 | D07881 |
| 1 | hsa_2902 | D04905 | 0 | hsa_2568 | D00399 | 0 | hsa_8001 | D08001 |
| 1 | hsa_2902 | D05145 | 0 | hsa_2741 | D02385 | 0 | hsa_8001 | D01514 |
| 1 | hsa_2902 | D05447 | 0 | hsa_2741 | D08377 | 0 | hsa_8001 | D08435 |
| 1 | hsa_2902 | D05453 | 0 | hsa_2741 | D08215 | 0 | hsa_8001 | D01292 |
| 1 | hsa_2902 | D05714 | 0 | hsa_2741 | D10627 | 0 | hsa_8001 | D01316 |
| 1 | hsa_2902 | D06146 | 0 | hsa_2741 | D00438 | 0 | hsa_8001 | D09383 |
| 1 | hsa_2902 | D07058 | 0 | hsa_2741 | D07272 | 0 | hsa_8001 | D05442 |
| 1 | hsa_2902 | D07283 | 0 | hsa_2741 | D01554 | 0 | hsa_8001 | D00764 |
| 1 | hsa_2902 | D07978 | 0 | hsa_2741 | D04257 | 0 | hsa_8001 | D09539 |
| 1 | hsa_2902 | D07979 | 0 | hsa_2741 | D00064 | 0 | hsa_8001 | D10626 |
| 1 | hsa_2902 | D08098 | 0 | hsa_2741 | D00557 | 0 | hsa_8001 | D01145 |
| 1 | hsa_2902 | D08100 | 0 | hsa_2741 | D07509 | 0 | hsa_8001 | D08356 |
| 1 | hsa_2902 | D08101 | 0 | hsa_2741 | D00375 | 0 | hsa_8001 | D08490 |
| 1 | hsa_2902 | D08174 | 0 | hsa_2741 | D01245 | 0 | hsa_8001 | D01562 |
| 1 | hsa_2902 | D08596 | 0 | hsa_2741 | D02103 | 0 | hsa_8001 | D10370 |
| 1 | hsa_2902 | D10627 | 0 | hsa_2741 | D00506 | 0 | hsa_8001 | D00499 |
| 1 | hsa_2903 | D00848 | 0 | hsa_2741 | D05145 | 0 | hsa_8001 | D02292 |
| 1 | hsa_2903 | D02410 | 0 | hsa_2741 | D02716 | 0 | hsa_8001 | D08340 |
| 1 | hsa_2903 | D02780 | 0 | hsa_2741 | D01856 | 0 | hsa_8001 | D01604 |
| 1 | hsa_2903 | D02973 | 0 | hsa_2741 | D02292 | 0 | hsa_8001 | D02202 |
| 1 | hsa_2903 | D03679 | 0 | hsa_2741 | D00376 | 0 | hsa_8001 | D04905 |
| 1 | hsa_2903 | D03742 | 0 | hsa_2741 | D08101 | 0 | hsa_8001 | D00765 |
| 1 | hsa_2903 | D03746 | 0 | hsa_2741 | D05714 | 0 | hsa_8001 | D02617 |
| 1 | hsa_2903 | D04226 | 0 | hsa_2741 | D00267 | 0 | hsa_8001 | D04308 |
| 1 | hsa_2903 | D04308 | 0 | hsa_2741 | D00252 | 0 | hsa_8001 | D03037 |
| 1 | hsa_2903 | D04728 | 0 | hsa_2741 | D01326 | 0 | hsa_8001 | D08356 |
| 1 | hsa_2903 | D04905 | 0 | hsa_2741 | D10470 | 0 | hsa_8001 | D00537 |
| 1 | hsa_2903 | D05145 | 0 | hsa_2741 | D00555 | 0 | hsa_8001 | D00700 |
| 1 | hsa_2903 | D05447 | 0 | hsa_2741 | D09382 | 0 | hsa_8001 | D01657 |
| 1 | hsa_2903 | D05453 | 0 | hsa_2741 | D04131 | 0 | hsa_8001 | D03742 |
| 1 | hsa_2903 | D05714 | 0 | hsa_2741 | D00643 | 0 | hsa_8001 | D03037 |
| 1 | hsa_2903 | D06146 | 0 | hsa_2741 | D08282 | 0 | hsa_8001 | D01293 |
| 1 | hsa_2903 | D07058 | 0 | hsa_2741 | D08145 | 0 | hsa_8001 | D01145 |
| 1 | hsa_2903 | D07283 | 0 | hsa_2741 | D05482 | 0 | hsa_8001 | D01810 |
| 1 | hsa_2903 | D07978 | 0 | hsa_2741 | D07784 | 0 | hsa_8001 | D08101 |
| 1 | hsa_2903 | D07979 | 0 | hsa_2741 | D07185 | 0 | hsa_8001 | D06146 |
| 1 | hsa_2903 | D08098 | 0 | hsa_2741 | D00375 | 0 | hsa_8001 | D09918 |
| 1 | hsa_2903 | D08100 | 0 | hsa_2741 | D06653 | 0 | hsa_8001 | D01810 |
| 1 | hsa_2903 | D08101 | 0 | hsa_2741 | D00512 | 0 | hsa_8001 | D09757 |
| 1 | hsa_2903 | D08174 | 0 | hsa_2741 | D08377 | 0 | hsa_8001 | D00329 |
| 1 | hsa_2903 | D08596 | 0 | hsa_2741 | D01104 | 0 | hsa_8001 | D00512 |
| 1 | hsa_2903 | D10627 | 0 | hsa_2741 | D04955 | 0 | hsa_8001 | D00064 |
| 1 | hsa_2904 | D00848 | 0 | hsa_2741 | D08435 | 0 | hsa_8001 | D04882 |
| 1 | hsa_2904 | D02410 | 0 | hsa_2741 | D00733 | 0 | hsa_8001 | D09917 |
| 1 | hsa_2904 | D02780 | 0 | hsa_2741 | D00250 | 0 | hsa_8001 | D00552 |
| 1 | hsa_2904 | D02973 | 0 | hsa_2741 | D01243 | 0 | hsa_8001 | D02220 |
| 1 | hsa_2904 | D03100 | 0 | hsa_2741 | D04282 | 0 | hsa_8001 | D07442 |
| 1 | hsa_2904 | D03679 | 0 | hsa_2741 | D07881 | 0 | hsa_8001 | D08275 |
| 1 | hsa_2904 | D03742 | 0 | hsa_2741 | D01564 | 0 | hsa_8001 | D02252 |
| 1 | hsa_2904 | D03746 | 0 | hsa_2741 | D04955 | 0 | hsa_8645 | D00643 |
| 1 | hsa_2904 | D04226 | 0 | hsa_2741 | D08127 | 0 | hsa_8645 | D01245 |
| 1 | hsa_2904 | D04308 | 0 | hsa_2742 | D01856 | 0 | hsa_8645 | D03742 |
| 1 | hsa_2904 | D04728 | 0 | hsa_2742 | D08215 | 0 | hsa_8645 | D09215 |
| 1 | hsa_2904 | D04905 | 0 | hsa_2742 | D02202 | 0 | hsa_8645 | D01593 |
| 1 | hsa_2904 | D05145 | 0 | hsa_2742 | D00512 | 0 | hsa_8645 | D00766 |
| 1 | hsa_2904 | D05447 | 0 | hsa_2742 | D00551 | 0 | hsa_8645 | D03562 |
| 1 | hsa_2904 | D05453 | 0 | hsa_2742 | D02347 | 0 | hsa_8645 | D00710 |
| 1 | hsa_2904 | D05714 | 0 | hsa_2742 | D04282 | 0 | hsa_8645 | D06653 |
| 1 | hsa_2904 | D06146 | 0 | hsa_2742 | D00098 | 0 | hsa_8645 | D02617 |
| 1 | hsa_2904 | D06204 | 0 | hsa_2742 | D00303 | 0 | hsa_8645 | D01908 |
| 1 | hsa_2904 | D07058 | 0 | hsa_2742 | D00252 | 0 | hsa_8645 | D01268 |
| 1 | hsa_2904 | D07283 | 0 | hsa_2742 | D09368 | 0 | hsa_8645 | D07552 |
| 1 | hsa_2904 | D07978 | 0 | hsa_2742 | D04905 | 0 | hsa_8645 | D02617 |
| 1 | hsa_2904 | D07979 | 0 | hsa_2742 | D02780 | 0 | hsa_8645 | D08001 |
| 1 | hsa_2904 | D08098 | 0 | hsa_2742 | D00758 | 0 | hsa_8645 | D09383 |
| 1 | hsa_2904 | D08100 | 0 | hsa_2742 | D09789 | 0 | hsa_8645 | D01292 |
| 1 | hsa_2904 | D08101 | 0 | hsa_2742 | D04303 | 0 | hsa_8645 | D07283 |
| 1 | hsa_2904 | D08174 | 0 | hsa_2742 | D01104 | 0 | hsa_8645 | D09035 |
| 1 | hsa_2904 | D08596 | 0 | hsa_2742 | D03655 | 0 | hsa_8645 | D08060 |
| 1 | hsa_2904 | D10627 | 0 | hsa_2742 | D07272 | 0 | hsa_8645 | D08964 |
| 1 | hsa_2905 | D00848 | 0 | hsa_2742 | D06388 | 0 | hsa_8645 | D00706 |
| 1 | hsa_2905 | D02410 | 0 | hsa_2742 | D03742 | 0 | hsa_8645 | D04257 |
| 1 | hsa_2905 | D02780 | 0 | hsa_2742 | D03155 | 0 | hsa_8645 | D00706 |
| 1 | hsa_2905 | D02973 | 0 | hsa_2742 | D00532 | 0 | hsa_8645 | D00370 |
| 1 | hsa_2905 | D03679 | 0 | hsa_2742 | D05447 | 0 | hsa_8645 | D09368 |
| 1 | hsa_2905 | D03742 | 0 | hsa_2742 | D06517 | 0 | hsa_8645 | D00759 |
| 1 | hsa_2905 | D03746 | 0 | hsa_2742 | D02616 | 0 | hsa_8645 | D09612 |
| 1 | hsa_2905 | D04226 | 0 | hsa_2742 | D00418 | 0 | hsa_8645 | D02973 |
| 1 | hsa_2905 | D04308 | 0 | hsa_2742 | D01071 | 0 | hsa_8645 | D06392 |
| 1 | hsa_2905 | D04728 | 0 | hsa_2742 | D01254 | 0 | hsa_8645 | D02275 |
| 1 | hsa_2905 | D04905 | 0 | hsa_2742 | D00532 | 0 | hsa_8645 | D03492 |
| 1 | hsa_2905 | D05145 | 0 | hsa_2742 | D06656 | 0 | hsa_8645 | D09368 |
| 1 | hsa_2905 | D05447 | 0 | hsa_2742 | D02252 | 0 | hsa_8645 | D05447 |
| 1 | hsa_2905 | D05453 | 0 | hsa_2742 | D10470 | 0 | hsa_8645 | D09789 |
| 1 | hsa_2905 | D05714 | 0 | hsa_2742 | D03914 | 0 | hsa_8645 | D07595 |
| 1 | hsa_2905 | D06146 | 0 | hsa_2742 | D07283 | 0 | hsa_8645 | D08669 |
| 1 | hsa_2905 | D07058 | 0 | hsa_2742 | D01230 | 0 | hsa_8645 | D00380 |
| 1 | hsa_2905 | D07283 | 0 | hsa_2742 | D00375 | 0 | hsa_8645 | D08377 |
| 1 | hsa_2905 | D07978 | 0 | hsa_2742 | D00555 | 0 | hsa_8645 | D00531 |
| 1 | hsa_2905 | D07979 | 0 | hsa_2742 | D01562 | 0 | hsa_8645 | D08275 |
| 1 | hsa_2905 | D08098 | 0 | hsa_2742 | D08892 | 0 | hsa_8645 | D00064 |
| 1 | hsa_2905 | D08100 | 0 | hsa_2742 | D00636 | 0 | hsa_8645 | D07979 |
| 1 | hsa_2905 | D08101 | 0 | hsa_2742 | D00430 | 0 | hsa_8645 | D08155 |
| 1 | hsa_2905 | D08174 | 0 | hsa_2742 | D02252 | 0 | hsa_8645 | D03679 |
| 1 | hsa_2905 | D08596 | 0 | hsa_2742 | D06204 | 0 | hsa_8645 | D03914 |
| 1 | hsa_2905 | D10627 | 0 | hsa_2742 | D09382 | 0 | hsa_8645 | D02275 |
| 1 | hsa_2906 | D00848 | 0 | hsa_2742 | D08669 | 0 | hsa_8645 | D00303 |
| 1 | hsa_2906 | D02410 | 0 | hsa_2742 | D00636 | 0 | hsa_8645 | D01740 |
| 1 | hsa_2906 | D02780 | 0 | hsa_2742 | D00492 | 0 | hsa_8645 | D00506 |
| 1 | hsa_2906 | D02973 | 0 | hsa_2742 | D09917 | 0 | hsa_8645 | D00294 |
| 1 | hsa_2906 | D03679 | 0 | hsa_2742 | D08215 | 0 | hsa_8645 | D02910 |
| 1 | hsa_2906 | D03742 | 0 | hsa_2742 | D01173 | 0 | hsa_8911 | D01316 |
| 1 | hsa_2906 | D03746 | 0 | hsa_2742 | D01785 | 0 | hsa_8911 | D00611 |
| 1 | hsa_2906 | D04226 | 0 | hsa_2742 | D01455 | 0 | hsa_8911 | D07999 |
| 1 | hsa_2906 | D04308 | 0 | hsa_2742 | D06388 | 0 | hsa_8911 | D00700 |
| 1 | hsa_2906 | D04728 | 0 | hsa_2742 | D03547 | 0 | hsa_8911 | D05447 |
| 1 | hsa_2906 | D04905 | 0 | hsa_2742 | D04226 | 0 | hsa_8911 | D01303 |
| 1 | hsa_2906 | D05145 | 0 | hsa_2742 | D01657 | 0 | hsa_8911 | D06146 |
| 1 | hsa_2906 | D05447 | 0 | hsa_2742 | D02096 | 0 | hsa_8911 | D00552 |
| 1 | hsa_2906 | D05453 | 0 | hsa_2742 | D02364 | 0 | hsa_8911 | D00064 |
| 1 | hsa_2906 | D05714 | 0 | hsa_2742 | D09382 | 0 | hsa_8911 | D07283 |
| 1 | hsa_2906 | D06146 | 0 | hsa_2743 | D02045 | 0 | hsa_8911 | D00555 |
| 1 | hsa_2906 | D07058 | 0 | hsa_2743 | D04282 | 0 | hsa_8911 | D00551 |
| 1 | hsa_2906 | D07283 | 0 | hsa_2743 | D07999 | 0 | hsa_8911 | D02103 |
| 1 | hsa_2906 | D07978 | 0 | hsa_2743 | D02252 | 0 | hsa_8911 | D00693 |
| 1 | hsa_2906 | D07979 | 0 | hsa_2743 | D06517 | 0 | hsa_8911 | D01303 |
| 1 | hsa_2906 | D08098 | 0 | hsa_2743 | D02594 | 0 | hsa_8911 | D08448 |
| 1 | hsa_2906 | D08100 | 0 | hsa_2743 | D01856 | 0 | hsa_8911 | D09215 |
| 1 | hsa_2906 | D08101 | 0 | hsa_2743 | D03914 | 0 | hsa_8911 | D01286 |
| 1 | hsa_2906 | D08174 | 0 | hsa_2743 | D01268 | 0 | hsa_8911 | D02364 |
| 1 | hsa_2906 | D08596 | 0 | hsa_2743 | D06146 | 0 | hsa_8911 | D08667 |
| 1 | hsa_2906 | D10627 | 0 | hsa_2743 | D01286 | 0 | hsa_8911 | D07993 |
| 1 | hsa_3736 | D01554 | 0 | hsa_2743 | D07894 | 0 | hsa_8911 | D00199 |
| 1 | hsa_3736 | D08377 | 0 | hsa_2743 | D07894 | 0 | hsa_8911 | D07272 |
| 1 | hsa_3741 | D03547 | 0 | hsa_2743 | D07595 | 0 | hsa_8911 | D00848 |
| 1 | hsa_3752 | D00636 | 0 | hsa_2743 | D08116 | 0 | hsa_8911 | D07520 |
| 1 | hsa_3752 | D01856 | 0 | hsa_2743 | D07962 | 0 | hsa_8911 | D02624 |
| 1 | hsa_3752 | D02910 | 0 | hsa_2743 | D01657 | 0 | hsa_8911 | D01908 |
| 1 | hsa_3752 | D06652 | 0 | hsa_2743 | D00695 | 0 | hsa_8911 | D00531 |
| 1 | hsa_3752 | D06653 | 0 | hsa_2743 | D07962 | 0 | hsa_8911 | D01408 |
| 1 | hsa_3757 | D03037 | 0 | hsa_2743 | D01554 | 0 | hsa_8911 | D00329 |
| 1 | hsa_3757 | D03732 | 0 | hsa_2743 | D00764 | 0 | hsa_8911 | D02410 |
| 1 | hsa_3757 | D04955 | 0 | hsa_2743 | D00643 | 0 | hsa_8911 | D04282 |
| 1 | hsa_3757 | D08060 | 0 | hsa_2743 | D08421 | 0 | hsa_8911 | D07886 |
| 1 | hsa_3757 | D09757 | 0 | hsa_2743 | D00550 | 0 | hsa_8911 | D03562 |
| 1 | hsa_3767 | D00294 | 0 | hsa_2743 | D10627 | 0 | hsa_8911 | D04849 |
| 1 | hsa_3767 | D00336 | 0 | hsa_2743 | D04308 | 0 | hsa_8911 | D00064 |
| 1 | hsa_3767 | D00380 | 0 | hsa_2743 | D10626 | 0 | hsa_8911 | D08435 |
| 1 | hsa_3767 | D00418 | 0 | hsa_2743 | D04905 | 0 | hsa_8912 | D08840 |
| 1 | hsa_3767 | D01810 | 0 | hsa_2743 | D00766 | 0 | hsa_8912 | D01230 |
| 1 | hsa_3767 | D02385 | 0 | hsa_2743 | D07886 | 0 | hsa_8912 | D01810 |
| 1 | hsa_3767 | D05482 | 0 | hsa_2743 | D09757 | 0 | hsa_8912 | D08138 |
| 1 | hsa_3784 | D04741 | 0 | hsa_2743 | D00531 | 0 | hsa_8912 | D07979 |
| 1 | hsa_3785 | D09569 | 0 | hsa_2743 | D01740 | 0 | hsa_8912 | D00336 |
| 1 | hsa_43 | D09917 | 0 | hsa_2743 | D01553 | 0 | hsa_8912 | D07894 |
| 1 | hsa_43 | D09918 | 0 | hsa_2743 | D10111 | 0 | hsa_8912 | D02616 |
| 1 | hsa_55584 | D00612 | 0 | hsa_2743 | D00706 | 0 | hsa_8912 | D05703 |
| 1 | hsa_55584 | D02202 | 0 | hsa_2743 | D08100 | 0 | hsa_8912 | D09382 |
| 1 | hsa_55584 | D02364 | 0 | hsa_2743 | D00311 | 0 | hsa_8912 | D01243 |
| 1 | hsa_55584 | D08138 | 0 | hsa_2743 | D07447 | 0 | hsa_8912 | D07185 |
| 1 | hsa_55799 | D02716 | 0 | hsa_2743 | D07894 | 0 | hsa_8912 | D00759 |
| 1 | hsa_55799 | D09539 | 0 | hsa_2743 | D02780 | 0 | hsa_8912 | D02973 |
| 1 | hsa_55879 | D00267 | 0 | hsa_2743 | D00252 | 0 | hsa_8912 | D00329 |
| 1 | hsa_55879 | D00280 | 0 | hsa_2743 | D02347 | 0 | hsa_8912 | D03914 |
| 1 | hsa_55879 | D00311 | 0 | hsa_2743 | D01328 | 0 | hsa_8912 | D04905 |
| 1 | hsa_55879 | D00365 | 0 | hsa_2743 | D08377 | 0 | hsa_8912 | D08009 |
| 1 | hsa_55879 | D00370 | 0 | hsa_2743 | D07450 | 0 | hsa_8912 | D02780 |
| 1 | hsa_55879 | D00376 | 0 | hsa_2743 | D02283 | 0 | hsa_8912 | D03492 |
| 1 | hsa_55879 | D00387 | 0 | hsa_2743 | D08100 | 0 | hsa_8912 | D07058 |
| 1 | hsa_55879 | D00430 | 0 | hsa_2743 | D08116 | 0 | hsa_8912 | D02364 |
| 1 | hsa_55879 | D00457 | 0 | hsa_2890 | D00399 | 0 | hsa_8912 | D03547 |
| 1 | hsa_55879 | D00470 | 0 | hsa_2890 | D03492 | 0 | hsa_8912 | D08145 |
| 1 | hsa_55879 | D00500 | 0 | hsa_2890 | D09382 | 0 | hsa_8912 | D00303 |
| 1 | hsa_55879 | D00506 | 0 | hsa_2890 | D04905 | 0 | hsa_8912 | D02624 |
| 1 | hsa_55879 | D00531 | 0 | hsa_2890 | D10112 | 0 | hsa_8912 | D00760 |
| 1 | hsa_55879 | D00532 | 0 | hsa_2890 | D02696 | 0 | hsa_8912 | D07283 |
| 1 | hsa_55879 | D00555 | 0 | hsa_2890 | D04257 | 0 | hsa_8912 | D03547 |
| 1 | hsa_55879 | D00557 | 0 | hsa_2890 | D00551 | 0 | hsa_8912 | D08377 |
| 1 | hsa_55879 | D00693 | 0 | hsa_2890 | D08127 | 0 | hsa_8912 | D08155 |
| 1 | hsa_55879 | D00694 | 0 | hsa_2890 | D07881 | 0 | hsa_8912 | D07962 |
| 1 | hsa_55879 | D00695 | 0 | hsa_2890 | D10112 | 0 | hsa_8912 | D07845 |
| 1 | hsa_55879 | D00696 | 0 | hsa_2890 | D09368 | 0 | hsa_8912 | D04308 |
| 1 | hsa_55879 | D00697 | 0 | hsa_2890 | D01071 | 0 | hsa_8912 | D00758 |
| 1 | hsa_55879 | D00700 | 0 | hsa_2890 | D01593 | 0 | hsa_8912 | D08935 |
| 1 | hsa_55879 | D00701 | 0 | hsa_2890 | D06653 | 0 | hsa_8912 | D00700 |
| 1 | hsa_55879 | D00706 | 0 | hsa_2890 | D08215 | 0 | hsa_8912 | D07678 |
| 1 | hsa_55879 | D00713 | 0 | hsa_2890 | D01564 | 0 | hsa_8912 | D10626 |
| 1 | hsa_55879 | D00714 | 0 | hsa_2890 | D00500 | 0 | hsa_8912 | D00499 |
| 1 | hsa_55879 | D01071 | 0 | hsa_2890 | D06204 | 0 | hsa_8912 | D08356 |
| 1 | hsa_55879 | D01230 | 0 | hsa_2890 | D00365 | 0 | hsa_8912 | D00267 |
| 1 | hsa_55879 | D01245 | 0 | hsa_2890 | D07058 | 0 | hsa_8912 | D08507 |
| 1 | hsa_55879 | D01253 | 0 | hsa_2890 | D01145 | 0 | hsa_8912 | D07509 |
| 1 | hsa_55879 | D01254 | 0 | hsa_2890 | D08669 | 0 | hsa_8912 | D01293 |
| 1 | hsa_55879 | D01268 | 0 | hsa_2890 | D01593 | 0 | hsa_8912 | D00457 |
| 1 | hsa_55879 | D01278 | 0 | hsa_2890 | D00365 | 0 | hsa_8912 | D07442 |
| 1 | hsa_55879 | D01279 | 0 | hsa_2890 | D00457 | 0 | hsa_8912 | D10111 |
| 1 | hsa_55879 | D01286 | 0 | hsa_2890 | D02347 | 0 | hsa_8912 | D03100 |
| 1 | hsa_55879 | D01292 | 0 | hsa_2890 | D05447 | 0 | hsa_8912 | D01230 |
| 1 | hsa_55879 | D01293 | 0 | hsa_2890 | D01245 | 0 | hsa_8912 | D01810 |
| 1 | hsa_55879 | D01310 | 0 | hsa_2890 | D00733 | 0 | hsa_8912 | D00764 |
| 1 | hsa_55879 | D01316 | 0 | hsa_2890 | D08394 | 0 | hsa_8912 | D04721 |
| 1 | hsa_55879 | D01328 | 0 | hsa_2890 | D00611 | 0 | hsa_8912 | D08935 |
| 1 | hsa_55879 | D01354 | 0 | hsa_2890 | D01243 | 0 | hsa_8912 | D08481 |
| 1 | hsa_55879 | D01372 | 0 | hsa_2890 | D08964 | 0 | hsa_8912 | D08283 |
| 1 | hsa_55879 | D01408 | 0 | hsa_2890 | D00713 | 0 | hsa_8913 | D00537 |
| 1 | hsa_55879 | D01514 | 0 | hsa_2890 | D02537 | 0 | hsa_8913 | D08655 |
| 1 | hsa_55879 | D01564 | 0 | hsa_2890 | D10626 | 0 | hsa_8913 | D00303 |
| 1 | hsa_55879 | D01593 | 0 | hsa_2890 | D05028 | 0 | hsa_8913 | D08138 |
| 1 | hsa_55879 | D01657 | 0 | hsa_2890 | D08060 | 0 | hsa_8913 | D08448 |
| 1 | hsa_55879 | D01740 | 0 | hsa_2890 | D00701 | 0 | hsa_8913 | D00550 |
| 1 | hsa_55879 | D01744 | 0 | hsa_2890 | D01810 | 0 | hsa_8913 | D10613 |
| 1 | hsa_55879 | D01758 | 0 | hsa_2890 | D06392 | 0 | hsa_8913 | D00549 |
| 1 | hsa_55879 | D02252 | 0 | hsa_2890 | D08127 | 0 | hsa_8913 | D01849 |
| 1 | hsa_55879 | D02253 | 0 | hsa_2890 | D08048 | 0 | hsa_8913 | D00329 |
| 1 | hsa_55879 | D02283 | 0 | hsa_2890 | D04131 | 0 | hsa_8913 | D03679 |
| 1 | hsa_55879 | D02594 | 0 | hsa_2890 | D02045 | 0 | hsa_8913 | D03547 |
| 1 | hsa_55879 | D02616 | 0 | hsa_2890 | D07678 | 0 | hsa_8913 | D01268 |
| 1 | hsa_55879 | D02617 | 0 | hsa_2890 | D00336 | 0 | hsa_8913 | D08060 |
| 1 | hsa_55879 | D02624 | 0 | hsa_2890 | D00636 | 0 | hsa_8913 | D08669 |
| 1 | hsa_55879 | D03155 | 0 | hsa_2891 | D00537 | 0 | hsa_8913 | D07881 |
| 1 | hsa_55879 | D03562 | 0 | hsa_2891 | D10470 | 0 | hsa_8913 | D00280 |
| 1 | hsa_55879 | D03737 | 0 | hsa_2891 | D03737 | 0 | hsa_8913 | D07784 |
| 1 | hsa_55879 | D04257 | 0 | hsa_2891 | D04728 | 0 | hsa_8913 | D07978 |
| 1 | hsa_55879 | D04282 | 0 | hsa_2891 | D08448 | 0 | hsa_8913 | D01316 |
| 1 | hsa_55879 | D04300 | 0 | hsa_2891 | D04955 | 0 | hsa_8913 | D06204 |
| 1 | hsa_55879 | D04721 | 0 | hsa_2891 | D07520 | 0 | hsa_8913 | D07409 |
| 1 | hsa_55879 | D04882 | 0 | hsa_2891 | D08048 | 0 | hsa_8913 | D02283 |
| 1 | hsa_55879 | D05028 | 0 | hsa_2891 | D00311 | 0 | hsa_8913 | D07881 |
| 1 | hsa_55879 | D06106 | 0 | hsa_2891 | D04048 | 0 | hsa_8913 | D00552 |
| 1 | hsa_55879 | D07326 | 0 | hsa_2891 | D02617 | 0 | hsa_8913 | D01292 |
| 1 | hsa_55879 | D07409 | 0 | hsa_2891 | D05028 | 0 | hsa_8913 | D08422 |
| 1 | hsa_55879 | D07784 | 0 | hsa_2891 | D09215 | 0 | hsa_8913 | D08987 |
| 1 | hsa_55879 | D08145 | 0 | hsa_2891 | D00848 | 0 | hsa_8913 | D08145 |
| 1 | hsa_55879 | D08283 | 0 | hsa_2891 | D01326 | 0 | hsa_8913 | D07784 |
| 1 | hsa_55879 | D08356 | 0 | hsa_2891 | D04282 | 0 | hsa_8913 | D05703 |
| 1 | hsa_55879 | D08481 | 0 | hsa_2891 | D04741 | 0 | hsa_8913 | D08155 |
| 1 | hsa_55879 | D08507 | 0 | hsa_2891 | D09368 | 0 | hsa_8913 | D07678 |
| 1 | hsa_55879 | D08690 | 0 | hsa_2891 | D00611 | 0 | hsa_8913 | D07450 |
| 1 | hsa_55879 | D08840 | 0 | hsa_2891 | D03655 | 0 | hsa_8913 | D01243 |
| 1 | hsa_55879 | D10194 | 0 | hsa_2891 | D00550 | 0 | hsa_8913 | D00643 |
| 1 | hsa_57053 | D00612 | 0 | hsa_2891 | D07999 | 0 | hsa_8913 | D00767 |
| 1 | hsa_57053 | D02202 | 0 | hsa_2891 | D01810 | 0 | hsa_8913 | D08111 |
| 1 | hsa_57053 | D02364 | 0 | hsa_2891 | D04048 | 0 | hsa_8913 | D08667 |
| 1 | hsa_57053 | D08138 | 0 | hsa_2891 | D01316 | 0 | hsa_8913 | D00531 |
| 1 | hsa_5743 | D08275 | 0 | hsa_2891 | D06282 | 0 | hsa_8913 | D01354 |
| 1 | hsa_6261 | D02274 | 0 | hsa_2891 | D06204 | 0 | hsa_8913 | D10626 |
| 1 | hsa_6261 | D02347 | 0 | hsa_2891 | D07272 | 0 | hsa_8913 | D00329 |
| 1 | hsa_6262 | D02274 | 0 | hsa_2891 | D07494 | 0 | hsa_8913 | D02385 |
| 1 | hsa_6262 | D02347 | 0 | hsa_2891 | D00701 | 0 | hsa_8913 | D10702 |
| 1 | hsa_6263 | D02274 | 0 | hsa_2891 | D01785 | 0 | hsa_8913 | D00758 |
| 1 | hsa_6263 | D02347 | 0 | hsa_2891 | D09368 | 0 | hsa_8913 | D08215 |
| 1 | hsa_6323 | D00199 | 0 | hsa_2891 | D08655 | 0 | hsa_8913 | D00370 |
| 1 | hsa_6323 | D00252 | 0 | hsa_2891 | D01145 | 0 | hsa_8913 | D03655 |
| 1 | hsa_6323 | D00303 | 0 | hsa_2891 | D08669 | 0 | hsa_8913 | D08111 |
| 1 | hsa_6323 | D00375 | 0 | hsa_2891 | D06517 | 0 | hsa_8913 | D00500 |
| 1 | hsa_6323 | D00512 | 0 | hsa_2891 | D07978 | 0 | hsa_8913 | D00848 |
| 1 | hsa_6323 | D00533 | 0 | hsa_2891 | D01740 | 0 | hsa_8913 | D01310 |
| 1 | hsa_6323 | D00551 | 0 | hsa_2891 | D00733 | 0 | hsa_8913 | D08507 |
| 1 | hsa_6323 | D00552 | 0 | hsa_2891 | D00760 | 0 | hsa_8913 | D08935 |
| 1 | hsa_6323 | D00637 | 0 | hsa_2891 | D07845 | 0 | hsa_8913 | D00199 |
| 1 | hsa_6323 | D00643 | 0 | hsa_2892 | D00767 | 0 | hsa_8913 | D05714 |
| 1 | hsa_6323 | D00733 | 0 | hsa_2892 | D08101 | 0 | hsa_8913 | D08481 |
| 1 | hsa_6323 | D01326 | 0 | hsa_2892 | D02973 | 0 | hsa_8913 | D00457 |
| 1 | hsa_6323 | D01455 | 0 | hsa_2892 | D04741 | 0 | hsa_8913 | D05447 |
| 1 | hsa_6323 | D01479 | 0 | hsa_2892 | D08421 | 0 | hsa_8973 | D00492 |
| 1 | hsa_6323 | D01785 | 0 | hsa_2892 | D00531 | 0 | hsa_8973 | D00550 |
| 1 | hsa_6323 | D02096 | 0 | hsa_2892 | D03732 | 0 | hsa_8973 | D00706 |
| 1 | hsa_6323 | D02103 | 0 | hsa_2892 | D08283 | 0 | hsa_8973 | D07283 |
| 1 | hsa_6323 | D02220 | 0 | hsa_2892 | D09367 | 0 | hsa_8973 | D03547 |
| 1 | hsa_6323 | D02969 | 0 | hsa_2892 | D00370 | 0 | hsa_8973 | D01293 |
| 1 | hsa_6323 | D03492 | 0 | hsa_2892 | D06388 | 0 | hsa_8973 | D02096 |
| 1 | hsa_6323 | D05077 | 0 | hsa_2892 | D01145 | 0 | hsa_8973 | D01785 |
| 1 | hsa_6323 | D06172 | 0 | hsa_2892 | D09539 | 0 | hsa_8973 | D01479 |
| 1 | hsa_6323 | D06517 | 0 | hsa_2892 | D02594 | 0 | hsa_8973 | D10112 |
| 1 | hsa_6323 | D07442 | 0 | hsa_2892 | D02347 | 0 | hsa_8973 | D00555 |
| 1 | hsa_6323 | D07595 | 0 | hsa_2892 | D01278 | 0 | hsa_8973 | D06172 |
| 1 | hsa_6323 | D07894 | 0 | hsa_2892 | D03100 | 0 | hsa_8973 | D01455 |
| 1 | hsa_6323 | D07962 | 0 | hsa_2892 | D08481 | 0 | hsa_8973 | D09539 |
| 1 | hsa_6323 | D07993 | 0 | hsa_2892 | D00380 | 0 | hsa_8973 | D07520 |
| 1 | hsa_6323 | D08048 | 0 | hsa_2892 | D04282 | 0 | hsa_8973 | D08060 |
| 1 | hsa_6323 | D08127 | 0 | hsa_2892 | D03562 | 0 | hsa_8973 | D00064 |
| 1 | hsa_6323 | D08215 | 0 | hsa_2892 | D00293 | 0 | hsa_8973 | D07881 |
| 1 | hsa_6323 | D08394 | 0 | hsa_2892 | D09382 | 0 | hsa_8973 | D01293 |
| 1 | hsa_6323 | D08421 | 0 | hsa_2892 | D06652 | 0 | hsa_8973 | D02616 |
| 1 | hsa_6323 | D08435 | 0 | hsa_2892 | D01785 | 0 | hsa_8973 | D07999 |
| 1 | hsa_6323 | D08458 | 0 | hsa_2892 | D00011 | 0 | hsa_8973 | D07881 |
| 1 | hsa_6323 | D08459 | 0 | hsa_2892 | D00399 | 0 | hsa_8973 | D00557 |
| 1 | hsa_6323 | D09215 | 0 | hsa_2892 | D00765 | 0 | hsa_8973 | D07971 |
| 1 | hsa_6323 | D09612 | 0 | hsa_2892 | D04131 | 0 | hsa_8973 | D08655 |
| 1 | hsa_6336 | D04048 | 0 | hsa_2892 | D01604 | 0 | hsa_8973 | D01593 |
| 1 | hsa_6336 | D07552 | 0 | hsa_2892 | D01553 | 0 | hsa_8973 | D08356 |
| 1 | hsa_6336 | D07678 | 0 | hsa_2892 | D00765 | 0 | hsa_8973 | D00764 |
| 1 | hsa_6336 | D07881 | 0 | hsa_2892 | D08098 | 0 | hsa_8973 | D01908 |
| 1 | hsa_6336 | D08116 | 0 | hsa_2892 | D06146 | 0 | hsa_8973 | D07058 |
| 1 | hsa_6336 | D08448 | 0 | hsa_2892 | D02275 | 0 | hsa_8973 | D00531 |
| 1 | hsa_6336 | D08490 | 0 | hsa_2892 | D07978 | 0 | hsa_8973 | D00695 |
| 1 | hsa_6337 | D00386 | 0 | hsa_2892 | D00250 | 0 | hsa_8973 | D01740 |
| 1 | hsa_6337 | D07447 | 0 | hsa_2892 | D00376 | 0 | hsa_8973 | D01856 |
| 1 | hsa_6338 | D00386 | 0 | hsa_2892 | D10693 | 0 | hsa_8973 | D08283 |
| 1 | hsa_6338 | D07447 | 0 | hsa_2892 | D00764 | 0 | hsa_8973 | D09215 |
| 1 | hsa_6340 | D00386 | 0 | hsa_2893 | D04721 | 0 | hsa_8973 | D07494 |
| 1 | hsa_6340 | D07447 | 0 | hsa_2893 | D02274 | 0 | hsa_8973 | D08935 |
| 1 | hsa_6557 | D00331 | 0 | hsa_2893 | D06282 | 0 | hsa_8973 | D08394 |
| 1 | hsa_6557 | D07999 | 0 | hsa_2893 | D00808 | 0 | hsa_8973 | D00733 |
| 1 | hsa_6557 | D08001 | 0 | hsa_2893 | D00438 | 0 | hsa_8973 | D02347 |
| 1 | hsa_6833 | D00418 | 0 | hsa_2893 | D07595 | 0 | hsa_8973 | D00370 |
| 1 | hsa_6833 | D01810 | 0 | hsa_2893 | D01604 | 0 | hsa_8973 | D07894 |
| 1 | hsa_6833 | D02385 | 0 | hsa_2893 | D07962 | 0 | hsa_8973 | D06656 |
| 1 | hsa_6833 | D05482 | 0 | hsa_2893 | D05442 | 0 | hsa_8973 | D08217 |
| 1 | hsa_7442 | D00250 | 0 | hsa_2893 | D00418 | 0 | hsa_8973 | D02385 |
| 1 | hsa_7442 | D06388 | 0 | hsa_2893 | D02617 | 0 | hsa_8973 | D02716 |
| 1 | hsa_7442 | D08282 | 0 | hsa_2893 | D00370 | 0 | hsa_8973 | D08892 |
| 1 | hsa_7442 | D10370 | 0 | hsa_2893 | D01278 | 0 | hsa_8973 | D00549 |
| 1 | hsa_775 | D00631 | 0 | hsa_2893 | D00733 | 0 | hsa_8973 | D01554 |
| 1 | hsa_775 | D01104 | 0 | hsa_2893 | D06146 | 0 | hsa_8973 | D03737 |
| 1 | hsa_775 | D01145 | 0 | hsa_2893 | D08667 | 0 | hsa_8973 | D07971 |
| 1 | hsa_775 | D01173 | 0 | hsa_2893 | D08356 | 0 | hsa_8973 | D09917 |
| 1 | hsa_775 | D01553 | 0 | hsa_2893 | D01514 | 0 | hsa_8973 | D04905 |
| 1 | hsa_775 | D01562 | 0 | hsa_2893 | D01326 | 0 | hsa_8973 | D00311 |
| 1 | hsa_775 | D01604 | 0 | hsa_2893 | D02410 | 0 | hsa_8973 | D04308 |
| 1 | hsa_775 | D01849 | 0 | hsa_2893 | D03746 | 0 | hsa_8973 | D00549 |
| 1 | hsa_775 | D01908 | 0 | hsa_2893 | D09382 | 0 | hsa_8973 | D03737 |
| 1 | hsa_775 | D02045 | 0 | hsa_2893 | D01856 | 0 | hsa_8989 | D01604 |
| 1 | hsa_775 | D02537 | 0 | hsa_2893 | D08377 | 0 | hsa_8989 | D06146 |
| 1 | hsa_775 | D03655 | 0 | hsa_2893 | D01604 | 0 | hsa_8989 | D02616 |
| 1 | hsa_775 | D03914 | 0 | hsa_2893 | D07993 | 0 | hsa_8989 | D01657 |
| 1 | hsa_775 | D04657 | 0 | hsa_2893 | D00199 | 0 | hsa_8989 | D05077 |
| 1 | hsa_775 | D05442 | 0 | hsa_2893 | D02364 | 0 | hsa_8989 | D00733 |
| 1 | hsa_775 | D07185 | 0 | hsa_2893 | D04882 | 0 | hsa_8989 | D03100 |
| 1 | hsa_775 | D07450 | 0 | hsa_2893 | D09382 | 0 | hsa_8989 | D08481 |
| 1 | hsa_775 | D07494 | 0 | hsa_2893 | D07494 | 0 | hsa_8989 | D05453 |
| 1 | hsa_775 | D07509 | 0 | hsa_2893 | D04131 | 0 | hsa_8989 | D01514 |
| 1 | hsa_775 | D07520 | 0 | hsa_2893 | D00759 | 0 | hsa_8989 | D01328 |
| 1 | hsa_775 | D07845 | 0 | hsa_2893 | D00499 | 0 | hsa_8989 | D08111 |
| 1 | hsa_775 | D07886 | 0 | hsa_2893 | D00696 | 0 | hsa_8989 | D02103 |
| 1 | hsa_775 | D07943 | 0 | hsa_2893 | D01849 | 0 | hsa_8989 | D02292 |
| 1 | hsa_775 | D08009 | 0 | hsa_2893 | D04226 | 0 | hsa_8989 | D08111 |
| 1 | hsa_775 | D08111 | 0 | hsa_2893 | D02696 | 0 | hsa_8989 | D02973 |
| 1 | hsa_775 | D08155 | 0 | hsa_2893 | D08459 | 0 | hsa_8989 | D02364 |
| 1 | hsa_775 | D08270 | 0 | hsa_2893 | D00294 | 0 | hsa_8989 | D04300 |
| 1 | hsa_775 | D08340 | 0 | hsa_2893 | D08690 | 0 | hsa_8989 | D00532 |
| 1 | hsa_775 | D08892 | 0 | hsa_2893 | D08060 | 0 | hsa_8989 | D01328 |
| 1 | hsa_775 | D09789 | 0 | hsa_2893 | D10370 | 0 | hsa_8989 | D08009 |
| 1 | hsa_776 | D00616 | 0 | hsa_2893 | D00380 | 0 | hsa_8989 | D00304 |
| 1 | hsa_776 | D00619 | 0 | hsa_2893 | D07894 | 0 | hsa_8989 | D03492 |
| 1 | hsa_776 | D00631 | 0 | hsa_2893 | D08377 | 0 | hsa_8989 | D01593 |
| 1 | hsa_776 | D01104 | 0 | hsa_2893 | D00533 | 0 | hsa_8989 | D01316 |
| 1 | hsa_776 | D01145 | 0 | hsa_2893 | D01657 | 0 | hsa_8989 | D00763 |
| 1 | hsa_776 | D01173 | 0 | hsa_2893 | D01564 | 0 | hsa_8989 | D00267 |
| 1 | hsa_776 | D01553 | 0 | hsa_2893 | D01173 | 0 | hsa_8989 | D01455 |
| 1 | hsa_776 | D01562 | 0 | hsa_2893 | D01408 | 0 | hsa_8989 | D08270 |
| 1 | hsa_776 | D01604 | 0 | hsa_2903 | D07552 | 0 | hsa_8989 | D06392 |
| 1 | hsa_776 | D01849 | 0 | hsa_2903 | D00552 | 0 | hsa_8989 | D04955 |
| 1 | hsa_776 | D01908 | 0 | hsa_2903 | D09918 | 0 | hsa_8989 | D00643 |
| 1 | hsa_776 | D01969 | 0 | hsa_2903 | D08435 | 0 | hsa_8989 | D03742 |
| 1 | hsa_776 | D02045 | 0 | hsa_2903 | D00553 | 0 | hsa_8989 | D08155 |
| 1 | hsa_776 | D02537 | 0 | hsa_2903 | D01785 | 0 | hsa_8989 | D00767 |
| 1 | hsa_776 | D03655 | 0 | hsa_2903 | D01856 | 0 | hsa_8989 | D00557 |
| 1 | hsa_776 | D03830 | 0 | hsa_2903 | D09569 | 0 | hsa_8989 | D00550 |
| 1 | hsa_776 | D03914 | 0 | hsa_2903 | D08215 | 0 | hsa_8989 | D00311 |
| 1 | hsa_776 | D04657 | 0 | hsa_2903 | D00380 | 0 | hsa_8989 | D02292 |
| 1 | hsa_776 | D05442 | 0 | hsa_2903 | D06653 | 0 | hsa_8989 | D02103 |
| 1 | hsa_776 | D07185 | 0 | hsa_2903 | D02910 | 0 | hsa_8989 | D00759 |
| 1 | hsa_776 | D07450 | 0 | hsa_2903 | D07894 | 0 | hsa_8989 | D00506 |
| 1 | hsa_776 | D07494 | 0 | hsa_2903 | D04741 | 0 | hsa_8989 | D01372 |
| 1 | hsa_776 | D07509 | 0 | hsa_2903 | D07678 | 0 | hsa_8989 | D00550 |
| 1 | hsa_776 | D07520 | 0 | hsa_2904 | D07999 | 0 | hsa_8989 | D01173 |
| 1 | hsa_776 | D07845 | 0 | hsa_2904 | D04918 | 0 | hsa_9254 | D08667 |
| 1 | hsa_776 | D07886 | 0 | hsa_2904 | D01856 | 0 | hsa_9254 | D00331 |
| 1 | hsa_776 | D07943 | 0 | hsa_2904 | D08935 | 0 | hsa_9254 | D07881 |
| 1 | hsa_776 | D08009 | 0 | hsa_2904 | D00808 | 0 | hsa_9254 | D02716 |
| 1 | hsa_776 | D08111 | 0 | hsa_2904 | D01479 | 0 | hsa_9254 | D08481 |
| 1 | hsa_776 | D08155 | 0 | hsa_2904 | D09917 | 0 | hsa_9254 | D04955 |
| 1 | hsa_776 | D08270 | 0 | hsa_2904 | D08669 | 0 | hsa_9254 | D07784 |
| 1 | hsa_776 | D08340 | 0 | hsa_2904 | D09215 | 0 | hsa_9254 | D04918 |
| 1 | hsa_776 | D08892 | 0 | hsa_2904 | D06517 | 0 | hsa_9254 | D07442 |
| 1 | hsa_776 | D09789 | 0 | hsa_2904 | D00380 | 0 | hsa_9254 | D02410 |
| 1 | hsa_778 | D00619 | 0 | hsa_2904 | D08669 | 0 | hsa_9254 | D01104 |
| 1 | hsa_778 | D00631 | 0 | hsa_2904 | D06392 | 0 | hsa_9254 | D08458 |
| 1 | hsa_778 | D01104 | 0 | hsa_2904 | D08435 | 0 | hsa_9254 | D04048 |
| 1 | hsa_778 | D01145 | 0 | hsa_2904 | D03547 | 0 | hsa_9254 | D00733 |
| 1 | hsa_778 | D01173 | 0 | hsa_2904 | D10370 | 0 | hsa_9254 | D08356 |
| 1 | hsa_778 | D01553 | 0 | hsa_2904 | D00064 | 0 | hsa_9254 | D04282 |
| 1 | hsa_778 | D01562 | 0 | hsa_2904 | D10112 | 0 | hsa_9254 | D08138 |
| 1 | hsa_778 | D01604 | 0 | hsa_2904 | D00399 | 0 | hsa_9254 | D08009 |
| 1 | hsa_778 | D01849 | 0 | hsa_2904 | D01785 | 0 | hsa_9254 | D01173 |
| 1 | hsa_778 | D01908 | 0 | hsa_2904 | D01856 | 0 | hsa_9254 | D02969 |
| 1 | hsa_778 | D01969 | 0 | hsa_2904 | D10111 | 0 | hsa_9254 | D00637 |
| 1 | hsa_778 | D02045 | 0 | hsa_2904 | D00375 | 0 | hsa_9254 | D10111 |
| 1 | hsa_778 | D02537 | 0 | hsa_2904 | D03547 | 0 | hsa_9254 | D08155 |
| 1 | hsa_778 | D03655 | 0 | hsa_2904 | D08060 | 0 | hsa_9254 | D05145 |
| 1 | hsa_778 | D03914 | 0 | hsa_2905 | D01479 | 0 | hsa_9254 | D10111 |
| 1 | hsa_778 | D04657 | 0 | hsa_2905 | D00550 | 0 | hsa_9254 | D01071 |
| 1 | hsa_778 | D05442 | 0 | hsa_2905 | D00418 | 0 | hsa_9254 | D01071 |
| 1 | hsa_778 | D07185 | 0 | hsa_2905 | D00303 | 0 | hsa_9254 | D08009 |
| 1 | hsa_778 | D07450 | 0 | hsa_2905 | D02347 | 0 | hsa_9254 | D02220 |
| 1 | hsa_778 | D07494 | 0 | hsa_2905 | D06172 | 0 | hsa_9254 | D08111 |
| 1 | hsa_778 | D07509 | 0 | hsa_2905 | D01303 | 0 | hsa_9254 | D07979 |
| 1 | hsa_778 | D07520 | 0 | hsa_2905 | D05482 | 0 | hsa_9254 | D02275 |
| 1 | hsa_778 | D07845 | 0 | hsa_2905 | D00636 | 0 | hsa_9254 | D00765 |
| 1 | hsa_778 | D07886 | 0 | hsa_2905 | D08215 | 0 | hsa_9254 | D08481 |
| 1 | hsa_778 | D07943 | 0 | hsa_2905 | D09918 | 0 | hsa_9254 | D04300 |
| 1 | hsa_778 | D08009 | 0 | hsa_2905 | D06653 | 0 | hsa_9254 | D00758 |
| 1 | hsa_778 | D08111 | 0 | hsa_2905 | D00336 | 0 | hsa_9254 | D04257 |
| 1 | hsa_778 | D08155 | 0 | hsa_2905 | D05077 | 0 | hsa_9254 | D00311 |
| 1 | hsa_778 | D08270 | 0 | hsa_2905 | D06172 | 0 | hsa_9254 | D01604 |
| 1 | hsa_778 | D08340 | 0 | hsa_2905 | D08669 | 0 | hsa_9254 | D01316 |
| 1 | hsa_778 | D08892 | 0 | hsa_2905 | D02103 | 0 | hsa_9254 | D01604 |
| 1 | hsa_778 | D09789 | 0 | hsa_2905 | D00375 | 0 | hsa_9254 | D03100 |
| 1 | hsa_779 | D00438 | 0 | hsa_2905 | D02220 | 0 | hsa_9254 | D05077 |
| 1 | hsa_779 | D00631 | 0 | hsa_2905 | D09917 | 0 | hsa_9254 | D03037 |
| 1 | hsa_779 | D01104 | 0 | hsa_2905 | D05077 | 0 | hsa_9254 | D02103 |
| 1 | hsa_779 | D01145 | 0 | hsa_2906 | D00386 | 0 | hsa_9254 | D00555 |
| 1 | hsa_779 | D01173 | 0 | hsa_2906 | D02096 | 0 | hsa_9254 | D07845 |
| 1 | hsa_779 | D01553 | 0 | hsa_2906 | D07845 | 0 | hsa_9254 | D02616 |
| 1 | hsa_779 | D01562 | 0 | hsa_2906 | D01145 | 0 | hsa_9254 | D03746 |
| 1 | hsa_779 | D01604 | 0 | hsa_2906 | D00011 | 0 | hsa_93589 | D00250 |
| 1 | hsa_779 | D01849 | 0 | hsa_2906 | D07993 | 0 | hsa_93589 | D10470 |
| 1 | hsa_779 | D01908 | 0 | hsa_2906 | D01849 | 0 | hsa_93589 | D01408 |
| 1 | hsa_779 | D02045 | 0 | hsa_2906 | D08217 | 0 | hsa_93589 | D02910 |
| 1 | hsa_779 | D02537 | 0 | hsa_2906 | D00533 | 0 | hsa_93589 | D08669 |
| 1 | hsa_779 | D03655 | 0 | hsa_2906 | D06392 | 0 | hsa_93589 | D01303 |
| 1 | hsa_779 | D03914 | 0 | hsa_2906 | D06392 | 0 | hsa_93589 | D05453 |
| 1 | hsa_779 | D04657 | 0 | hsa_2906 | D04048 | 0 | hsa_93589 | D00470 |
| 1 | hsa_779 | D05442 | 0 | hsa_2906 | D04849 | 0 | hsa_93589 | D03914 |
| 1 | hsa_779 | D07185 | 0 | hsa_2906 | D04849 | 0 | hsa_93589 | D00438 |
| 1 | hsa_779 | D07450 | 0 | hsa_2906 | D01303 | 0 | hsa_93589 | D02696 |
| 1 | hsa_779 | D07494 | 0 | hsa_2906 | D10693 | 0 | hsa_93589 | D01758 |
| 1 | hsa_779 | D07509 | 0 | hsa_2906 | D00808 | 0 | hsa_93589 | D10613 |
| 1 | hsa_779 | D07520 | 0 | hsa_2906 | D00064 | 0 | hsa_93589 | D01657 |
| 1 | hsa_779 | D07845 | 0 | hsa_2906 | D08448 | 0 | hsa_93589 | D07509 |
| 1 | hsa_779 | D07886 | 0 | hsa_2906 | D07678 | 0 | hsa_93589 | D00758 |
| 1 | hsa_779 | D07943 | 0 | hsa_2906 | D06653 | 0 | hsa_93589 | D00549 |
| 1 | hsa_779 | D08009 | 0 | hsa_2906 | D07442 | 0 | hsa_93589 | D07978 |
| 1 | hsa_779 | D08111 | 0 | hsa_2906 | D04955 | 0 | hsa_93589 | D00293 |
| 1 | hsa_779 | D08155 | 0 | hsa_2906 | D00553 | 0 | hsa_93589 | D02616 |
| 1 | hsa_779 | D08270 | 0 | hsa_2906 | D03732 | 0 | hsa_93589 | D08490 |
| 1 | hsa_779 | D08340 | 0 | hsa_2906 | D06653 | 0 | hsa_93589 | D10702 |
| 1 | hsa_779 | D08892 | 0 | hsa_2906 | D04657 | 0 | hsa_93589 | D00551 |
| 1 | hsa_779 | D09789 | 0 | hsa_2906 | D02274 | 0 | hsa_93589 | D00365 |
| 1 | hsa_781 | D02716 | 0 | hsa_3736 | D02594 | 0 | hsa_93589 | D00267 |
| 1 | hsa_781 | D09539 | 0 | hsa_3736 | D10626 | 0 | hsa_93589 | D07552 |
| 1 | hsa_79054 | D00064 | 0 | hsa_3736 | D02910 | 0 | hsa_93589 | D02096 |
| 1 | hsa_79054 | D04849 | 0 | hsa_3736 | D00329 | 0 | hsa_93589 | D06146 |
| 1 | hsa_79054 | D04918 | 0 | hsa_3736 | D07494 | 0 | hsa_93589 | D08987 |
| 1 | hsa_8001 | D00011 | 0 | hsa_3736 | D05447 | 0 | hsa_93589 | D08217 |
| 1 | hsa_8645 | D08422 | 0 | hsa_3736 | D02780 | 0 | hsa_93589 | D08459 |
| 1 | hsa_8911 | D00553 | 0 | hsa_3736 | D08458 | 0 | hsa_93589 | D09035 |
| 1 | hsa_8911 | D01243 | 0 | hsa_3736 | D05028 | 0 | hsa_93589 | D01657 |
| 1 | hsa_8911 | D01303 | 0 | hsa_3736 | D08048 | 0 | hsa_93589 | D00399 |
| 1 | hsa_8911 | D07971 | 0 | hsa_3736 | D08145 | 0 | hsa_93589 | D02347 |
| 1 | hsa_8911 | D08217 | 0 | hsa_3736 | D00500 | 0 | hsa_93589 | D00376 |
| 1 | hsa_8912 | D01303 | 0 | hsa_3736 | D03492 | 0 | hsa_93589 | D04728 |
| 1 | hsa_8912 | D07971 | 0 | hsa_3736 | D03037 | 0 | hsa_93589 | D01479 |
| 1 | hsa_8912 | D08217 | 0 | hsa_3736 | D01657 | 0 | hsa_93589 | D00533 |
| 1 | hsa_8913 | D01303 | 0 | hsa_3736 | D01564 | 0 | hsa_93589 | D08283 |
| 1 | hsa_8913 | D07971 | 0 | hsa_3736 | D04303 | 0 | hsa_93589 | D07999 |
| 1 | hsa_8913 | D08217 | 0 | hsa_3736 | D05442 | 0 | hsa_93589 | D00225 |
| 1 | hsa_8973 | D00612 | 0 | hsa_3736 | D00098 | 0 | hsa_93589 | D10112 |
| 1 | hsa_8973 | D02202 | 0 | hsa_3736 | D00553 | 0 | hsa_93589 | D07943 |
| 1 | hsa_8973 | D02364 | 0 | hsa_3736 | D00550 | 0 | hsa_93589 | D01245 |
| 1 | hsa_8973 | D08138 | 0 | hsa_3736 | D00555 | 0 | hsa_93589 | D04905 |
| 1 | hsa_8989 | D00098 | 0 | hsa_3736 | D08101 | 0 | hsa_93589 | D07999 |
| 1 | hsa_8989 | D06392 | 0 | hsa_3736 | D04955 | 0 | hsa_93589 | D05447 |
| 1 | hsa_9254 | D02716 | 0 | hsa_3736 | D01173 | 0 | hsa_93589 | D00555 |
| 1 | hsa_9254 | D09539 | 0 | hsa_3736 | D05442 | 0 | hsa_93589 | D01292 |
| 1 | hsa_93589 | D02716 | 0 | hsa_3736 | D07450 | 0 | hsa_93589 | D07552 |
| 1 | hsa_93589 | D09539 | 0 | hsa_3736 | D01479 | 0 | hsa_93589 | D00695 |

Table III The overall of drug-target interactions for GPCRs class on *Dataset2*

| **class** | **Targets** | **Drugs** | **class** | **Targets** | **Drugs** | **class** | **Targets** | **Drugs** |
| --- | --- | --- | --- | --- | --- | --- | --- | --- |
| 1 | hsa_80834 | D00025 | 0 | hsa_5734 | D01336 | 0 | hsa_150 | D00669 |
| 1 | hsa_134 | D00045 | 0 | hsa_3062 | D10424 | 0 | hsa_6752 | D02205 |
| 1 | hsa_2554 | D00058 | 0 | hsa_59340 | D07463 | 0 | hsa_2862 | D08687 |
| 1 | hsa_2555 | D00058 | 0 | hsa_4988 | D07125 | 0 | hsa_1131 | D01355 |
| 1 | hsa_2556 | D00058 | 0 | hsa_3274 | D00841 | 0 | hsa_2557 | D05008 |
| 1 | hsa_2557 | D00058 | 0 | hsa_4889 | D10170 | 0 | hsa_5143 | D07831 |
| 1 | hsa_2558 | D00058 | 0 | hsa_1814 | D06569 | 0 | hsa_10203 | D08595 |
| 1 | hsa_2559 | D00058 | 0 | hsa_10280 | D03212 | 0 | hsa_152 | D02238 |
| 1 | hsa_2560 | D00058 | 0 | hsa_5142 | D02681 | 0 | hsa_2557 | D06633 |
| 1 | hsa_2561 | D00058 | 0 | hsa_2567 | D02893 | 0 | hsa_554 | D03014 |
| 1 | hsa_2562 | D00058 | 0 | hsa_624 | D02208 | 0 | hsa_2562 | D01592 |
| 1 | hsa_2563 | D00058 | 0 | hsa_5144 | D01213 | 0 | hsa_4987 | D01399 |
| 1 | hsa_2564 | D00058 | 0 | hsa_3360 | D01955 | 0 | hsa_10800 | D10516 |
| 1 | hsa_2565 | D00058 | 0 | hsa_2798 | D08041 | 0 | hsa_1814 | D01101 |
| 1 | hsa_2566 | D00058 | 0 | hsa_552 | D08474 | 0 | hsa_6571 | D04501 |
| 1 | hsa_2567 | D00058 | 0 | hsa_155 | D08560 | 0 | hsa_5142 | D04888 |
| 1 | hsa_2568 | D00058 | 0 | hsa_5143 | D02676 | 0 | hsa_4543 | D00249 |
| 1 | hsa_55879 | D00058 | 0 | hsa_2554 | D05575 | 0 | hsa_624 | D00778 |
| 1 | hsa_146 | D00076 | 0 | hsa_1131 | D01347 | 0 | hsa_151 | D00485 |
| 1 | hsa_147 | D00076 | 0 | hsa_146 | D01898 | 0 | hsa_6344 | D10219 |
| 1 | hsa_148 | D00076 | 0 | hsa_2566 | D05429 | 0 | hsa_2561 | D04611 |
| 1 | hsa_5732 | D00079 | 0 | hsa_1129 | D02192 | 0 | hsa_170572 | D01362 |
| 1 | hsa_5737 | D00081 | 0 | hsa_3757 | D08353 | 0 | hsa_59340 | D01504 |
| 1 | hsa_5021 | D00089 | 0 | hsa_1909 | D03360 | 0 | hsa_846 | D08157 |
| 1 | hsa_146 | D00095 | 0 | hsa_9177 | D07312 | 0 | hsa_155 | D03267 |
| 1 | hsa_147 | D00095 | 0 | hsa_6532 | D10325 | 0 | hsa_64805 | D05667 |
| 1 | hsa_552 | D00101 | 0 | hsa_2554 | D04642 | 0 | hsa_3352 | D02901 |
| 1 | hsa_5739 | D00106 | 0 | hsa_1129 | D05649 | 0 | hsa_2862 | D01006 |
| 1 | hsa_146 | D00124 | 0 | hsa_55879 | D08182 | 0 | hsa_6532 | D07997 |
| 1 | hsa_147 | D00124 | 0 | hsa_2554 | D10478 | 0 | hsa_285242 | D08039 |
| 1 | hsa_148 | D00124 | 0 | hsa_1394 | D02101 | 0 | hsa_3360 | D05343 |
| 1 | hsa_1128 | D00138 | 0 | hsa_2561 | D04970 | 0 | hsa_6870 | D08067 |
| 1 | hsa_1129 | D00138 | 0 | hsa_1133 | D07809 | 0 | hsa_3357 | D02048 |
| 1 | hsa_1131 | D00138 | 0 | hsa_3357 | D05099 | 0 | hsa_5144 | D07837 |
| 1 | hsa_1132 | D00138 | 0 | hsa_2563 | D01386 | 0 | hsa_886 | D02109 |
| 1 | hsa_1133 | D00138 | 0 | hsa_3352 | D00076 | 0 | hsa_155 | D06574 |
| 1 | hsa_1128 | D00147 | 0 | hsa_3358 | D10170 | 0 | hsa_624 | D10099 |
| 1 | hsa_1129 | D00147 | 0 | hsa_799 | D00839 | 0 | hsa_3352 | D00778 |
| 1 | hsa_1131 | D00147 | 0 | hsa_1241 | D01477 | 0 | hsa_2567 | D07976 |
| 1 | hsa_1132 | D00147 | 0 | hsa_6752 | D00721 | 0 | hsa_5734 | D01006 |
| 1 | hsa_1133 | D00147 | 0 | hsa_3354 | D08595 | 0 | hsa_2566 | D01096 |
| 1 | hsa_7201 | D00176 | 0 | hsa_6608 | D03276 | 0 | hsa_153 | D07667 |
| 1 | hsa_4988 | D00195 | 0 | hsa_6755 | D01600 | 0 | hsa_155 | D07868 |
| 1 | hsa_1132 | D00232 | 0 | hsa_624 | D07890 | 0 | hsa_2562 | D04820 |
| 1 | hsa_1133 | D00232 | 0 | hsa_2566 | D08322 | 0 | hsa_5734 | D08122 |
| 1 | hsa_9568 | D00241 | 0 | hsa_10280 | D08362 | 0 | hsa_146 | D05690 |
| 1 | hsa_799 | D00249 | 0 | hsa_2915 | D10184 | 0 | hsa_185 | D02750 |
| 1 | hsa_3357 | D00270 | 0 | hsa_10203 | D08482 | 0 | hsa_2561 | D01006 |
| 1 | hsa_3358 | D00270 | 0 | hsa_2693 | D03290 | 0 | hsa_11255 | D02281 |
| 1 | hsa_552 | D00291 | 0 | hsa_3274 | D07946 | 0 | hsa_147 | D08026 |
| 1 | hsa_1813 | D00308 | 0 | hsa_5737 | D02682 | 0 | hsa_147 | D00663 |
| 1 | hsa_4988 | D00320 | 0 | hsa_6751 | D08598 | 0 | hsa_3757 | D01003 |
| 1 | hsa_552 | D00366 | 0 | hsa_4986 | D07459 | 0 | hsa_6608 | D01026 |
| 1 | hsa_134 | D00371 | 0 | hsa_4986 | D00847 | 0 | hsa_3757 | D04973 |
| 1 | hsa_1813 | D00373 | 0 | hsa_4988 | D08585 | 0 | hsa_7201 | D08482 |
| 1 | hsa_1813 | D00374 | 0 | hsa_285242 | D08093 | 0 | hsa_4988 | D00634 |
| 1 | hsa_153 | D00378 | 0 | hsa_11255 | D01794 | 0 | hsa_3354 | D05037 |
| 1 | hsa_1813 | D00390 | 0 | hsa_2915 | D05970 | 0 | hsa_6344 | D09990 |
| 1 | hsa_1131 | D00397 | 0 | hsa_55879 | D00106 | 0 | hsa_2864 | D02101 |
| 1 | hsa_1133 | D00397 | 0 | hsa_59340 | D04626 | 0 | hsa_3360 | D01952 |
| 1 | hsa_3356 | D00403 | 0 | hsa_846 | D00124 | 0 | hsa_6755 | D08234 |
| 1 | hsa_3357 | D00403 | 0 | hsa_5724 | D03361 | 0 | hsa_3352 | D09752 |
| 1 | hsa_3358 | D00403 | 0 | hsa_5143 | D05010 | 0 | hsa_5142 | D02004 |
| 1 | hsa_150 | D00405 | 0 | hsa_150 | D04970 | 0 | hsa_10203 | D08309 |
| 1 | hsa_151 | D00405 | 0 | hsa_6344 | D08923 | 0 | hsa_6532 | D01359 |
| 1 | hsa_152 | D00405 | 0 | hsa_3061 | D03359 | 0 | hsa_2554 | D02090 |
| 1 | hsa_3359 | D00456 | 0 | hsa_1133 | D10493 | 0 | hsa_3062 | D08091 |
| 1 | hsa_9177 | D00456 | 0 | hsa_55879 | D01355 | 0 | hsa_147 | D08685 |
| 1 | hsa_170572 | D00456 | 0 | hsa_2798 | D02109 | 0 | hsa_5139 | D10117 |
| 1 | hsa_200909 | D00456 | 0 | hsa_886 | D01385 | 0 | hsa_6344 | D03854 |
| 1 | hsa_285242 | D00456 | 0 | hsa_1814 | D08649 | 0 | hsa_9177 | D03725 |
| 1 | hsa_3356 | D00458 | 0 | hsa_5732 | D01635 | 0 | hsa_3362 | D01624 |
| 1 | hsa_1131 | D00465 | 0 | hsa_2912 | D04611 | 0 | hsa_6532 | D05682 |
| 1 | hsa_1813 | D00479 | 0 | hsa_9177 | D02022 | 0 | hsa_152 | D02250 |
| 1 | hsa_1128 | D00481 | 0 | hsa_3355 | D07451 | 0 | hsa_846 | D03622 |
| 1 | hsa_1129 | D00481 | 0 | hsa_3354 | D02095 | 0 | hsa_3274 | D09976 |
| 1 | hsa_1131 | D00481 | 0 | hsa_5144 | D07660 | 0 | hsa_170572 | D10181 |
| 1 | hsa_1132 | D00481 | 0 | hsa_2556 | D01028 | 0 | hsa_153 | D04098 |
| 1 | hsa_1133 | D00481 | 0 | hsa_154 | D09977 | 0 | hsa_4986 | D08988 |
| 1 | hsa_4988 | D00482 | 0 | hsa_3356 | D05731 | 0 | hsa_148 | D09349 |
| 1 | hsa_146 | D00485 | 0 | hsa_3360 | D05035 | 0 | hsa_146 | D01369 |
| 1 | hsa_147 | D00485 | 0 | hsa_200909 | D02071 | 0 | hsa_2555 | D01922 |
| 1 | hsa_148 | D00485 | 0 | hsa_2915 | D02765 | 0 | hsa_2568 | D00677 |
| 1 | hsa_4986 | D00498 | 0 | hsa_6752 | D08182 | 0 | hsa_5724 | D10147 |
| 1 | hsa_1812 | D00502 | 0 | hsa_2557 | D05738 | 0 | hsa_4987 | D03535 |
| 1 | hsa_146 | D00507 | 0 | hsa_6751 | D01887 | 0 | hsa_4157 | D08561 |
| 1 | hsa_147 | D00507 | 0 | hsa_147 | D06329 | 0 | hsa_2554 | D09954 |
| 1 | hsa_148 | D00507 | 0 | hsa_3356 | D02192 | 0 | hsa_3359 | D04492 |
| 1 | hsa_146 | D00511 | 0 | hsa_886 | D04701 | 0 | hsa_6755 | D07475 |
| 1 | hsa_147 | D00511 | 0 | hsa_1394 | D07718 | 0 | hsa_1909 | D03197 |
| 1 | hsa_148 | D00511 | 0 | hsa_2561 | D00757 | 0 | hsa_799 | D08685 |
| 1 | hsa_151 | D00514 | 0 | hsa_147 | D01879 | 0 | hsa_2557 | D02848 |
| 1 | hsa_152 | D00514 | 0 | hsa_1901 | D02846 | 0 | hsa_155 | D10220 |
| 1 | hsa_1131 | D00524 | 0 | hsa_1268 | D09697 | 0 | hsa_3354 | D09402 |
| 1 | hsa_1132 | D00524 | 0 | hsa_7201 | D00793 | 0 | hsa_5734 | D10441 |
| 1 | hsa_1133 | D00524 | 0 | hsa_3757 | D02035 | 0 | hsa_154 | D00997 |
| 1 | hsa_1129 | D00525 | 0 | hsa_55879 | D00686 | 0 | hsa_200909 | D02983 |
| 1 | hsa_1131 | D00525 | 0 | hsa_2557 | D08010 | 0 | hsa_10203 | D02384 |
| 1 | hsa_1132 | D00525 | 0 | hsa_6915 | D07682 | 0 | hsa_6571 | D10671 |
| 1 | hsa_1133 | D00525 | 0 | hsa_2561 | D08293 | 0 | hsa_5140 | D00847 |
| 1 | hsa_10800 | D00529 | 0 | hsa_1241 | D00820 | 0 | hsa_10203 | D06355 |
| 1 | hsa_1131 | D00540 | 0 | hsa_2864 | D04497 | 0 | hsa_6532 | D03854 |
| 1 | hsa_1813 | D00559 | 0 | hsa_6753 | D09964 | 0 | hsa_2568 | D00756 |
| 1 | hsa_3356 | D00561 | 0 | hsa_2563 | D03402 | 0 | hsa_3350 | D00291 |
| 1 | hsa_3357 | D00563 | 0 | hsa_7201 | D00819 | 0 | hsa_5724 | D03051 |
| 1 | hsa_2798 | D00573 | 0 | hsa_3062 | D07171 | 0 | hsa_3362 | D08384 |
| 1 | hsa_153 | D00597 | 0 | hsa_4157 | D08687 | 0 | hsa_154 | D04788 |
| 1 | hsa_153 | D00599 | 0 | hsa_4889 | D04057 | 0 | hsa_185 | D02396 |
| 1 | hsa_146 | D00600 | 0 | hsa_2862 | D06574 | 0 | hsa_59340 | D00634 |
| 1 | hsa_147 | D00600 | 0 | hsa_886 | D09397 | 0 | hsa_5139 | D09752 |
| 1 | hsa_148 | D00600 | 0 | hsa_5732 | D04625 | 0 | hsa_552 | D01307 |
| 1 | hsa_153 | D00602 | 0 | hsa_3355 | D07861 | 0 | hsa_3352 | D09991 |
| 1 | hsa_153 | D00603 | 0 | hsa_10800 | D05939 | 0 | hsa_3351 | D08426 |
| 1 | hsa_150 | D00605 | 0 | hsa_150 | D03002 | 0 | hsa_59340 | D08226 |
| 1 | hsa_151 | D00605 | 0 | hsa_2149 | D07181 | 0 | hsa_5140 | D10677 |
| 1 | hsa_152 | D00605 | 0 | hsa_5141 | D02236 | 0 | hsa_624 | D03502 |
| 1 | hsa_146 | D00608 | 0 | hsa_1812 | D07603 | 0 | hsa_2567 | D00835 |
| 1 | hsa_147 | D00608 | 0 | hsa_1131 | D00597 | 0 | hsa_135 | D02151 |
| 1 | hsa_148 | D00608 | 0 | hsa_151 | D00511 | 0 | hsa_1132 | D07990 |
| 1 | hsa_148 | D00610 | 0 | hsa_6754 | D05677 | 0 | hsa_2915 | D05127 |
| 1 | hsa_147 | D00610 | 0 | hsa_3354 | D03502 | 0 | hsa_3358 | D07489 |
| 1 | hsa_146 | D00610 | 0 | hsa_4157 | D01385 | 0 | hsa_146 | D06268 |
| 1 | hsa_185 | D00626 | 0 | hsa_6532 | D08355 | 0 | hsa_5143 | D02363 |
| 1 | hsa_1812 | D00633 | 0 | hsa_2564 | D09610 | 0 | hsa_2554 | D02739 |
| 1 | hsa_153 | D00634 | 0 | hsa_154 | D08986 | 0 | hsa_4985 | D00669 |
| 1 | hsa_153 | D00644 | 0 | hsa_3061 | D10147 | 0 | hsa_3356 | D07522 |
| 1 | hsa_1131 | D00646 | 0 | hsa_7201 | D06293 | 0 | hsa_2562 | D10391 |
| 1 | hsa_3269 | D00659 | 0 | hsa_3350 | D08594 | 0 | hsa_10203 | D09765 |
| 1 | hsa_1128 | D00661 | 0 | hsa_5729 | D08054 | 0 | hsa_2568 | D09391 |
| 1 | hsa_1131 | D00661 | 0 | hsa_5142 | D02590 | 0 | hsa_5021 | D09791 |
| 1 | hsa_3269 | D00662 | 0 | hsa_7201 | D07684 | 0 | hsa_6570 | D08226 |
| 1 | hsa_3269 | D00663 | 0 | hsa_2563 | D08449 | 0 | hsa_2557 | D10677 |
| 1 | hsa_3269 | D00664 | 0 | hsa_6751 | D02090 | 0 | hsa_1128 | D02663 |
| 1 | hsa_3269 | D00668 | 0 | hsa_6754 | D07406 | 0 | hsa_55879 | D08560 |
| 1 | hsa_3269 | D00669 | 0 | hsa_2862 | D03953 | 0 | hsa_1131 | D03399 |
| 1 | hsa_3269 | D00671 | 0 | hsa_152 | D04500 | 0 | hsa_3358 | D08525 |
| 1 | hsa_3269 | D00672 | 0 | hsa_3362 | D00847 | 0 | hsa_3357 | D07459 |
| 1 | hsa_3350 | D00674 | 0 | hsa_2562 | D01007 | 0 | hsa_886 | D10747 |
| 1 | hsa_3359 | D00677 | 0 | hsa_6344 | D01500 | 0 | hsa_135 | D01006 |
| 1 | hsa_9177 | D00677 | 0 | hsa_1132 | D09610 | 0 | hsa_3357 | D02725 |
| 1 | hsa_170572 | D00677 | 0 | hsa_10280 | D05677 | 0 | hsa_5737 | D00820 |
| 1 | hsa_200909 | D00677 | 0 | hsa_2862 | D01263 | 0 | hsa_5729 | D01830 |
| 1 | hsa_285242 | D00677 | 0 | hsa_55879 | D09205 | 0 | hsa_170572 | D02227 |
| 1 | hsa_3359 | D00678 | 0 | hsa_43 | D00839 | 0 | hsa_799 | D06659 |
| 1 | hsa_9177 | D00678 | 0 | hsa_1133 | D08384 | 0 | hsa_59340 | D08457 |
| 1 | hsa_170572 | D00678 | 0 | hsa_4987 | D08614 | 0 | hsa_2913 | D04405 |
| 1 | hsa_200909 | D00678 | 0 | hsa_2563 | D04717 | 0 | hsa_200909 | D06569 |
| 1 | hsa_285242 | D00678 | 0 | hsa_6608 | D06280 | 0 | hsa_624 | D02151 |
| 1 | hsa_3350 | D00679 | 0 | hsa_1241 | D06659 | 0 | hsa_5737 | D01044 |
| 1 | hsa_3350 | D00680 | 0 | hsa_3360 | D06328 | 0 | hsa_3358 | D03704 |
| 1 | hsa_3351 | D00680 | 0 | hsa_1128 | D02192 | 0 | hsa_5139 | D01177 |
| 1 | hsa_3352 | D00680 | 0 | hsa_56413 | D00681 | 0 | hsa_2556 | D03535 |
| 1 | hsa_3354 | D00680 | 0 | hsa_3350 | D03177 | 0 | hsa_3359 | D08648 |
| 1 | hsa_3355 | D00680 | 0 | hsa_6753 | D07286 | 0 | hsa_5737 | D04264 |
| 1 | hsa_3350 | D00681 | 0 | hsa_4987 | D00481 | 0 | hsa_846 | D01481 |
| 1 | hsa_5737 | D00682 | 0 | hsa_9568 | D10170 | 0 | hsa_1813 | D03506 |
| 1 | hsa_154 | D00685 | 0 | hsa_4986 | D05111 | 0 | hsa_1814 | D04517 |
| 1 | hsa_154 | D00686 | 0 | hsa_3359 | D08183 | 0 | hsa_2565 | D07905 |
| 1 | hsa_3350 | D00702 | 0 | hsa_134 | D10345 | 0 | hsa_185 | D04625 |
| 1 | hsa_1129 | D00715 | 0 | hsa_185 | D04057 | 0 | hsa_3362 | D01445 |
| 1 | hsa_1131 | D00715 | 0 | hsa_1128 | D08054 | 0 | hsa_4987 | D03102 |
| 1 | hsa_1132 | D00715 | 0 | hsa_2904 | D04883 | 0 | hsa_2564 | D07226 |
| 1 | hsa_1133 | D00715 | 0 | hsa_2864 | D07617 | 0 | hsa_2149 | D08035 |
| 1 | hsa_1128 | D00717 | 0 | hsa_1813 | D01021 | 0 | hsa_3358 | D02093 |
| 1 | hsa_1128 | D00719 | 0 | hsa_5142 | D03267 | 0 | hsa_5142 | D10671 |
| 1 | hsa_1129 | D00719 | 0 | hsa_1901 | D09699 | 0 | hsa_5729 | D01008 |
| 1 | hsa_1131 | D00719 | 0 | hsa_3354 | D04889 | 0 | hsa_148 | D08865 |
| 1 | hsa_1132 | D00719 | 0 | hsa_2912 | D07937 | 0 | hsa_5139 | D05008 |
| 1 | hsa_1133 | D00719 | 0 | hsa_3357 | D01201 | 0 | hsa_10203 | D07946 |
| 1 | hsa_1128 | D00720 | 0 | hsa_3350 | D05037 | 0 | hsa_5141 | D08940 |
| 1 | hsa_1129 | D00720 | 0 | hsa_2556 | D10199 | 0 | hsa_56413 | D01861 |
| 1 | hsa_1131 | D00720 | 0 | hsa_5737 | D01451 | 0 | hsa_155 | D01448 |
| 1 | hsa_1132 | D00720 | 0 | hsa_6751 | D03518 | 0 | hsa_1812 | D06157 |
| 1 | hsa_1133 | D00720 | 0 | hsa_1129 | D07937 | 0 | hsa_4543 | D01773 |
| 1 | hsa_1128 | D00721 | 0 | hsa_3355 | D01019 | 0 | hsa_6869 | D08626 |
| 1 | hsa_1129 | D00721 | 0 | hsa_1813 | D00663 | 0 | hsa_2555 | D09981 |
| 1 | hsa_1131 | D00721 | 0 | hsa_3355 | D08901 | 0 | hsa_55879 | D08284 |
| 1 | hsa_1132 | D00721 | 0 | hsa_2561 | D03014 | 0 | hsa_6608 | D02104 |
| 1 | hsa_1133 | D00721 | 0 | hsa_2913 | D01386 | 0 | hsa_1901 | D04710 |
| 1 | hsa_1128 | D00722 | 0 | hsa_1909 | D03891 | 0 | hsa_2566 | D05677 |
| 1 | hsa_1129 | D00722 | 0 | hsa_5140 | D02212 | 0 | hsa_1813 | D01462 |
| 1 | hsa_1131 | D00722 | 0 | hsa_6608 | D01438 | 0 | hsa_886 | D04157 |
| 1 | hsa_1128 | D00723 | 0 | hsa_4889 | D01177 | 0 | hsa_153 | D07402 |
| 1 | hsa_1129 | D00723 | 0 | hsa_153 | D03854 | 0 | hsa_5737 | D10185 |
| 1 | hsa_1131 | D00723 | 0 | hsa_10800 | D00674 | 0 | hsa_2556 | D02599 |
| 1 | hsa_1132 | D00723 | 0 | hsa_146 | D00799 | 0 | hsa_2555 | D08323 |
| 1 | hsa_1133 | D00723 | 0 | hsa_2561 | D09732 | 0 | hsa_1901 | D02834 |
| 1 | hsa_3359 | D00725 | 0 | hsa_150 | D10729 | 0 | hsa_3360 | D02645 |
| 1 | hsa_9177 | D00725 | 0 | hsa_552 | D00729 | 0 | hsa_1812 | D01887 |
| 1 | hsa_170572 | D00725 | 0 | hsa_59340 | D07905 | 0 | hsa_1394 | D04531 |
| 1 | hsa_200909 | D00725 | 0 | hsa_3351 | D01023 | 0 | hsa_2693 | D05699 |
| 1 | hsa_285242 | D00725 | 0 | hsa_5732 | D02250 | 0 | hsa_6608 | D03693 |
| 1 | hsa_4988 | D00729 | 0 | hsa_2561 | D00291 | 0 | hsa_56413 | D03891 |
| 1 | hsa_146 | D00743 | 0 | hsa_5140 | D07228 | 0 | hsa_6869 | D07534 |
| 1 | hsa_147 | D00743 | 0 | hsa_552 | D02950 | 0 | hsa_6755 | D02710 |
| 1 | hsa_148 | D00743 | 0 | hsa_5724 | D07076 | 0 | hsa_6608 | D09344 |
| 1 | hsa_146 | D00756 | 0 | hsa_285242 | D00663 | 0 | hsa_1131 | D01648 |
| 1 | hsa_147 | D00756 | 0 | hsa_3757 | D04126 | 0 | hsa_6754 | D01654 |
| 1 | hsa_148 | D00756 | 0 | hsa_2561 | D01343 | 0 | hsa_6870 | D10740 |
| 1 | hsa_146 | D00757 | 0 | hsa_3350 | D07718 | 0 | hsa_1268 | D02774 |
| 1 | hsa_147 | D00757 | 0 | hsa_80834 | D08123 | 0 | hsa_1133 | D08205 |
| 1 | hsa_148 | D00757 | 0 | hsa_2864 | D01635 | 0 | hsa_1814 | D05699 |
| 1 | hsa_1128 | D00774 | 0 | hsa_2550 | D08090 | 0 | hsa_3352 | D06268 |
| 1 | hsa_1129 | D00774 | 0 | hsa_147 | D10129 | 0 | hsa_5734 | D01600 |
| 1 | hsa_1131 | D00774 | 0 | hsa_4157 | D08092 | 0 | hsa_4157 | D01182 |
| 1 | hsa_1132 | D00774 | 0 | hsa_152 | D07129 | 0 | hsa_153 | D09964 |
| 1 | hsa_1133 | D00774 | 0 | hsa_9177 | D08305 | 0 | hsa_7201 | D02702 |
| 1 | hsa_150 | D00776 | 0 | hsa_2566 | D05127 | 0 | hsa_150 | D07349 |
| 1 | hsa_151 | D00776 | 0 | hsa_4889 | D03783 | 0 | hsa_1812 | D04611 |
| 1 | hsa_152 | D00776 | 0 | hsa_1814 | D10135 | 0 | hsa_64805 | D01698 |
| 1 | hsa_1128 | D00778 | 0 | hsa_6753 | D01017 | 0 | hsa_1132 | D01648 |
| 1 | hsa_1129 | D00778 | 0 | hsa_6570 | D01182 | 0 | hsa_3356 | D04710 |
| 1 | hsa_1131 | D00778 | 0 | hsa_5737 | D03618 | 0 | hsa_5140 | D10364 |
| 1 | hsa_1132 | D00778 | 0 | hsa_10800 | D07402 | 0 | hsa_6570 | D08241 |
| 1 | hsa_1133 | D00778 | 0 | hsa_3350 | D07551 | 0 | hsa_1909 | D08362 |
| 1 | hsa_1131 | D00779 | 0 | hsa_2693 | D03177 | 0 | hsa_43 | D04517 |
| 1 | hsa_1132 | D00779 | 0 | hsa_3357 | D03704 | 0 | hsa_886 | D02208 |
| 1 | hsa_1133 | D00779 | 0 | hsa_2560 | D01481 | 0 | hsa_1814 | D08106 |
| 1 | hsa_1128 | D00782 | 0 | hsa_3757 | D04641 | 0 | hsa_846 | D00291 |
| 1 | hsa_1129 | D00782 | 0 | hsa_6751 | D05732 | 0 | hsa_150 | D07226 |
| 1 | hsa_1131 | D00782 | 0 | hsa_3359 | D03014 | 0 | hsa_6570 | D02104 |
| 1 | hsa_1132 | D00782 | 0 | hsa_2564 | D00743 | 0 | hsa_5731 | D00672 |
| 1 | hsa_1133 | D00782 | 0 | hsa_2556 | D04517 | 0 | hsa_6571 | D00405 |
| 1 | hsa_1813 | D00784 | 0 | hsa_2566 | D08384 | 0 | hsa_6571 | D01691 |
| 1 | hsa_1128 | D00787 | 0 | hsa_2557 | D01123 | 0 | hsa_200909 | D07684 |
| 1 | hsa_1129 | D00787 | 0 | hsa_2913 | D04155 | 0 | hsa_55879 | D08093 |
| 1 | hsa_1131 | D00787 | 0 | hsa_2566 | D07557 | 0 | hsa_3352 | D04973 |
| 1 | hsa_1132 | D00787 | 0 | hsa_134 | D08376 | 0 | hsa_3354 | D03170 |
| 1 | hsa_1133 | D00787 | 0 | hsa_3757 | D01861 | 0 | hsa_3356 | D02091 |
| 1 | hsa_3356 | D00789 | 0 | hsa_2862 | D05339 | 0 | hsa_170572 | D05366 |
| 1 | hsa_3357 | D00789 | 0 | hsa_3354 | D00076 | 0 | hsa_56413 | D07713 |
| 1 | hsa_3358 | D00789 | 0 | hsa_1813 | D00723 | 0 | hsa_4157 | D01773 |
| 1 | hsa_1813 | D00791 | 0 | hsa_2564 | D00797 | 0 | hsa_2566 | D08494 |
| 1 | hsa_1813 | D00792 | 0 | hsa_2693 | D02860 | 0 | hsa_5724 | D06495 |
| 1 | hsa_1813 | D00793 | 0 | hsa_135 | D05941 | 0 | hsa_2567 | D08691 |
| 1 | hsa_1813 | D00794 | 0 | hsa_799 | D01438 | 0 | hsa_5731 | D06660 |
| 1 | hsa_1813 | D00796 | 0 | hsa_6344 | D10219 | 0 | hsa_2554 | D07990 |
| 1 | hsa_1813 | D00797 | 0 | hsa_4986 | D02343 | 0 | hsa_552 | D01003 |
| 1 | hsa_1813 | D00798 | 0 | hsa_64805 | D01007 | 0 | hsa_3757 | D07958 |
| 1 | hsa_1813 | D00799 | 0 | hsa_5732 | D06274 | 0 | hsa_2912 | D10006 |
| 1 | hsa_1813 | D00800 | 0 | hsa_6870 | D01123 | 0 | hsa_6915 | D08241 |
| 1 | hsa_3356 | D00819 | 0 | hsa_3061 | D08121 | 0 | hsa_80834 | D01898 |
| 1 | hsa_6532 | D00820 | 0 | hsa_55879 | D01383 | 0 | hsa_1128 | D00458 |
| 1 | hsa_4988 | D00835 | 0 | hsa_2912 | D04155 | 0 | hsa_200909 | D10001 |
| 1 | hsa_4988 | D00839 | 0 | hsa_3350 | D08639 | 0 | hsa_155 | D06394 |
| 1 | hsa_4988 | D00840 | 0 | hsa_10800 | D03449 | 0 | hsa_151 | D02234 |
| 1 | hsa_4988 | D00841 | 0 | hsa_200909 | D07461 | 0 | hsa_10800 | D08864 |
| 1 | hsa_4988 | D00842 | 0 | hsa_3061 | D05996 | 0 | hsa_4987 | D08685 |
| 1 | hsa_4986 | D00843 | 0 | hsa_2562 | D08090 | 0 | hsa_3356 | D04788 |
| 1 | hsa_4988 | D00844 | 0 | hsa_5139 | D06274 | 0 | hsa_155 | D04314 |
| 1 | hsa_4988 | D00847 | 0 | hsa_2555 | D02995 | 0 | hsa_799 | D02632 |
| 1 | hsa_2798 | D00988 | 0 | hsa_1129 | D09607 | 0 | hsa_3757 | D04649 |
| 1 | hsa_2798 | D00989 | 0 | hsa_2912 | D08594 | 0 | hsa_2912 | D00843 |
| 1 | hsa_2798 | D00990 | 0 | hsa_2555 | D00626 | 0 | hsa_1815 | D06672 |
| 1 | hsa_146 | D00997 | 0 | hsa_6751 | D01336 | 0 | hsa_4543 | D01383 |
| 1 | hsa_147 | D00997 | 0 | hsa_799 | D01213 | 0 | hsa_2912 | D05053 |
| 1 | hsa_148 | D00997 | 0 | hsa_799 | D02950 | 0 | hsa_9568 | D08226 |
| 1 | hsa_1128 | D00999 | 0 | hsa_6571 | D01469 | 0 | hsa_846 | D10740 |
| 1 | hsa_1129 | D00999 | 0 | hsa_5141 | D01263 | 0 | hsa_9568 | D01463 |
| 1 | hsa_1131 | D00999 | 0 | hsa_11255 | D01021 | 0 | hsa_3350 | D08067 |
| 1 | hsa_1132 | D00999 | 0 | hsa_6570 | D00646 | 0 | hsa_6870 | D01478 |
| 1 | hsa_1133 | D00999 | 0 | hsa_552 | D00076 | 0 | hsa_3357 | D00796 |
| 1 | hsa_1128 | D01000 | 0 | hsa_2557 | D10326 | 0 | hsa_150 | D02774 |
| 1 | hsa_1129 | D01000 | 0 | hsa_134 | D07831 | 0 | hsa_147 | D01939 |
| 1 | hsa_1131 | D01000 | 0 | hsa_64805 | D01522 | 0 | hsa_3357 | D01028 |
| 1 | hsa_1132 | D01000 | 0 | hsa_2566 | D01887 | 0 | hsa_10203 | D08132 |
| 1 | hsa_1133 | D01000 | 0 | hsa_151 | D00195 | 0 | hsa_1813 | D03264 |
| 1 | hsa_1128 | D01002 | 0 | hsa_5724 | D08092 | 0 | hsa_2557 | D00176 |
| 1 | hsa_1129 | D01002 | 0 | hsa_2564 | D10128 | 0 | hsa_152 | D02236 |
| 1 | hsa_1131 | D01002 | 0 | hsa_1394 | D08236 | 0 | hsa_4986 | D08395 |
| 1 | hsa_1132 | D01002 | 0 | hsa_146 | D03051 | 0 | hsa_2565 | D08626 |
| 1 | hsa_1133 | D01002 | 0 | hsa_1129 | D07259 | 0 | hsa_2149 | D04157 |
| 1 | hsa_1128 | D01003 | 0 | hsa_552 | D00840 | 0 | hsa_6869 | D07997 |
| 1 | hsa_1129 | D01003 | 0 | hsa_4987 | D01386 | 0 | hsa_6755 | D03008 |
| 1 | hsa_1131 | D01003 | 0 | hsa_5734 | D01795 | 0 | hsa_43 | D07537 |
| 1 | hsa_1132 | D01003 | 0 | hsa_10280 | D00147 | 0 | hsa_3360 | D01263 |
| 1 | hsa_1133 | D01003 | 0 | hsa_552 | D09400 | 0 | hsa_3357 | D02983 |
| 1 | hsa_1128 | D01004 | 0 | hsa_3757 | D06613 | 0 | hsa_10800 | D06632 |
| 1 | hsa_1129 | D01004 | 0 | hsa_3062 | D10628 | 0 | hsa_3757 | D07125 |
| 1 | hsa_1131 | D01004 | 0 | hsa_151 | D08585 | 0 | hsa_3360 | D02388 |
| 1 | hsa_1132 | D01004 | 0 | hsa_2862 | D08397 | 0 | hsa_5141 | D02417 |
| 1 | hsa_1133 | D01004 | 0 | hsa_155 | D08966 | 0 | hsa_4543 | D03197 |
| 1 | hsa_1128 | D01005 | 0 | hsa_80834 | D09400 | 0 | hsa_4543 | D07148 |
| 1 | hsa_1129 | D01005 | 0 | hsa_185 | D01869 | 0 | hsa_64805 | D08216 |
| 1 | hsa_1131 | D01005 | 0 | hsa_6869 | D03814 | 0 | hsa_886 | D02622 |
| 1 | hsa_1132 | D01005 | 0 | hsa_7201 | D03693 | 0 | hsa_153 | D10742 |
| 1 | hsa_1133 | D01005 | 0 | hsa_3757 | D06622 | 0 | hsa_10280 | D01891 |
| 1 | hsa_1131 | D01006 | 0 | hsa_170572 | D07099 | 0 | hsa_3061 | D08443 |
| 1 | hsa_1128 | D01007 | 0 | hsa_155 | D00677 | 0 | hsa_6344 | D05085 |
| 1 | hsa_1129 | D01007 | 0 | hsa_6751 | D01025 | 0 | hsa_5731 | D03195 |
| 1 | hsa_1131 | D01007 | 0 | hsa_56413 | D05804 | 0 | hsa_2913 | D08246 |
| 1 | hsa_1132 | D01007 | 0 | hsa_64805 | D07538 | 0 | hsa_1129 | D07904 |
| 1 | hsa_1133 | D01007 | 0 | hsa_80834 | D00664 | 0 | hsa_3350 | D09867 |
| 1 | hsa_150 | D01008 | 0 | hsa_147 | D08183 | 0 | hsa_5143 | D07682 |
| 1 | hsa_151 | D01008 | 0 | hsa_1901 | D04889 | 0 | hsa_846 | D01635 |
| 1 | hsa_152 | D01008 | 0 | hsa_10203 | D05015 | 0 | hsa_2149 | D02688 |
| 1 | hsa_146 | D01017 | 0 | hsa_7201 | D05306 | 0 | hsa_6869 | D05015 |
| 1 | hsa_147 | D01017 | 0 | hsa_1131 | D06569 | 0 | hsa_5732 | D08144 |
| 1 | hsa_148 | D01017 | 0 | hsa_10203 | D08343 | 0 | hsa_11255 | D10648 |
| 1 | hsa_146 | D01019 | 0 | hsa_10203 | D03518 | 0 | hsa_554 | D01741 |
| 1 | hsa_147 | D01019 | 0 | hsa_151 | D10478 | 0 | hsa_10800 | D02765 |
| 1 | hsa_148 | D01019 | 0 | hsa_11255 | D02048 | 0 | hsa_5021 | D05891 |
| 1 | hsa_146 | D01021 | 0 | hsa_148 | D00819 | 0 | hsa_59340 | D08031 |
| 1 | hsa_147 | D01021 | 0 | hsa_1815 | D07182 | 0 | hsa_4985 | D00776 |
| 1 | hsa_148 | D01021 | 0 | hsa_10800 | D05230 | 0 | hsa_2561 | D02108 |
| 1 | hsa_146 | D01023 | 0 | hsa_1131 | D09992 | 0 | hsa_11255 | D07181 |
| 1 | hsa_147 | D01023 | 0 | hsa_7201 | D07125 | 0 | hsa_2149 | D08099 |
| 1 | hsa_148 | D01023 | 0 | hsa_1815 | D07286 | 0 | hsa_6532 | D08983 |
| 1 | hsa_153 | D01025 | 0 | hsa_3359 | D02238 | 0 | hsa_4985 | D02829 |
| 1 | hsa_3757 | D01026 | 0 | hsa_4157 | D07838 | 0 | hsa_6915 | D03274 |
| 1 | hsa_64805 | D01028 | 0 | hsa_6869 | D04680 | 0 | hsa_1133 | D00847 |
| 1 | hsa_1813 | D01044 | 0 | hsa_2560 | D07538 | 0 | hsa_56413 | D10663 |
| 1 | hsa_1813 | D01051 | 0 | hsa_3357 | D07887 | 0 | hsa_285242 | D01795 |
| 1 | hsa_1128 | D01077 | 0 | hsa_3062 | D04361 | 0 | hsa_1268 | D01958 |
| 1 | hsa_1129 | D01077 | 0 | hsa_285242 | D08182 | 0 | hsa_1132 | D05677 |
| 1 | hsa_1131 | D01077 | 0 | hsa_846 | D00373 | 0 | hsa_3757 | D06213 |
| 1 | hsa_1132 | D01077 | 0 | hsa_1814 | D10424 | 0 | hsa_3350 | D05781 |
| 1 | hsa_1133 | D01077 | 0 | hsa_2862 | D00789 | 0 | hsa_3360 | D08768 |
| 1 | hsa_80834 | D01085 | 0 | hsa_2798 | D09976 | 0 | hsa_3359 | D08382 |
| 1 | hsa_3269 | D01096 | 0 | hsa_6753 | D05206 | 0 | hsa_6344 | D08449 |
| 1 | hsa_1813 | D01101 | 0 | hsa_80834 | D02104 | 0 | hsa_80834 | D04098 |
| 1 | hsa_1129 | D01103 | 0 | hsa_624 | D02249 | 0 | hsa_5729 | D06618 |
| 1 | hsa_1131 | D01103 | 0 | hsa_4543 | D03860 | 0 | hsa_6753 | D04074 |
| 1 | hsa_1132 | D01103 | 0 | hsa_200909 | D04264 | 0 | hsa_1812 | D08571 |
| 1 | hsa_1133 | D01103 | 0 | hsa_6915 | D10129 | 0 | hsa_5731 | D08047 |
| 1 | hsa_1813 | D01105 | 0 | hsa_2562 | D08991 | 0 | hsa_3358 | D08220 |
| 1 | hsa_3269 | D01117 | 0 | hsa_2864 | D09319 | 0 | hsa_3355 | D00729 |
| 1 | hsa_1129 | D01118 | 0 | hsa_148 | D00089 | 0 | hsa_3362 | D07718 |
| 1 | hsa_1131 | D01118 | 0 | hsa_2563 | D01987 | 0 | hsa_6753 | D08093 |
| 1 | hsa_1132 | D01118 | 0 | hsa_6915 | D05742 | 0 | hsa_2558 | D01006 |
| 1 | hsa_1133 | D01118 | 0 | hsa_5139 | D10129 | 0 | hsa_146 | D10020 |
| 1 | hsa_6915 | D01123 | 0 | hsa_2566 | D07181 | 0 | hsa_155 | D00602 |
| 1 | hsa_6915 | D01128 | 0 | hsa_147 | D07718 | 0 | hsa_552 | D10710 |
| 1 | hsa_3274 | D01131 | 0 | hsa_2566 | D01315 | 0 | hsa_155 | D00671 |
| 1 | hsa_3269 | D01143 | 0 | hsa_2560 | D00841 | 0 | hsa_4985 | D04533 |
| 1 | hsa_1131 | D01148 | 0 | hsa_150 | D02632 | 0 | hsa_1813 | D08856 |
| 1 | hsa_3350 | D01163 | 0 | hsa_2550 | D02670 | 0 | hsa_3354 | D04264 |
| 1 | hsa_3351 | D01163 | 0 | hsa_552 | D00672 | 0 | hsa_4988 | D00374 |
| 1 | hsa_3352 | D01163 | 0 | hsa_5729 | D10171 | 0 | hsa_846 | D07821 |
| 1 | hsa_3354 | D01163 | 0 | hsa_135 | D02417 | 0 | hsa_2864 | D01148 |
| 1 | hsa_3355 | D01163 | 0 | hsa_5140 | D01269 | 0 | hsa_5731 | D09991 |
| 1 | hsa_1128 | D01165 | 0 | hsa_6753 | D08366 | 0 | hsa_3350 | D02205 |
| 1 | hsa_1129 | D01165 | 0 | hsa_2564 | D07860 | 0 | hsa_6532 | D01939 |
| 1 | hsa_1131 | D01165 | 0 | hsa_2693 | D05597 | 0 | hsa_1901 | D05690 |
| 1 | hsa_1132 | D01165 | 0 | hsa_5734 | D07904 | 0 | hsa_2913 | D08692 |
| 1 | hsa_1133 | D01165 | 0 | hsa_6532 | D09953 | 0 | hsa_2915 | D03221 |
| 1 | hsa_3269 | D01172 | 0 | hsa_4157 | D10145 | 0 | hsa_6755 | D01987 |
| 1 | hsa_3269 | D01174 | 0 | hsa_3354 | D02939 | 0 | hsa_5143 | D07563 |
| 1 | hsa_1128 | D01175 | 0 | hsa_9177 | D03214 | 0 | hsa_135 | D09732 |
| 1 | hsa_1129 | D01175 | 0 | hsa_147 | D09008 | 0 | hsa_2558 | D04724 |
| 1 | hsa_1131 | D01175 | 0 | hsa_146 | D05742 | 0 | hsa_10203 | D01500 |
| 1 | hsa_1132 | D01175 | 0 | hsa_554 | D07803 | 0 | hsa_2556 | D01177 |
| 1 | hsa_1133 | D01175 | 0 | hsa_2915 | D10147 | 0 | hsa_3351 | D07862 |
| 1 | hsa_3356 | D01176 | 0 | hsa_5734 | D02108 | 0 | hsa_10203 | D00659 |
| 1 | hsa_4988 | D01177 | 0 | hsa_4889 | D04501 | 0 | hsa_2798 | D07904 |
| 1 | hsa_153 | D01182 | 0 | hsa_154 | D00702 | 0 | hsa_4889 | D07102 |
| 1 | hsa_5739 | D01188 | 0 | hsa_153 | D00106 | 0 | hsa_147 | D03415 |
| 1 | hsa_3269 | D01192 | 0 | hsa_6344 | D04492 | 0 | hsa_2560 | D01307 |
| 1 | hsa_3274 | D01193 | 0 | hsa_2555 | D00686 | 0 | hsa_2558 | D07076 |
| 1 | hsa_1128 | D01201 | 0 | hsa_2862 | D04970 | 0 | hsa_200909 | D05939 |
| 1 | hsa_1129 | D01201 | 0 | hsa_2912 | D05230 | 0 | hsa_4988 | D10219 |
| 1 | hsa_1131 | D01201 | 0 | hsa_2149 | D00997 | 0 | hsa_153 | D06622 |
| 1 | hsa_1132 | D01201 | 0 | hsa_2561 | D02878 | 0 | hsa_2558 | D00124 |
| 1 | hsa_1133 | D01201 | 0 | hsa_6570 | D02613 | 0 | hsa_1901 | D01952 |
| 1 | hsa_185 | D01204 | 0 | hsa_2565 | D10562 | 0 | hsa_2862 | D02613 |
| 1 | hsa_150 | D01205 | 0 | hsa_2550 | D01952 | 0 | hsa_6344 | D00791 |
| 1 | hsa_151 | D01205 | 0 | hsa_55879 | D08945 | 0 | hsa_3354 | D01879 |
| 1 | hsa_152 | D01205 | 0 | hsa_9177 | D00720 | 0 | hsa_1129 | D10362 |
| 1 | hsa_554 | D01213 | 0 | hsa_2565 | D01359 | 0 | hsa_56413 | D07537 |
| 1 | hsa_1813 | D01226 | 0 | hsa_1129 | D01263 | 0 | hsa_5139 | D01831 |
| 1 | hsa_1128 | D01231 | 0 | hsa_152 | D02148 | 0 | hsa_9177 | D06274 |
| 1 | hsa_1129 | D01231 | 0 | hsa_5144 | D01771 | 0 | hsa_624 | D08587 |
| 1 | hsa_1131 | D01231 | 0 | hsa_6571 | D02213 | 0 | hsa_146 | D00176 |
| 1 | hsa_1132 | D01231 | 0 | hsa_150 | D10436 | 0 | hsa_3269 | D03216 |
| 1 | hsa_1133 | D01231 | 0 | hsa_5737 | D08170 | 0 | hsa_4157 | D03402 |
| 1 | hsa_154 | D01263 | 0 | hsa_5144 | D05306 | 0 | hsa_154 | D10660 |
| 1 | hsa_1131 | D01269 | 0 | hsa_4543 | D04479 | 0 | hsa_2798 | D01077 |
| 1 | hsa_146 | D01290 | 0 | hsa_6608 | D02205 | 0 | hsa_2149 | D01096 |
| 1 | hsa_147 | D01290 | 0 | hsa_2566 | D00778 | 0 | hsa_6753 | D01008 |
| 1 | hsa_148 | D01290 | 0 | hsa_59340 | D08636 | 0 | hsa_624 | D00482 |
| 1 | hsa_146 | D01307 | 0 | hsa_11255 | D10128 | 0 | hsa_3357 | D04888 |
| 1 | hsa_147 | D01307 | 0 | hsa_5734 | D01691 | 0 | hsa_2864 | D08309 |
| 1 | hsa_148 | D01307 | 0 | hsa_552 | D08123 | 0 | hsa_146 | D08983 |
| 1 | hsa_1128 | D01315 | 0 | hsa_1909 | D09009 | 0 | hsa_2912 | D07129 |
| 1 | hsa_1129 | D01315 | 0 | hsa_3356 | D00671 | 0 | hsa_3061 | D02111 |
| 1 | hsa_1131 | D01315 | 0 | hsa_5724 | D10375 | 0 | hsa_2550 | D00573 |
| 1 | hsa_1132 | D01315 | 0 | hsa_200909 | D03221 | 0 | hsa_3350 | D09732 |
| 1 | hsa_1133 | D01315 | 0 | hsa_150 | D02705 | 0 | hsa_5141 | D04694 |
| 1 | hsa_3269 | D01317 | 0 | hsa_1128 | D01269 | 0 | hsa_2913 | D07667 |
| 1 | hsa_3356 | D01321 | 0 | hsa_2568 | D08571 | 0 | hsa_6754 | D07229 |
| 1 | hsa_146 | D01333 | 0 | hsa_134 | D01512 | 0 | hsa_6753 | D01096 |
| 1 | hsa_147 | D01333 | 0 | hsa_3351 | D01023 | 0 | hsa_10203 | D02878 |
| 1 | hsa_148 | D01333 | 0 | hsa_6755 | D04488 | 0 | hsa_2798 | D05035 |
| 1 | hsa_3269 | D01336 | 0 | hsa_3062 | D08241 | 0 | hsa_6870 | D01627 |
| 1 | hsa_5739 | D01337 | 0 | hsa_2904 | D00608 | 0 | hsa_5021 | D07832 |
| 1 | hsa_4988 | D01340 | 0 | hsa_155 | D07557 | 0 | hsa_4157 | D03361 |
| 1 | hsa_3269 | D01343 | 0 | hsa_155 | D01685 | 0 | hsa_552 | D02682 |
| 1 | hsa_146 | D01347 | 0 | hsa_5737 | D00089 | 0 | hsa_552 | D00720 |
| 1 | hsa_147 | D01347 | 0 | hsa_6755 | D03494 | 0 | hsa_6751 | D05230 |
| 1 | hsa_148 | D01347 | 0 | hsa_846 | D04888 | 0 | hsa_5021 | D01831 |
| 1 | hsa_1813 | D01348 | 0 | hsa_2567 | D01492 | 0 | hsa_3351 | D01188 |
| 1 | hsa_154 | D01349 | 0 | hsa_2913 | D01818 | 0 | hsa_3274 | D05818 |
| 1 | hsa_4988 | D01355 | 0 | hsa_10280 | D01123 | 0 | hsa_5141 | D00989 |
| 1 | hsa_3350 | D01358 | 0 | hsa_1129 | D08597 | 0 | hsa_1394 | D04860 |
| 1 | hsa_3351 | D01358 | 0 | hsa_2563 | D01007 | 0 | hsa_154 | D03953 |
| 1 | hsa_3352 | D01358 | 0 | hsa_2904 | D08837 | 0 | hsa_6570 | D04626 |
| 1 | hsa_3354 | D01358 | 0 | hsa_4987 | D00366 | 0 | hsa_6753 | D05217 |
| 1 | hsa_3355 | D01358 | 0 | hsa_3360 | D00502 | 0 | hsa_1901 | D10628 |
| 1 | hsa_1128 | D01359 | 0 | hsa_2559 | D03269 | 0 | hsa_1132 | D02404 |
| 1 | hsa_1129 | D01359 | 0 | hsa_5021 | D08861 | 0 | hsa_59340 | D02271 |
| 1 | hsa_1131 | D01359 | 0 | hsa_9568 | D07461 | 0 | hsa_1132 | D09378 |
| 1 | hsa_1132 | D01359 | 0 | hsa_200909 | D00800 | 0 | hsa_153 | D10563 |
| 1 | hsa_1133 | D01359 | 0 | hsa_6571 | D01444 | 0 | hsa_1129 | D04034 |
| 1 | hsa_154 | D01360 | 0 | hsa_4987 | D06104 | 0 | hsa_2568 | D03505 |
| 1 | hsa_154 | D01362 | 0 | hsa_6753 | D05085 | 0 | hsa_1132 | D08856 |
| 1 | hsa_153 | D01369 | 0 | hsa_2798 | D10184 | 0 | hsa_5140 | D08569 |
| 1 | hsa_154 | D01373 | 0 | hsa_134 | D05011 | 0 | hsa_5724 | D01685 |
| 1 | hsa_1128 | D01377 | 0 | hsa_3351 | D02568 | 0 | hsa_151 | D00999 |
| 1 | hsa_1129 | D01377 | 0 | hsa_4157 | D00999 | 0 | hsa_148 | D07217 |
| 1 | hsa_1131 | D01377 | 0 | hsa_1241 | D04611 | 0 | hsa_4988 | D00819 |
| 1 | hsa_1132 | D01377 | 0 | hsa_3757 | D03060 | 0 | hsa_147 | D03449 |
| 1 | hsa_1133 | D01377 | 0 | hsa_134 | D01000 | 0 | hsa_4986 | D03216 |
| 1 | hsa_4988 | D01383 | 0 | hsa_64805 | D08637 | 0 | hsa_3359 | D01858 |
| 1 | hsa_5139 | D01385 | 0 | hsa_146 | D07906 | 0 | hsa_4543 | D02419 |
| 1 | hsa_5140 | D01385 | 0 | hsa_2862 | D08587 | 0 | hsa_1813 | D07312 |
| 1 | hsa_146 | D01386 | 0 | hsa_552 | D07617 | 0 | hsa_5139 | D10662 |
| 1 | hsa_147 | D01386 | 0 | hsa_2864 | D08342 | 0 | hsa_170572 | D01101 |
| 1 | hsa_148 | D01386 | 0 | hsa_2563 | D01571 | 0 | hsa_846 | D04710 |
| 1 | hsa_4988 | D01399 | 0 | hsa_4988 | D00101 | 0 | hsa_154 | D02825 |
| 1 | hsa_146 | D01405 | 0 | hsa_1131 | D02022 | 0 | hsa_134 | D02236 |
| 1 | hsa_147 | D01405 | 0 | hsa_5144 | D10545 | 0 | hsa_5142 | D00799 |
| 1 | hsa_148 | D01405 | 0 | hsa_1133 | D00507 | 0 | hsa_5139 | D01952 |
| 1 | hsa_154 | D01428 | 0 | hsa_9568 | D05649 | 0 | hsa_151 | D02389 |
| 1 | hsa_4543 | D01438 | 0 | hsa_5139 | D01879 | 0 | hsa_200909 | D04038 |
| 1 | hsa_146 | D01444 | 0 | hsa_2550 | D08685 | 0 | hsa_155 | D09009 |
| 1 | hsa_147 | D01444 | 0 | hsa_6752 | D08639 | 0 | hsa_4988 | D08494 |
| 1 | hsa_148 | D01444 | 0 | hsa_3062 | D01445 | 0 | hsa_5141 | D07402 |
| 1 | hsa_2904 | D01445 | 0 | hsa_2693 | D02090 | 0 | hsa_3352 | D00721 |
| 1 | hsa_1813 | D01447 | 0 | hsa_2904 | D07228 | 0 | hsa_2862 | D08234 |
| 1 | hsa_1813 | D01448 | 0 | hsa_2557 | D06103 | 0 | hsa_2560 | D07491 |
| 1 | hsa_1128 | D01451 | 0 | hsa_5729 | D02247 | 0 | hsa_1129 | D00249 |
| 1 | hsa_1129 | D01451 | 0 | hsa_1133 | D01512 | 0 | hsa_55879 | D07537 |
| 1 | hsa_1131 | D01451 | 0 | hsa_10280 | D08691 | 0 | hsa_5141 | D10375 |
| 1 | hsa_1132 | D01451 | 0 | hsa_152 | D00663 | 0 | hsa_3357 | D08522 |
| 1 | hsa_1133 | D01451 | 0 | hsa_5143 | D01386 | 0 | hsa_2555 | D08487 |
| 1 | hsa_5737 | D01452 | 0 | hsa_4889 | D05711 | 0 | hsa_3061 | D06566 |
| 1 | hsa_134 | D01453 | 0 | hsa_2556 | D02372 | 0 | hsa_2798 | D07820 |
| 1 | hsa_3269 | D01460 | 0 | hsa_6753 | D05732 | 0 | hsa_6870 | D01794 |
| 1 | hsa_3350 | D01462 | 0 | hsa_2567 | D00721 | 0 | hsa_5140 | D07483 |
| 1 | hsa_1128 | D01463 | 0 | hsa_2864 | D02213 | 0 | hsa_1131 | D08473 |
| 1 | hsa_1129 | D01463 | 0 | hsa_6752 | D00722 | 0 | hsa_2559 | D09698 |
| 1 | hsa_1131 | D01463 | 0 | hsa_2555 | D07590 | 0 | hsa_886 | D04710 |
| 1 | hsa_1132 | D01463 | 0 | hsa_185 | D02419 | 0 | hsa_5737 | D05768 |
| 1 | hsa_1133 | D01463 | 0 | hsa_5732 | D09645 | 0 | hsa_1128 | D00791 |
| 1 | hsa_3274 | D01467 | 0 | hsa_2558 | D00529 | 0 | hsa_4987 | D00999 |
| 1 | hsa_1813 | D01468 | 0 | hsa_5732 | D07684 | 0 | hsa_151 | D03496 |
| 1 | hsa_146 | D01469 | 0 | hsa_6532 | D05902 | 0 | hsa_6870 | D07853 |
| 1 | hsa_147 | D01469 | 0 | hsa_1901 | D03014 | 0 | hsa_1901 | D09319 |
| 1 | hsa_148 | D01469 | 0 | hsa_2561 | D10129 | 0 | hsa_55879 | D10355 |
| 1 | hsa_153 | D01471 | 0 | hsa_3269 | D09774 | 0 | hsa_5737 | D04006 |
| 1 | hsa_1813 | D01477 | 0 | hsa_3355 | D08683 | 0 | hsa_3351 | D06671 |
| 1 | hsa_3269 | D01478 | 0 | hsa_4986 | D08590 | 0 | hsa_185 | D05196 |
| 1 | hsa_4988 | D01481 | 0 | hsa_2554 | D00603 | 0 | hsa_1131 | D09645 |
| 1 | hsa_3356 | D01482 | 0 | hsa_2558 | D09650 | 0 | hsa_624 | D08638 |
| 1 | hsa_1813 | D01485 | 0 | hsa_5139 | D10636 | 0 | hsa_2557 | D02200 |
| 1 | hsa_1128 | D01491 | 0 | hsa_1128 | D04710 | 0 | hsa_2904 | D07148 |
| 1 | hsa_1129 | D01491 | 0 | hsa_6571 | D05832 | 0 | hsa_3061 | D08041 |
| 1 | hsa_1131 | D01491 | 0 | hsa_2556 | D10336 | 0 | hsa_2693 | D04501 |
| 1 | hsa_1132 | D01491 | 0 | hsa_1394 | D08861 | 0 | hsa_1909 | D00308 |
| 1 | hsa_1133 | D01491 | 0 | hsa_64805 | D01978 | 0 | hsa_7201 | D03891 |
| 1 | hsa_153 | D01492 | 0 | hsa_1268 | D08768 | 0 | hsa_7201 | D04533 |
| 1 | hsa_1128 | D01500 | 0 | hsa_1268 | D01204 | 0 | hsa_5731 | D02825 |
| 1 | hsa_1129 | D01500 | 0 | hsa_5143 | D10703 | 0 | hsa_2913 | D05879 |
| 1 | hsa_1131 | D01500 | 0 | hsa_1901 | D04184 | 0 | hsa_886 | D08986 |
| 1 | hsa_1132 | D01500 | 0 | hsa_2565 | D02417 | 0 | hsa_4987 | D08862 |
| 1 | hsa_1133 | D01500 | 0 | hsa_6755 | D02073 | 0 | hsa_147 | D05471 |
| 1 | hsa_153 | D01504 | 0 | hsa_4986 | D08457 | 0 | hsa_2554 | D10556 |
| 1 | hsa_1813 | D01505 | 0 | hsa_6570 | D08246 | 0 | hsa_2561 | D01451 |
| 1 | hsa_153 | D01512 | 0 | hsa_9568 | D00840 | 0 | hsa_147 | D08594 |
| 1 | hsa_3356 | D01520 | 0 | hsa_2560 | D05230 | 0 | hsa_43 | D01004 |
| 1 | hsa_3357 | D01520 | 0 | hsa_3362 | D09391 | 0 | hsa_1813 | D10747 |
| 1 | hsa_3358 | D01520 | 0 | hsa_1133 | D02271 | 0 | hsa_1132 | D02741 |
| 1 | hsa_1128 | D01521 | 0 | hsa_154 | D07496 | 0 | hsa_6754 | D00997 |
| 1 | hsa_1129 | D01521 | 0 | hsa_5140 | D01795 | 0 | hsa_5724 | D03402 |
| 1 | hsa_1131 | D01521 | 0 | hsa_1909 | D08856 | 0 | hsa_135 | D02227 |
| 1 | hsa_1132 | D01521 | 0 | hsa_3362 | D00842 | 0 | hsa_886 | D07665 |
| 1 | hsa_1133 | D01521 | 0 | hsa_2862 | D08249 | 0 | hsa_2564 | D03008 |
| 1 | hsa_1813 | D01522 | 0 | hsa_56413 | D07461 | 0 | hsa_1814 | D02130 |
| 1 | hsa_1128 | D01538 | 0 | hsa_4157 | D02235 | 0 | hsa_1815 | D02670 |
| 1 | hsa_1129 | D01538 | 0 | hsa_3357 | D01548 | 0 | hsa_6869 | D00702 |
| 1 | hsa_1131 | D01538 | 0 | hsa_2862 | D06273 | 0 | hsa_3358 | D01405 |
| 1 | hsa_1132 | D01538 | 0 | hsa_135 | D08569 | 0 | hsa_6753 | D02111 |
| 1 | hsa_1133 | D01538 | 0 | hsa_6571 | D01383 | 0 | hsa_56413 | D01193 |
| 1 | hsa_153 | D01543 | 0 | hsa_2564 | D06671 | 0 | hsa_5731 | D07229 |
| 1 | hsa_3356 | D01548 | 0 | hsa_3351 | D08121 | 0 | hsa_1132 | D10631 |
| 1 | hsa_5739 | D01551 | 0 | hsa_2555 | D04860 | 0 | hsa_6869 | D10545 |
| 1 | hsa_146 | D01571 | 0 | hsa_5144 | D00835 | 0 | hsa_3359 | D03809 |
| 1 | hsa_147 | D01571 | 0 | hsa_4157 | D01847 | 0 | hsa_55879 | D04502 |
| 1 | hsa_148 | D01571 | 0 | hsa_6532 | D09990 | 0 | hsa_155 | D01592 |
| 1 | hsa_146 | D01573 | 0 | hsa_2556 | D05206 | 0 | hsa_624 | D07122 |
| 1 | hsa_147 | D01573 | 0 | hsa_7201 | D01551 | 0 | hsa_185 | D07183 |
| 1 | hsa_148 | D01573 | 0 | hsa_3351 | D07305 | 0 | hsa_2563 | D05206 |
| 1 | hsa_154 | D01589 | 0 | hsa_1133 | D02836 | 0 | hsa_1133 | D08443 |
| 1 | hsa_3269 | D01592 | 0 | hsa_3061 | D04765 | 0 | hsa_2693 | D09349 |
| 1 | hsa_1128 | D01600 | 0 | hsa_1133 | D01505 | 0 | hsa_2556 | D02163 |
| 1 | hsa_1129 | D01600 | 0 | hsa_846 | D07667 | 0 | hsa_624 | D08494 |
| 1 | hsa_1131 | D01600 | 0 | hsa_3269 | D09402 | 0 | hsa_152 | D06566 |
| 1 | hsa_1132 | D01600 | 0 | hsa_154 | D02419 | 0 | hsa_3351 | D07458 |
| 1 | hsa_1133 | D01600 | 0 | hsa_2567 | D08124 | 0 | hsa_2798 | D07496 |
| 1 | hsa_3359 | D01613 | 0 | hsa_56413 | D10181 | 0 | hsa_2557 | D06574 |
| 1 | hsa_9177 | D01613 | 0 | hsa_148 | D10567 | 0 | hsa_2568 | D06213 |
| 1 | hsa_170572 | D01613 | 0 | hsa_6344 | D09318 | 0 | hsa_2912 | D01269 |
| 1 | hsa_200909 | D01613 | 0 | hsa_3359 | D05902 | 0 | hsa_5142 | D06246 |
| 1 | hsa_285242 | D01613 | 0 | hsa_7201 | D02102 | 0 | hsa_2566 | D06660 |
| 1 | hsa_3356 | D01624 | 0 | hsa_153 | D02213 | 0 | hsa_1901 | D01485 |
| 1 | hsa_3269 | D01627 | 0 | hsa_5732 | D02048 | 0 | hsa_846 | D01019 |
| 1 | hsa_154 | D01635 | 0 | hsa_1128 | D07861 | 0 | hsa_5737 | D00089 |
| 1 | hsa_1128 | D01648 | 0 | hsa_3358 | D03580 | 0 | hsa_4543 | D02623 |
| 1 | hsa_1129 | D01648 | 0 | hsa_7201 | D07837 | 0 | hsa_4985 | D06495 |
| 1 | hsa_1131 | D01648 | 0 | hsa_135 | D09009 | 0 | hsa_5021 | D02968 |
| 1 | hsa_1132 | D01648 | 0 | hsa_3362 | D04680 | 0 | hsa_2798 | D01521 |
| 1 | hsa_1133 | D01648 | 0 | hsa_2798 | D07129 | 0 | hsa_3362 | D00796 |
| 1 | hsa_3269 | D01654 | 0 | hsa_1268 | D07496 | 0 | hsa_5142 | D02625 |
| 1 | hsa_146 | D01674 | 0 | hsa_5141 | D04694 | 0 | hsa_5140 | D01521 |
| 1 | hsa_147 | D01674 | 0 | hsa_148 | D00661 | 0 | hsa_3355 | D08923 |
| 1 | hsa_148 | D01674 | 0 | hsa_3061 | D07977 | 0 | hsa_10280 | D10129 |
| 1 | hsa_2798 | D01685 | 0 | hsa_4157 | D02878 | 0 | hsa_5737 | D10740 |
| 1 | hsa_153 | D01691 | 0 | hsa_2149 | D03889 | 0 | hsa_151 | D01698 |
| 1 | hsa_147 | D01692 | 0 | hsa_3351 | D09570 | 0 | hsa_2862 | D04492 |
| 1 | hsa_146 | D01692 | 0 | hsa_3362 | D05345 | 0 | hsa_1813 | D00819 |
| 1 | hsa_3274 | D01698 | 0 | hsa_147 | D08195 | 0 | hsa_2561 | D02893 |
| 1 | hsa_153 | D01741 | 0 | hsa_2558 | D00794 | 0 | hsa_1268 | D00373 |
| 1 | hsa_1812 | D01742 | 0 | hsa_200909 | D10660 | 0 | hsa_5140 | D07129 |
| 1 | hsa_154 | D01748 | 0 | hsa_846 | D02850 | 0 | hsa_5141 | D01177 |
| 1 | hsa_134 | D01771 | 0 | hsa_3351 | D06147 | 0 | hsa_10280 | D00374 |
| 1 | hsa_3269 | D01773 | 0 | hsa_5143 | D07837 | 0 | hsa_3757 | D06672 |
| 1 | hsa_153 | D01794 | 0 | hsa_134 | D08078 | 0 | hsa_6344 | D05832 |
| 1 | hsa_153 | D01795 | 0 | hsa_3359 | D01002 | 0 | hsa_3352 | D08226 |
| 1 | hsa_3269 | D01801 | 0 | hsa_6754 | D10545 | 0 | hsa_6754 | D08092 |
| 1 | hsa_153 | D01806 | 0 | hsa_154 | D03060 | 0 | hsa_4987 | D08376 |
| 1 | hsa_1128 | D01815 | 0 | hsa_886 | D05277 | 0 | hsa_43 | D02834 |
| 1 | hsa_1129 | D01815 | 0 | hsa_2568 | D10006 | 0 | hsa_5140 | D05768 |
| 1 | hsa_1131 | D01815 | 0 | hsa_4985 | D08047 | 0 | hsa_6869 | D08397 |
| 1 | hsa_1132 | D01815 | 0 | hsa_80834 | D00681 | 0 | hsa_2149 | D06405 |
| 1 | hsa_1133 | D01815 | 0 | hsa_1132 | D00605 | 0 | hsa_2912 | D09008 |
| 1 | hsa_886 | D01818 | 0 | hsa_10800 | D08637 | 0 | hsa_2565 | D01362 |
| 1 | hsa_146 | D01830 | 0 | hsa_1812 | D08937 | 0 | hsa_2904 | D01922 |
| 1 | hsa_147 | D01830 | 0 | hsa_1129 | D01123 | 0 | hsa_1268 | D01742 |
| 1 | hsa_148 | D01830 | 0 | hsa_4543 | D01182 | 0 | hsa_148 | D02090 |
| 1 | hsa_2798 | D01831 | 0 | hsa_552 | D00374 | 0 | hsa_5143 | D09349 |
| 1 | hsa_154 | D01835 | 0 | hsa_6532 | D01463 | 0 | hsa_200909 | D02666 |
| 1 | hsa_153 | D01847 | 0 | hsa_5139 | D03216 | 0 | hsa_2565 | D01463 |
| 1 | hsa_554 | D01855 | 0 | hsa_10280 | D04314 | 0 | hsa_2560 | D01946 |
| 1 | hsa_886 | D01858 | 0 | hsa_2693 | D04693 | 0 | hsa_554 | D01340 |
| 1 | hsa_3274 | D01861 | 0 | hsa_5737 | D06566 | 0 | hsa_64805 | D02248 |
| 1 | hsa_5731 | D01869 | 0 | hsa_10800 | D07890 | 0 | hsa_2559 | D06246 |
| 1 | hsa_1131 | D01875 | 0 | hsa_4985 | D10362 | 0 | hsa_151 | D02626 |
| 1 | hsa_4986 | D01879 | 0 | hsa_5724 | D02071 | 0 | hsa_6570 | D04532 |
| 1 | hsa_146 | D01887 | 0 | hsa_2557 | D07459 | 0 | hsa_4987 | D00138 |
| 1 | hsa_147 | D01887 | 0 | hsa_624 | D07513 | 0 | hsa_5737 | D08205 |
| 1 | hsa_148 | D01887 | 0 | hsa_552 | D03060 | 0 | hsa_3356 | D01613 |
| 1 | hsa_5732 | D01891 | 0 | hsa_6344 | D05462 | 0 | hsa_2562 | D05732 |
| 1 | hsa_1813 | D01898 | 0 | hsa_5143 | D02109 | 0 | hsa_146 | D03170 |
| 1 | hsa_3356 | D01902 | 0 | hsa_6915 | D08558 | 0 | hsa_2564 | D04868 |
| 1 | hsa_3357 | D01902 | 0 | hsa_1394 | D01992 | 0 | hsa_6915 | D04973 |
| 1 | hsa_3358 | D01902 | 0 | hsa_6570 | D00659 | 0 | hsa_1133 | D01987 |
| 1 | hsa_185 | D01922 | 0 | hsa_2862 | D09749 | 0 | hsa_1812 | D05632 |
| 1 | hsa_1131 | D01929 | 0 | hsa_846 | D01589 | 0 | hsa_2554 | D03177 |
| 1 | hsa_3356 | D01939 | 0 | hsa_3359 | D01359 | 0 | hsa_4987 | D09964 |
| 1 | hsa_3357 | D01939 | 0 | hsa_2798 | D08560 | 0 | hsa_134 | D09977 |
| 1 | hsa_3358 | D01939 | 0 | hsa_2149 | D02404 | 0 | hsa_4988 | D07837 |
| 1 | hsa_1128 | D01946 | 0 | hsa_134 | D04314 | 0 | hsa_6532 | D08343 |
| 1 | hsa_1129 | D01946 | 0 | hsa_146 | D05230 | 0 | hsa_9568 | D00403 |
| 1 | hsa_1131 | D01946 | 0 | hsa_2565 | D08395 | 0 | hsa_6870 | D01315 |
| 1 | hsa_1132 | D01946 | 0 | hsa_4157 | D02741 | 0 | hsa_3062 | D09359 |
| 1 | hsa_1133 | D01946 | 0 | hsa_5139 | D05478 | 0 | hsa_552 | D00138 |
| 1 | hsa_146 | D01952 | 0 | hsa_1394 | D01405 | 0 | hsa_2561 | D06274 |
| 1 | hsa_147 | D01952 | 0 | hsa_2915 | D07451 | 0 | hsa_150 | D08441 |
| 1 | hsa_148 | D01952 | 0 | hsa_2913 | D02069 | 0 | hsa_1128 | D02419 |
| 1 | hsa_1128 | D01955 | 0 | hsa_6915 | D08026 | 0 | hsa_285242 | D01226 |
| 1 | hsa_1129 | D01955 | 0 | hsa_2568 | D02116 | 0 | hsa_1128 | D10020 |
| 1 | hsa_1131 | D01955 | 0 | hsa_5731 | D02702 | 0 | hsa_5724 | D00999 |
| 1 | hsa_1132 | D01955 | 0 | hsa_5734 | D07493 | 0 | hsa_200909 | D01830 |
| 1 | hsa_1133 | D01955 | 0 | hsa_170572 | D01548 | 0 | hsa_1815 | D02590 |
| 1 | hsa_153 | D01958 | 0 | hsa_3355 | D05902 | 0 | hsa_59340 | D00793 |
| 1 | hsa_1128 | D01976 | 0 | hsa_55879 | D02613 | 0 | hsa_151 | D03156 |
| 1 | hsa_1129 | D01976 | 0 | hsa_2693 | D01815 | 0 | hsa_1131 | D00626 |
| 1 | hsa_1131 | D01976 | 0 | hsa_135 | D05711 | 0 | hsa_2149 | D02236 |
| 1 | hsa_1132 | D01976 | 0 | hsa_5143 | D09570 | 0 | hsa_185 | D03891 |
| 1 | hsa_1133 | D01976 | 0 | hsa_2563 | D08165 | 0 | hsa_1241 | D02041 |
| 1 | hsa_154 | D01978 | 0 | hsa_4986 | D09982 | 0 | hsa_2562 | D07550 |
| 1 | hsa_1128 | D01987 | 0 | hsa_5734 | D03177 | 0 | hsa_3360 | D00757 |
| 1 | hsa_1129 | D01987 | 0 | hsa_3350 | D03587 | 0 | hsa_5021 | D02829 |
| 1 | hsa_1131 | D01987 | 0 | hsa_624 | D10336 | 0 | hsa_56413 | D01000 |
| 1 | hsa_1132 | D01987 | 0 | hsa_624 | D09988 | 0 | hsa_2564 | D09366 |
| 1 | hsa_1133 | D01987 | 0 | hsa_3757 | D01978 | 0 | hsa_3360 | D00608 |
| 1 | hsa_3350 | D01992 | 0 | hsa_2904 | D04190 | 0 | hsa_624 | D01453 |
| 1 | hsa_1812 | D02004 | 0 | hsa_7201 | D08182 | 0 | hsa_6870 | D06353 |
| 1 | hsa_185 | D02014 | 0 | hsa_3062 | D03505 | 0 | hsa_2798 | D10677 |
| 1 | hsa_3359 | D02016 | 0 | hsa_799 | D01105 | 0 | hsa_3757 | D08594 |
| 1 | hsa_9177 | D02016 | 0 | hsa_6344 | D02236 | 0 | hsa_152 | D01174 |
| 1 | hsa_170572 | D02016 | 0 | hsa_148 | D03618 | 0 | hsa_6753 | D00997 |
| 1 | hsa_200909 | D02016 | 0 | hsa_3358 | D06405 | 0 | hsa_886 | D10375 |
| 1 | hsa_285242 | D02016 | 0 | hsa_3757 | D02535 | 0 | hsa_2561 | D04694 |
| 1 | hsa_6344 | D02021 | 0 | hsa_2556 | D10478 | 0 | hsa_152 | D01831 |
| 1 | hsa_1813 | D02022 | 0 | hsa_886 | D02250 | 0 | hsa_2566 | D00685 |
| 1 | hsa_150 | D02034 | 0 | hsa_3354 | D00176 | 0 | hsa_1131 | D05970 |
| 1 | hsa_151 | D02034 | 0 | hsa_1131 | D08482 | 0 | hsa_285242 | D07838 |
| 1 | hsa_152 | D02034 | 0 | hsa_2798 | D08836 | 0 | hsa_10280 | D07534 |
| 1 | hsa_1813 | D02035 | 0 | hsa_3274 | D10479 | 0 | hsa_6915 | D01193 |
| 1 | hsa_1813 | D02037 | 0 | hsa_1241 | D10676 | 0 | hsa_3356 | D08235 |
| 1 | hsa_2693 | D02040 | 0 | hsa_1909 | D09570 | 0 | hsa_1909 | D08522 |
| 1 | hsa_3359 | D02041 | 0 | hsa_6344 | D01478 | 0 | hsa_3359 | D02101 |
| 1 | hsa_9177 | D02041 | 0 | hsa_200909 | D01505 | 0 | hsa_1128 | D04122 |
| 1 | hsa_170572 | D02041 | 0 | hsa_5732 | D09876 | 0 | hsa_5141 | D00722 |
| 1 | hsa_200909 | D02041 | 0 | hsa_3359 | D02824 | 0 | hsa_2556 | D10441 |
| 1 | hsa_285242 | D02041 | 0 | hsa_7201 | D08207 | 0 | hsa_6755 | D07862 |
| 1 | hsa_5739 | D02048 | 0 | hsa_1133 | D10556 | 0 | hsa_5737 | D02250 |
| 1 | hsa_1128 | D02069 | 0 | hsa_2560 | D09994 | 0 | hsa_2149 | D08308 |
| 1 | hsa_1129 | D02069 | 0 | hsa_3351 | D03195 | 0 | hsa_3355 | D01794 |
| 1 | hsa_1131 | D02069 | 0 | hsa_3269 | D00678 | 0 | hsa_6608 | D01468 |
| 1 | hsa_1132 | D02069 | 0 | hsa_2912 | D04765 | 0 | hsa_4986 | D00682 |
| 1 | hsa_1133 | D02069 | 0 | hsa_5141 | D03759 | 0 | hsa_6344 | D05711 |
| 1 | hsa_1128 | D02071 | 0 | hsa_10203 | D09358 | 0 | hsa_1815 | D08473 |
| 1 | hsa_1129 | D02071 | 0 | hsa_5144 | D07077 | 0 | hsa_2560 | D01399 |
| 1 | hsa_1131 | D02071 | 0 | hsa_1268 | D01929 | 0 | hsa_185 | D01143 |
| 1 | hsa_1132 | D02071 | 0 | hsa_4986 | D06405 | 0 | hsa_150 | D10128 |
| 1 | hsa_1133 | D02071 | 0 | hsa_3061 | D02684 | 0 | hsa_170572 | D05008 |
| 1 | hsa_5731 | D02073 | 0 | hsa_1901 | D00025 | 0 | hsa_1901 | D00600 |
| 1 | hsa_153 | D02081 | 0 | hsa_3360 | D03953 | 0 | hsa_170572 | D05699 |
| 1 | hsa_3269 | D02090 | 0 | hsa_55879 | D04057 | 0 | hsa_5734 | D10349 |
| 1 | hsa_3269 | D02091 | 0 | hsa_1268 | D02682 | 0 | hsa_624 | D08626 |
| 1 | hsa_3360 | D02092 | 0 | hsa_6915 | D08064 | 0 | hsa_6915 | D08244 |
| 1 | hsa_154 | D02093 | 0 | hsa_2562 | D07684 | 0 | hsa_2559 | D01349 |
| 1 | hsa_4988 | D02095 | 0 | hsa_3061 | D08216 | 0 | hsa_1133 | D01355 |
| 1 | hsa_3356 | D02100 | 0 | hsa_1132 | D08322 | 0 | hsa_3062 | D02396 |
| 1 | hsa_3357 | D02100 | 0 | hsa_3274 | D03506 | 0 | hsa_2554 | D09391 |
| 1 | hsa_3358 | D02100 | 0 | hsa_2563 | D08293 | 0 | hsa_2566 | D06015 |
| 1 | hsa_4988 | D02101 | 0 | hsa_6570 | D02211 | 0 | hsa_6751 | D06007 |
| 1 | hsa_4988 | D02102 | 0 | hsa_2566 | D04501 | 0 | hsa_10280 | D02750 |
| 1 | hsa_4988 | D02104 | 0 | hsa_2565 | D10001 | 0 | hsa_6755 | D08648 |
| 1 | hsa_6751 | D02108 | 0 | hsa_1128 | D02760 | 0 | hsa_2904 | D08365 |
| 1 | hsa_6752 | D02108 | 0 | hsa_2550 | D02040 | 0 | hsa_5021 | D00797 |
| 1 | hsa_6753 | D02108 | 0 | hsa_4157 | D01492 | 0 | hsa_886 | D02684 |
| 1 | hsa_6754 | D02108 | 0 | hsa_5144 | D03982 | 0 | hsa_3757 | D10478 |
| 1 | hsa_6755 | D02108 | 0 | hsa_3351 | D00722 | 0 | hsa_1133 | D07868 |
| 1 | hsa_148 | D02109 | 0 | hsa_1814 | D00603 | 0 | hsa_3356 | D01205 |
| 1 | hsa_147 | D02109 | 0 | hsa_4987 | D07867 | 0 | hsa_2915 | D03504 |
| 1 | hsa_146 | D02109 | 0 | hsa_56413 | D08864 | 0 | hsa_6870 | D01193 |
| 1 | hsa_4986 | D02111 | 0 | hsa_6870 | D06394 | 0 | hsa_5732 | D02205 |
| 1 | hsa_2798 | D02116 | 0 | hsa_151 | D04184 | 0 | hsa_3356 | D00540 |
| 1 | hsa_3359 | D02130 | 0 | hsa_846 | D08341 | 0 | hsa_285242 | D07072 |
| 1 | hsa_9177 | D02130 | 0 | hsa_2550 | D08966 | 0 | hsa_6570 | D02071 |
| 1 | hsa_170572 | D02130 | 0 | hsa_2567 | D00597 | 0 | hsa_6752 | D01858 |
| 1 | hsa_200909 | D02130 | 0 | hsa_3358 | D07227 | 0 | hsa_150 | D09699 |
| 1 | hsa_285242 | D02130 | 0 | hsa_3360 | D03102 | 0 | hsa_6869 | D05779 |
| 1 | hsa_3269 | D02148 | 0 | hsa_2904 | D06157 | 0 | hsa_80834 | D04034 |
| 1 | hsa_154 | D02151 | 0 | hsa_59340 | D05996 | 0 | hsa_1128 | D09362 |
| 1 | hsa_1813 | D02163 | 0 | hsa_135 | D10663 | 0 | hsa_2559 | D00702 |
| 1 | hsa_80834 | D02192 | 0 | hsa_151 | D10545 | 0 | hsa_2556 | D04533 |
| 1 | hsa_1128 | D02200 | 0 | hsa_2566 | D08983 | 0 | hsa_150 | D00664 |
| 1 | hsa_1129 | D02200 | 0 | hsa_3359 | D03495 | 0 | hsa_2562 | D01478 |
| 1 | hsa_1131 | D02200 | 0 | hsa_3269 | D10184 | 0 | hsa_5140 | D04361 |
| 1 | hsa_1132 | D02200 | 0 | hsa_3350 | D00796 | 0 | hsa_80834 | D07831 |
| 1 | hsa_1133 | D02200 | 0 | hsa_846 | D04098 | 0 | hsa_10203 | D05891 |
| 1 | hsa_4988 | D02205 | 0 | hsa_1909 | D10100 | 0 | hsa_3757 | D00124 |
| 1 | hsa_1813 | D02208 | 0 | hsa_846 | D02037 | 0 | hsa_2566 | D01685 |
| 1 | hsa_3350 | D02211 | 0 | hsa_5143 | D00782 | 0 | hsa_10280 | D02090 |
| 1 | hsa_1128 | D02212 | 0 | hsa_6571 | D01445 | 0 | hsa_6532 | D08305 |
| 1 | hsa_1129 | D02212 | 0 | hsa_5142 | D02163 | 0 | hsa_2550 | D10562 |
| 1 | hsa_1131 | D02212 | 0 | hsa_846 | D06329 | 0 | hsa_799 | D00680 |
| 1 | hsa_1132 | D02212 | 0 | hsa_10280 | D00610 | 0 | hsa_5144 | D06394 |
| 1 | hsa_1133 | D02212 | 0 | hsa_6870 | D05730 | 0 | hsa_147 | D07458 |
| 1 | hsa_1813 | D02213 | 0 | hsa_6753 | D02983 | 0 | hsa_3357 | D08425 |
| 1 | hsa_4986 | D02227 | 0 | hsa_55879 | D00757 | 0 | hsa_59340 | D05276 |
| 1 | hsa_3269 | D02234 | 0 | hsa_285242 | D09610 | 0 | hsa_6915 | D00789 |
| 1 | hsa_552 | D02235 | 0 | hsa_1241 | D08249 | 0 | hsa_134 | D01520 |
| 1 | hsa_1813 | D02236 | 0 | hsa_3355 | D01385 | 0 | hsa_155 | D08255 |
| 1 | hsa_4986 | D02238 | 0 | hsa_1133 | D08837 | 0 | hsa_1394 | D07489 |
| 1 | hsa_3269 | D02245 | 0 | hsa_6755 | D10725 | 0 | hsa_2798 | D01691 |
| 1 | hsa_1128 | D02246 | 0 | hsa_150 | D07837 | 0 | hsa_147 | D10220 |
| 1 | hsa_1129 | D02246 | 0 | hsa_3359 | D07590 | 0 | hsa_2693 | D02681 |
| 1 | hsa_1131 | D02246 | 0 | hsa_886 | D07491 | 0 | hsa_6571 | D00599 |
| 1 | hsa_1132 | D02246 | 0 | hsa_554 | D10326 | 0 | hsa_6532 | D10493 |
| 1 | hsa_1133 | D02246 | 0 | hsa_200909 | D08078 | 0 | hsa_6344 | D04501 |
| 1 | hsa_1128 | D02247 | 0 | hsa_6870 | D10671 | 0 | hsa_5140 | D09696 |
| 1 | hsa_1129 | D02247 | 0 | hsa_5142 | D09797 | 0 | hsa_150 | D07740 |
| 1 | hsa_1131 | D02247 | 0 | hsa_5724 | D01428 | 0 | hsa_2554 | D07513 |
| 1 | hsa_1132 | D02247 | 0 | hsa_4987 | D02641 | 0 | hsa_3362 | D10375 |
| 1 | hsa_1133 | D02247 | 0 | hsa_10203 | D07734 | 0 | hsa_6608 | D10181 |
| 1 | hsa_3356 | D02248 | 0 | hsa_554 | D00608 | 0 | hsa_6608 | D00626 |
| 1 | hsa_3357 | D02248 | 0 | hsa_5731 | D10628 | 0 | hsa_6751 | D06355 |
| 1 | hsa_3358 | D02248 | 0 | hsa_2904 | D06328 | 0 | hsa_146 | D05010 |
| 1 | hsa_3269 | D02249 | 0 | hsa_170572 | D10338 | 0 | hsa_3358 | D01485 |
| 1 | hsa_6751 | D02250 | 0 | hsa_80834 | D02148 | 0 | hsa_150 | D08146 |
| 1 | hsa_4988 | D02271 | 0 | hsa_1815 | D01105 | 0 | hsa_3351 | D08192 |
| 1 | hsa_154 | D02281 | 0 | hsa_3350 | D00717 | 0 | hsa_6344 | D04405 |
| 1 | hsa_5737 | D02343 | 0 | hsa_59340 | D08099 | 0 | hsa_3354 | D04626 |
| 1 | hsa_146 | D02349 | 0 | hsa_3354 | D02964 | 0 | hsa_846 | D04683 |
| 1 | hsa_147 | D02349 | 0 | hsa_2566 | D09205 | 0 | hsa_2559 | D06021 |
| 1 | hsa_148 | D02349 | 0 | hsa_9568 | D03060 | 0 | hsa_146 | D05649 |
| 1 | hsa_3350 | D02357 | 0 | hsa_3062 | D02750 | 0 | hsa_10800 | D01818 |
| 1 | hsa_3351 | D02357 | 0 | hsa_1241 | D00997 | 0 | hsa_2915 | D04038 |
| 1 | hsa_3352 | D02357 | 0 | hsa_3350 | D09934 | 0 | hsa_56413 | D06103 |
| 1 | hsa_3356 | D02363 | 0 | hsa_1128 | D02670 | 0 | hsa_1268 | D07557 |
| 1 | hsa_2798 | D02369 | 0 | hsa_153 | D06659 | 0 | hsa_2555 | D04533 |
| 1 | hsa_1813 | D02371 | 0 | hsa_10203 | D08389 | 0 | hsa_3362 | D08293 |
| 1 | hsa_3356 | D02372 | 0 | hsa_5140 | D02693 | 0 | hsa_3359 | D09336 |
| 1 | hsa_80834 | D02381 | 0 | hsa_5139 | D01205 | 0 | hsa_2566 | D02724 |
| 1 | hsa_10800 | D02384 | 0 | hsa_6915 | D02212 | 0 | hsa_2568 | D01773 |
| 1 | hsa_150 | D02388 | 0 | hsa_151 | D04683 | 0 | hsa_6753 | D08305 |
| 1 | hsa_151 | D02388 | 0 | hsa_10800 | D07665 | 0 | hsa_5729 | D02725 |
| 1 | hsa_152 | D02388 | 0 | hsa_3269 | D05099 | 0 | hsa_6870 | D00308 |
| 1 | hsa_153 | D02389 | 0 | hsa_6532 | D08684 | 0 | hsa_1909 | D05008 |
| 1 | hsa_154 | D02396 | 0 | hsa_6608 | D02095 | 0 | hsa_2913 | D10677 |
| 1 | hsa_154 | D02404 | 0 | hsa_5141 | D07990 | 0 | hsa_5729 | D06234 |
| 1 | hsa_1128 | D02417 | 0 | hsa_886 | D00482 | 0 | hsa_4543 | D10220 |
| 1 | hsa_1129 | D02417 | 0 | hsa_2912 | D09954 | 0 | hsa_2556 | D07765 |
| 1 | hsa_1131 | D02417 | 0 | hsa_5141 | D03402 | 0 | hsa_846 | D08124 |
| 1 | hsa_1132 | D02417 | 0 | hsa_886 | D01143 | 0 | hsa_1813 | D01026 |
| 1 | hsa_1133 | D02417 | 0 | hsa_152 | D02846 | 0 | hsa_10203 | D00720 |
| 1 | hsa_3269 | D02419 | 0 | hsa_152 | D09699 | 0 | hsa_3354 | D09994 |
| 1 | hsa_1128 | D02535 | 0 | hsa_6753 | D10185 | 0 | hsa_5731 | D09607 |
| 1 | hsa_1129 | D02535 | 0 | hsa_4543 | D06247 | 0 | hsa_1394 | D09842 |
| 1 | hsa_1131 | D02535 | 0 | hsa_9568 | D10117 | 0 | hsa_147 | D03618 |
| 1 | hsa_1132 | D02535 | 0 | hsa_146 | D07890 | 0 | hsa_2564 | D06569 |
| 1 | hsa_1133 | D02535 | 0 | hsa_5724 | D03170 | 0 | hsa_2912 | D01685 |
| 1 | hsa_3350 | D02568 | 0 | hsa_59340 | D06015 | 0 | hsa_1129 | D02271 |
| 1 | hsa_3350 | D02576 | 0 | hsa_2563 | D08117 | 0 | hsa_3355 | D01521 |
| 1 | hsa_3350 | D02577 | 0 | hsa_5734 | D08856 | 0 | hsa_554 | D03783 |
| 1 | hsa_5724 | D02590 | 0 | hsa_5021 | D07482 | 0 | hsa_1394 | D07795 |
| 1 | hsa_1128 | D02599 | 0 | hsa_154 | D05366 | 0 | hsa_2567 | D04533 |
| 1 | hsa_1129 | D02599 | 0 | hsa_6755 | D05738 | 0 | hsa_5734 | D01654 |
| 1 | hsa_1131 | D02599 | 0 | hsa_1131 | D00291 | 0 | hsa_6752 | D01773 |
| 1 | hsa_1132 | D02599 | 0 | hsa_10800 | D10725 | 0 | hsa_2564 | D06273 |
| 1 | hsa_1133 | D02599 | 0 | hsa_135 | D08343 | 0 | hsa_3359 | D10403 |
| 1 | hsa_1813 | D02605 | 0 | hsa_886 | D01818 | 0 | hsa_2557 | D08192 |
| 1 | hsa_1813 | D02609 | 0 | hsa_2562 | D06622 | 0 | hsa_10800 | D03898 |
| 1 | hsa_1813 | D02612 | 0 | hsa_155 | D05035 | 0 | hsa_10203 | D06140 |
| 1 | hsa_1813 | D02613 | 0 | hsa_10800 | D00784 | 0 | hsa_5143 | D10349 |
| 1 | hsa_3356 | D02622 | 0 | hsa_2693 | D10663 | 0 | hsa_5144 | D04868 |
| 1 | hsa_1813 | D02623 | 0 | hsa_148 | D09645 | 0 | hsa_4987 | D00685 |
| 1 | hsa_1813 | D02625 | 0 | hsa_1394 | D07887 | 0 | hsa_9177 | D08241 |
| 1 | hsa_1813 | D02626 | 0 | hsa_6344 | D02774 | 0 | hsa_1133 | D07305 |
| 1 | hsa_3359 | D02632 | 0 | hsa_6870 | D05478 | 0 | hsa_2149 | D03415 |
| 1 | hsa_9177 | D02632 | 0 | hsa_3351 | D00644 | 0 | hsa_10280 | D07259 |
| 1 | hsa_170572 | D02632 | 0 | hsa_56413 | D02249 | 0 | hsa_155 | D10375 |
| 1 | hsa_200909 | D02632 | 0 | hsa_10203 | D08235 | 0 | hsa_3274 | D02775 |
| 1 | hsa_285242 | D02632 | 0 | hsa_6915 | D06613 | 0 | hsa_4543 | D02363 |
| 1 | hsa_1813 | D02641 | 0 | hsa_1129 | D08341 | 0 | hsa_56413 | D09994 |
| 1 | hsa_1813 | D02645 | 0 | hsa_2559 | D09797 | 0 | hsa_5732 | D08249 |
| 1 | hsa_1813 | D02663 | 0 | hsa_185 | D05660 | 0 | hsa_4543 | D07682 |
| 1 | hsa_3356 | D02666 | 0 | hsa_2555 | D03725 | 0 | hsa_4543 | D08297 |
| 1 | hsa_1813 | D02670 | 0 | hsa_3355 | D02710 | 0 | hsa_285242 | D02577 |
| 1 | hsa_1812 | D02676 | 0 | hsa_5724 | D00784 | 0 | hsa_6571 | D02246 |
| 1 | hsa_1813 | D02681 | 0 | hsa_2554 | D07861 | 0 | hsa_4543 | D08850 |
| 1 | hsa_1813 | D02682 | 0 | hsa_3356 | D00561 | 0 | hsa_1132 | D10362 |
| 1 | hsa_1813 | D02683 | 0 | hsa_6608 | D02702 | 0 | hsa_2558 | D00776 |
| 1 | hsa_10280 | D02684 | 0 | hsa_155 | D07076 | 0 | hsa_552 | D03198 |
| 1 | hsa_1813 | D02688 | 0 | hsa_3352 | D01858 | 0 | hsa_2562 | D06246 |
| 1 | hsa_4543 | D02689 | 0 | hsa_3352 | D04531 | 0 | hsa_799 | D01674 |
| 1 | hsa_886 | D02693 | 0 | hsa_846 | D08057 | 0 | hsa_2561 | D07990 |
| 1 | hsa_155 | D02702 | 0 | hsa_55879 | D01879 | 0 | hsa_64805 | D06140 |
| 1 | hsa_5731 | D02705 | 0 | hsa_5729 | D00796 | 0 | hsa_150 | D04184 |
| 1 | hsa_886 | D02710 | 0 | hsa_155 | D07458 | 0 | hsa_2912 | D10497 |
| 1 | hsa_5737 | D02719 | 0 | hsa_5140 | D09845 | 0 | hsa_3359 | D01452 |
| 1 | hsa_5739 | D02720 | 0 | hsa_6570 | D00717 | 0 | hsa_799 | D08856 |
| 1 | hsa_5731 | D02722 | 0 | hsa_1133 | D08395 | 0 | hsa_6751 | D02208 |
| 1 | hsa_5737 | D02724 | 0 | hsa_2904 | D00798 | 0 | hsa_146 | D08561 |
| 1 | hsa_5732 | D02725 | 0 | hsa_2567 | D00308 | 0 | hsa_5729 | D08836 |
| 1 | hsa_3360 | D02730 | 0 | hsa_3269 | D01835 | 0 | hsa_6754 | D03693 |
| 1 | hsa_10800 | D02732 | 0 | hsa_2862 | D02722 | 0 | hsa_2864 | D00702 |
| 1 | hsa_2798 | D02738 | 0 | hsa_2558 | D07312 | 0 | hsa_5732 | D05099 |
| 1 | hsa_10800 | D02739 | 0 | hsa_1133 | D02739 | 0 | hsa_2565 | D02104 |
| 1 | hsa_10800 | D02741 | 0 | hsa_1131 | D08040 | 0 | hsa_9568 | D08207 |
| 1 | hsa_1128 | D02750 | 0 | hsa_2558 | D06495 | 0 | hsa_150 | D10563 |
| 1 | hsa_3269 | D02760 | 0 | hsa_4987 | D08692 | 0 | hsa_59340 | D01922 |
| 1 | hsa_153 | D02765 | 0 | hsa_1128 | D04823 | 0 | hsa_151 | D05085 |
| 1 | hsa_3350 | D02767 | 0 | hsa_2563 | D04006 | 0 | hsa_3351 | D10375 |
| 1 | hsa_146 | D02774 | 0 | hsa_846 | D04924 | 0 | hsa_1815 | D07218 |
| 1 | hsa_147 | D02774 | 0 | hsa_4986 | D05731 | 0 | hsa_2915 | D09766 |
| 1 | hsa_148 | D02774 | 0 | hsa_3274 | D02092 | 0 | hsa_6751 | D07937 |
| 1 | hsa_1812 | D02775 | 0 | hsa_2862 | D07729 | 0 | hsa_6753 | D05970 |
| 1 | hsa_2862 | D02801 | 0 | hsa_552 | D10562 | 0 | hsa_5737 | D10567 |
| 1 | hsa_3351 | D02824 | 0 | hsa_285242 | D00679 | 0 | hsa_3351 | D07838 |
| 1 | hsa_3351 | D02825 | 0 | hsa_56413 | D02535 | 0 | hsa_6751 | D00602 |
| 1 | hsa_3359 | D02829 | 0 | hsa_2568 | D01898 | 0 | hsa_4985 | D08216 |
| 1 | hsa_9177 | D02829 | 0 | hsa_1132 | D08578 | 0 | hsa_5734 | D00502 |
| 1 | hsa_170572 | D02829 | 0 | hsa_6869 | D07458 | 0 | hsa_7201 | D02834 |
| 1 | hsa_200909 | D02829 | 0 | hsa_6752 | D08358 | 0 | hsa_799 | D05742 |
| 1 | hsa_285242 | D02829 | 0 | hsa_5732 | D02625 | 0 | hsa_846 | D00079 |
| 1 | hsa_153 | D02834 | 0 | hsa_3357 | D01592 | 0 | hsa_1909 | D09991 |
| 1 | hsa_3356 | D02836 | 0 | hsa_4889 | D03898 | 0 | hsa_4987 | D08253 |
| 1 | hsa_10800 | D02846 | 0 | hsa_1268 | D02116 | 0 | hsa_6869 | D07862 |
| 1 | hsa_10800 | D02848 | 0 | hsa_3352 | D10145 | 0 | hsa_2565 | D08248 |
| 1 | hsa_10800 | D02849 | 0 | hsa_1241 | D02041 | 0 | hsa_6755 | D03702 |
| 1 | hsa_10800 | D02850 | 0 | hsa_185 | D00603 | 0 | hsa_5732 | D01355 |
| 1 | hsa_10800 | D02851 | 0 | hsa_3357 | D03264 | 0 | hsa_2149 | D00844 |
| 1 | hsa_5731 | D02860 | 0 | hsa_1241 | D05007 | 0 | hsa_886 | D01573 |
| 1 | hsa_1128 | D02876 | 0 | hsa_6870 | D07076 | 0 | hsa_151 | D01898 |
| 1 | hsa_4988 | D02878 | 0 | hsa_147 | D03290 | 0 | hsa_2563 | D08395 |
| 1 | hsa_3356 | D02893 | 0 | hsa_3274 | D07402 | 0 | hsa_151 | D00661 |
| 1 | hsa_146 | D02901 | 0 | hsa_56413 | D07977 | 0 | hsa_3061 | D07837 |
| 1 | hsa_147 | D02901 | 0 | hsa_3362 | D03102 | 0 | hsa_10800 | D01543 |
| 1 | hsa_148 | D02901 | 0 | hsa_3351 | D00479 | 0 | hsa_2904 | D04126 |
| 1 | hsa_185 | D02939 | 0 | hsa_64805 | D09994 | 0 | hsa_5143 | D03556 |
| 1 | hsa_3269 | D02950 | 0 | hsa_3355 | D08117 | 0 | hsa_2915 | D05649 |
| 1 | hsa_134 | D02964 | 0 | hsa_152 | D08940 | 0 | hsa_43 | D06569 |
| 1 | hsa_6869 | D02968 | 0 | hsa_2566 | D01205 | 0 | hsa_1268 | D10631 |
| 1 | hsa_150 | D02972 | 0 | hsa_3354 | D08305 | 0 | hsa_1814 | D05340 |
| 1 | hsa_151 | D02972 | 0 | hsa_5143 | D05217 | 0 | hsa_1129 | D04184 |
| 1 | hsa_152 | D02972 | 0 | hsa_4889 | D08027 | 0 | hsa_56413 | D01471 |
| 1 | hsa_146 | D02976 | 0 | hsa_1909 | D04641 | 0 | hsa_2565 | D01500 |
| 1 | hsa_147 | D02976 | 0 | hsa_6570 | D03580 | 0 | hsa_151 | D07217 |
| 1 | hsa_148 | D02976 | 0 | hsa_1394 | D02632 | 0 | hsa_11255 | D10325 |
| 1 | hsa_154 | D02981 | 0 | hsa_185 | D07511 | 0 | hsa_80834 | D05478 |
| 1 | hsa_552 | D02983 | 0 | hsa_1901 | D08293 | 0 | hsa_3062 | D06103 |
| 1 | hsa_3356 | D02995 | 0 | hsa_2915 | D02972 | 0 | hsa_4987 | D05011 |
| 1 | hsa_3357 | D02995 | 0 | hsa_1133 | D04888 | 0 | hsa_2693 | D07451 |
| 1 | hsa_3358 | D02995 | 0 | hsa_5737 | D01004 | 0 | hsa_43 | D03937 |
| 1 | hsa_150 | D03002 | 0 | hsa_2557 | D06317 | 0 | hsa_5729 | D08598 |
| 1 | hsa_151 | D03002 | 0 | hsa_2798 | D03449 | 0 | hsa_3352 | D07560 |
| 1 | hsa_152 | D03002 | 0 | hsa_2567 | D08572 | 0 | hsa_5737 | D04501 |
| 1 | hsa_5021 | D03008 | 0 | hsa_2558 | D02245 | 0 | hsa_5737 | D01369 |
| 1 | hsa_1909 | D03009 | 0 | hsa_2567 | D03504 | 0 | hsa_3352 | D04038 |
| 1 | hsa_3351 | D03014 | 0 | hsa_2912 | D00680 | 0 | hsa_43 | D08508 |
| 1 | hsa_134 | D03051 | 0 | hsa_11255 | D02192 | 0 | hsa_147 | D00195 |
| 1 | hsa_3359 | D03060 | 0 | hsa_6570 | D07560 | 0 | hsa_3357 | D02760 |
| 1 | hsa_9177 | D03060 | 0 | hsa_3360 | D00677 | 0 | hsa_147 | D03198 |
| 1 | hsa_170572 | D03060 | 0 | hsa_6753 | D07406 | 0 | hsa_185 | D01362 |
| 1 | hsa_200909 | D03060 | 0 | hsa_5142 | D01025 | 0 | hsa_134 | D05366 |
| 1 | hsa_285242 | D03060 | 0 | hsa_1815 | D09994 | 0 | hsa_5144 | D06274 |
| 1 | hsa_1128 | D03087 | 0 | hsa_3356 | D01444 | 0 | hsa_135 | D02705 |
| 1 | hsa_1129 | D03087 | 0 | hsa_886 | D02732 | 0 | hsa_5143 | D01978 |
| 1 | hsa_1131 | D03087 | 0 | hsa_3350 | D10391 | 0 | hsa_1132 | D05366 |
| 1 | hsa_1132 | D03087 | 0 | hsa_11255 | D00840 | 0 | hsa_3757 | D09949 |
| 1 | hsa_1133 | D03087 | 0 | hsa_3757 | D07667 | 0 | hsa_5144 | D05818 |
| 1 | hsa_3269 | D03102 | 0 | hsa_3362 | D10430 | 0 | hsa_3357 | D01520 |
| 1 | hsa_135 | D03120 | 0 | hsa_2864 | D01794 | 0 | hsa_6754 | D01023 |
| 1 | hsa_4988 | D03156 | 0 | hsa_799 | D08246 | 0 | hsa_147 | D07665 |
| 1 | hsa_153 | D03170 | 0 | hsa_5142 | D07937 | 0 | hsa_1909 | D02720 |
| 1 | hsa_153 | D03177 | 0 | hsa_10280 | D04533 | 0 | hsa_3360 | D02705 |
| 1 | hsa_153 | D03195 | 0 | hsa_6754 | D01477 | 0 | hsa_6870 | D07990 |
| 1 | hsa_4986 | D03197 | 0 | hsa_1132 | D08195 | 0 | hsa_5140 | D08384 |
| 1 | hsa_154 | D03198 | 0 | hsa_1131 | D06622 | 0 | hsa_1129 | D02878 |
| 1 | hsa_135 | D03212 | 0 | hsa_6570 | D06567 | 0 | hsa_6755 | D05691 |
| 1 | hsa_1813 | D03214 | 0 | hsa_3350 | D01522 | 0 | hsa_5729 | D02683 |
| 1 | hsa_2862 | D03216 | 0 | hsa_7201 | D02108 | 0 | hsa_2913 | D08031 |
| 1 | hsa_6869 | D03221 | 0 | hsa_2554 | D05345 | 0 | hsa_80834 | D10563 |
| 1 | hsa_1128 | D03264 | 0 | hsa_5729 | D08233 | 0 | hsa_10800 | D08585 |
| 1 | hsa_1129 | D03264 | 0 | hsa_3360 | D08045 | 0 | hsa_6915 | D09766 |
| 1 | hsa_1131 | D03264 | 0 | hsa_170572 | D02850 | 0 | hsa_2904 | D08300 |
| 1 | hsa_1132 | D03264 | 0 | hsa_5144 | D04502 | 0 | hsa_59340 | D02205 |
| 1 | hsa_1133 | D03264 | 0 | hsa_2559 | D02860 | 0 | hsa_6570 | D07867 |
| 1 | hsa_2798 | D03267 | 0 | hsa_846 | D00378 | 0 | hsa_9177 | D02725 |
| 1 | hsa_5732 | D03269 | 0 | hsa_2562 | D10326 | 0 | hsa_2557 | D09797 |
| 1 | hsa_3356 | D03274 | 0 | hsa_1812 | D09205 | 0 | hsa_4157 | D08244 |
| 1 | hsa_3357 | D03274 | 0 | hsa_3354 | D09400 | 0 | hsa_6915 | D03898 |
| 1 | hsa_3358 | D03274 | 0 | hsa_1815 | D01101 | 0 | hsa_3062 | D08598 |
| 1 | hsa_1128 | D03276 | 0 | hsa_2550 | D02705 | 0 | hsa_3757 | D07765 |
| 1 | hsa_1129 | D03276 | 0 | hsa_4986 | D09705 | 0 | hsa_3274 | D00997 |
| 1 | hsa_1131 | D03276 | 0 | hsa_2558 | D08318 | 0 | hsa_147 | D10309 |
| 1 | hsa_1132 | D03276 | 0 | hsa_200909 | D09705 | 0 | hsa_554 | D04649 |
| 1 | hsa_1133 | D03276 | 0 | hsa_3269 | D01004 | 0 | hsa_1132 | D07183 |
| 1 | hsa_3269 | D03290 | 0 | hsa_2566 | D01201 | 0 | hsa_2864 | D02381 |
| 1 | hsa_64805 | D03359 | 0 | hsa_155 | D02419 | 0 | hsa_3062 | D01355 |
| 1 | hsa_3269 | D03360 | 0 | hsa_2565 | D07937 | 0 | hsa_1812 | D09784 |
| 1 | hsa_64805 | D03361 | 0 | hsa_3269 | D01978 | 0 | hsa_4543 | D08257 |
| 1 | hsa_2693 | D03373 | 0 | hsa_3757 | D03783 | 0 | hsa_2913 | D02014 |
| 1 | hsa_5737 | D03399 | 0 | hsa_2568 | D03860 | 0 | hsa_2862 | D08683 |
| 1 | hsa_154 | D03402 | 0 | hsa_9177 | D07465 | 0 | hsa_148 | D04625 |
| 1 | hsa_4988 | D03405 | 0 | hsa_3352 | D03221 | 0 | hsa_2554 | D09693 |
| 1 | hsa_146 | D03415 | 0 | hsa_4987 | D08768 | 0 | hsa_7201 | D03518 |
| 1 | hsa_147 | D03415 | 0 | hsa_9177 | D00797 | 0 | hsa_2561 | D00720 |
| 1 | hsa_148 | D03415 | 0 | hsa_6751 | D09344 | 0 | hsa_2862 | D09866 |
| 1 | hsa_153 | D03449 | 0 | hsa_4988 | D04820 | 0 | hsa_3062 | D02834 |
| 1 | hsa_1812 | D03494 | 0 | hsa_6753 | D09749 | 0 | hsa_624 | D03402 |
| 1 | hsa_3359 | D03495 | 0 | hsa_2562 | D04122 | 0 | hsa_10280 | D05053 |
| 1 | hsa_9177 | D03495 | 0 | hsa_624 | D03405 | 0 | hsa_80834 | D01504 |
| 1 | hsa_170572 | D03495 | 0 | hsa_2563 | D08255 | 0 | hsa_4987 | D08485 |
| 1 | hsa_200909 | D03495 | 0 | hsa_3274 | D08525 | 0 | hsa_1241 | D04626 |
| 1 | hsa_285242 | D03495 | 0 | hsa_285242 | D08986 | 0 | hsa_3757 | D09693 |
| 1 | hsa_3359 | D03496 | 0 | hsa_2555 | D05478 | 0 | hsa_64805 | D10119 |
| 1 | hsa_9177 | D03496 | 0 | hsa_1394 | D05015 | 0 | hsa_56413 | D06374 |
| 1 | hsa_170572 | D03496 | 0 | hsa_2693 | D00378 | 0 | hsa_5021 | D02246 |
| 1 | hsa_200909 | D03496 | 0 | hsa_1132 | D10725 | 0 | hsa_170572 | D00081 |
| 1 | hsa_285242 | D03496 | 0 | hsa_10800 | D07175 | 0 | hsa_2568 | D00081 |
| 1 | hsa_153 | D03502 | 0 | hsa_4889 | D10660 | 0 | hsa_6752 | D01482 |
| 1 | hsa_846 | D03504 | 0 | hsa_43 | D04717 | 0 | hsa_6344 | D03937 |
| 1 | hsa_846 | D03505 | 0 | hsa_3274 | D05107 | 0 | hsa_7201 | D07997 |
| 1 | hsa_3356 | D03506 | 0 | hsa_2557 | D08473 | 0 | hsa_9568 | D01952 |
| 1 | hsa_11255 | D03518 | 0 | hsa_2563 | D08241 | 0 | hsa_886 | D08046 |
| 1 | hsa_1812 | D03534 | 0 | hsa_1812 | D05085 | 0 | hsa_4543 | D07076 |
| 1 | hsa_3269 | D03535 | 0 | hsa_11255 | D07218 | 0 | hsa_170572 | D08064 |
| 1 | hsa_1813 | D03556 | 0 | hsa_5021 | D00481 | 0 | hsa_3350 | D01193 |
| 1 | hsa_4988 | D03580 | 0 | hsa_6752 | D05007 | 0 | hsa_1813 | D08861 |
| 1 | hsa_154 | D03587 | 0 | hsa_2912 | D00819 | 0 | hsa_2864 | D05970 |
| 1 | hsa_4986 | D03618 | 0 | hsa_1909 | D02371 | 0 | hsa_2556 | D08047 |
| 1 | hsa_3269 | D03622 | 0 | hsa_3274 | D08216 | 0 | hsa_5732 | D05727 |
| 1 | hsa_146 | D03648 | 0 | hsa_2556 | D07810 | 0 | hsa_846 | D00669 |
| 1 | hsa_147 | D03648 | 0 | hsa_155 | D07832 | 0 | hsa_5141 | D06147 |
| 1 | hsa_148 | D03648 | 0 | hsa_2564 | D08132 | 0 | hsa_1241 | D01008 |
| 1 | hsa_150 | D03672 | 0 | hsa_146 | D08047 | 0 | hsa_2565 | D08473 |
| 1 | hsa_151 | D03672 | 0 | hsa_4987 | D06280 | 0 | hsa_3274 | D00682 |
| 1 | hsa_152 | D03672 | 0 | hsa_4889 | D01017 | 0 | hsa_2567 | D08474 |
| 1 | hsa_3269 | D03693 | 0 | hsa_2566 | D01830 | 0 | hsa_6915 | D00796 |
| 1 | hsa_150 | D03702 | 0 | hsa_7201 | D01175 | 0 | hsa_2862 | D06293 |
| 1 | hsa_151 | D03702 | 0 | hsa_285242 | D01573 | 0 | hsa_5724 | D02836 |
| 1 | hsa_152 | D03702 | 0 | hsa_3360 | D01485 | 0 | hsa_5021 | D03264 |
| 1 | hsa_3269 | D03704 | 0 | hsa_152 | D06007 | 0 | hsa_4988 | D01226 |
| 1 | hsa_1128 | D03711 | 0 | hsa_6915 | D02849 | 0 | hsa_154 | D09400 |
| 1 | hsa_1129 | D03711 | 0 | hsa_4889 | D00646 | 0 | hsa_1394 | D02349 |
| 1 | hsa_1131 | D03711 | 0 | hsa_2149 | D09934 | 0 | hsa_1241 | D01358 |
| 1 | hsa_1132 | D03711 | 0 | hsa_6532 | D01444 | 0 | hsa_2912 | D05196 |
| 1 | hsa_1133 | D03711 | 0 | hsa_5139 | D08684 | 0 | hsa_9568 | D03502 |
| 1 | hsa_4988 | D03725 | 0 | hsa_1394 | D04868 | 0 | hsa_2864 | D04642 |
| 1 | hsa_153 | D03729 | 0 | hsa_2798 | D00249 | 0 | hsa_2915 | D09695 |
| 1 | hsa_153 | D03759 | 0 | hsa_6751 | D01478 | 0 | hsa_3357 | D01359 |
| 1 | hsa_4988 | D03783 | 0 | hsa_2556 | D08937 | 0 | hsa_886 | D07184 |
| 1 | hsa_4988 | D03809 | 0 | hsa_5724 | D08293 | 0 | hsa_155 | D08598 |
| 1 | hsa_1128 | D03814 | 0 | hsa_59340 | D05366 | 0 | hsa_4985 | D00663 |
| 1 | hsa_1129 | D03814 | 0 | hsa_6915 | D09934 | 0 | hsa_80834 | D02666 |
| 1 | hsa_1131 | D03814 | 0 | hsa_5729 | D10349 | 0 | hsa_1813 | D10362 |
| 1 | hsa_1132 | D03814 | 0 | hsa_6753 | D07259 | 0 | hsa_3269 | D00794 |
| 1 | hsa_1133 | D03814 | 0 | hsa_148 | D05008 | 0 | hsa_1241 | D00784 |
| 1 | hsa_1128 | D03824 | 0 | hsa_2693 | D03495 | 0 | hsa_5140 | D01467 |
| 1 | hsa_1129 | D03824 | 0 | hsa_4987 | D05366 | 0 | hsa_5142 | D00124 |
| 1 | hsa_1131 | D03824 | 0 | hsa_56413 | D03415 | 0 | hsa_2915 | D02666 |
| 1 | hsa_1132 | D03824 | 0 | hsa_3356 | D10099 | 0 | hsa_56413 | D02235 |
| 1 | hsa_1133 | D03824 | 0 | hsa_152 | D08425 | 0 | hsa_2556 | D02004 |
| 1 | hsa_4988 | D03825 | 0 | hsa_6571 | D01176 | 0 | hsa_9177 | D05768 |
| 1 | hsa_3269 | D03854 | 0 | hsa_3757 | D01017 | 0 | hsa_64805 | D08113 |
| 1 | hsa_4988 | D03860 | 0 | hsa_5141 | D01148 | 0 | hsa_150 | D04184 |
| 1 | hsa_3274 | D03889 | 0 | hsa_6869 | D02964 | 0 | hsa_1909 | D04122 |
| 1 | hsa_154 | D03891 | 0 | hsa_55879 | D01193 | 0 | hsa_2568 | D10546 |
| 1 | hsa_5141 | D03898 | 0 | hsa_1131 | D01337 | 0 | hsa_59340 | D01348 |
| 1 | hsa_5142 | D03898 | 0 | hsa_3355 | D01818 | 0 | hsa_2555 | D02236 |
| 1 | hsa_5143 | D03898 | 0 | hsa_5729 | D02724 | 0 | hsa_3359 | D02372 |
| 1 | hsa_5144 | D03898 | 0 | hsa_3359 | D01955 | 0 | hsa_5142 | D09336 |
| 1 | hsa_1812 | D03937 | 0 | hsa_6755 | D10562 | 0 | hsa_1812 | D01939 |
| 1 | hsa_1909 | D03953 | 0 | hsa_2567 | D08639 | 0 | hsa_5021 | D07624 |
| 1 | hsa_3351 | D03982 | 0 | hsa_6754 | D02246 | 0 | hsa_6755 | D00529 |
| 1 | hsa_3351 | D03983 | 0 | hsa_285242 | D00840 | 0 | hsa_3358 | D00782 |
| 1 | hsa_134 | D04006 | 0 | hsa_3357 | D01875 | 0 | hsa_1129 | D09610 |
| 1 | hsa_146 | D04018 | 0 | hsa_5141 | D07458 | 0 | hsa_4889 | D08229 |
| 1 | hsa_147 | D04018 | 0 | hsa_59340 | D10100 | 0 | hsa_6344 | D05345 |
| 1 | hsa_148 | D04018 | 0 | hsa_2561 | D04243 | 0 | hsa_285242 | D09867 |
| 1 | hsa_3356 | D04034 | 0 | hsa_134 | D01685 | 0 | hsa_5140 | D01771 |
| 1 | hsa_3357 | D04034 | 0 | hsa_5144 | D08054 | 0 | hsa_1133 | D10556 |
| 1 | hsa_3358 | D04034 | 0 | hsa_2555 | D05339 | 0 | hsa_146 | D07217 |
| 1 | hsa_1813 | D04038 | 0 | hsa_200909 | D01447 | 0 | hsa_200909 | D08342 |
| 1 | hsa_1128 | D04057 | 0 | hsa_150 | D00685 | 0 | hsa_1132 | D09977 |
| 1 | hsa_1129 | D04057 | 0 | hsa_2915 | D00776 | 0 | hsa_5729 | D08074 |
| 1 | hsa_1131 | D04057 | 0 | hsa_135 | D02825 | 0 | hsa_3354 | D02676 |
| 1 | hsa_1241 | D04074 | 0 | hsa_2560 | D03889 | 0 | hsa_1901 | D00058 |
| 1 | hsa_56413 | D04074 | 0 | hsa_4543 | D08220 | 0 | hsa_6753 | D08432 |
| 1 | hsa_1128 | D04087 | 0 | hsa_64805 | D05037 | 0 | hsa_3062 | D05941 |
| 1 | hsa_1129 | D04087 | 0 | hsa_5731 | D08432 | 0 | hsa_2564 | D07931 |
| 1 | hsa_1131 | D04087 | 0 | hsa_5737 | D06574 | 0 | hsa_1241 | D10612 |
| 1 | hsa_1132 | D04087 | 0 | hsa_64805 | D05048 | 0 | hsa_3351 | D00723 |
| 1 | hsa_1133 | D04087 | 0 | hsa_285242 | D03494 | 0 | hsa_3061 | D02021 |
| 1 | hsa_3274 | D04098 | 0 | hsa_1132 | D01624 | 0 | hsa_2563 | D10628 |
| 1 | hsa_1128 | D04116 | 0 | hsa_2915 | D04074 | 0 | hsa_6915 | D07072 |
| 1 | hsa_1129 | D04116 | 0 | hsa_2556 | D09366 | 0 | hsa_151 | D02211 |
| 1 | hsa_1131 | D04116 | 0 | hsa_1813 | D04820 | 0 | hsa_2565 | D08160 |
| 1 | hsa_1132 | D04116 | 0 | hsa_2798 | D08856 | 0 | hsa_2562 | D04361 |
| 1 | hsa_1133 | D04116 | 0 | hsa_552 | D10330 | 0 | hsa_1133 | D02688 |
| 1 | hsa_6869 | D04122 | 0 | hsa_2693 | D01592 | 0 | hsa_2864 | D08257 |
| 1 | hsa_185 | D04126 | 0 | hsa_3061 | D07513 | 0 | hsa_1241 | D00662 |
| 1 | hsa_2915 | D04155 | 0 | hsa_3359 | D00573 | 0 | hsa_2915 | D07662 |
| 1 | hsa_154 | D04157 | 0 | hsa_151 | D09607 | 0 | hsa_3359 | D01481 |
| 1 | hsa_148 | D04184 | 0 | hsa_886 | D02983 | 0 | hsa_1128 | D08940 |
| 1 | hsa_1901 | D04187 | 0 | hsa_3061 | D00249 | 0 | hsa_3350 | D04087 |
| 1 | hsa_153 | D04190 | 0 | hsa_1128 | D01165 | 0 | hsa_155 | D09349 |
| 1 | hsa_150 | D04223 | 0 | hsa_2564 | D08105 | 0 | hsa_2567 | D08343 |
| 1 | hsa_151 | D04223 | 0 | hsa_2567 | D01182 | 0 | hsa_4987 | D06293 |
| 1 | hsa_152 | D04223 | 0 | hsa_5140 | D04494 | 0 | hsa_2912 | D00058 |
| 1 | hsa_185 | D04243 | 0 | hsa_5731 | D08148 | 0 | hsa_56413 | D02641 |
| 1 | hsa_3351 | D04264 | 0 | hsa_2565 | D07870 | 0 | hsa_2563 | D00778 |
| 1 | hsa_2798 | D04302 | 0 | hsa_3757 | D01263 | 0 | hsa_6532 | D09693 |
| 1 | hsa_3350 | D04314 | 0 | hsa_3269 | D04157 | 0 | hsa_5737 | D08205 |
| 1 | hsa_3356 | D04320 | 0 | hsa_2555 | D02200 | 0 | hsa_2558 | D06353 |
| 1 | hsa_3357 | D04320 | 0 | hsa_2913 | D03618 | 0 | hsa_1133 | D08645 |
| 1 | hsa_3358 | D04320 | 0 | hsa_80834 | D03535 | 0 | hsa_2563 | D02041 |
| 1 | hsa_2798 | D04361 | 0 | hsa_3354 | D03983 | 0 | hsa_1813 | D07862 |
| 1 | hsa_3359 | D04370 | 0 | hsa_135 | D00722 | 0 | hsa_5737 | D01004 |
| 1 | hsa_9177 | D04370 | 0 | hsa_2862 | D03269 | 0 | hsa_5729 | D08341 |
| 1 | hsa_170572 | D04370 | 0 | hsa_80834 | D01861 | 0 | hsa_6754 | D08106 |
| 1 | hsa_200909 | D04370 | 0 | hsa_2559 | D00025 | 0 | hsa_6755 | D02939 |
| 1 | hsa_285242 | D04370 | 0 | hsa_6870 | D08192 | 0 | hsa_2864 | D08325 |
| 1 | hsa_151 | D04375 | 0 | hsa_3357 | D10563 | 0 | hsa_4986 | D08207 |
| 1 | hsa_152 | D04375 | 0 | hsa_1129 | D08635 | 0 | hsa_3350 | D07590 |
| 1 | hsa_2798 | D04405 | 0 | hsa_6752 | D05587 | 0 | hsa_151 | D01143 |
| 1 | hsa_1128 | D04479 | 0 | hsa_151 | D01165 | 0 | hsa_3354 | D06495 |
| 1 | hsa_1129 | D04479 | 0 | hsa_135 | D01855 | 0 | hsa_6608 | D02964 |
| 1 | hsa_1131 | D04479 | 0 | hsa_3061 | D08693 | 0 | hsa_3357 | D10710 |
| 1 | hsa_1132 | D04479 | 0 | hsa_6753 | D03704 | 0 | hsa_2864 | D08382 |
| 1 | hsa_1133 | D04479 | 0 | hsa_3061 | D07931 | 0 | hsa_2563 | D00249 |
| 1 | hsa_1812 | D04488 | 0 | hsa_285242 | D08397 | 0 | hsa_152 | D00685 |
| 1 | hsa_624 | D04492 | 0 | hsa_6571 | D02417 | 0 | hsa_624 | D02682 |
| 1 | hsa_3269 | D04494 | 0 | hsa_170572 | D09766 | 0 | hsa_3274 | D07906 |
| 1 | hsa_2862 | D04497 | 0 | hsa_5144 | D02645 | 0 | hsa_3350 | D01051 |
| 1 | hsa_6915 | D04500 | 0 | hsa_64805 | D01360 | 0 | hsa_153 | D01373 |
| 1 | hsa_6915 | D04501 | 0 | hsa_2149 | D08636 | 0 | hsa_11255 | D09684 |
| 1 | hsa_10280 | D04502 | 0 | hsa_9568 | D06569 | 0 | hsa_9568 | D07667 |
| 1 | hsa_151 | D04514 | 0 | hsa_154 | D07662 | 0 | hsa_2560 | D01482 |
| 1 | hsa_3274 | D04517 | 0 | hsa_10800 | D01571 | 0 | hsa_10203 | D08636 |
| 1 | hsa_146 | D04531 | 0 | hsa_3350 | D08494 | 0 | hsa_3062 | D08146 |
| 1 | hsa_147 | D04531 | 0 | hsa_6752 | D07377 | 0 | hsa_1814 | D01801 |
| 1 | hsa_148 | D04531 | 0 | hsa_5144 | D01939 | 0 | hsa_3357 | D04611 |
| 1 | hsa_146 | D04532 | 0 | hsa_3359 | D05891 | 0 | hsa_4987 | D08473 |
| 1 | hsa_147 | D04532 | 0 | hsa_64805 | D03672 | 0 | hsa_5139 | D02245 |
| 1 | hsa_148 | D04532 | 0 | hsa_6532 | D07832 | 0 | hsa_2915 | D08241 |
| 1 | hsa_3350 | D04533 | 0 | hsa_154 | D02246 | 0 | hsa_10203 | D08045 |
| 1 | hsa_3351 | D04533 | 0 | hsa_2568 | D02775 | 0 | hsa_3356 | D04703 |
| 1 | hsa_3352 | D04533 | 0 | hsa_6571 | D03534 | 0 | hsa_2566 | D10735 |
| 1 | hsa_3354 | D04533 | 0 | hsa_5021 | D00721 | 0 | hsa_6344 | D08595 |
| 1 | hsa_3355 | D04533 | 0 | hsa_886 | D01315 | 0 | hsa_2550 | D10100 |
| 1 | hsa_3350 | D04611 | 0 | hsa_153 | D03009 | 0 | hsa_6608 | D08988 |
| 1 | hsa_154 | D04625 | 0 | hsa_2912 | D05196 | 0 | hsa_10203 | D07102 |
| 1 | hsa_154 | D04626 | 0 | hsa_185 | D01742 | 0 | hsa_1132 | D09997 |
| 1 | hsa_135 | D04641 | 0 | hsa_2566 | D05699 | 0 | hsa_6754 | D08235 |
| 1 | hsa_3359 | D04642 | 0 | hsa_9177 | D02234 | 0 | hsa_1394 | D02901 |
| 1 | hsa_9177 | D04642 | 0 | hsa_56413 | D09797 | 0 | hsa_2912 | D01373 |
| 1 | hsa_170572 | D04642 | 0 | hsa_5144 | D01902 | 0 | hsa_1133 | D05345 |
| 1 | hsa_200909 | D04642 | 0 | hsa_5141 | D10174 | 0 | hsa_10280 | D01624 |
| 1 | hsa_285242 | D04642 | 0 | hsa_200909 | D09981 | 0 | hsa_5734 | D09336 |
| 1 | hsa_4986 | D04649 | 0 | hsa_3357 | D08027 | 0 | hsa_2565 | D02100 |
| 1 | hsa_6751 | D04666 | 0 | hsa_5141 | D01182 | 0 | hsa_3356 | D05276 |
| 1 | hsa_3274 | D04680 | 0 | hsa_2563 | D00106 | 0 | hsa_624 | D08683 |
| 1 | hsa_3350 | D04683 | 0 | hsa_6754 | D05429 | 0 | hsa_2563 | D02939 |
| 1 | hsa_1813 | D04693 | 0 | hsa_6753 | D01172 | 0 | hsa_5731 | D03587 |
| 1 | hsa_1813 | D04694 | 0 | hsa_6754 | D00725 | 0 | hsa_155 | D08683 |
| 1 | hsa_154 | D04701 | 0 | hsa_3355 | D02719 | 0 | hsa_5729 | D02073 |
| 1 | hsa_154 | D04703 | 0 | hsa_9568 | D04693 | 0 | hsa_6571 | D02388 |
| 1 | hsa_153 | D04708 | 0 | hsa_2555 | D03216 | 0 | hsa_55879 | D09784 |
| 1 | hsa_153 | D04710 | 0 | hsa_2904 | D00686 | 0 | hsa_799 | D07072 |
| 1 | hsa_1268 | D04717 | 0 | hsa_4985 | D01369 | 0 | hsa_2556 | D09717 |
| 1 | hsa_5724 | D04724 | 0 | hsa_2913 | D08099 | 0 | hsa_2564 | D02004 |
| 1 | hsa_554 | D04752 | 0 | hsa_799 | D07125 | 0 | hsa_3274 | D00605 |
| 1 | hsa_4988 | D04764 | 0 | hsa_59340 | D01573 | 0 | hsa_2904 | D07482 |
| 1 | hsa_151 | D04765 | 0 | hsa_152 | D08864 | 0 | hsa_4889 | D05730 |
| 1 | hsa_3356 | D04788 | 0 | hsa_9568 | D00774 | 0 | hsa_1132 | D01648 |
| 1 | hsa_3274 | D04794 | 0 | hsa_3362 | D10662 | 0 | hsa_799 | D02248 |
| 1 | hsa_3350 | D04820 | 0 | hsa_2561 | D08856 | 0 | hsa_2560 | D03051 |
| 1 | hsa_2798 | D04823 | 0 | hsa_2915 | D02576 | 0 | hsa_6344 | D06103 |
| 1 | hsa_6869 | D04860 | 0 | hsa_59340 | D06405 | 0 | hsa_2556 | D02738 |
| 1 | hsa_1128 | D04868 | 0 | hsa_5021 | D00723 | 0 | hsa_200909 | D08495 |
| 1 | hsa_1129 | D04868 | 0 | hsa_3356 | D01939 | 0 | hsa_2565 | D09607 |
| 1 | hsa_1131 | D04868 | 0 | hsa_55879 | D03214 | 0 | hsa_2555 | D00076 |
| 1 | hsa_1132 | D04868 | 0 | hsa_846 | D00835 | 0 | hsa_2564 | D03290 |
| 1 | hsa_1133 | D04868 | 0 | hsa_6571 | D00511 | 0 | hsa_155 | D02396 |
| 1 | hsa_150 | D04883 | 0 | hsa_1812 | D04703 | 0 | hsa_9568 | D00723 |
| 1 | hsa_151 | D04883 | 0 | hsa_1815 | D04868 | 0 | hsa_10280 | D00045 |
| 1 | hsa_152 | D04883 | 0 | hsa_3357 | D04820 | 0 | hsa_5141 | D01469 |
| 1 | hsa_153 | D04888 | 0 | hsa_2904 | D01175 | 0 | hsa_3362 | D09349 |
| 1 | hsa_153 | D04889 | 0 | hsa_5140 | D08074 | 0 | hsa_6570 | D04683 |
| 1 | hsa_4988 | D04924 | 0 | hsa_56413 | D00668 | 0 | hsa_5732 | D02849 |
| 1 | hsa_1813 | D04965 | 0 | hsa_2563 | D06157 | 0 | hsa_6751 | D02682 |
| 1 | hsa_1128 | D04970 | 0 | hsa_10203 | D05007 | 0 | hsa_1133 | D07821 |
| 1 | hsa_1129 | D04970 | 0 | hsa_3061 | D01204 | 0 | hsa_624 | D08500 |
| 1 | hsa_1131 | D04970 | 0 | hsa_2915 | D05699 | 0 | hsa_2562 | D10128 |
| 1 | hsa_1132 | D04970 | 0 | hsa_2904 | D01175 | 0 | hsa_185 | D08500 |
| 1 | hsa_1133 | D04970 | 0 | hsa_6870 | D10219 | 0 | hsa_624 | D01469 |
| 1 | hsa_4988 | D04973 | 0 | hsa_6753 | D08253 | 0 | hsa_1133 | D09388 |
| 1 | hsa_3274 | D05004 | 0 | hsa_1812 | D07990 | 0 | hsa_3757 | D00374 |
| 1 | hsa_4985 | D05007 | 0 | hsa_2564 | D08233 | 0 | hsa_1909 | D00843 |
| 1 | hsa_1813 | D05008 | 0 | hsa_1812 | D05196 | 0 | hsa_152 | D08497 |
| 1 | hsa_1813 | D05010 | 0 | hsa_5737 | D09732 | 0 | hsa_1241 | D08850 |
| 1 | hsa_153 | D05011 | 0 | hsa_1241 | D02108 | 0 | hsa_5142 | D10117 |
| 1 | hsa_3356 | D05015 | 0 | hsa_151 | D00644 | 0 | hsa_151 | D08123 |
| 1 | hsa_3357 | D05015 | 0 | hsa_6608 | D09994 | 0 | hsa_2554 | D10424 |
| 1 | hsa_3358 | D05015 | 0 | hsa_5021 | D01462 | 0 | hsa_886 | D10362 |
| 1 | hsa_1128 | D05035 | 0 | hsa_1394 | D04820 | 0 | hsa_2555 | D09363 |
| 1 | hsa_1129 | D05035 | 0 | hsa_1812 | D06147 | 0 | hsa_1268 | D10566 |
| 1 | hsa_1131 | D05035 | 0 | hsa_5021 | D05690 | 0 | hsa_2561 | D08045 |
| 1 | hsa_1132 | D05035 | 0 | hsa_2693 | D02227 | 0 | hsa_886 | D01902 |
| 1 | hsa_1133 | D05035 | 0 | hsa_6753 | D08864 | 0 | hsa_3274 | D00101 |
| 1 | hsa_154 | D05037 | 0 | hsa_3355 | D08397 | 0 | hsa_6870 | D08525 |
| 1 | hsa_4988 | D05048 | 0 | hsa_2862 | D05711 | 0 | hsa_1129 | D03156 |
| 1 | hsa_2862 | D05053 | 0 | hsa_10800 | D02004 | 0 | hsa_6532 | D07682 |
| 1 | hsa_1241 | D05085 | 0 | hsa_5729 | D02090 | 0 | hsa_1129 | D03814 |
| 1 | hsa_56413 | D05085 | 0 | hsa_5142 | D02976 | 0 | hsa_10800 | D02836 |
| 1 | hsa_1268 | D05099 | 0 | hsa_134 | D02363 | 0 | hsa_146 | D07946 |
| 1 | hsa_3356 | D05107 | 0 | hsa_1813 | D08376 | 0 | hsa_624 | D07660 |
| 1 | hsa_4988 | D05111 | 0 | hsa_6532 | D01482 | 0 | hsa_1909 | D05127 |
| 1 | hsa_153 | D05127 | 0 | hsa_2558 | D00793 | 0 | hsa_3757 | D04611 |
| 1 | hsa_3269 | D05196 | 0 | hsa_43 | D00502 | 0 | hsa_1268 | D05879 |
| 1 | hsa_148 | D05206 | 0 | hsa_6869 | D01891 | 0 | hsa_6571 | D01831 |
| 1 | hsa_147 | D05206 | 0 | hsa_59340 | D03102 | 0 | hsa_2558 | D02968 |
| 1 | hsa_146 | D05206 | 0 | hsa_5143 | D00498 | 0 | hsa_6915 | D08045 |
| 1 | hsa_4988 | D05217 | 0 | hsa_3359 | D03267 | 0 | hsa_5724 | D00685 |
| 1 | hsa_6751 | D05230 | 0 | hsa_55879 | D01172 | 0 | hsa_2904 | D05660 |
| 1 | hsa_1128 | D05276 | 0 | hsa_185 | D04680 | 0 | hsa_886 | D02234 |
| 1 | hsa_154 | D05277 | 0 | hsa_2564 | D01017 | 0 | hsa_6752 | D00685 |
| 1 | hsa_3274 | D05306 | 0 | hsa_3358 | D02271 | 0 | hsa_3357 | D07958 |
| 1 | hsa_3274 | D05307 | 0 | hsa_6869 | D04006 | 0 | hsa_7201 | D10545 |
| 1 | hsa_4988 | D05312 | 0 | hsa_3757 | D08693 | 0 | hsa_55879 | D08441 |
| 1 | hsa_3356 | D05339 | 0 | hsa_2693 | D08623 | 0 | hsa_155 | D03506 |
| 1 | hsa_3356 | D05340 | 0 | hsa_1129 | D10562 | 0 | hsa_3062 | D05667 |
| 1 | hsa_3359 | D05343 | 0 | hsa_153 | D07511 | 0 | hsa_5021 | D04492 |
| 1 | hsa_9177 | D05343 | 0 | hsa_2554 | D03170 | 0 | hsa_59340 | D04794 |
| 1 | hsa_170572 | D05343 | 0 | hsa_2864 | D00717 | 0 | hsa_6752 | D01317 |
| 1 | hsa_200909 | D05343 | 0 | hsa_151 | D04889 | 0 | hsa_1815 | D02396 |
| 1 | hsa_285242 | D05343 | 0 | hsa_4985 | D07513 | 0 | hsa_2562 | D05307 |
| 1 | hsa_153 | D05345 | 0 | hsa_4987 | D01976 | 0 | hsa_2567 | D02236 |
| 1 | hsa_154 | D05366 | 0 | hsa_2912 | D03982 | 0 | hsa_200909 | D07156 |
| 1 | hsa_3356 | D05395 | 0 | hsa_1268 | D09765 | 0 | hsa_11255 | D01000 |
| 1 | hsa_3357 | D05395 | 0 | hsa_2555 | D08661 | 0 | hsa_1812 | D08358 |
| 1 | hsa_3358 | D05395 | 0 | hsa_80834 | D06405 | 0 | hsa_624 | D09017 |
| 1 | hsa_134 | D05429 | 0 | hsa_2560 | D10430 | 0 | hsa_2561 | D05718 |
| 1 | hsa_4988 | D05462 | 0 | hsa_80834 | D08353 | 0 | hsa_1268 | D08397 |
| 1 | hsa_3360 | D05471 | 0 | hsa_2915 | D00058 | 0 | hsa_2558 | D09362 |
| 1 | hsa_154 | D05476 | 0 | hsa_5734 | D07259 | 0 | hsa_2912 | D08132 |
| 1 | hsa_1128 | D05478 | 0 | hsa_1814 | D00847 | 0 | hsa_846 | D07475 |
| 1 | hsa_1129 | D05478 | 0 | hsa_2568 | D05939 | 0 | hsa_2564 | D08638 |
| 1 | hsa_1131 | D05478 | 0 | hsa_799 | D00686 | 0 | hsa_6344 | D00843 |
| 1 | hsa_1132 | D05478 | 0 | hsa_5724 | D04710 | 0 | hsa_3351 | D08236 |
| 1 | hsa_1133 | D05478 | 0 | hsa_200909 | D08027 | 0 | hsa_5142 | D05035 |
| 1 | hsa_3356 | D05523 | 0 | hsa_10800 | D05395 | 0 | hsa_3351 | D02645 |
| 1 | hsa_1813 | D05575 | 0 | hsa_3357 | D08443 | 0 | hsa_2912 | D05575 |
| 1 | hsa_153 | D05587 | 0 | hsa_1133 | D06330 | 0 | hsa_6753 | D01467 |
| 1 | hsa_64805 | D05597 | 0 | hsa_2555 | D02613 | 0 | hsa_4988 | D01551 |
| 1 | hsa_153 | D05606 | 0 | hsa_6752 | D08629 | 0 | hsa_5021 | D07482 |
| 1 | hsa_4988 | D05632 | 0 | hsa_11255 | D10001 | 0 | hsa_1901 | D08611 |
| 1 | hsa_146 | D05649 | 0 | hsa_146 | D09990 | 0 | hsa_3354 | D05099 |
| 1 | hsa_147 | D05649 | 0 | hsa_6570 | D08091 | 0 | hsa_170572 | D00479 |
| 1 | hsa_148 | D05649 | 0 | hsa_2904 | D02824 | 0 | hsa_6571 | D07838 |
| 1 | hsa_3269 | D05660 | 0 | hsa_59340 | D02599 | 0 | hsa_1394 | D03809 |
| 1 | hsa_4986 | D05667 | 0 | hsa_3274 | D04122 | 0 | hsa_5141 | D07522 |
| 1 | hsa_1813 | D05677 | 0 | hsa_2864 | D02836 | 0 | hsa_6571 | D01478 |
| 1 | hsa_1813 | D05682 | 0 | hsa_3360 | D03399 | 0 | hsa_10800 | D09344 |
| 1 | hsa_3359 | D05685 | 0 | hsa_6869 | D02738 | 0 | hsa_3274 | D00138 |
| 1 | hsa_9177 | D05685 | 0 | hsa_2862 | D00756 | 0 | hsa_6532 | D09535 |
| 1 | hsa_170572 | D05685 | 0 | hsa_5724 | D02612 | 0 | hsa_2561 | D04488 |
| 1 | hsa_200909 | D05685 | 0 | hsa_10280 | D08838 | 0 | hsa_1129 | D02363 |
| 1 | hsa_285242 | D05685 | 0 | hsa_3359 | D09608 | 0 | hsa_3061 | D08425 |
| 1 | hsa_1813 | D05690 | 0 | hsa_2561 | D10735 | 0 | hsa_154 | D10006 |
| 1 | hsa_154 | D05691 | 0 | hsa_2564 | D07183 | 0 | hsa_151 | D10128 |
| 1 | hsa_3274 | D05699 | 0 | hsa_1909 | D07460 | 0 | hsa_2561 | D09982 |
| 1 | hsa_135 | D05711 | 0 | hsa_3061 | D01674 | 0 | hsa_285242 | D10729 |
| 1 | hsa_154 | D05718 | 0 | hsa_1129 | D06268 | 0 | hsa_5143 | D01806 |
| 1 | hsa_6915 | D05727 | 0 | hsa_3350 | D02349 | 0 | hsa_2915 | D03504 |
| 1 | hsa_154 | D05730 | 0 | hsa_554 | D00720 | 0 | hsa_64805 | D03825 |
| 1 | hsa_1268 | D05731 | 0 | hsa_7201 | D00756 | 0 | hsa_135 | D00835 |
| 1 | hsa_5732 | D05732 | 0 | hsa_1815 | D01044 | 0 | hsa_5021 | D00403 |
| 1 | hsa_3356 | D05738 | 0 | hsa_151 | D01360 | 0 | hsa_2555 | D02577 |
| 1 | hsa_3269 | D05742 | 0 | hsa_3351 | D07125 | 0 | hsa_1394 | D07522 |
| 1 | hsa_1813 | D05768 | 0 | hsa_624 | D02227 | 0 | hsa_80834 | D06140 |
| 1 | hsa_1128 | D05779 | 0 | hsa_2560 | D09693 | 0 | hsa_4157 | D08300 |
| 1 | hsa_1129 | D05779 | 0 | hsa_846 | D08587 | 0 | hsa_6571 | D04788 |
| 1 | hsa_1131 | D05779 | 0 | hsa_5144 | D08041 | 0 | hsa_2565 | D01830 |
| 1 | hsa_1132 | D05779 | 0 | hsa_1132 | D08449 | 0 | hsa_155 | D06293 |
| 1 | hsa_1133 | D05779 | 0 | hsa_554 | D10692 | 0 | hsa_43 | D00079 |
| 1 | hsa_80834 | D05781 | 0 | hsa_2562 | D00138 | 0 | hsa_200909 | D08649 |
| 1 | hsa_3350 | D05804 | 0 | hsa_153 | D08594 | 0 | hsa_11255 | D00403 |
| 1 | hsa_134 | D05818 | 0 | hsa_135 | D04965 | 0 | hsa_10280 | D10567 |
| 1 | hsa_154 | D05832 | 0 | hsa_4889 | D09845 | 0 | hsa_6571 | D03415 |
| 1 | hsa_155 | D05879 | 0 | hsa_9568 | D03502 | 0 | hsa_3757 | D01315 |
| 1 | hsa_1815 | D05891 | 0 | hsa_153 | D07077 | 0 | hsa_2567 | D05996 |
| 1 | hsa_154 | D05902 | 0 | hsa_1901 | D03495 | 0 | hsa_3274 | D01177 |
| 1 | hsa_4986 | D05907 | 0 | hsa_2562 | D04122 | 0 | hsa_55879 | D06374 |
| 1 | hsa_3274 | D05939 | 0 | hsa_2566 | D04018 | 0 | hsa_1813 | D10545 |
| 1 | hsa_3360 | D05941 | 0 | hsa_7201 | D02211 | 0 | hsa_154 | D04611 |
| 1 | hsa_6915 | D05970 | 0 | hsa_10800 | D08035 | 0 | hsa_3061 | D07560 |
| 1 | hsa_6870 | D05996 | 0 | hsa_9177 | D01879 | 0 | hsa_64805 | D08041 |
| 1 | hsa_1128 | D06000 | 0 | hsa_6344 | D00840 | 0 | hsa_2559 | D07101 |
| 1 | hsa_4988 | D06007 | 0 | hsa_1394 | D01571 | 0 | hsa_6753 | D04641 |
| 1 | hsa_1128 | D06015 | 0 | hsa_3351 | D04243 | 0 | hsa_6344 | D01520 |
| 1 | hsa_1129 | D06015 | 0 | hsa_6344 | D08148 | 0 | hsa_147 | D01978 |
| 1 | hsa_1131 | D06015 | 0 | hsa_2554 | D06552 | 0 | hsa_2554 | D08239 |
| 1 | hsa_1132 | D06015 | 0 | hsa_3354 | D00668 | 0 | hsa_799 | D07550 |
| 1 | hsa_1133 | D06015 | 0 | hsa_5021 | D01469 | 0 | hsa_4985 | D08322 |
| 1 | hsa_134 | D06019 | 0 | hsa_152 | D04860 | 0 | hsa_6344 | D03214 |
| 1 | hsa_3269 | D06021 | 0 | hsa_5729 | D10710 | 0 | hsa_1814 | D05053 |
| 1 | hsa_3269 | D06063 | 0 | hsa_170572 | D10336 | 0 | hsa_6570 | D07493 |
| 1 | hsa_134 | D06103 | 0 | hsa_1132 | D10725 | 0 | hsa_3269 | D02576 |
| 1 | hsa_134 | D06104 | 0 | hsa_3350 | D10663 | 0 | hsa_1813 | D03269 |
| 1 | hsa_1241 | D06140 | 0 | hsa_6870 | D08611 | 0 | hsa_2558 | D01386 |
| 1 | hsa_56413 | D06140 | 0 | hsa_1812 | D10391 | 0 | hsa_1132 | D09977 |
| 1 | hsa_4988 | D06147 | 0 | hsa_2915 | D07122 | 0 | hsa_3356 | D09949 |
| 1 | hsa_3274 | D06157 | 0 | hsa_2862 | D00498 | 0 | hsa_4889 | D08148 |
| 1 | hsa_5739 | D06213 | 0 | hsa_3355 | D09570 | 0 | hsa_2568 | D00776 |
| 1 | hsa_146 | D06234 | 0 | hsa_2558 | D01085 | 0 | hsa_5021 | D08449 |
| 1 | hsa_147 | D06234 | 0 | hsa_1129 | D09318 | 0 | hsa_6870 | D05660 |
| 1 | hsa_148 | D06234 | 0 | hsa_170572 | D03982 | 0 | hsa_1394 | D00663 |
| 1 | hsa_3269 | D06246 | 0 | hsa_1133 | D03494 | 0 | hsa_154 | D06253 |
| 1 | hsa_2798 | D06247 | 0 | hsa_11255 | D01806 | 0 | hsa_6754 | D03008 |
| 1 | hsa_2798 | D06248 | 0 | hsa_846 | D01548 | 0 | hsa_43 | D07795 |
| 1 | hsa_3356 | D06253 | 0 | hsa_3352 | D01485 | 0 | hsa_1815 | D09335 |
| 1 | hsa_3357 | D06253 | 0 | hsa_6608 | D07129 | 0 | hsa_1268 | D01021 |
| 1 | hsa_3358 | D06253 | 0 | hsa_3062 | D00839 | 0 | hsa_6755 | D01635 |
| 1 | hsa_4988 | D06268 | 0 | hsa_5139 | D08229 | 0 | hsa_7201 | D01512 |
| 1 | hsa_1128 | D06273 | 0 | hsa_1901 | D05011 | 0 | hsa_3062 | D00479 |
| 1 | hsa_1131 | D06273 | 0 | hsa_1129 | D02357 | 0 | hsa_64805 | D07183 |
| 1 | hsa_5737 | D06274 | 0 | hsa_6753 | D09997 | 0 | hsa_6344 | D01101 |
| 1 | hsa_6915 | D06280 | 0 | hsa_6751 | D07537 | 0 | hsa_6532 | D09990 |
| 1 | hsa_6751 | D06281 | 0 | hsa_2568 | D08569 | 0 | hsa_5737 | D05730 |
| 1 | hsa_6869 | D06293 | 0 | hsa_6751 | D07660 | 0 | hsa_4986 | D07522 |
| 1 | hsa_6869 | D06317 | 0 | hsa_2562 | D03814 | 0 | hsa_43 | D07831 |
| 1 | hsa_153 | D06328 | 0 | hsa_9177 | D07550 | 0 | hsa_3359 | D10199 |
| 1 | hsa_153 | D06329 | 0 | hsa_1813 | D02208 | 0 | hsa_846 | D04703 |
| 1 | hsa_1128 | D06330 | 0 | hsa_285242 | D05107 | 0 | hsa_3352 | D06374 |
| 1 | hsa_1132 | D06330 | 0 | hsa_3355 | D00661 | 0 | hsa_3360 | D02100 |
| 1 | hsa_1128 | D06331 | 0 | hsa_799 | D00081 | 0 | hsa_5734 | D06273 |
| 1 | hsa_1132 | D06331 | 0 | hsa_6752 | D08425 | 0 | hsa_147 | D00502 |
| 1 | hsa_150 | D06344 | 0 | hsa_56413 | D10436 | 0 | hsa_4986 | D08241 |
| 1 | hsa_151 | D06344 | 0 | hsa_2564 | D04649 | 0 | hsa_3354 | D02705 |
| 1 | hsa_152 | D06344 | 0 | hsa_6915 | D10185 | 0 | hsa_185 | D02775 |
| 1 | hsa_3360 | D06353 | 0 | hsa_2563 | D07124 | 0 | hsa_4986 | D08983 |
| 1 | hsa_3274 | D06355 | 0 | hsa_554 | D08482 | 0 | hsa_135 | D00644 |
| 1 | hsa_154 | D06374 | 0 | hsa_3362 | D08474 | 0 | hsa_1132 | D09990 |
| 1 | hsa_148 | D06394 | 0 | hsa_3355 | D01025 | 0 | hsa_80834 | D02834 |
| 1 | hsa_4986 | D06395 | 0 | hsa_6754 | D04924 | 0 | hsa_153 | D10118 |
| 1 | hsa_4986 | D06405 | 0 | hsa_4987 | D02102 | 0 | hsa_134 | D05345 |
| 1 | hsa_6751 | D06495 | 0 | hsa_7201 | D02208 | 0 | hsa_3356 | D07765 |
| 1 | hsa_3269 | D06552 | 0 | hsa_2568 | D10736 | 0 | hsa_6915 | D08638 |
| 1 | hsa_3350 | D06566 | 0 | hsa_2693 | D07217 | 0 | hsa_9177 | D09954 |
| 1 | hsa_3350 | D06567 | 0 | hsa_2560 | D09765 | 0 | hsa_3358 | D04625 |
| 1 | hsa_4157 | D06569 | 0 | hsa_1131 | D00671 | 0 | hsa_2556 | D07460 |
| 1 | hsa_6869 | D06574 | 0 | hsa_10280 | D05575 | 0 | hsa_152 | D01438 |
| 1 | hsa_6869 | D06597 | 0 | hsa_2149 | D04074 | 0 | hsa_6344 | D09366 |
| 1 | hsa_3358 | D06613 | 0 | hsa_185 | D08355 | 0 | hsa_5724 | D10117 |
| 1 | hsa_4988 | D06618 | 0 | hsa_3351 | D00644 | 0 | hsa_6753 | D02666 |
| 1 | hsa_153 | D06622 | 0 | hsa_6753 | D00847 | 0 | hsa_5139 | D08300 |
| 1 | hsa_3356 | D06623 | 0 | hsa_4889 | D10001 | 0 | hsa_1812 | D09953 |
| 1 | hsa_3356 | D06632 | 0 | hsa_1132 | D04883 | 0 | hsa_1268 | D03060 |
| 1 | hsa_3356 | D06633 | 0 | hsa_2912 | D07684 | 0 | hsa_6532 | D01855 |
| 1 | hsa_153 | D06646 | 0 | hsa_2149 | D08648 | 0 | hsa_148 | D07765 |
| 1 | hsa_10800 | D06659 | 0 | hsa_148 | D02683 | 0 | hsa_10800 | D00366 |
| 1 | hsa_3358 | D06660 | 0 | hsa_3355 | D10663 | 0 | hsa_5141 | D05277 |
| 1 | hsa_150 | D06671 | 0 | hsa_1815 | D08157 | 0 | hsa_2864 | D08057 |
| 1 | hsa_151 | D06671 | 0 | hsa_1909 | D04764 | 0 | hsa_3359 | D05217 |
| 1 | hsa_152 | D06671 | 0 | hsa_5734 | D03982 | 0 | hsa_4988 | D09336 |
| 1 | hsa_552 | D06672 | 0 | hsa_6869 | D02212 | 0 | hsa_9568 | D08850 |
| 1 | hsa_3274 | D07072 | 0 | hsa_151 | D07593 | 0 | hsa_185 | D08318 |
| 1 | hsa_1131 | D07076 | 0 | hsa_886 | D08078 | 0 | hsa_5142 | D01500 |
| 1 | hsa_1909 | D07077 | 0 | hsa_2864 | D02151 | 0 | hsa_170572 | D02724 |
| 1 | hsa_1128 | D07099 | 0 | hsa_1901 | D04820 | 0 | hsa_1128 | D07977 |
| 1 | hsa_1129 | D07099 | 0 | hsa_6915 | D01399 | 0 | hsa_2864 | D08662 |
| 1 | hsa_1131 | D07099 | 0 | hsa_2559 | D09608 | 0 | hsa_146 | D05742 |
| 1 | hsa_1132 | D07099 | 0 | hsa_3757 | D08635 | 0 | hsa_10280 | D04264 |
| 1 | hsa_1133 | D07099 | 0 | hsa_4543 | D10129 | 0 | hsa_3757 | D00101 |
| 1 | hsa_1813 | D07101 | 0 | hsa_185 | D06394 | 0 | hsa_10800 | D01492 |
| 1 | hsa_1813 | D07102 | 0 | hsa_1814 | D02101 | 0 | hsa_5143 | D08687 |
| 1 | hsa_4988 | D07113 | 0 | hsa_10203 | D08384 | 0 | hsa_5731 | D04626 |
| 1 | hsa_4988 | D07122 | 0 | hsa_3352 | D07286 | 0 | hsa_3354 | D08241 |
| 1 | hsa_148 | D07124 | 0 | hsa_4889 | D04116 | 0 | hsa_2693 | D07684 |
| 1 | hsa_147 | D07124 | 0 | hsa_3757 | D04765 | 0 | hsa_11255 | D06569 |
| 1 | hsa_146 | D07124 | 0 | hsa_2568 | D06394 | 0 | hsa_6755 | D07831 |
| 1 | hsa_3269 | D07125 | 0 | hsa_11255 | D00456 | 0 | hsa_1901 | D01741 |
| 1 | hsa_3359 | D07129 | 0 | hsa_1909 | D08365 | 0 | hsa_6608 | D03008 |
| 1 | hsa_9177 | D07129 | 0 | hsa_6753 | D02109 | 0 | hsa_4986 | D01025 |
| 1 | hsa_170572 | D07129 | 0 | hsa_5737 | D09362 | 0 | hsa_2561 | D01902 |
| 1 | hsa_200909 | D07129 | 0 | hsa_5139 | D00677 | 0 | hsa_5734 | D08293 |
| 1 | hsa_285242 | D07129 | 0 | hsa_5737 | D08940 | 0 | hsa_5731 | D10180 |
| 1 | hsa_6915 | D07140 | 0 | hsa_1133 | D02626 | 0 | hsa_799 | D04794 |
| 1 | hsa_146 | D07148 | 0 | hsa_59340 | D02622 | 0 | hsa_3351 | D02568 |
| 1 | hsa_147 | D07148 | 0 | hsa_5144 | D03415 | 0 | hsa_2862 | D05217 |
| 1 | hsa_148 | D07148 | 0 | hsa_200909 | D08235 | 0 | hsa_3358 | D01929 |
| 1 | hsa_153 | D07156 | 0 | hsa_10800 | D01891 | 0 | hsa_3351 | D01815 |
| 1 | hsa_1909 | D07171 | 0 | hsa_1909 | D07489 | 0 | hsa_64805 | D07931 |
| 1 | hsa_3359 | D07175 | 0 | hsa_80834 | D04708 | 0 | hsa_3358 | D07198 |
| 1 | hsa_9177 | D07175 | 0 | hsa_5729 | D08639 | 0 | hsa_4543 | D08850 |
| 1 | hsa_170572 | D07175 | 0 | hsa_5734 | D03399 | 0 | hsa_3274 | D09774 |
| 1 | hsa_200909 | D07175 | 0 | hsa_2568 | D05606 | 0 | hsa_1909 | D00791 |
| 1 | hsa_285242 | D07175 | 0 | hsa_2557 | D03825 | 0 | hsa_1132 | D08923 |
| 1 | hsa_153 | D07181 | 0 | hsa_6532 | D05939 | 0 | hsa_2562 | D10371 |
| 1 | hsa_153 | D07182 | 0 | hsa_2560 | D01891 | 0 | hsa_1241 | D05682 |
| 1 | hsa_153 | D07183 | 0 | hsa_2559 | D09766 | 0 | hsa_2915 | D02964 |
| 1 | hsa_153 | D07184 | 0 | hsa_152 | D02281 | 0 | hsa_2862 | D08123 |
| 1 | hsa_3269 | D07197 | 0 | hsa_10203 | D05048 | 0 | hsa_1813 | D10677 |
| 1 | hsa_3269 | D07198 | 0 | hsa_1815 | D08838 | 0 | hsa_134 | D10703 |
| 1 | hsa_1813 | D07217 | 0 | hsa_4543 | D08160 | 0 | hsa_59340 | D07853 |
| 1 | hsa_3350 | D07218 | 0 | hsa_6608 | D08355 | 0 | hsa_2554 | D07543 |
| 1 | hsa_3351 | D07218 | 0 | hsa_55879 | D03618 | 0 | hsa_80834 | D01830 |
| 1 | hsa_3352 | D07218 | 0 | hsa_146 | D03399 | 0 | hsa_3355 | D08121 |
| 1 | hsa_3354 | D07218 | 0 | hsa_6570 | D08623 | 0 | hsa_4985 | D02641 |
| 1 | hsa_3355 | D07218 | 0 | hsa_2864 | D02767 | 0 | hsa_6755 | D02281 |
| 1 | hsa_1128 | D07226 | 0 | hsa_43 | D07175 | 0 | hsa_3062 | D08249 |
| 1 | hsa_1129 | D07226 | 0 | hsa_3351 | D02995 | 0 | hsa_6570 | D09992 |
| 1 | hsa_1131 | D07226 | 0 | hsa_64805 | D02071 | 0 | hsa_1241 | D00371 |
| 1 | hsa_1132 | D07226 | 0 | hsa_2904 | D08047 | 0 | hsa_2565 | D08856 |
| 1 | hsa_1133 | D07226 | 0 | hsa_2563 | D09717 | 0 | hsa_624 | D08027 |
| 1 | hsa_552 | D07227 | 0 | hsa_80834 | D01085 | 0 | hsa_4986 | D07550 |
| 1 | hsa_5021 | D07228 | 0 | hsa_2558 | D07946 | 0 | hsa_3757 | D10391 |
| 1 | hsa_5021 | D07229 | 0 | hsa_153 | D00723 | 0 | hsa_554 | D09335 |
| 1 | hsa_2798 | D07259 | 0 | hsa_1241 | D02372 | 0 | hsa_3355 | D05008 |
| 1 | hsa_4988 | D07286 | 0 | hsa_2912 | D01051 | 0 | hsa_6751 | D01830 |
| 1 | hsa_1813 | D07305 | 0 | hsa_2561 | D07890 | 0 | hsa_886 | D04717 |
| 1 | hsa_1813 | D07312 | 0 | hsa_6754 | D09008 | 0 | hsa_200909 | D00559 |
| 1 | hsa_1128 | D07349 | 0 | hsa_5142 | D04883 | 0 | hsa_2862 | D05462 |
| 1 | hsa_1129 | D07349 | 0 | hsa_5732 | D02022 | 0 | hsa_2912 | D01685 |
| 1 | hsa_1131 | D07349 | 0 | hsa_1131 | D00482 | 0 | hsa_3062 | D07831 |
| 1 | hsa_1132 | D07349 | 0 | hsa_6754 | D00663 | 0 | hsa_2798 | D02681 |
| 1 | hsa_1133 | D07349 | 0 | hsa_6570 | D02876 | 0 | hsa_6870 | D00646 |
| 1 | hsa_154 | D07377 | 0 | hsa_2566 | D01369 | 0 | hsa_80834 | D08300 |
| 1 | hsa_3269 | D07398 | 0 | hsa_2566 | D01987 | 0 | hsa_4543 | D00672 |
| 1 | hsa_3269 | D07402 | 0 | hsa_151 | D05343 | 0 | hsa_3274 | D10185 |
| 1 | hsa_3269 | D07406 | 0 | hsa_155 | D03504 | 0 | hsa_64805 | D07937 |
| 1 | hsa_3269 | D07407 | 0 | hsa_3359 | D05008 | 0 | hsa_1901 | D02590 |
| 1 | hsa_146 | D07451 | 0 | hsa_147 | D05127 | 0 | hsa_3355 | D02101 |
| 1 | hsa_147 | D07451 | 0 | hsa_1268 | D07821 | 0 | hsa_6344 | D10563 |
| 1 | hsa_148 | D07451 | 0 | hsa_3274 | D07465 | 0 | hsa_153 | D05099 |
| 1 | hsa_3269 | D07458 | 0 | hsa_3358 | D01801 | 0 | hsa_148 | D04973 |
| 1 | hsa_3269 | D07459 | 0 | hsa_5724 | D10152 | 0 | hsa_1129 | D03051 |
| 1 | hsa_1812 | D07460 | 0 | hsa_6869 | D02369 | 0 | hsa_2558 | D01269 |
| 1 | hsa_150 | D07461 | 0 | hsa_2567 | D05037 | 0 | hsa_3062 | D03170 |
| 1 | hsa_151 | D07461 | 0 | hsa_64805 | D08560 | 0 | hsa_2904 | D01573 |
| 1 | hsa_152 | D07461 | 0 | hsa_3352 | D00540 | 0 | hsa_4987 | D08861 |
| 1 | hsa_154 | D07463 | 0 | hsa_2559 | D00610 | 0 | hsa_1133 | D05111 |
| 1 | hsa_146 | D07465 | 0 | hsa_5140 | D01491 | 0 | hsa_134 | D08424 |
| 1 | hsa_147 | D07465 | 0 | hsa_4889 | D00600 | 0 | hsa_5141 | D08192 |
| 1 | hsa_148 | D07465 | 0 | hsa_3062 | D08229 | 0 | hsa_64805 | D04122 |
| 1 | hsa_5021 | D07475 | 0 | hsa_2555 | D00605 | 0 | hsa_4986 | D00147 |
| 1 | hsa_3269 | D07482 | 0 | hsa_4543 | D05879 | 0 | hsa_3269 | D08983 |
| 1 | hsa_3269 | D07483 | 0 | hsa_2693 | D02577 | 0 | hsa_148 | D01771 |
| 1 | hsa_154 | D07489 | 0 | hsa_2550 | D00573 | 0 | hsa_6869 | D01453 |
| 1 | hsa_134 | D07491 | 0 | hsa_3062 | D07662 | 0 | hsa_2915 | D02612 |
| 1 | hsa_3269 | D07492 | 0 | hsa_3351 | D01801 | 0 | hsa_5140 | D07072 |
| 1 | hsa_3269 | D07493 | 0 | hsa_2562 | D00502 | 0 | hsa_1133 | D00989 |
| 1 | hsa_153 | D07496 | 0 | hsa_59340 | D01806 | 0 | hsa_153 | D09697 |
| 1 | hsa_1128 | D07511 | 0 | hsa_846 | D09362 | 0 | hsa_2554 | D04924 |
| 1 | hsa_1129 | D07511 | 0 | hsa_5142 | D01213 | 0 | hsa_5144 | D10119 |
| 1 | hsa_1131 | D07511 | 0 | hsa_5141 | D06374 | 0 | hsa_1133 | D03274 |
| 1 | hsa_1132 | D07511 | 0 | hsa_185 | D10563 | 0 | hsa_147 | D00796 |
| 1 | hsa_1133 | D07511 | 0 | hsa_1132 | D02893 | 0 | hsa_886 | D02130 |
| 1 | hsa_1128 | D07513 | 0 | hsa_1909 | D02102 | 0 | hsa_4987 | D02245 |
| 1 | hsa_1129 | D07513 | 0 | hsa_1132 | D09693 | 0 | hsa_1909 | D05632 |
| 1 | hsa_1131 | D07513 | 0 | hsa_3355 | D06374 | 0 | hsa_3360 | D06567 |
| 1 | hsa_1132 | D07513 | 0 | hsa_2912 | D05879 | 0 | hsa_152 | D08569 |
| 1 | hsa_1133 | D07513 | 0 | hsa_2912 | D07838 | 0 | hsa_3356 | D04889 |
| 1 | hsa_3269 | D07522 | 0 | hsa_2559 | D08362 | 0 | hsa_4987 | D04492 |
| 1 | hsa_153 | D07526 | 0 | hsa_154 | D00101 | 0 | hsa_2557 | D02981 |
| 1 | hsa_154 | D07534 | 0 | hsa_2559 | D08305 | 0 | hsa_552 | D01263 |
| 1 | hsa_153 | D07537 | 0 | hsa_134 | D05004 | 0 | hsa_1815 | D01343 |
| 1 | hsa_1909 | D07538 | 0 | hsa_6344 | D02388 | 0 | hsa_1909 | D00482 |
| 1 | hsa_3269 | D07543 | 0 | hsa_5021 | D07809 | 0 | hsa_155 | D01548 |
| 1 | hsa_146 | D07550 | 0 | hsa_153 | D09650 | 0 | hsa_624 | D03704 |
| 1 | hsa_147 | D07550 | 0 | hsa_6870 | D05196 | 0 | hsa_2565 | D05471 |
| 1 | hsa_148 | D07550 | 0 | hsa_1132 | D00797 | 0 | hsa_3354 | D08837 |
| 1 | hsa_153 | D07551 | 0 | hsa_1394 | D07312 | 0 | hsa_2550 | D10441 |
| 1 | hsa_4988 | D07557 | 0 | hsa_1241 | D08693 | 0 | hsa_3352 | D05732 |
| 1 | hsa_146 | D07560 | 0 | hsa_6751 | D05462 | 0 | hsa_4988 | D08989 |
| 1 | hsa_147 | D07560 | 0 | hsa_2558 | D04724 | 0 | hsa_2913 | D05217 |
| 1 | hsa_148 | D07560 | 0 | hsa_1813 | D01674 | 0 | hsa_6755 | D10478 |
| 1 | hsa_1813 | D07563 | 0 | hsa_1394 | D00079 | 0 | hsa_1128 | D05832 |
| 1 | hsa_153 | D07590 | 0 | hsa_5143 | D05206 | 0 | hsa_5141 | D10171 |
| 1 | hsa_3350 | D07593 | 0 | hsa_10280 | D07522 | 0 | hsa_5141 | D05037 |
| 1 | hsa_134 | D07603 | 0 | hsa_135 | D03622 | 0 | hsa_7201 | D09954 |
| 1 | hsa_154 | D07608 | 0 | hsa_2568 | D07407 | 0 | hsa_135 | D02081 |
| 1 | hsa_3269 | D07617 | 0 | hsa_624 | D02163 | 0 | hsa_285242 | D03267 |
| 1 | hsa_4988 | D07620 | 0 | hsa_3061 | D00720 | 0 | hsa_886 | D05718 |
| 1 | hsa_153 | D07624 | 0 | hsa_1394 | D08115 | 0 | hsa_5139 | D00124 |
| 1 | hsa_153 | D07660 | 0 | hsa_6755 | D03212 | 0 | hsa_3062 | D09933 |
| 1 | hsa_3269 | D07662 | 0 | hsa_2564 | D05048 | 0 | hsa_147 | D02705 |
| 1 | hsa_2798 | D07665 | 0 | hsa_200909 | D02101 | 0 | hsa_4157 | D07803 |
| 1 | hsa_1128 | D07667 | 0 | hsa_6753 | D01004 | 0 | hsa_6870 | D02983 |
| 1 | hsa_1131 | D07667 | 0 | hsa_2559 | D03860 | 0 | hsa_552 | D00717 |
| 1 | hsa_1909 | D07682 | 0 | hsa_147 | D08067 | 0 | hsa_134 | D00373 |
| 1 | hsa_3269 | D07684 | 0 | hsa_5143 | D04184 | 0 | hsa_3352 | D02722 |
| 1 | hsa_154 | D07713 | 0 | hsa_5144 | D00320 | 0 | hsa_6754 | D07461 |
| 1 | hsa_1813 | D07718 | 0 | hsa_3357 | D07140 | 0 | hsa_151 | D08989 |
| 1 | hsa_64805 | D07729 | 0 | hsa_886 | D09017 | 0 | hsa_5142 | D08239 |
| 1 | hsa_3269 | D07734 | 0 | hsa_6755 | D09765 | 0 | hsa_3356 | D02901 |
| 1 | hsa_4988 | D07740 | 0 | hsa_64805 | D04694 | 0 | hsa_6752 | D08297 |
| 1 | hsa_552 | D07748 | 0 | hsa_624 | D02705 | 0 | hsa_799 | D01600 |
| 1 | hsa_1128 | D07759 | 0 | hsa_2564 | D08160 | 0 | hsa_5142 | D00678 |
| 1 | hsa_1129 | D07759 | 0 | hsa_11255 | D08236 | 0 | hsa_3274 | D02016 |
| 1 | hsa_1131 | D07759 | 0 | hsa_2798 | D08105 | 0 | hsa_10800 | D02205 |
| 1 | hsa_1132 | D07759 | 0 | hsa_6751 | D01263 | 0 | hsa_1901 | D03506 |
| 1 | hsa_1133 | D07759 | 0 | hsa_10203 | D10648 | 0 | hsa_1131 | D07377 |
| 1 | hsa_3269 | D07765 | 0 | hsa_2904 | D02245 | 0 | hsa_1128 | D01858 |
| 1 | hsa_150 | D07795 | 0 | hsa_6570 | D06552 | 0 | hsa_6344 | D02609 |
| 1 | hsa_151 | D07795 | 0 | hsa_5143 | D10660 | 0 | hsa_6532 | D06157 |
| 1 | hsa_152 | D07795 | 0 | hsa_7201 | D05649 | 0 | hsa_1812 | D01674 |
| 1 | hsa_3269 | D07803 | 0 | hsa_886 | D09008 | 0 | hsa_2864 | D08046 |
| 1 | hsa_1128 | D07804 | 0 | hsa_5143 | D08411 | 0 | hsa_2556 | D01176 |
| 1 | hsa_1129 | D07804 | 0 | hsa_1132 | D02396 | 0 | hsa_2862 | D01360 |
| 1 | hsa_1131 | D07804 | 0 | hsa_151 | D02950 | 0 | hsa_285242 | D08836 |
| 1 | hsa_1132 | D07804 | 0 | hsa_1394 | D08248 | 0 | hsa_1133 | D04492 |
| 1 | hsa_1133 | D07804 | 0 | hsa_2693 | D00661 | 0 | hsa_2554 | D08645 |
| 1 | hsa_4988 | D07809 | 0 | hsa_1814 | D09400 | 0 | hsa_155 | D08511 |
| 1 | hsa_4988 | D07810 | 0 | hsa_4889 | D02846 | 0 | hsa_3061 | D01096 |
| 1 | hsa_1128 | D07820 | 0 | hsa_2912 | D10441 | 0 | hsa_3352 | D02739 |
| 1 | hsa_1128 | D07821 | 0 | hsa_624 | D05471 | 0 | hsa_3062 | D02200 |
| 1 | hsa_1129 | D07821 | 0 | hsa_3357 | D07809 | 0 | hsa_3354 | D06328 |
| 1 | hsa_1131 | D07821 | 0 | hsa_3354 | D01521 | 0 | hsa_64805 | D07129 |
| 1 | hsa_1132 | D07821 | 0 | hsa_2555 | D00719 | 0 | hsa_6571 | D04184 |
| 1 | hsa_1133 | D07821 | 0 | hsa_1129 | D01355 | 0 | hsa_5732 | D04710 |
| 1 | hsa_4988 | D07831 | 0 | hsa_4889 | D04116 | 0 | hsa_6755 | D10493 |
| 1 | hsa_4988 | D07832 | 0 | hsa_147 | D08624 | 0 | hsa_9177 | D02037 |
| 1 | hsa_3350 | D07837 | 0 | hsa_9177 | D07821 | 0 | hsa_1241 | D06566 |
| 1 | hsa_3350 | D07838 | 0 | hsa_6571 | D02248 | 0 | hsa_2562 | D07451 |
| 1 | hsa_3269 | D07853 | 0 | hsa_2563 | D05478 | 0 | hsa_5737 | D08966 |
| 1 | hsa_3356 | D07854 | 0 | hsa_1394 | D02750 | 0 | hsa_135 | D08937 |
| 1 | hsa_3357 | D07854 | 0 | hsa_3359 | D10355 | 0 | hsa_285242 | D01448 |
| 1 | hsa_3358 | D07854 | 0 | hsa_1268 | D00778 | 0 | hsa_1394 | D08578 |
| 1 | hsa_3269 | D07860 | 0 | hsa_3061 | D07181 | 0 | hsa_2798 | D08322 |
| 1 | hsa_4988 | D07861 | 0 | hsa_134 | D06280 | 0 | hsa_2557 | D08988 |
| 1 | hsa_3269 | D07862 | 0 | hsa_1394 | D07148 | 0 | hsa_4987 | D07491 |
| 1 | hsa_3359 | D07867 | 0 | hsa_1128 | D05476 | 0 | hsa_5140 | D02363 |
| 1 | hsa_9177 | D07867 | 0 | hsa_6753 | D08661 | 0 | hsa_6752 | D08578 |
| 1 | hsa_170572 | D07867 | 0 | hsa_6608 | D00782 | 0 | hsa_1909 | D01830 |
| 1 | hsa_200909 | D07867 | 0 | hsa_6570 | D07665 | 0 | hsa_846 | D01385 |
| 1 | hsa_285242 | D07867 | 0 | hsa_2913 | D07483 | 0 | hsa_3357 | D10703 |
| 1 | hsa_1813 | D07868 | 0 | hsa_6915 | D07748 | 0 | hsa_6751 | D09008 |
| 1 | hsa_1812 | D07870 | 0 | hsa_1131 | D08343 | 0 | hsa_6570 | D00664 |
| 1 | hsa_146 | D07874 | 0 | hsa_285242 | D08297 | 0 | hsa_152 | D00076 |
| 1 | hsa_147 | D07874 | 0 | hsa_2915 | D01077 | 0 | hsa_1133 | D07182 |
| 1 | hsa_148 | D07874 | 0 | hsa_3269 | D05606 | 0 | hsa_1241 | D08861 |
| 1 | hsa_3269 | D07878 | 0 | hsa_5731 | D02192 | 0 | hsa_56413 | D02666 |
| 1 | hsa_3351 | D07887 | 0 | hsa_154 | D03002 | 0 | hsa_1132 | D02836 |
| 1 | hsa_3269 | D07890 | 0 | hsa_2915 | D08247 | 0 | hsa_2913 | D01077 |
| 1 | hsa_3269 | D07900 | 0 | hsa_147 | D00702 | 0 | hsa_1129 | D08923 |
| 1 | hsa_4986 | D07904 | 0 | hsa_150 | D10362 | 0 | hsa_2556 | D05523 |
| 1 | hsa_3350 | D07905 | 0 | hsa_3350 | D01028 | 0 | hsa_846 | D07459 |
| 1 | hsa_3351 | D07905 | 0 | hsa_4985 | D08525 | 0 | hsa_2562 | D01105 |
| 1 | hsa_3352 | D07905 | 0 | hsa_9568 | D02605 | 0 | hsa_147 | D01794 |
| 1 | hsa_3354 | D07905 | 0 | hsa_2568 | D05718 | 0 | hsa_3358 | D00835 |
| 1 | hsa_3355 | D07905 | 0 | hsa_152 | D01573 | 0 | hsa_6754 | D06007 |
| 1 | hsa_3350 | D07906 | 0 | hsa_2149 | D01801 | 0 | hsa_2912 | D08124 |
| 1 | hsa_153 | D07916 | 0 | hsa_3356 | D04823 | 0 | hsa_846 | D07870 |
| 1 | hsa_4988 | D07929 | 0 | hsa_2561 | D00776 | 0 | hsa_4986 | D01887 |
| 1 | hsa_146 | D07931 | 0 | hsa_2565 | D01347 | 0 | hsa_624 | D00610 |
| 1 | hsa_147 | D07931 | 0 | hsa_6571 | D04794 | 0 | hsa_886 | D02577 |
| 1 | hsa_148 | D07931 | 0 | hsa_185 | D02396 | 0 | hsa_146 | D03953 |
| 1 | hsa_4988 | D07937 | 0 | hsa_10800 | D08234 | 0 | hsa_1268 | D00999 |
| 1 | hsa_1812 | D07946 | 0 | hsa_285242 | D09698 | 0 | hsa_2862 | D02968 |
| 1 | hsa_1128 | D07951 | 0 | hsa_154 | D02212 | 0 | hsa_1129 | D09732 |
| 1 | hsa_1129 | D07951 | 0 | hsa_55879 | D03891 | 0 | hsa_3358 | D02950 |
| 1 | hsa_1131 | D07951 | 0 | hsa_1131 | D01307 | 0 | hsa_1132 | D01477 |
| 1 | hsa_1132 | D07951 | 0 | hsa_59340 | D08057 | 0 | hsa_1132 | D01847 |
| 1 | hsa_1133 | D07951 | 0 | hsa_6755 | D00835 | 0 | hsa_55879 | D00540 |
| 1 | hsa_3269 | D07958 | 0 | hsa_552 | D09717 | 0 | hsa_3362 | D08184 |
| 1 | hsa_1813 | D07976 | 0 | hsa_3269 | D07803 | 0 | hsa_3355 | D00511 |
| 1 | hsa_1813 | D07977 | 0 | hsa_10800 | D08940 | 0 | hsa_3269 | D00776 |
| 1 | hsa_154 | D07990 | 0 | hsa_155 | D06374 | 0 | hsa_6751 | D08249 |
| 1 | hsa_3351 | D07997 | 0 | hsa_6608 | D08341 | 0 | hsa_185 | D02632 |
| 1 | hsa_2798 | D08010 | 0 | hsa_3359 | D04973 | 0 | hsa_6869 | D08861 |
| 1 | hsa_4889 | D08026 | 0 | hsa_5141 | D02683 | 0 | hsa_5144 | D07734 |
| 1 | hsa_2798 | D08027 | 0 | hsa_3351 | D04765 | 0 | hsa_2563 | D04626 |
| 1 | hsa_150 | D08031 | 0 | hsa_1815 | D06659 | 0 | hsa_5144 | D09693 |
| 1 | hsa_151 | D08031 | 0 | hsa_5139 | D08234 | 0 | hsa_154 | D01452 |
| 1 | hsa_152 | D08031 | 0 | hsa_846 | D04724 | 0 | hsa_1128 | D08115 |
| 1 | hsa_1813 | D08035 | 0 | hsa_3269 | D02101 | 0 | hsa_1241 | D02104 |
| 1 | hsa_154 | D08039 | 0 | hsa_2558 | D06021 | 0 | hsa_3362 | D08639 |
| 1 | hsa_3269 | D08040 | 0 | hsa_2559 | D00599 | 0 | hsa_2563 | D09645 |
| 1 | hsa_3269 | D08041 | 0 | hsa_2562 | D08611 | 0 | hsa_1128 | D05996 |
| 1 | hsa_4988 | D08045 | 0 | hsa_4889 | D08010 | 0 | hsa_5143 | D09982 |
| 1 | hsa_4988 | D08046 | 0 | hsa_5729 | D10184 | 0 | hsa_552 | D08047 |
| 1 | hsa_4988 | D08047 | 0 | hsa_2912 | D00076 | 0 | hsa_2554 | D06633 |
| 1 | hsa_3269 | D08054 | 0 | hsa_148 | D09845 | 0 | hsa_146 | D04074 |
| 1 | hsa_1812 | D08057 | 0 | hsa_5732 | D04361 | 0 | hsa_148 | D01317 |
| 1 | hsa_2904 | D08064 | 0 | hsa_3269 | D02693 | 0 | hsa_4987 | D07229 |
| 1 | hsa_5739 | D08065 | 0 | hsa_6753 | D05907 | 0 | hsa_2149 | D01624 |
| 1 | hsa_185 | D08067 | 0 | hsa_3351 | D07560 | 0 | hsa_2149 | D07148 |
| 1 | hsa_153 | D08074 | 0 | hsa_10280 | D10006 | 0 | hsa_6755 | D03587 |
| 1 | hsa_153 | D08078 | 0 | hsa_1241 | D03495 | 0 | hsa_3354 | D10729 |
| 1 | hsa_153 | D08090 | 0 | hsa_3356 | D09988 | 0 | hsa_1129 | D07563 |
| 1 | hsa_3269 | D08091 | 0 | hsa_135 | D01006 | 0 | hsa_5731 | D05941 |
| 1 | hsa_154 | D08092 | 0 | hsa_2693 | D03982 | 0 | hsa_2564 | D01148 |
| 1 | hsa_154 | D08093 | 0 | hsa_2562 | D07684 | 0 | hsa_55879 | D08099 |
| 1 | hsa_3356 | D08099 | 0 | hsa_799 | D03622 | 0 | hsa_1133 | D02739 |
| 1 | hsa_3269 | D08105 | 0 | hsa_147 | D00997 | 0 | hsa_5142 | D05277 |
| 1 | hsa_146 | D08106 | 0 | hsa_43 | D04823 | 0 | hsa_4988 | D02846 |
| 1 | hsa_147 | D08106 | 0 | hsa_2864 | D01337 | 0 | hsa_9177 | D05230 |
| 1 | hsa_148 | D08106 | 0 | hsa_6869 | D10703 | 0 | hsa_6870 | D10612 |
| 1 | hsa_2798 | D08113 | 0 | hsa_5724 | D05587 | 0 | hsa_1268 | D10119 |
| 1 | hsa_153 | D08115 | 0 | hsa_2568 | D01172 | 0 | hsa_59340 | D01460 |
| 1 | hsa_3269 | D08117 | 0 | hsa_3362 | D05660 | 0 | hsa_2566 | D01172 |
| 1 | hsa_3269 | D08118 | 0 | hsa_6608 | D06329 | 0 | hsa_5139 | D02878 |
| 1 | hsa_4988 | D08121 | 0 | hsa_5142 | D04184 | 0 | hsa_2554 | D08382 |
| 1 | hsa_4988 | D08122 | 0 | hsa_9177 | D02192 | 0 | hsa_170572 | D04710 |
| 1 | hsa_4988 | D08123 | 0 | hsa_2913 | D02612 | 0 | hsa_6608 | D04752 |
| 1 | hsa_154 | D08124 | 0 | hsa_3357 | D08443 | 0 | hsa_2864 | D07102 |
| 1 | hsa_3350 | D08132 | 0 | hsa_2864 | D07667 | 0 | hsa_2555 | D02071 |
| 1 | hsa_151 | D08141 | 0 | hsa_2862 | D07218 | 0 | hsa_2550 | D10563 |
| 1 | hsa_4988 | D08144 | 0 | hsa_11255 | D08144 | 0 | hsa_6755 | D08165 |
| 1 | hsa_185 | D08146 | 0 | hsa_6915 | D02235 | 0 | hsa_5139 | D02227 |
| 1 | hsa_1813 | D08148 | 0 | hsa_4987 | D02093 | 0 | hsa_2904 | D02693 |
| 1 | hsa_1128 | D08157 | 0 | hsa_5724 | D10188 | 0 | hsa_3359 | D08989 |
| 1 | hsa_1129 | D08157 | 0 | hsa_3358 | D08118 | 0 | hsa_1268 | D05738 |
| 1 | hsa_1131 | D08157 | 0 | hsa_2555 | D07997 | 0 | hsa_146 | D08408 |
| 1 | hsa_1132 | D08157 | 0 | hsa_2554 | D02101 | 0 | hsa_154 | D03587 |
| 1 | hsa_1133 | D08157 | 0 | hsa_799 | D00058 | 0 | hsa_5724 | D05340 |
| 1 | hsa_1128 | D08160 | 0 | hsa_5144 | D08201 | 0 | hsa_6870 | D01333 |
| 1 | hsa_1129 | D08160 | 0 | hsa_2904 | D03504 | 0 | hsa_5732 | D05340 |
| 1 | hsa_1131 | D08160 | 0 | hsa_799 | D02108 | 0 | hsa_6753 | D01955 |
| 1 | hsa_1132 | D08160 | 0 | hsa_148 | D10324 | 0 | hsa_2558 | D00147 |
| 1 | hsa_1133 | D08160 | 0 | hsa_2560 | D07550 | 0 | hsa_1901 | D10478 |
| 1 | hsa_3269 | D08163 | 0 | hsa_2915 | D06374 | 0 | hsa_2798 | D08235 |
| 1 | hsa_150 | D08165 | 0 | hsa_2798 | D01815 | 0 | hsa_1815 | D10309 |
| 1 | hsa_151 | D08165 | 0 | hsa_3269 | D02281 | 0 | hsa_5142 | D08195 |
| 1 | hsa_152 | D08165 | 0 | hsa_2904 | D02849 | 0 | hsa_2862 | D06405 |
| 1 | hsa_4543 | D08170 | 0 | hsa_1241 | D08549 | 0 | hsa_6753 | D07713 |
| 1 | hsa_4988 | D08182 | 0 | hsa_554 | D01861 | 0 | hsa_1901 | D09570 |
| 1 | hsa_3269 | D08183 | 0 | hsa_799 | D07820 | 0 | hsa_6870 | D00124 |
| 1 | hsa_3269 | D08184 | 0 | hsa_134 | D08325 | 0 | hsa_155 | D07874 |
| 1 | hsa_146 | D08192 | 0 | hsa_64805 | D06246 | 0 | hsa_4986 | D03361 |
| 1 | hsa_147 | D08192 | 0 | hsa_886 | D07795 | 0 | hsa_2149 | D08389 |
| 1 | hsa_148 | D08192 | 0 | hsa_2568 | D00796 | 0 | hsa_5142 | D08489 |
| 1 | hsa_4988 | D08195 | 0 | hsa_3352 | D07837 | 0 | hsa_5731 | D01451 |
| 1 | hsa_146 | D08201 | 0 | hsa_1241 | D03504 | 0 | hsa_2915 | D07312 |
| 1 | hsa_147 | D08201 | 0 | hsa_3356 | D09949 | 0 | hsa_6532 | D10703 |
| 1 | hsa_148 | D08201 | 0 | hsa_6751 | D08342 | 0 | hsa_134 | D00663 |
| 1 | hsa_150 | D08205 | 0 | hsa_3354 | D10364 | 0 | hsa_3350 | D01654 |
| 1 | hsa_151 | D08205 | 0 | hsa_6571 | D06330 | 0 | hsa_2913 | D10662 |
| 1 | hsa_152 | D08205 | 0 | hsa_5144 | D01600 | 0 | hsa_4157 | D03008 |
| 1 | hsa_146 | D08206 | 0 | hsa_4987 | D10628 | 0 | hsa_3757 | D02622 |
| 1 | hsa_147 | D08206 | 0 | hsa_3351 | D02130 | 0 | hsa_59340 | D02040 |
| 1 | hsa_148 | D08206 | 0 | hsa_1133 | D02666 | 0 | hsa_64805 | D09732 |
| 1 | hsa_3350 | D08207 | 0 | hsa_1268 | D02371 | 0 | hsa_2565 | D07557 |
| 1 | hsa_3351 | D08207 | 0 | hsa_6570 | D08635 | 0 | hsa_2568 | D07228 |
| 1 | hsa_3352 | D08207 | 0 | hsa_3062 | D01469 | 0 | hsa_6571 | D05738 |
| 1 | hsa_3354 | D08207 | 0 | hsa_2561 | D08639 | 0 | hsa_3274 | D02670 |
| 1 | hsa_3355 | D08207 | 0 | hsa_6571 | D04868 | 0 | hsa_134 | D07184 |
| 1 | hsa_1128 | D08209 | 0 | hsa_1129 | D01007 | 0 | hsa_4985 | D10660 |
| 1 | hsa_1129 | D08209 | 0 | hsa_2564 | D08074 | 0 | hsa_2558 | D06566 |
| 1 | hsa_1131 | D08209 | 0 | hsa_3757 | D07976 | 0 | hsa_6751 | D08341 |
| 1 | hsa_1132 | D08209 | 0 | hsa_552 | D07460 | 0 | hsa_2864 | D07175 |
| 1 | hsa_1133 | D08209 | 0 | hsa_1815 | D01006 | 0 | hsa_3355 | D02384 |
| 1 | hsa_146 | D08212 | 0 | hsa_2561 | D03198 | 0 | hsa_2567 | D08343 |
| 1 | hsa_147 | D08212 | 0 | hsa_185 | D07077 | 0 | hsa_2149 | D01469 |
| 1 | hsa_148 | D08212 | 0 | hsa_2557 | D00366 | 0 | hsa_624 | D01627 |
| 1 | hsa_3350 | D08216 | 0 | hsa_147 | D06613 | 0 | hsa_3350 | D06317 |
| 1 | hsa_3351 | D08216 | 0 | hsa_9177 | D03197 | 0 | hsa_2798 | D08508 |
| 1 | hsa_3352 | D08216 | 0 | hsa_799 | D09003 | 0 | hsa_151 | D03860 |
| 1 | hsa_3354 | D08216 | 0 | hsa_80834 | D00797 | 0 | hsa_1131 | D05196 |
| 1 | hsa_3355 | D08216 | 0 | hsa_2864 | D09794 | 0 | hsa_6570 | D06293 |
| 1 | hsa_146 | D08220 | 0 | hsa_2798 | D06019 | 0 | hsa_2563 | D01463 |
| 1 | hsa_147 | D08220 | 0 | hsa_153 | D01008 | 0 | hsa_59340 | D01405 |
| 1 | hsa_148 | D08220 | 0 | hsa_6570 | D00633 | 0 | hsa_4986 | D07099 |
| 1 | hsa_1813 | D08226 | 0 | hsa_6915 | D07810 | 0 | hsa_1268 | D02684 |
| 1 | hsa_10800 | D08229 | 0 | hsa_5734 | D07493 | 0 | hsa_9177 | D09732 |
| 1 | hsa_4988 | D08233 | 0 | hsa_4987 | D05711 | 0 | hsa_2559 | D00661 |
| 1 | hsa_4988 | D08234 | 0 | hsa_2550 | D00663 | 0 | hsa_6752 | D04752 |
| 1 | hsa_3356 | D08235 | 0 | hsa_9568 | D07377 | 0 | hsa_2555 | D04126 |
| 1 | hsa_3360 | D08236 | 0 | hsa_2565 | D09319 | 0 | hsa_3350 | D08040 |
| 1 | hsa_146 | D08239 | 0 | hsa_3358 | D02676 | 0 | hsa_4985 | D10188 |
| 1 | hsa_147 | D08239 | 0 | hsa_1132 | D08397 | 0 | hsa_153 | D03506 |
| 1 | hsa_148 | D08239 | 0 | hsa_3351 | D00559 | 0 | hsa_10280 | D03937 |
| 1 | hsa_2798 | D08241 | 0 | hsa_6752 | D02968 | 0 | hsa_5021 | D07229 |
| 1 | hsa_3356 | D08244 | 0 | hsa_150 | D08497 | 0 | hsa_80834 | D04703 |
| 1 | hsa_4986 | D08246 | 0 | hsa_1909 | D08571 | 0 | hsa_2864 | D00147 |
| 1 | hsa_4988 | D08247 | 0 | hsa_2564 | D00784 | 0 | hsa_3269 | D08411 |
| 1 | hsa_4988 | D08248 | 0 | hsa_151 | D00782 | 0 | hsa_285242 | D05818 |
| 1 | hsa_4988 | D08249 | 0 | hsa_151 | D07312 | 0 | hsa_3357 | D02071 |
| 1 | hsa_146 | D08253 | 0 | hsa_11255 | D07603 | 0 | hsa_6915 | D05730 |
| 1 | hsa_147 | D08253 | 0 | hsa_5140 | D08183 | 0 | hsa_7201 | D01085 |
| 1 | hsa_148 | D08253 | 0 | hsa_3354 | D10631 | 0 | hsa_1132 | D07667 |
| 1 | hsa_3350 | D08255 | 0 | hsa_152 | D10677 | 0 | hsa_2550 | D01538 |
| 1 | hsa_3356 | D08257 | 0 | hsa_3350 | D01520 | 0 | hsa_5732 | D02848 |
| 1 | hsa_146 | D08271 | 0 | hsa_3358 | D08244 | 0 | hsa_2565 | D00511 |
| 1 | hsa_147 | D08271 | 0 | hsa_5734 | D04680 | 0 | hsa_9177 | D09876 |
| 1 | hsa_148 | D08271 | 0 | hsa_5731 | D00835 | 0 | hsa_152 | D00799 |
| 1 | hsa_146 | D08284 | 0 | hsa_3360 | D08121 | 0 | hsa_9177 | D09699 |
| 1 | hsa_147 | D08284 | 0 | hsa_4889 | D03087 | 0 | hsa_3350 | D07617 |
| 1 | hsa_148 | D08284 | 0 | hsa_3359 | D10391 | 0 | hsa_4987 | D10170 |
| 1 | hsa_146 | D08286 | 0 | hsa_3061 | D02211 | 0 | hsa_3362 | D01771 |
| 1 | hsa_147 | D08286 | 0 | hsa_1813 | D02369 | 0 | hsa_6754 | D05206 |
| 1 | hsa_148 | D08286 | 0 | hsa_6532 | D00559 | 0 | hsa_1394 | D07682 |
| 1 | hsa_3269 | D08293 | 0 | hsa_5734 | D02016 | 0 | hsa_552 | D05587 |
| 1 | hsa_1813 | D08297 | 0 | hsa_2550 | D08684 | 0 | hsa_2563 | D01462 |
| 1 | hsa_154 | D08300 | 0 | hsa_2554 | D01698 | 0 | hsa_5737 | D06328 |
| 1 | hsa_1128 | D08305 | 0 | hsa_3350 | D01818 | 0 | hsa_2565 | D10391 |
| 1 | hsa_1129 | D08305 | 0 | hsa_10280 | D08300 | 0 | hsa_6751 | D03937 |
| 1 | hsa_1131 | D08305 | 0 | hsa_2566 | D08123 | 0 | hsa_152 | D04625 |
| 1 | hsa_1132 | D08305 | 0 | hsa_9177 | D02081 | 0 | hsa_2566 | D09651 |
| 1 | hsa_1133 | D08305 | 0 | hsa_43 | D03983 | 0 | hsa_3062 | D02034 |
| 1 | hsa_146 | D08308 | 0 | hsa_3359 | D07124 | 0 | hsa_1128 | D01386 |
| 1 | hsa_147 | D08308 | 0 | hsa_846 | D09997 | 0 | hsa_1814 | D03618 |
| 1 | hsa_148 | D08308 | 0 | hsa_154 | D07148 | 0 | hsa_5021 | D08648 |
| 1 | hsa_146 | D08309 | 0 | hsa_10203 | D08684 | 0 | hsa_6532 | D02760 |
| 1 | hsa_147 | D08309 | 0 | hsa_150 | D02090 | 0 | hsa_2798 | D05035 |
| 1 | hsa_148 | D08309 | 0 | hsa_1133 | D04703 | 0 | hsa_7201 | D02396 |
| 1 | hsa_153 | D08318 | 0 | hsa_2862 | D03535 | 0 | hsa_5144 | D01771 |
| 1 | hsa_146 | D08322 | 0 | hsa_1394 | D07406 | 0 | hsa_4987 | D02014 |
| 1 | hsa_147 | D08322 | 0 | hsa_1133 | D02738 | 0 | hsa_151 | D02860 |
| 1 | hsa_148 | D08322 | 0 | hsa_2912 | D08241 | 0 | hsa_135 | D08645 |
| 1 | hsa_4988 | D08323 | 0 | hsa_3352 | D07148 | 0 | hsa_154 | D02417 |
| 1 | hsa_1131 | D08325 | 0 | hsa_2563 | D00820 | 0 | hsa_1128 | D08635 |
| 1 | hsa_1812 | D08339 | 0 | hsa_200909 | D01500 | 0 | hsa_155 | D05010 |
| 1 | hsa_1813 | D08341 | 0 | hsa_3351 | D10433 | 0 | hsa_3356 | D07156 |
| 1 | hsa_1813 | D08342 | 0 | hsa_3757 | D08628 | 0 | hsa_5737 | D01600 |
| 1 | hsa_4988 | D08343 | 0 | hsa_6753 | D08010 | 0 | hsa_10203 | D01101 |
| 1 | hsa_3269 | D08353 | 0 | hsa_153 | D01003 | 0 | hsa_3357 | D08246 |
| 1 | hsa_3269 | D08355 | 0 | hsa_2904 | D01551 | 0 | hsa_5734 | D01468 |
| 1 | hsa_146 | D08358 | 0 | hsa_4157 | D08614 | 0 | hsa_1812 | D02849 |
| 1 | hsa_147 | D08358 | 0 | hsa_185 | D08969 | 0 | hsa_6753 | D08026 |
| 1 | hsa_148 | D08358 | 0 | hsa_170572 | D04765 | 0 | hsa_5141 | D10129 |
| 1 | hsa_146 | D08362 | 0 | hsa_5729 | D06063 | 0 | hsa_4157 | D10563 |
| 1 | hsa_147 | D08362 | 0 | hsa_5021 | D03264 | 0 | hsa_552 | D09752 |
| 1 | hsa_148 | D08362 | 0 | hsa_185 | D00366 | 0 | hsa_5139 | D02022 |
| 1 | hsa_146 | D08365 | 0 | hsa_3357 | D03982 | 0 | hsa_2913 | D02666 |
| 1 | hsa_147 | D08365 | 0 | hsa_846 | D06659 | 0 | hsa_6344 | D04098 |
| 1 | hsa_148 | D08365 | 0 | hsa_150 | D01861 | 0 | hsa_5734 | D04187 |
| 1 | hsa_146 | D08366 | 0 | hsa_3357 | D08093 | 0 | hsa_2559 | D02850 |
| 1 | hsa_147 | D08366 | 0 | hsa_2562 | D00847 | 0 | hsa_4987 | D08343 |
| 1 | hsa_148 | D08366 | 0 | hsa_10203 | D04517 | 0 | hsa_3269 | D00561 |
| 1 | hsa_1128 | D08376 | 0 | hsa_3359 | D03622 | 0 | hsa_6754 | D04494 |
| 1 | hsa_1129 | D08376 | 0 | hsa_5724 | D03495 | 0 | hsa_3351 | D08495 |
| 1 | hsa_1131 | D08376 | 0 | hsa_3061 | D03622 | 0 | hsa_3354 | D06103 |
| 1 | hsa_1132 | D08376 | 0 | hsa_5139 | D05587 | 0 | hsa_1129 | D10362 |
| 1 | hsa_1133 | D08376 | 0 | hsa_155 | D08182 | 0 | hsa_6752 | D05395 |
| 1 | hsa_1128 | D08382 | 0 | hsa_3352 | D00681 | 0 | hsa_2558 | D04038 |
| 1 | hsa_1129 | D08382 | 0 | hsa_3357 | D03504 | 0 | hsa_146 | D07878 |
| 1 | hsa_1131 | D08382 | 0 | hsa_1814 | D01573 | 0 | hsa_3354 | D01021 |
| 1 | hsa_1132 | D08382 | 0 | hsa_7201 | D00787 | 0 | hsa_2560 | D01946 |
| 1 | hsa_1133 | D08382 | 0 | hsa_2559 | D00573 | 0 | hsa_6751 | D08318 |
| 1 | hsa_1128 | D08384 | 0 | hsa_3360 | D01992 | 0 | hsa_6570 | D10338 |
| 1 | hsa_1129 | D08384 | 0 | hsa_2563 | D05575 | 0 | hsa_10203 | D02095 |
| 1 | hsa_1131 | D08384 | 0 | hsa_170572 | D02034 | 0 | hsa_6532 | D02739 |
| 1 | hsa_1132 | D08384 | 0 | hsa_6751 | D07803 | 0 | hsa_3274 | D08163 |
| 1 | hsa_1133 | D08384 | 0 | hsa_6753 | D08293 | 0 | hsa_11255 | D07946 |
| 1 | hsa_154 | D08387 | 0 | hsa_1268 | D04868 | 0 | hsa_1132 | D10119 |
| 1 | hsa_1128 | D08389 | 0 | hsa_6755 | D02599 | 0 | hsa_154 | D09791 |
| 1 | hsa_1128 | D08395 | 0 | hsa_3061 | D08358 | 0 | hsa_3274 | D04501 |
| 1 | hsa_1129 | D08395 | 0 | hsa_151 | D08384 | 0 | hsa_154 | D02111 |
| 1 | hsa_1131 | D08395 | 0 | hsa_5729 | D02357 | 0 | hsa_5724 | D07171 |
| 1 | hsa_1132 | D08395 | 0 | hsa_5021 | D01003 | 0 | hsa_55879 | D02972 |
| 1 | hsa_1133 | D08395 | 0 | hsa_1901 | D01201 | 0 | hsa_1241 | D04970 |
| 1 | hsa_3356 | D08397 | 0 | hsa_147 | D08184 | 0 | hsa_2566 | D00559 |
| 1 | hsa_10800 | D08408 | 0 | hsa_5139 | D00990 | 0 | hsa_846 | D08040 |
| 1 | hsa_146 | D08411 | 0 | hsa_2557 | D08578 | 0 | hsa_150 | D05660 |
| 1 | hsa_147 | D08411 | 0 | hsa_6870 | D07460 | 0 | hsa_6754 | D04531 |
| 1 | hsa_148 | D08411 | 0 | hsa_152 | D08339 | 0 | hsa_5724 | D10403 |
| 1 | hsa_154 | D08424 | 0 | hsa_9177 | D04531 | 0 | hsa_5143 | D02683 |
| 1 | hsa_1128 | D08425 | 0 | hsa_6915 | D05471 | 0 | hsa_5140 | D03221 |
| 1 | hsa_1129 | D08425 | 0 | hsa_2567 | D00999 | 0 | hsa_1129 | D06274 |
| 1 | hsa_1131 | D08425 | 0 | hsa_3360 | D03060 | 0 | hsa_2550 | D06007 |
| 1 | hsa_1132 | D08425 | 0 | hsa_4889 | D02981 | 0 | hsa_6751 | D03087 |
| 1 | hsa_1133 | D08425 | 0 | hsa_2556 | D10336 | 0 | hsa_3757 | D10403 |
| 1 | hsa_1128 | D08426 | 0 | hsa_2912 | D09003 | 0 | hsa_80834 | D00573 |
| 1 | hsa_1129 | D08426 | 0 | hsa_2568 | D10170 | 0 | hsa_135 | D01347 |
| 1 | hsa_1131 | D08426 | 0 | hsa_4987 | D10677 | 0 | hsa_2559 | D08511 |
| 1 | hsa_1132 | D08426 | 0 | hsa_10280 | D02069 | 0 | hsa_846 | D08988 |
| 1 | hsa_1133 | D08426 | 0 | hsa_6532 | D03198 | 0 | hsa_6608 | D08487 |
| 1 | hsa_3269 | D08432 | 0 | hsa_1241 | D03087 | 0 | hsa_2566 | D07522 |
| 1 | hsa_1128 | D08441 | 0 | hsa_6869 | D10516 | 0 | hsa_200909 | D08229 |
| 1 | hsa_1129 | D08441 | 0 | hsa_2864 | D10174 | 0 | hsa_1813 | D01163 |
| 1 | hsa_1131 | D08441 | 0 | hsa_80834 | D01201 | 0 | hsa_846 | D05902 |
| 1 | hsa_1132 | D08441 | 0 | hsa_10280 | D01447 | 0 | hsa_3357 | D08389 |
| 1 | hsa_1133 | D08441 | 0 | hsa_9568 | D01349 | 0 | hsa_1814 | D07831 |
| 1 | hsa_153 | D08443 | 0 | hsa_4985 | D01685 | 0 | hsa_2561 | D03535 |
| 1 | hsa_146 | D08449 | 0 | hsa_3062 | D01685 | 0 | hsa_2564 | D08424 |
| 1 | hsa_147 | D08449 | 0 | hsa_5021 | D08494 | 0 | hsa_4889 | D03405 |
| 1 | hsa_148 | D08449 | 0 | hsa_2555 | D07713 | 0 | hsa_7201 | D00840 |
| 1 | hsa_3356 | D08456 | 0 | hsa_1268 | D07407 | 0 | hsa_2693 | D08220 |
| 1 | hsa_1813 | D08457 | 0 | hsa_2912 | D00840 | 0 | hsa_1241 | D01383 |
| 1 | hsa_3359 | D08466 | 0 | hsa_2798 | D07620 | 0 | hsa_135 | D01340 |
| 1 | hsa_9177 | D08466 | 0 | hsa_2864 | D07590 | 0 | hsa_5734 | D05395 |
| 1 | hsa_170572 | D08466 | 0 | hsa_1901 | D04500 | 0 | hsa_155 | D05035 |
| 1 | hsa_200909 | D08466 | 0 | hsa_3360 | D08389 | 0 | hsa_2563 | D10128 |
| 1 | hsa_285242 | D08466 | 0 | hsa_3355 | D00600 | 0 | hsa_5140 | D08206 |
| 1 | hsa_4988 | D08473 | 0 | hsa_6755 | D01123 | 0 | hsa_1901 | D00366 |
| 1 | hsa_154 | D08474 | 0 | hsa_43 | D08517 | 0 | hsa_5139 | D08662 |
| 1 | hsa_150 | D08482 | 0 | hsa_3351 | D05902 | 0 | hsa_3062 | D07617 |
| 1 | hsa_151 | D08482 | 0 | hsa_5141 | D07740 | 0 | hsa_3269 | D00723 |
| 1 | hsa_152 | D08482 | 0 | hsa_2554 | D01958 | 0 | hsa_5139 | D06140 |
| 1 | hsa_3351 | D08485 | 0 | hsa_4988 | D09349 | 0 | hsa_3351 | D08626 |
| 1 | hsa_150 | D08487 | 0 | hsa_152 | D10135 | 0 | hsa_1901 | D08600 |
| 1 | hsa_151 | D08487 | 0 | hsa_2864 | D02632 | 0 | hsa_6344 | D07101 |
| 1 | hsa_152 | D08487 | 0 | hsa_2149 | D06000 | 0 | hsa_154 | D01231 |
| 1 | hsa_1813 | D08489 | 0 | hsa_3269 | D09976 | 0 | hsa_2567 | D04502 |
| 1 | hsa_3274 | D08494 | 0 | hsa_1131 | D08525 | 0 | hsa_2566 | D04788 |
| 1 | hsa_3274 | D08495 | 0 | hsa_146 | D06495 | 0 | hsa_886 | D05366 |
| 1 | hsa_3269 | D08497 | 0 | hsa_2554 | D01592 | 0 | hsa_1815 | D08768 |
| 1 | hsa_80834 | D08500 | 0 | hsa_3061 | D02040 | 0 | hsa_5142 | D05523 |
| 1 | hsa_3356 | D08508 | 0 | hsa_5143 | D01978 | 0 | hsa_200909 | D04889 |
| 1 | hsa_150 | D08511 | 0 | hsa_552 | D01000 | 0 | hsa_5732 | D02040 |
| 1 | hsa_151 | D08511 | 0 | hsa_146 | D08525 | 0 | hsa_285242 | D01175 |
| 1 | hsa_152 | D08511 | 0 | hsa_150 | D08456 | 0 | hsa_10800 | D03622 |
| 1 | hsa_1909 | D08517 | 0 | hsa_799 | D10516 | 0 | hsa_5729 | D00819 |
| 1 | hsa_1131 | D08522 | 0 | hsa_799 | D07563 | 0 | hsa_1241 | D03937 |
| 1 | hsa_3757 | D08525 | 0 | hsa_56413 | D00678 | 0 | hsa_9177 | D01521 |
| 1 | hsa_1813 | D08549 | 0 | hsa_285242 | D10006 | 0 | hsa_285242 | D06274 |
| 1 | hsa_1813 | D08558 | 0 | hsa_55879 | D08578 | 0 | hsa_56413 | D01471 |
| 1 | hsa_148 | D08560 | 0 | hsa_1268 | D09698 | 0 | hsa_6608 | D03953 |
| 1 | hsa_147 | D08560 | 0 | hsa_5140 | D08497 | 0 | hsa_151 | D07458 |
| 1 | hsa_146 | D08560 | 0 | hsa_2565 | D01858 | 0 | hsa_3357 | D08861 |
| 1 | hsa_3350 | D08561 | 0 | hsa_5021 | D04717 | 0 | hsa_6752 | D01448 |
| 1 | hsa_148 | D08569 | 0 | hsa_59340 | D00669 | 0 | hsa_1814 | D05587 |
| 1 | hsa_147 | D08569 | 0 | hsa_2864 | D02357 | 0 | hsa_5731 | D08487 |
| 1 | hsa_146 | D08569 | 0 | hsa_2561 | D05007 | 0 | hsa_2912 | D03556 |
| 1 | hsa_154 | D08570 | 0 | hsa_2563 | D02901 | 0 | hsa_2864 | D02192 |
| 1 | hsa_552 | D08571 | 0 | hsa_6755 | D06157 | 0 | hsa_5732 | D03102 |
| 1 | hsa_153 | D08572 | 0 | hsa_2798 | D08358 | 0 | hsa_56413 | D10556 |
| 1 | hsa_146 | D08578 | 0 | hsa_1131 | D06147 | 0 | hsa_2693 | D01504 |
| 1 | hsa_147 | D08578 | 0 | hsa_2904 | D01386 | 0 | hsa_1131 | D02739 |
| 1 | hsa_148 | D08578 | 0 | hsa_11255 | D02834 | 0 | hsa_55879 | D10171 |
| 1 | hsa_1813 | D08585 | 0 | hsa_4543 | D08121 | 0 | hsa_56413 | D01007 |
| 1 | hsa_3269 | D08587 | 0 | hsa_3350 | D07460 | 0 | hsa_1241 | D07804 |
| 1 | hsa_1813 | D08590 | 0 | hsa_3355 | D04223 | 0 | hsa_2149 | D08991 |
| 1 | hsa_5737 | D08591 | 0 | hsa_1812 | D04494 | 0 | hsa_3352 | D01869 |
| 1 | hsa_64805 | D08594 | 0 | hsa_2550 | D02247 | 0 | hsa_2559 | D05649 |
| 1 | hsa_1128 | D08595 | 0 | hsa_1901 | D08322 | 0 | hsa_6755 | D01438 |
| 1 | hsa_1129 | D08595 | 0 | hsa_6608 | D01373 | 0 | hsa_2563 | D02109 |
| 1 | hsa_1131 | D08595 | 0 | hsa_3062 | D05343 | 0 | hsa_134 | D09344 |
| 1 | hsa_1132 | D08595 | 0 | hsa_2562 | D00498 | 0 | hsa_1129 | D01193 |
| 1 | hsa_1133 | D08595 | 0 | hsa_5141 | D01231 | 0 | hsa_5729 | D07976 |
| 1 | hsa_4988 | D08597 | 0 | hsa_2915 | D08376 | 0 | hsa_3356 | D02741 |
| 1 | hsa_153 | D08598 | 0 | hsa_1128 | D04502 | 0 | hsa_6870 | D02101 |
| 1 | hsa_153 | D08600 | 0 | hsa_1241 | D01051 | 0 | hsa_3062 | D10546 |
| 1 | hsa_150 | D08611 | 0 | hsa_2568 | D03982 | 0 | hsa_2556 | D05307 |
| 1 | hsa_151 | D08611 | 0 | hsa_135 | D08549 | 0 | hsa_1812 | D08235 |
| 1 | hsa_152 | D08611 | 0 | hsa_4889 | D08165 | 0 | hsa_154 | D08661 |
| 1 | hsa_146 | D08614 | 0 | hsa_3351 | D05939 | 0 | hsa_4986 | D09358 |
| 1 | hsa_147 | D08614 | 0 | hsa_1813 | D04710 | 0 | hsa_6752 | D06274 |
| 1 | hsa_148 | D08614 | 0 | hsa_5141 | D09359 | 0 | hsa_6532 | D00511 |
| 1 | hsa_4988 | D08623 | 0 | hsa_5144 | D07832 | 0 | hsa_10280 | D01600 |
| 1 | hsa_146 | D08624 | 0 | hsa_2560 | D02976 | 0 | hsa_285242 | D03937 |
| 1 | hsa_147 | D08624 | 0 | hsa_2568 | D00702 | 0 | hsa_2862 | D03725 |
| 1 | hsa_148 | D08624 | 0 | hsa_3362 | D01025 | 0 | hsa_4157 | D02725 |
| 1 | hsa_6532 | D08626 | 0 | hsa_6870 | D07976 | 0 | hsa_5139 | D04965 |
| 1 | hsa_5739 | D08628 | 0 | hsa_6753 | D10516 | 0 | hsa_146 | D08090 |
| 1 | hsa_154 | D08629 | 0 | hsa_3358 | D01512 | 0 | hsa_1394 | D03216 |
| 1 | hsa_2798 | D08635 | 0 | hsa_6571 | D09866 | 0 | hsa_134 | D01290 |
| 1 | hsa_1813 | D08636 | 0 | hsa_151 | D08611 | 0 | hsa_6915 | D01492 |
| 1 | hsa_1813 | D08637 | 0 | hsa_11255 | D01992 | 0 | hsa_6532 | D07122 |
| 1 | hsa_1128 | D08638 | 0 | hsa_1813 | D02069 | 0 | hsa_55879 | D02404 |
| 1 | hsa_1129 | D08638 | 0 | hsa_55879 | D04018 | 0 | hsa_6571 | D02775 |
| 1 | hsa_1131 | D08638 | 0 | hsa_5732 | D10185 | 0 | hsa_5724 | D09794 |
| 1 | hsa_1132 | D08638 | 0 | hsa_3350 | D01231 | 0 | hsa_5021 | D10430 |
| 1 | hsa_1133 | D08638 | 0 | hsa_5737 | D07713 | 0 | hsa_3360 | D09388 |
| 1 | hsa_1128 | D08639 | 0 | hsa_1901 | D06015 | 0 | hsa_5731 | D08122 |
| 1 | hsa_1129 | D08639 | 0 | hsa_3062 | D02040 | 0 | hsa_64805 | D02041 |
| 1 | hsa_1131 | D08639 | 0 | hsa_4889 | D02995 | 0 | hsa_2550 | D00291 |
| 1 | hsa_1132 | D08639 | 0 | hsa_3354 | D01343 | 0 | hsa_2562 | D02666 |
| 1 | hsa_1133 | D08639 | 0 | hsa_2566 | D00672 | 0 | hsa_80834 | D02693 |
| 1 | hsa_3269 | D08645 | 0 | hsa_3360 | D01521 | 0 | hsa_6570 | D06328 |
| 1 | hsa_3269 | D08648 | 0 | hsa_1131 | D08093 | 0 | hsa_2864 | D07463 |
| 1 | hsa_2798 | D08649 | 0 | hsa_3269 | D09797 | 0 | hsa_2550 | D09752 |
| 1 | hsa_5737 | D08661 | 0 | hsa_3357 | D02041 | 0 | hsa_4986 | D03814 |
| 1 | hsa_146 | D08662 | 0 | hsa_554 | D00721 | 0 | hsa_554 | D05711 |
| 1 | hsa_147 | D08662 | 0 | hsa_2550 | D07099 | 0 | hsa_9568 | D00843 |
| 1 | hsa_148 | D08662 | 0 | hsa_11255 | D02623 | 0 | hsa_2565 | D03504 |
| 1 | hsa_150 | D08683 | 0 | hsa_2568 | D01377 | 0 | hsa_10800 | D02968 |
| 1 | hsa_151 | D08683 | 0 | hsa_2568 | D08901 | 0 | hsa_2558 | D07534 |
| 1 | hsa_152 | D08683 | 0 | hsa_80834 | D00602 | 0 | hsa_624 | D01946 |
| 1 | hsa_146 | D08684 | 0 | hsa_5729 | D04823 | 0 | hsa_200909 | D01795 |
| 1 | hsa_147 | D08684 | 0 | hsa_5142 | D09977 | 0 | hsa_7201 | D02040 |
| 1 | hsa_148 | D08684 | 0 | hsa_170572 | D00672 | 0 | hsa_11255 | D08495 |
| 1 | hsa_150 | D08685 | 0 | hsa_2915 | D04708 | 0 | hsa_59340 | D02248 |
| 1 | hsa_151 | D08685 | 0 | hsa_2912 | D10736 | 0 | hsa_5724 | D06157 |
| 1 | hsa_152 | D08685 | 0 | hsa_1394 | D02663 | 0 | hsa_1813 | D09319 |
| 1 | hsa_3356 | D08687 | 0 | hsa_2912 | D08157 | 0 | hsa_2565 | D00787 |
| 1 | hsa_3357 | D08687 | 0 | hsa_6751 | D10128 | 0 | hsa_64805 | D02092 |
| 1 | hsa_3358 | D08687 | 0 | hsa_5732 | D07511 | 0 | hsa_2550 | D03009 |
| 1 | hsa_1813 | D08691 | 0 | hsa_6571 | D10219 | 0 | hsa_3352 | D02417 |
| 1 | hsa_1813 | D08692 | 0 | hsa_10800 | D01026 | 0 | hsa_154 | D08325 |
| 1 | hsa_1813 | D08693 | 0 | hsa_185 | D04924 | 0 | hsa_11255 | D08322 |
| 1 | hsa_3269 | D08768 | 0 | hsa_55879 | D07931 | 0 | hsa_3360 | D02016 |
| 1 | hsa_80834 | D08836 | 0 | hsa_2798 | D08969 | 0 | hsa_5140 | D10119 |
| 1 | hsa_1128 | D08837 | 0 | hsa_2560 | D04642 | 0 | hsa_5737 | D08376 |
| 1 | hsa_1129 | D08837 | 0 | hsa_147 | D07229 | 0 | hsa_152 | D02605 |
| 1 | hsa_1131 | D08837 | 0 | hsa_3269 | D00842 | 0 | hsa_4543 | D02381 |
| 1 | hsa_1132 | D08837 | 0 | hsa_4543 | D01006 | 0 | hsa_3354 | D08093 |
| 1 | hsa_1133 | D08837 | 0 | hsa_2565 | D06015 | 0 | hsa_3757 | D00756 |
| 1 | hsa_43 | D08838 | 0 | hsa_2568 | D08353 | 0 | hsa_1129 | D00778 |
| 1 | hsa_155 | D08850 | 0 | hsa_3354 | D01869 | 0 | hsa_2565 | D02689 |
| 1 | hsa_155 | D08851 | 0 | hsa_6753 | D02613 | 0 | hsa_5142 | D01358 |
| 1 | hsa_2693 | D08856 | 0 | hsa_3356 | D09845 | 0 | hsa_3351 | D10478 |
| 1 | hsa_2550 | D08861 | 0 | hsa_2904 | D06248 | 0 | hsa_2864 | D07946 |
| 1 | hsa_9568 | D08861 | 0 | hsa_151 | D07838 | 0 | hsa_146 | D04973 |
| 1 | hsa_80834 | D08862 | 0 | hsa_150 | D04965 | 0 | hsa_2693 | D09791 |
| 1 | hsa_185 | D08864 | 0 | hsa_2915 | D06660 | 0 | hsa_3359 | D02091 |
| 1 | hsa_185 | D08865 | 0 | hsa_3350 | D01315 | 0 | hsa_285242 | D07682 |
| 1 | hsa_2798 | D08901 | 0 | hsa_3359 | D01026 | 0 | hsa_10203 | D02684 |
| 1 | hsa_1128 | D08923 | 0 | hsa_1129 | D01875 | 0 | hsa_624 | D02834 |
| 1 | hsa_1129 | D08923 | 0 | hsa_56413 | D00843 | 0 | hsa_2912 | D04531 |
| 1 | hsa_1131 | D08923 | 0 | hsa_5142 | D01635 | 0 | hsa_5142 | D07522 |
| 1 | hsa_1132 | D08923 | 0 | hsa_2568 | D10436 | 0 | hsa_1268 | D07906 |
| 1 | hsa_1133 | D08923 | 0 | hsa_2568 | D09765 | 0 | hsa_4985 | D08989 |
| 1 | hsa_624 | D08937 | 0 | hsa_6570 | D09954 | 0 | hsa_5142 | D03102 |
| 1 | hsa_5729 | D08940 | 0 | hsa_1815 | D01521 | 0 | hsa_5729 | D00844 |
| 1 | hsa_154 | D08945 | 0 | hsa_154 | D03580 | 0 | hsa_1901 | D01855 |
| 1 | hsa_146 | D08966 | 0 | hsa_6755 | D00124 | 0 | hsa_3359 | D10330 |
| 1 | hsa_147 | D08966 | 0 | hsa_2904 | D09318 | 0 | hsa_6751 | D03506 |
| 1 | hsa_148 | D08966 | 0 | hsa_5140 | D05732 | 0 | hsa_55879 | D08623 |
| 1 | hsa_3356 | D08969 | 0 | hsa_2560 | D08170 | 0 | hsa_10203 | D05732 |
| 1 | hsa_64805 | D08983 | 0 | hsa_148 | D05010 | 0 | hsa_1901 | D05718 |
| 1 | hsa_5021 | D08986 | 0 | hsa_4889 | D01551 | 0 | hsa_3358 | D02693 |
| 1 | hsa_6869 | D08988 | 0 | hsa_6571 | D01077 | 0 | hsa_9177 | D04361 |
| 1 | hsa_134 | D08989 | 0 | hsa_148 | D08041 | 0 | hsa_3062 | D02590 |
| 1 | hsa_846 | D08991 | 0 | hsa_10280 | D02730 | 0 | hsa_6752 | D07929 |
| 1 | hsa_135 | D09003 | 0 | hsa_6753 | D05395 | 0 | hsa_6755 | D00798 |
| 1 | hsa_2912 | D09008 | 0 | hsa_154 | D04611 | 0 | hsa_1128 | D01741 |
| 1 | hsa_2913 | D09008 | 0 | hsa_2558 | D08170 | 0 | hsa_1813 | D02102 |
| 1 | hsa_1268 | D09009 | 0 | hsa_5021 | D05478 | 0 | hsa_59340 | D03402 |
| 1 | hsa_64805 | D09017 | 0 | hsa_5729 | D04501 | 0 | hsa_64805 | D00540 |
| 1 | hsa_3360 | D09205 | 0 | hsa_150 | D07951 | 0 | hsa_1128 | D04626 |
| 1 | hsa_154 | D09318 | 0 | hsa_9568 | D07493 | 0 | hsa_1129 | D10349 |
| 1 | hsa_154 | D09319 | 0 | hsa_5144 | D02235 | 0 | hsa_2693 | D01522 |
| 1 | hsa_2798 | D09335 | 0 | hsa_5734 | D08205 | 0 | hsa_7201 | D08253 |
| 1 | hsa_2798 | D09336 | 0 | hsa_3360 | D04500 | 0 | hsa_134 | D02101 |
| 1 | hsa_155 | D09344 | 0 | hsa_3362 | D02212 | 0 | hsa_2563 | D08549 |
| 1 | hsa_1268 | D09349 | 0 | hsa_3061 | D04361 | 0 | hsa_2798 | D08046 |
| 1 | hsa_3350 | D09358 | 0 | hsa_4889 | D09766 | 0 | hsa_5732 | D08157 |
| 1 | hsa_3350 | D09359 | 0 | hsa_2912 | D10099 | 0 | hsa_6344 | D10676 |
| 1 | hsa_1268 | D09362 | 0 | hsa_6570 | D01624 | 0 | hsa_2915 | D02850 |
| 1 | hsa_1268 | D09363 | 0 | hsa_2562 | D05008 | 0 | hsa_6754 | D08249 |
| 1 | hsa_1813 | D09366 | 0 | hsa_2862 | D03711 | 0 | hsa_2557 | D00605 |
| 1 | hsa_6869 | D09378 | 0 | hsa_6754 | D05011 | 0 | hsa_2554 | D09949 |
| 1 | hsa_4543 | D09388 | 0 | hsa_2915 | D02071 | 0 | hsa_3351 | D05099 |
| 1 | hsa_10203 | D09391 | 0 | hsa_3350 | D02730 | 0 | hsa_3360 | D04018 |
| 1 | hsa_10203 | D09392 | 0 | hsa_624 | D02825 | 0 | hsa_5142 | D07537 |
| 1 | hsa_1813 | D09397 | 0 | hsa_5140 | D02363 | 0 | hsa_846 | D01451 |
| 1 | hsa_2798 | D09400 | 0 | hsa_5737 | D07878 | 0 | hsa_3359 | D08235 |
| 1 | hsa_1128 | D09402 | 0 | hsa_2864 | D09964 | 0 | hsa_554 | D10199 |
| 1 | hsa_1129 | D09402 | 0 | hsa_6869 | D06328 | 0 | hsa_6571 | D05099 |
| 1 | hsa_1131 | D09402 | 0 | hsa_1241 | D01051 | 0 | hsa_80834 | D02846 |
| 1 | hsa_1132 | D09402 | 0 | hsa_6344 | D00662 | 0 | hsa_2915 | D04680 |
| 1 | hsa_1133 | D09402 | 0 | hsa_2568 | D00371 | 0 | hsa_2562 | D01005 |
| 1 | hsa_155 | D09535 | 0 | hsa_1815 | D01307 | 0 | hsa_6755 | D00798 |
| 1 | hsa_3269 | D09570 | 0 | hsa_6869 | D07803 | 0 | hsa_43 | D10119 |
| 1 | hsa_64805 | D09607 | 0 | hsa_4889 | D07887 | 0 | hsa_624 | D06569 |
| 1 | hsa_64805 | D09608 | 0 | hsa_2561 | D10129 | 0 | hsa_2556 | D02388 |
| 1 | hsa_1394 | D09610 | 0 | hsa_2557 | D04370 | 0 | hsa_4543 | D01691 |
| 1 | hsa_3356 | D09645 | 0 | hsa_3355 | D05587 | 0 | hsa_185 | D08160 |
| 1 | hsa_6869 | D09650 | 0 | hsa_55879 | D09697 | 0 | hsa_6532 | D00559 |
| 1 | hsa_6869 | D09651 | 0 | hsa_1128 | D05606 | 0 | hsa_59340 | D00842 |
| 1 | hsa_134 | D09684 | 0 | hsa_80834 | D08578 | 0 | hsa_6869 | D00308 |
| 1 | hsa_3360 | D09693 | 0 | hsa_2149 | D08065 | 0 | hsa_10280 | D01355 |
| 1 | hsa_3360 | D09694 | 0 | hsa_59340 | D09693 | 0 | hsa_3274 | D01691 |
| 1 | hsa_1394 | D09695 | 0 | hsa_3359 | D08160 | 0 | hsa_3357 | D00089 |
| 1 | hsa_154 | D09696 | 0 | hsa_2555 | D08594 | 0 | hsa_2561 | D10562 |
| 1 | hsa_154 | D09697 | 0 | hsa_1814 | D10174 | 0 | hsa_1812 | D07463 |
| 1 | hsa_3350 | D09698 | 0 | hsa_3274 | D00481 | 0 | hsa_5144 | D05085 |
| 1 | hsa_3350 | D09699 | 0 | hsa_5729 | D02073 | 0 | hsa_6870 | D08115 |
| 1 | hsa_3269 | D09705 | 0 | hsa_1814 | D09391 | 0 | hsa_1901 | D09397 |
| 1 | hsa_135 | D09717 | 0 | hsa_3274 | D02681 | 0 | hsa_1815 | D03953 |
| 1 | hsa_155 | D09732 | 0 | hsa_9568 | D06103 | 0 | hsa_6608 | D07853 |
| 1 | hsa_3359 | D09749 | 0 | hsa_5141 | D10478 | 0 | hsa_152 | D00371 |
| 1 | hsa_9177 | D09749 | 0 | hsa_5140 | D09766 | 0 | hsa_3354 | D01358 |
| 1 | hsa_170572 | D09749 | 0 | hsa_146 | D01818 | 0 | hsa_5734 | D08645 |
| 1 | hsa_200909 | D09749 | 0 | hsa_2559 | D08560 | 0 | hsa_2904 | D08010 |
| 1 | hsa_285242 | D09749 | 0 | hsa_10280 | D02016 | 0 | hsa_6571 | D02163 |
| 1 | hsa_1128 | D09752 | 0 | hsa_185 | D07461 | 0 | hsa_3352 | D01231 |
| 1 | hsa_1129 | D09752 | 0 | hsa_3355 | D08485 | 0 | hsa_150 | D04500 |
| 1 | hsa_1131 | D09752 | 0 | hsa_59340 | D10662 | 0 | hsa_3362 | D02236 |
| 1 | hsa_1132 | D09752 | 0 | hsa_170572 | D10433 | 0 | hsa_2562 | D09845 |
| 1 | hsa_1133 | D09752 | 0 | hsa_2913 | D03535 | 0 | hsa_134 | D00458 |
| 1 | hsa_2149 | D09765 | 0 | hsa_11255 | D02071 | 0 | hsa_55879 | D09535 |
| 1 | hsa_2149 | D09766 | 0 | hsa_3355 | D04531 | 0 | hsa_5144 | D06021 |
| 1 | hsa_6915 | D09774 | 0 | hsa_552 | D00378 | 0 | hsa_80834 | D05649 |
| 1 | hsa_148 | D09784 | 0 | hsa_6752 | D03672 | 0 | hsa_4985 | D07906 |
| 1 | hsa_2550 | D09791 | 0 | hsa_5729 | D01172 | 0 | hsa_3350 | D00366 |
| 1 | hsa_9568 | D09791 | 0 | hsa_9177 | D02073 | 0 | hsa_43 | D07312 |
| 1 | hsa_146 | D09794 | 0 | hsa_4985 | D05902 | 0 | hsa_2693 | D01355 |
| 1 | hsa_147 | D09794 | 0 | hsa_3358 | D07862 | 0 | hsa_4987 | D05738 |
| 1 | hsa_148 | D09794 | 0 | hsa_4988 | D01349 | 0 | hsa_59340 | D02730 |
| 1 | hsa_5021 | D09797 | 0 | hsa_10800 | D10324 | 0 | hsa_6755 | D03556 |
| 1 | hsa_154 | D09842 | 0 | hsa_2555 | D01025 | 0 | hsa_10203 | D02071 |
| 1 | hsa_7201 | D09845 | 0 | hsa_10800 | D04494 | 0 | hsa_5731 | D08286 |
| 1 | hsa_2149 | D09866 | 0 | hsa_5021 | D03177 | 0 | hsa_5021 | D09866 |
| 1 | hsa_2149 | D09867 | 0 | hsa_3269 | D10375 | 0 | hsa_2564 | D08595 |
| 1 | hsa_1814 | D09876 | 0 | hsa_2798 | D01445 | 0 | hsa_152 | D04794 |
| 1 | hsa_3360 | D09933 | 0 | hsa_2864 | D10185 | 0 | hsa_1814 | D01685 |
| 1 | hsa_3360 | D09934 | 0 | hsa_886 | D00997 | 0 | hsa_1268 | D02983 |
| 1 | hsa_2912 | D09949 | 0 | hsa_2567 | D08637 | 0 | hsa_185 | D03198 |
| 1 | hsa_2913 | D09949 | 0 | hsa_6754 | D07905 | 0 | hsa_2560 | D06353 |
| 1 | hsa_1813 | D09953 | 0 | hsa_148 | D02765 | 0 | hsa_846 | D00025 |
| 1 | hsa_1813 | D09954 | 0 | hsa_2558 | D07809 | 0 | hsa_5139 | D10219 |
| 1 | hsa_3061 | D09964 | 0 | hsa_2567 | D06660 | 0 | hsa_2556 | D07560 |
| 1 | hsa_3062 | D09964 | 0 | hsa_6751 | D04116 | 0 | hsa_2565 | D02739 |
| 1 | hsa_3356 | D09976 | 0 | hsa_2556 | D08923 | 0 | hsa_2566 | D02983 |
| 1 | hsa_3356 | D09977 | 0 | hsa_56413 | D01085 | 0 | hsa_6752 | D02684 |
| 1 | hsa_2693 | D09981 | 0 | hsa_552 | D07460 | 0 | hsa_5729 | D08234 |
| 1 | hsa_2693 | D09982 | 0 | hsa_6570 | D06234 | 0 | hsa_152 | D07491 |
| 1 | hsa_6751 | D09988 | 0 | hsa_2567 | D08384 | 0 | hsa_6571 | D04765 |
| 1 | hsa_5729 | D09990 | 0 | hsa_3350 | D01462 | 0 | hsa_2904 | D03622 |
| 1 | hsa_135 | D09991 | 0 | hsa_4157 | D08635 | 0 | hsa_1815 | D01348 |
| 1 | hsa_6608 | D09992 | 0 | hsa_552 | D06646 | 0 | hsa_56413 | D02964 |
| 1 | hsa_5739 | D09994 | 0 | hsa_170572 | D00076 | 0 | hsa_2693 | D10184 |
| 1 | hsa_1814 | D09997 | 0 | hsa_2912 | D07832 | 0 | hsa_6752 | D08234 |
| 1 | hsa_1901 | D10001 | 0 | hsa_10800 | D01269 | 0 | hsa_3356 | D04494 |
| 1 | hsa_3356 | D10006 | 0 | hsa_552 | D07665 | 0 | hsa_200909 | D00634 |
| 1 | hsa_154 | D10020 | 0 | hsa_3269 | D02208 | 0 | hsa_2912 | D03504 |
| 1 | hsa_1394 | D10022 | 0 | hsa_5734 | D07493 | 0 | hsa_6608 | D08838 |
| 1 | hsa_3362 | D10099 | 0 | hsa_4157 | D02381 | 0 | hsa_2562 | D00076 |
| 1 | hsa_3362 | D10100 | 0 | hsa_6755 | D09349 | 0 | hsa_6532 | D02568 |
| 1 | hsa_5021 | D10117 | 0 | hsa_2557 | D03494 | 0 | hsa_5141 | D09992 |
| 1 | hsa_5021 | D10118 | 0 | hsa_185 | D00756 | 0 | hsa_1812 | D02739 |
| 1 | hsa_6608 | D10119 | 0 | hsa_5139 | D02981 | 0 | hsa_3062 | D07451 |
| 1 | hsa_11255 | D10128 | 0 | hsa_10800 | D05085 | 0 | hsa_846 | D01929 |
| 1 | hsa_11255 | D10129 | 0 | hsa_151 | D07887 | 0 | hsa_134 | D04683 |
| 1 | hsa_1909 | D10135 | 0 | hsa_2556 | D08517 | 0 | hsa_2912 | D02577 |
| 1 | hsa_154 | D10145 | 0 | hsa_4986 | D02111 | 0 | hsa_3062 | D00481 |
| 1 | hsa_6755 | D10147 | 0 | hsa_2904 | D08549 | 0 | hsa_3269 | D02645 |
| 1 | hsa_3360 | D10152 | 0 | hsa_9177 | D09349 | 0 | hsa_3352 | D06140 |
| 1 | hsa_6570 | D10170 | 0 | hsa_1133 | D09732 | 0 | hsa_2555 | D04765 |
| 1 | hsa_6571 | D10170 | 0 | hsa_6869 | D07729 | 0 | hsa_6870 | D01468 |
| 1 | hsa_6570 | D10171 | 0 | hsa_552 | D08220 | 0 | hsa_2555 | D04223 |
| 1 | hsa_6571 | D10171 | 0 | hsa_4985 | D02417 | 0 | hsa_3352 | D05682 |
| 1 | hsa_135 | D10174 | 0 | hsa_2559 | D07617 | 0 | hsa_1812 | D07398 |
| 1 | hsa_1128 | D10180 | 0 | hsa_3352 | D01551 | 0 | hsa_59340 | D07122 |
| 1 | hsa_1129 | D10180 | 0 | hsa_5142 | D01485 | 0 | hsa_9568 | D00371 |
| 1 | hsa_1131 | D10180 | 0 | hsa_6753 | D02372 | 0 | hsa_6870 | D01307 |
| 1 | hsa_1132 | D10180 | 0 | hsa_1901 | D07803 | 0 | hsa_285242 | D03402 |
| 1 | hsa_1133 | D10180 | 0 | hsa_2566 | D01445 | 0 | hsa_10800 | D06344 |
| 1 | hsa_1128 | D10181 | 0 | hsa_3360 | D04087 | 0 | hsa_2915 | D08074 |
| 1 | hsa_1129 | D10181 | 0 | hsa_3757 | D04823 | 0 | hsa_554 | D07076 |
| 1 | hsa_1131 | D10181 | 0 | hsa_6915 | D08561 | 0 | hsa_3274 | D08645 |
| 1 | hsa_1132 | D10181 | 0 | hsa_552 | D03495 | 0 | hsa_554 | D01987 |
| 1 | hsa_1133 | D10181 | 0 | hsa_3354 | D03556 | 0 | hsa_6344 | D04694 |
| 1 | hsa_3359 | D10184 | 0 | hsa_7201 | D08443 | 0 | hsa_4986 | D08692 |
| 1 | hsa_9177 | D10184 | 0 | hsa_1131 | D09696 | 0 | hsa_5143 | D08473 |
| 1 | hsa_170572 | D10184 | 0 | hsa_3352 | D00374 | 0 | hsa_1128 | D10612 |
| 1 | hsa_200909 | D10184 | 0 | hsa_6751 | D10220 | 0 | hsa_9177 | D01831 |
| 1 | hsa_285242 | D10184 | 0 | hsa_150 | D01543 | 0 | hsa_285242 | D05312 |
| 1 | hsa_3359 | D10185 | 0 | hsa_1128 | D05004 | 0 | hsa_5142 | D10119 |
| 1 | hsa_9177 | D10185 | 0 | hsa_285242 | D08318 | 0 | hsa_2555 | D07550 |
| 1 | hsa_170572 | D10185 | 0 | hsa_846 | D03399 | 0 | hsa_10280 | D05107 |
| 1 | hsa_200909 | D10185 | 0 | hsa_5732 | D00602 | 0 | hsa_2561 | D08623 |
| 1 | hsa_285242 | D10185 | 0 | hsa_5732 | D10403 | 0 | hsa_1132 | D07887 |
| 1 | hsa_4988 | D10188 | 0 | hsa_3351 | D06632 | 0 | hsa_1815 | D10563 |
| 1 | hsa_4988 | D10199 | 0 | hsa_135 | D00722 | 0 | hsa_6608 | D02730 |
| 1 | hsa_154 | D10219 | 0 | hsa_3061 | D02092 | 0 | hsa_10280 | D08865 |
| 1 | hsa_154 | D10220 | 0 | hsa_11255 | D03704 | 0 | hsa_6754 | D05575 |
| 1 | hsa_1813 | D10309 | 0 | hsa_4988 | D01462 | 0 | hsa_1901 | D02205 |
| 1 | hsa_6608 | D10324 | 0 | hsa_3269 | D10516 | 0 | hsa_6608 | D02568 |
| 1 | hsa_6608 | D10325 | 0 | hsa_2559 | D07977 | 0 | hsa_135 | D03415 |
| 1 | hsa_5729 | D10326 | 0 | hsa_10203 | D07990 | 0 | hsa_2568 | D08305 |
| 1 | hsa_2862 | D10330 | 0 | hsa_3062 | D01589 | 0 | hsa_6608 | D01460 |
| 1 | hsa_2864 | D10336 | 0 | hsa_2561 | D00603 | 0 | hsa_7201 | D01349 |
| 1 | hsa_3355 | D10338 | 0 | hsa_1133 | D00498 | 0 | hsa_1812 | D02381 |
| 1 | hsa_3061 | D10345 | 0 | hsa_2904 | D07593 | 0 | hsa_7201 | D04034 |
| 1 | hsa_3062 | D10345 | 0 | hsa_2566 | D04494 | 0 | hsa_799 | D09610 |
| 1 | hsa_4988 | D10349 | 0 | hsa_2798 | D10545 | 0 | hsa_285242 | D05649 |
| 1 | hsa_1813 | D10355 | 0 | hsa_1128 | D00796 | 0 | hsa_80834 | D01654 |
| 1 | hsa_135 | D10362 | 0 | hsa_2565 | D04708 | 0 | hsa_2550 | D08067 |
| 1 | hsa_1813 | D10364 | 0 | hsa_5140 | D06355 | 0 | hsa_1131 | D10220 |
| 1 | hsa_4988 | D10371 | 0 | hsa_5737 | D08572 | 0 | hsa_1909 | D06328 |
| 1 | hsa_4988 | D10375 | 0 | hsa_2558 | D07734 | 0 | hsa_6869 | D06281 |
| 1 | hsa_6869 | D10391 | 0 | hsa_6532 | D09949 | 0 | hsa_2559 | D07821 |
| 1 | hsa_4988 | D10403 | 0 | hsa_2555 | D02801 | 0 | hsa_1901 | D01955 |
| 1 | hsa_3355 | D10424 | 0 | hsa_4889 | D03449 | 0 | hsa_200909 | D00633 |
| 1 | hsa_5739 | D10430 | 0 | hsa_6752 | D02535 | 0 | hsa_5731 | D08923 |
| 1 | hsa_155 | D10433 | 0 | hsa_2564 | D01269 | 0 | hsa_2567 | D00559 |
| 1 | hsa_4987 | D10436 | 0 | hsa_6752 | D09008 | 0 | hsa_6753 | D08395 |
| 1 | hsa_5737 | D10441 | 0 | hsa_2558 | D02071 | 0 | hsa_846 | D07538 |
| 1 | hsa_4988 | D10478 | 0 | hsa_4986 | D01835 | 0 | hsa_43 | D06253 |
| 1 | hsa_4988 | D10479 | 0 | hsa_55879 | D01123 | 0 | hsa_6608 | D01929 |
| 1 | hsa_134 | D10493 | 0 | hsa_2864 | D00681 | 0 | hsa_170572 | D01958 |
| 1 | hsa_6755 | D10497 | 0 | hsa_6753 | D09797 | 0 | hsa_554 | D09400 |
| 1 | hsa_1813 | D10516 | 0 | hsa_148 | D08585 | 0 | hsa_4889 | D08466 |
| 1 | hsa_1128 | D10545 | 0 | hsa_2568 | D07740 | 0 | hsa_3360 | D05732 |
| 1 | hsa_1129 | D10545 | 0 | hsa_6532 | D04532 | 0 | hsa_3062 | D02613 |
| 1 | hsa_1131 | D10545 | 0 | hsa_5021 | D04514 | 0 | hsa_9177 | D10338 |
| 1 | hsa_1132 | D10545 | 0 | hsa_3061 | D09535 | 0 | hsa_2554 | D10692 |
| 1 | hsa_1133 | D10545 | 0 | hsa_2560 | D05107 | 0 | hsa_155 | D06552 |
| 1 | hsa_1128 | D10546 | 0 | hsa_134 | D02801 | 0 | hsa_9177 | D01445 |
| 1 | hsa_1129 | D10546 | 0 | hsa_3357 | D08209 | 0 | hsa_2864 | D06566 |
| 1 | hsa_1131 | D10546 | 0 | hsa_3360 | D04641 | 0 | hsa_7201 | D01007 |
| 1 | hsa_1132 | D10546 | 0 | hsa_1812 | D03506 | 0 | hsa_3356 | D08395 |
| 1 | hsa_1133 | D10546 | 0 | hsa_3352 | D02069 | 0 | hsa_2564 | D04701 |
| 1 | hsa_185 | D10556 | 0 | hsa_2565 | D10433 | 0 | hsa_4157 | D02343 |
| 1 | hsa_2693 | D10562 | 0 | hsa_3354 | D10478 | 0 | hsa_285242 | D00456 |
| 1 | hsa_2693 | D10563 | 0 | hsa_3350 | D10099 | 0 | hsa_4988 | D07171 |
| 1 | hsa_6755 | D10566 | 0 | hsa_5734 | D02245 | 0 | hsa_6751 | D00798 |
| 1 | hsa_2864 | D10567 | 0 | hsa_3350 | D00842 | 0 | hsa_6870 | D09867 |
| 1 | hsa_4988 | D10612 | 0 | hsa_1129 | D05731 | 0 | hsa_153 | D02381 |
| 1 | hsa_5739 | D10628 | 0 | hsa_5731 | D07113 | 0 | hsa_2693 | D08184 |
| 1 | hsa_5729 | D10631 | 0 | hsa_6869 | D03814 | 0 | hsa_5143 | D03618 |
| 1 | hsa_6608 | D10636 | 0 | hsa_153 | D05010 | 0 | hsa_5737 | D00664 |
| 1 | hsa_5734 | D10638 | 0 | hsa_2565 | D04087 | 0 | hsa_3362 | D03704 |
| 1 | hsa_4988 | D10648 | 0 | hsa_3062 | D06552 | 0 | hsa_799 | D10516 |
| 1 | hsa_2693 | D10660 | 0 | hsa_6870 | D03704 | 0 | hsa_3062 | D01188 |
| 1 | hsa_10203 | D10662 | 0 | hsa_1128 | D02590 | 0 | hsa_1132 | D04765 |
| 1 | hsa_10203 | D10663 | 0 | hsa_3274 | D00481 | 0 | hsa_3351 | D07662 |
| 1 | hsa_6608 | D10671 | 0 | hsa_147 | D00529 | 0 | hsa_5729 | D06671 |
| 1 | hsa_846 | D10676 | 0 | hsa_2862 | D01101 | 0 | hsa_6344 | D03953 |
| 1 | hsa_846 | D10677 | 0 | hsa_5141 | D00308 | 0 | hsa_7201 | D08105 |
| 1 | hsa_4988 | D10690 | 0 | hsa_2560 | D04889 | 0 | hsa_3757 | D08212 |
| 1 | hsa_4988 | D10692 | 0 | hsa_1813 | D06007 | 0 | hsa_6532 | D06268 |
| 1 | hsa_5739 | D10703 | 0 | hsa_4985 | D07860 | 0 | hsa_3358 | D07099 |
| 1 | hsa_3362 | D10710 | 0 | hsa_5141 | D02208 | 0 | hsa_152 | D03009 |
| 1 | hsa_5739 | D10725 | 0 | hsa_147 | D03725 | 0 | hsa_3362 | D01835 |
| 1 | hsa_6608 | D10729 | 0 | hsa_5734 | D10677 | 0 | hsa_6754 | D07862 |
| 1 | hsa_59340 | D10735 | 0 | hsa_11255 | D01815 | 0 | hsa_4987 | D01976 |
| 1 | hsa_59340 | D10736 | 0 | hsa_2563 | D02035 | 0 | hsa_135 | D04502 |
| 1 | hsa_4988 | D10740 | 0 | hsa_1394 | D01898 | 0 | hsa_3351 | D02016 |
| 1 | hsa_6869 | D10742 | 0 | hsa_5142 | D10326 | 0 | hsa_2915 | D05476 |
| 1 | hsa_3362 | D10747 | 0 | hsa_2556 | D00481 | 0 | hsa_4987 | D01205 |

Table IV The overall of drug-target interactions for nuclear receptors class on *Dataset2*

| **class** | **Targets** | **Drugs** | **class** | **Targets** | **Drugs** | **class** | **Targets** | **Drugs** |
| --- | --- | --- | --- | --- | --- | --- | --- | --- |
| 1 | hsa_7421 | D00129 | 0 | hsa_367 | D00129 | 0 | hsa_6258 | D04214 |
| 1 | hsa_5914 | D00164 | 0 | hsa_5915 | D00129 | 0 | hsa_7421 | D04214 |
| 1 | hsa_5915 | D00164 | 0 | hsa_6257 | D00129 | 0 | hsa_367 | D04217 |
| 1 | hsa_5916 | D00164 | 0 | hsa_7421 | D00129 | 0 | hsa_5465 | D04217 |
| 1 | hsa_2908 | D00165 | 0 | hsa_5241 | D00164 | 0 | hsa_5914 | D04217 |
| 1 | hsa_2099 | D00185 | 0 | hsa_5468 | D00164 | 0 | hsa_7068 | D04217 |
| 1 | hsa_2908 | D00244 | 0 | hsa_9971 | D00164 | 0 | hsa_5467 | D04218 |
| 1 | hsa_367 | D00289 | 0 | hsa_367 | D00165 | 0 | hsa_5467 | D04218 |
| 1 | hsa_2908 | D00292 | 0 | hsa_5916 | D00165 | 0 | hsa_5914 | D04218 |
| 1 | hsa_2908 | D00324 | 0 | hsa_7068 | D00165 | 0 | hsa_5467 | D04219 |
| 1 | hsa_2908 | D00325 | 0 | hsa_367 | D00185 | 0 | hsa_7421 | D04219 |
| 1 | hsa_2908 | D00328 | 0 | hsa_4306 | D00185 | 0 | hsa_2100 | D04221 |
| 1 | hsa_5465 | D00334 | 0 | hsa_5914 | D00185 | 0 | hsa_367 | D04221 |
| 1 | hsa_5915 | D00348 | 0 | hsa_5915 | D00185 | 0 | hsa_5916 | D04221 |
| 1 | hsa_5916 | D00348 | 0 | hsa_5916 | D00185 | 0 | hsa_6258 | D04221 |
| 1 | hsa_7067 | D00361 | 0 | hsa_6258 | D00185 | 0 | hsa_9971 | D04316 |
| 1 | hsa_2908 | D00385 | 0 | hsa_7421 | D00185 | 0 | hsa_5241 | D04409 |
| 1 | hsa_367 | D00389 | 0 | hsa_6258 | D00244 | 0 | hsa_5468 | D04409 |
| 1 | hsa_5468 | D00395 | 0 | hsa_7067 | D00244 | 0 | hsa_7068 | D04409 |
| 1 | hsa_2908 | D00407 | 0 | hsa_2100 | D00289 | 0 | hsa_6257 | D04467 |
| 1 | hsa_367 | D00408 | 0 | hsa_4306 | D00289 | 0 | hsa_7421 | D04467 |
| 1 | hsa_367 | D00444 | 0 | hsa_5467 | D00289 | 0 | hsa_4306 | D04496 |
| 1 | hsa_2908 | D00472 | 0 | hsa_5916 | D00289 | 0 | hsa_7067 | D04636 |
| 1 | hsa_2908 | D00473 | 0 | hsa_5915 | D00292 | 0 | hsa_7421 | D04636 |
| 1 | hsa_367 | D00490 | 0 | hsa_5916 | D00292 | 0 | hsa_9971 | D04636 |
| 1 | hsa_2099 | D00575 | 0 | hsa_7068 | D00324 | 0 | hsa_367 | D04672 |
| 1 | hsa_2908 | D00689 | 0 | hsa_7421 | D00324 | 0 | hsa_5241 | D04672 |
| 1 | hsa_2908 | D00690 | 0 | hsa_5468 | D00385 | 0 | hsa_5914 | D04672 |
| 1 | hsa_2908 | D00751 | 0 | hsa_5915 | D00385 | 0 | hsa_5915 | D04672 |
| 1 | hsa_5468 | D00945 | 0 | hsa_2100 | D00389 | 0 | hsa_7068 | D04672 |
| 1 | hsa_2099 | D00946 | 0 | hsa_6257 | D00389 | 0 | hsa_2100 | D04885 |
| 1 | hsa_2099 | D00948 | 0 | hsa_9971 | D00389 | 0 | hsa_5916 | D04885 |
| 1 | hsa_5241 | D00949 | 0 | hsa_7421 | D00395 | 0 | hsa_2099 | D04947 |
| 1 | hsa_5241 | D00952 | 0 | hsa_367 | D00407 | 0 | hsa_5914 | D04947 |
| 1 | hsa_5241 | D00953 | 0 | hsa_2100 | D00408 | 0 | hsa_6258 | D04947 |
| 1 | hsa_367 | D00955 | 0 | hsa_2100 | D00472 | 0 | hsa_4306 | D05000 |
| 1 | hsa_367 | D00957 | 0 | hsa_5241 | D00472 | 0 | hsa_6258 | D05000 |
| 1 | hsa_367 | D00958 | 0 | hsa_7421 | D00472 | 0 | hsa_7068 | D05000 |
| 1 | hsa_367 | D00959 | 0 | hsa_2099 | D00473 | 0 | hsa_5241 | D05001 |
| 1 | hsa_2099 | D00966 | 0 | hsa_7068 | D00490 | 0 | hsa_4306 | D05002 |
| 1 | hsa_2099 | D00967 | 0 | hsa_4306 | D00575 | 0 | hsa_6256 | D05002 |
| 1 | hsa_2908 | D00972 | 0 | hsa_2099 | D00689 | 0 | hsa_7068 | D05002 |
| 1 | hsa_2908 | D00973 | 0 | hsa_367 | D00689 | 0 | hsa_7421 | D05002 |
| 1 | hsa_2908 | D00975 | 0 | hsa_5241 | D00751 | 0 | hsa_7421 | D05002 |
| 1 | hsa_2908 | D00976 | 0 | hsa_6256 | D00751 | 0 | hsa_5916 | D05003 |
| 1 | hsa_2908 | D00977 | 0 | hsa_6256 | D00945 | 0 | hsa_4306 | D05020 |
| 1 | hsa_2908 | D00978 | 0 | hsa_5468 | D00946 | 0 | hsa_5241 | D05020 |
| 1 | hsa_2908 | D00979 | 0 | hsa_6256 | D00946 | 0 | hsa_5468 | D05020 |
| 1 | hsa_2908 | D00980 | 0 | hsa_2100 | D00948 | 0 | hsa_5914 | D05020 |
| 1 | hsa_2908 | D00981 | 0 | hsa_4306 | D00948 | 0 | hsa_6257 | D05020 |
| 1 | hsa_2908 | D00982 | 0 | hsa_5916 | D00948 | 0 | hsa_4306 | D05025 |
| 1 | hsa_2908 | D00983 | 0 | hsa_7067 | D00948 | 0 | hsa_5241 | D05025 |
| 1 | hsa_2908 | D00984 | 0 | hsa_5915 | D00949 | 0 | hsa_5916 | D05025 |
| 1 | hsa_2908 | D00985 | 0 | hsa_6257 | D00949 | 0 | hsa_6256 | D05025 |
| 1 | hsa_2908 | D00986 | 0 | hsa_6258 | D00949 | 0 | hsa_6257 | D05025 |
| 1 | hsa_7067 | D01010 | 0 | hsa_7068 | D00949 | 0 | hsa_6258 | D05025 |
| 1 | hsa_7067 | D01011 | 0 | hsa_2099 | D00952 | 0 | hsa_6258 | D05025 |
| 1 | hsa_7421 | D01098 | 0 | hsa_7421 | D00952 | 0 | hsa_6257 | D05030 |
| 1 | hsa_5914 | D01112 | 0 | hsa_9971 | D00952 | 0 | hsa_4306 | D05091 |
| 1 | hsa_5915 | D01112 | 0 | hsa_5467 | D00953 | 0 | hsa_6258 | D05091 |
| 1 | hsa_5916 | D01112 | 0 | hsa_6258 | D00953 | 0 | hsa_6258 | D05091 |
| 1 | hsa_7421 | D01125 | 0 | hsa_2100 | D00955 | 0 | hsa_5467 | D05106 |
| 1 | hsa_367 | D01149 | 0 | hsa_5468 | D00955 | 0 | hsa_5465 | D05116 |
| 1 | hsa_5241 | D01159 | 0 | hsa_6256 | D00955 | 0 | hsa_9971 | D05116 |
| 1 | hsa_5465 | D01208 | 0 | hsa_7067 | D00955 | 0 | hsa_5468 | D05192 |
| 1 | hsa_2908 | D01229 | 0 | hsa_7068 | D00957 | 0 | hsa_5915 | D05192 |
| 1 | hsa_2908 | D01239 | 0 | hsa_5467 | D00958 | 0 | hsa_2099 | D05209 |
| 1 | hsa_2099 | D01265 | 0 | hsa_5468 | D00959 | 0 | hsa_6256 | D05209 |
| 1 | hsa_2908 | D01272 | 0 | hsa_5916 | D00959 | 0 | hsa_5467 | D05601 |
| 1 | hsa_2908 | D01273 | 0 | hsa_2100 | D00966 | 0 | hsa_6256 | D05674 |
| 1 | hsa_5241 | D01299 | 0 | hsa_5468 | D00966 | 0 | hsa_6257 | D05674 |
| 1 | hsa_367 | D01301 | 0 | hsa_5914 | D00966 | 0 | hsa_6257 | D05679 |
| 1 | hsa_2908 | D01327 | 0 | hsa_9971 | D00966 | 0 | hsa_6257 | D05679 |
| 1 | hsa_367 | D01329 | 0 | hsa_367 | D00967 | 0 | hsa_367 | D05719 |
| 1 | hsa_2908 | D01357 | 0 | hsa_5241 | D00967 | 0 | hsa_367 | D05719 |
| 1 | hsa_5465 | D01366 | 0 | hsa_5915 | D00967 | 0 | hsa_5465 | D05719 |
| 1 | hsa_2908 | D01367 | 0 | hsa_6257 | D00967 | 0 | hsa_2099 | D05729 |
| 1 | hsa_367 | D01368 | 0 | hsa_9971 | D00972 | 0 | hsa_5916 | D05729 |
| 1 | hsa_5241 | D01374 | 0 | hsa_4306 | D00976 | 0 | hsa_9971 | D05729 |
| 1 | hsa_367 | D01375 | 0 | hsa_5241 | D00977 | 0 | hsa_367 | D05739 |
| 1 | hsa_2908 | D01402 | 0 | hsa_5468 | D00977 | 0 | hsa_6256 | D05739 |
| 1 | hsa_2099 | D01413 | 0 | hsa_7067 | D00978 | 0 | hsa_7067 | D05739 |
| 1 | hsa_367 | D01414 | 0 | hsa_2100 | D00979 | 0 | hsa_4306 | D05837 |
| 1 | hsa_5914 | D01418 | 0 | hsa_6257 | D00979 | 0 | hsa_5465 | D05837 |
| 1 | hsa_5915 | D01418 | 0 | hsa_6258 | D00979 | 0 | hsa_5468 | D05837 |
| 1 | hsa_5916 | D01418 | 0 | hsa_2100 | D00980 | 0 | hsa_367 | D06085 |
| 1 | hsa_2908 | D01442 | 0 | hsa_4306 | D00980 | 0 | hsa_5916 | D06085 |
| 1 | hsa_2908 | D01464 | 0 | hsa_5241 | D00981 | 0 | hsa_5916 | D06085 |
| 1 | hsa_7421 | D01472 | 0 | hsa_5467 | D00981 | 0 | hsa_7421 | D06085 |
| 1 | hsa_367 | D01476 | 0 | hsa_5468 | D00981 | 0 | hsa_367 | D06086 |
| 1 | hsa_2908 | D01510 | 0 | hsa_2100 | D00982 | 0 | hsa_5465 | D06086 |
| 1 | hsa_5914 | D01516 | 0 | hsa_9971 | D00982 | 0 | hsa_5468 | D06086 |
| 1 | hsa_5915 | D01516 | 0 | hsa_2100 | D00983 | 0 | hsa_5914 | D06086 |
| 1 | hsa_5916 | D01516 | 0 | hsa_6258 | D00983 | 0 | hsa_5916 | D06086 |
| 1 | hsa_7421 | D01518 | 0 | hsa_7068 | D00983 | 0 | hsa_6258 | D06086 |
| 1 | hsa_5241 | D01580 | 0 | hsa_367 | D00984 | 0 | hsa_7068 | D06086 |
| 1 | hsa_2099 | D01602 | 0 | hsa_5241 | D00984 | 0 | hsa_5915 | D06102 |
| 1 | hsa_2908 | D01615 | 0 | hsa_5916 | D00985 | 0 | hsa_6258 | D06102 |
| 1 | hsa_367 | D01616 | 0 | hsa_6257 | D00985 | 0 | hsa_5241 | D06216 |
| 1 | hsa_2099 | D01617 | 0 | hsa_7068 | D00986 | 0 | hsa_5467 | D06216 |
| 1 | hsa_2908 | D01619 | 0 | hsa_7068 | D01010 | 0 | hsa_5468 | D06216 |
| 1 | hsa_5914 | D01621 | 0 | hsa_367 | D01011 | 0 | hsa_4306 | D06245 |
| 1 | hsa_5915 | D01621 | 0 | hsa_6258 | D01011 | 0 | hsa_5468 | D06245 |
| 1 | hsa_5916 | D01621 | 0 | hsa_7421 | D01011 | 0 | hsa_5467 | D06315 |
| 1 | hsa_2908 | D01632 | 0 | hsa_5916 | D01098 | 0 | hsa_5914 | D06315 |
| 1 | hsa_2908 | D01637 | 0 | hsa_7068 | D01098 | 0 | hsa_5916 | D06315 |
| 1 | hsa_2099 | D01639 | 0 | hsa_2100 | D01112 | 0 | hsa_6256 | D06315 |
| 1 | hsa_7421 | D01662 | 0 | hsa_4306 | D01112 | 0 | hsa_6257 | D06315 |
| 1 | hsa_2908 | D01703 | 0 | hsa_6258 | D01112 | 0 | hsa_5916 | D06543 |
| 1 | hsa_2908 | D01708 | 0 | hsa_7421 | D01112 | 0 | hsa_7068 | D06543 |
| 1 | hsa_367 | D01737 | 0 | hsa_5916 | D01125 | 0 | hsa_7421 | D06543 |
| 1 | hsa_2908 | D01743 | 0 | hsa_6257 | D01125 | 0 | hsa_5468 | D06551 |
| 1 | hsa_2908 | D01764 | 0 | hsa_7421 | D01125 | 0 | hsa_5465 | D06876 |
| 1 | hsa_2908 | D01820 | 0 | hsa_2100 | D01149 | 0 | hsa_2100 | D07073 |
| 1 | hsa_2908 | D01825 | 0 | hsa_5241 | D01149 | 0 | hsa_4306 | D07073 |
| 1 | hsa_2908 | D01886 | 0 | hsa_5465 | D01149 | 0 | hsa_5467 | D07073 |
| 1 | hsa_4306 | D01943 | 0 | hsa_5468 | D01149 | 0 | hsa_2099 | D07096 |
| 1 | hsa_2908 | D01948 | 0 | hsa_5916 | D01149 | 0 | hsa_5914 | D07096 |
| 1 | hsa_2099 | D01953 | 0 | hsa_7068 | D01149 | 0 | hsa_9971 | D07096 |
| 1 | hsa_2099 | D01986 | 0 | hsa_5467 | D01159 | 0 | hsa_6258 | D07127 |
| 1 | hsa_2099 | D01989 | 0 | hsa_5915 | D01159 | 0 | hsa_5465 | D07187 |
| 1 | hsa_2908 | D01998 | 0 | hsa_5915 | D01159 | 0 | hsa_5914 | D07187 |
| 1 | hsa_2908 | D02032 | 0 | hsa_5467 | D01208 | 0 | hsa_4306 | D07201 |
| 1 | hsa_2908 | D02156 | 0 | hsa_5467 | D01208 | 0 | hsa_6257 | D07201 |
| 1 | hsa_2908 | D02174 | 0 | hsa_5914 | D01208 | 0 | hsa_6258 | D07201 |
| 1 | hsa_2908 | D02286 | 0 | hsa_9971 | D01229 | 0 | hsa_9971 | D07202 |
| 1 | hsa_2908 | D02287 | 0 | hsa_5465 | D01239 | 0 | hsa_9971 | D07202 |
| 1 | hsa_2908 | D02288 | 0 | hsa_9971 | D01239 | 0 | hsa_5465 | D07203 |
| 1 | hsa_2908 | D02289 | 0 | hsa_2099 | D01265 | 0 | hsa_5467 | D07203 |
| 1 | hsa_2908 | D02591 | 0 | hsa_367 | D01265 | 0 | hsa_7068 | D07203 |
| 1 | hsa_2908 | D02592 | 0 | hsa_6258 | D01265 | 0 | hsa_6258 | D07214 |
| 1 | hsa_5914 | D02754 | 0 | hsa_7068 | D01265 | 0 | hsa_367 | D07220 |
| 1 | hsa_5915 | D02754 | 0 | hsa_5467 | D01272 | 0 | hsa_5468 | D07220 |
| 1 | hsa_5916 | D02754 | 0 | hsa_5916 | D01272 | 0 | hsa_2100 | D07221 |
| 1 | hsa_2099 | D02758 | 0 | hsa_6258 | D01272 | 0 | hsa_5916 | D07221 |
| 1 | hsa_6256 | D02815 | 0 | hsa_7067 | D01272 | 0 | hsa_7068 | D07221 |
| 1 | hsa_6257 | D02815 | 0 | hsa_5465 | D01273 | 0 | hsa_7421 | D07221 |
| 1 | hsa_6258 | D02815 | 0 | hsa_5465 | D01299 | 0 | hsa_2100 | D07222 |
| 1 | hsa_2099 | D02993 | 0 | hsa_5467 | D01299 | 0 | hsa_9971 | D07222 |
| 1 | hsa_5241 | D02996 | 0 | hsa_5914 | D01299 | 0 | hsa_5241 | D07230 |
| 1 | hsa_2099 | D03062 | 0 | hsa_6256 | D01299 | 0 | hsa_6256 | D07230 |
| 1 | hsa_6256 | D03106 | 0 | hsa_5465 | D01301 | 0 | hsa_4306 | D07434 |
| 1 | hsa_6257 | D03106 | 0 | hsa_5467 | D01301 | 0 | hsa_7067 | D07434 |
| 1 | hsa_6258 | D03106 | 0 | hsa_5468 | D01301 | 0 | hsa_2100 | D07456 |
| 1 | hsa_367 | D03144 | 0 | hsa_6256 | D01301 | 0 | hsa_4306 | D07456 |
| 1 | hsa_367 | D03145 | 0 | hsa_2099 | D01327 | 0 | hsa_6256 | D07456 |
| 1 | hsa_2908 | D03301 | 0 | hsa_367 | D01327 | 0 | hsa_2100 | D07495 |
| 1 | hsa_2908 | D03325 | 0 | hsa_5467 | D01327 | 0 | hsa_5468 | D07495 |
| 1 | hsa_4306 | D03363 | 0 | hsa_6256 | D01327 | 0 | hsa_7067 | D07578 |
| 1 | hsa_5468 | D03493 | 0 | hsa_6256 | D01329 | 0 | hsa_5916 | D07670 |
| 1 | hsa_5465 | D03521 | 0 | hsa_6256 | D01329 | 0 | hsa_6256 | D07715 |
| 1 | hsa_2908 | D03541 | 0 | hsa_7067 | D01329 | 0 | hsa_7421 | D07715 |
| 1 | hsa_2908 | D03561 | 0 | hsa_7067 | D01366 | 0 | hsa_2100 | D07717 |
| 1 | hsa_2908 | D03594 | 0 | hsa_367 | D01368 | 0 | hsa_7068 | D07717 |
| 1 | hsa_2908 | D03595 | 0 | hsa_5468 | D01374 | 0 | hsa_2099 | D07719 |
| 1 | hsa_5468 | D03653 | 0 | hsa_6258 | D01374 | 0 | hsa_5467 | D07719 |
| 1 | hsa_2908 | D03671 | 0 | hsa_2100 | D01375 | 0 | hsa_5467 | D07719 |
| 1 | hsa_2908 | D03696 | 0 | hsa_4306 | D01375 | 0 | hsa_5916 | D07719 |
| 1 | hsa_2908 | D03697 | 0 | hsa_5914 | D01375 | 0 | hsa_5468 | D07724 |
| 1 | hsa_4306 | D03698 | 0 | hsa_6257 | D01375 | 0 | hsa_6256 | D07724 |
| 1 | hsa_4306 | D03699 | 0 | hsa_5465 | D01402 | 0 | hsa_7067 | D07724 |
| 1 | hsa_4306 | D03792 | 0 | hsa_5467 | D01402 | 0 | hsa_9971 | D07726 |
| 1 | hsa_5241 | D03799 | 0 | hsa_5467 | D01402 | 0 | hsa_5467 | D07749 |
| 1 | hsa_2908 | D03812 | 0 | hsa_5468 | D01402 | 0 | hsa_5468 | D07792 |
| 1 | hsa_2908 | D03813 | 0 | hsa_5468 | D01413 | 0 | hsa_367 | D07796 |
| 1 | hsa_2099 | D03911 | 0 | hsa_5914 | D01418 | 0 | hsa_5468 | D07796 |
| 1 | hsa_2099 | D03912 | 0 | hsa_5914 | D01418 | 0 | hsa_7421 | D07796 |
| 1 | hsa_4306 | D03917 | 0 | hsa_5915 | D01418 | 0 | hsa_367 | D07797 |
| 1 | hsa_5468 | D03941 | 0 | hsa_6256 | D01418 | 0 | hsa_5916 | D07797 |
| 1 | hsa_2099 | D04041 | 0 | hsa_7067 | D01442 | 0 | hsa_7067 | D07797 |
| 1 | hsa_2099 | D04061 | 0 | hsa_5465 | D01464 | 0 | hsa_7421 | D07797 |
| 1 | hsa_2099 | D04063 | 0 | hsa_7067 | D01464 | 0 | hsa_9971 | D07797 |
| 1 | hsa_2099 | D04064 | 0 | hsa_2099 | D01472 | 0 | hsa_5914 | D07798 |
| 1 | hsa_2099 | D04065 | 0 | hsa_4306 | D01472 | 0 | hsa_5241 | D07800 |
| 1 | hsa_5241 | D04104 | 0 | hsa_5465 | D01472 | 0 | hsa_5467 | D07800 |
| 1 | hsa_5914 | D04162 | 0 | hsa_9971 | D01472 | 0 | hsa_5915 | D07800 |
| 1 | hsa_5915 | D04162 | 0 | hsa_5241 | D01476 | 0 | hsa_5916 | D07800 |
| 1 | hsa_5916 | D04162 | 0 | hsa_5468 | D01476 | 0 | hsa_5914 | D07802 |
| 1 | hsa_2908 | D04208 | 0 | hsa_9971 | D01476 | 0 | hsa_7068 | D07802 |
| 1 | hsa_2908 | D04214 | 0 | hsa_2100 | D01510 | 0 | hsa_5914 | D07826 |
| 1 | hsa_2908 | D04217 | 0 | hsa_5465 | D01510 | 0 | hsa_5914 | D07826 |
| 1 | hsa_2908 | D04218 | 0 | hsa_5467 | D01510 | 0 | hsa_5468 | D07827 |
| 1 | hsa_2908 | D04219 | 0 | hsa_5914 | D01510 | 0 | hsa_9971 | D07827 |
| 1 | hsa_2908 | D04221 | 0 | hsa_6257 | D01510 | 0 | hsa_5914 | D07918 |
| 1 | hsa_2908 | D04227 | 0 | hsa_6258 | D01510 | 0 | hsa_7421 | D07918 |
| 1 | hsa_5241 | D04316 | 0 | hsa_4306 | D01518 | 0 | hsa_5468 | D07919 |
| 1 | hsa_2908 | D04409 | 0 | hsa_2099 | D01580 | 0 | hsa_5468 | D07919 |
| 1 | hsa_2908 | D04467 | 0 | hsa_6257 | D01580 | 0 | hsa_5915 | D07919 |
| 1 | hsa_2099 | D04496 | 0 | hsa_6258 | D01602 | 0 | hsa_5468 | D07920 |
| 1 | hsa_5914 | D04636 | 0 | hsa_9971 | D01602 | 0 | hsa_9971 | D07920 |
| 1 | hsa_5915 | D04636 | 0 | hsa_5241 | D01615 | 0 | hsa_6256 | D07921 |
| 1 | hsa_5916 | D04636 | 0 | hsa_7067 | D01615 | 0 | hsa_5916 | D07928 |
| 1 | hsa_2099 | D04672 | 0 | hsa_7068 | D01615 | 0 | hsa_7067 | D07928 |
| 1 | hsa_5241 | D04885 | 0 | hsa_5468 | D01616 | 0 | hsa_7067 | D07928 |
| 1 | hsa_367 | D04947 | 0 | hsa_6257 | D01617 | 0 | hsa_2099 | D07939 |
| 1 | hsa_2908 | D05000 | 0 | hsa_6258 | D01617 | 0 | hsa_5241 | D07939 |
| 1 | hsa_2908 | D05001 | 0 | hsa_7068 | D01617 | 0 | hsa_7067 | D07967 |
| 1 | hsa_2908 | D05002 | 0 | hsa_7068 | D01621 | 0 | hsa_5916 | D07972 |
| 1 | hsa_5241 | D05003 | 0 | hsa_5241 | D01632 | 0 | hsa_6256 | D07972 |
| 1 | hsa_4306 | D05020 | 0 | hsa_5915 | D01632 | 0 | hsa_2100 | D07973 |
| 1 | hsa_367 | D05025 | 0 | hsa_5915 | D01637 | 0 | hsa_5916 | D07973 |
| 1 | hsa_5468 | D05030 | 0 | hsa_5916 | D01639 | 0 | hsa_7421 | D07973 |
| 1 | hsa_5465 | D05091 | 0 | hsa_6258 | D01662 | 0 | hsa_2099 | D07980 |
| 1 | hsa_2099 | D05106 | 0 | hsa_5467 | D01703 | 0 | hsa_2100 | D07980 |
| 1 | hsa_367 | D05116 | 0 | hsa_5914 | D01703 | 0 | hsa_2100 | D07980 |
| 1 | hsa_5468 | D05150 | 0 | hsa_6258 | D01703 | 0 | hsa_5915 | D07980 |
| 1 | hsa_2099 | D05192 | 0 | hsa_9971 | D01703 | 0 | hsa_2100 | D07981 |
| 1 | hsa_5241 | D05209 | 0 | hsa_7421 | D01708 | 0 | hsa_367 | D08036 |
| 1 | hsa_2908 | D05601 | 0 | hsa_2099 | D01737 | 0 | hsa_367 | D08052 |
| 1 | hsa_4306 | D05640 | 0 | hsa_2100 | D01737 | 0 | hsa_4306 | D08053 |
| 1 | hsa_367 | D05674 | 0 | hsa_6257 | D01737 | 0 | hsa_5468 | D08053 |
| 1 | hsa_5241 | D05679 | 0 | hsa_7421 | D01737 | 0 | hsa_5241 | D08125 |
| 1 | hsa_2908 | D05719 | 0 | hsa_5241 | D01743 | 0 | hsa_5916 | D08128 |
| 1 | hsa_2908 | D05729 | 0 | hsa_5914 | D01743 | 0 | hsa_9971 | D08128 |
| 1 | hsa_5468 | D05739 | 0 | hsa_7067 | D01743 | 0 | hsa_5468 | D08147 |
| 1 | hsa_367 | D05837 | 0 | hsa_7421 | D01743 | 0 | hsa_5915 | D08147 |
| 1 | hsa_367 | D06085 | 0 | hsa_2099 | D01764 | 0 | hsa_6257 | D08147 |
| 1 | hsa_367 | D06086 | 0 | hsa_5241 | D01764 | 0 | hsa_7067 | D08147 |
| 1 | hsa_367 | D06087 | 0 | hsa_5468 | D01764 | 0 | hsa_9971 | D08166 |
| 1 | hsa_5465 | D06102 | 0 | hsa_2099 | D01820 | 0 | hsa_5465 | D08167 |
| 1 | hsa_2908 | D06171 | 0 | hsa_5914 | D01820 | 0 | hsa_9971 | D08167 |
| 1 | hsa_2908 | D06216 | 0 | hsa_7067 | D01820 | 0 | hsa_5241 | D08193 |
| 1 | hsa_2099 | D06245 | 0 | hsa_7421 | D01820 | 0 | hsa_5467 | D08193 |
| 1 | hsa_2908 | D06315 | 0 | hsa_5241 | D01825 | 0 | hsa_6257 | D08193 |
| 1 | hsa_5914 | D06543 | 0 | hsa_5467 | D01825 | 0 | hsa_2100 | D08196 |
| 1 | hsa_5915 | D06543 | 0 | hsa_5915 | D01825 | 0 | hsa_367 | D08196 |
| 1 | hsa_5916 | D06543 | 0 | hsa_6256 | D01825 | 0 | hsa_5914 | D08197 |
| 1 | hsa_2099 | D06551 | 0 | hsa_7067 | D01825 | 0 | hsa_7068 | D08197 |
| 1 | hsa_2908 | D06673 | 0 | hsa_9971 | D01825 | 0 | hsa_367 | D08227 |
| 1 | hsa_2908 | D06876 | 0 | hsa_9971 | D01825 | 0 | hsa_367 | D08227 |
| 1 | hsa_2908 | D07073 | 0 | hsa_2100 | D01886 | 0 | hsa_5915 | D08227 |
| 1 | hsa_5241 | D07096 | 0 | hsa_4306 | D01886 | 0 | hsa_5915 | D08227 |
| 1 | hsa_2908 | D07116 | 0 | hsa_5915 | D01886 | 0 | hsa_5915 | D08227 |
| 1 | hsa_367 | D07127 | 0 | hsa_6258 | D01886 | 0 | hsa_7068 | D08227 |
| 1 | hsa_5465 | D07187 | 0 | hsa_2099 | D01943 | 0 | hsa_4306 | D08250 |
| 1 | hsa_2908 | D07201 | 0 | hsa_2099 | D01943 | 0 | hsa_7067 | D08250 |
| 1 | hsa_2908 | D07202 | 0 | hsa_367 | D01943 | 0 | hsa_2100 | D08251 |
| 1 | hsa_2908 | D07203 | 0 | hsa_6256 | D01943 | 0 | hsa_7067 | D08251 |
| 1 | hsa_7067 | D07214 | 0 | hsa_9971 | D01943 | 0 | hsa_2100 | D08277 |
| 1 | hsa_5241 | D07220 | 0 | hsa_5465 | D01948 | 0 | hsa_5468 | D08277 |
| 1 | hsa_2099 | D07221 | 0 | hsa_5915 | D01953 | 0 | hsa_5468 | D08277 |
| 1 | hsa_5241 | D07222 | 0 | hsa_6256 | D01953 | 0 | hsa_367 | D08281 |
| 1 | hsa_5241 | D07223 | 0 | hsa_6256 | D01986 | 0 | hsa_5241 | D08281 |
| 1 | hsa_2908 | D07230 | 0 | hsa_7067 | D01986 | 0 | hsa_7421 | D08281 |
| 1 | hsa_2099 | D07434 | 0 | hsa_7068 | D01986 | 0 | hsa_2100 | D08285 |
| 1 | hsa_367 | D07456 | 0 | hsa_5916 | D01989 | 0 | hsa_5915 | D08285 |
| 1 | hsa_2908 | D07495 | 0 | hsa_6256 | D01989 | 0 | hsa_7421 | D08285 |
| 1 | hsa_367 | D07536 | 0 | hsa_6257 | D01989 | 0 | hsa_5241 | D08378 |
| 1 | hsa_7421 | D07578 | 0 | hsa_2099 | D02156 | 0 | hsa_6256 | D08378 |
| 1 | hsa_5241 | D07670 | 0 | hsa_6256 | D02174 | 0 | hsa_7067 | D08378 |
| 1 | hsa_2908 | D07715 | 0 | hsa_7421 | D02174 | 0 | hsa_367 | D08409 |
| 1 | hsa_2908 | D07717 | 0 | hsa_7068 | D02286 | 0 | hsa_5914 | D08412 |
| 1 | hsa_2908 | D07719 | 0 | hsa_4306 | D02287 | 0 | hsa_6257 | D08412 |
| 1 | hsa_5465 | D07724 | 0 | hsa_5915 | D02287 | 0 | hsa_7067 | D08412 |
| 1 | hsa_2099 | D07726 | 0 | hsa_7421 | D02288 | 0 | hsa_4306 | D08413 |
| 1 | hsa_2908 | D07749 | 0 | hsa_4306 | D02289 | 0 | hsa_7067 | D08413 |
| 1 | hsa_367 | D07766 | 0 | hsa_7067 | D02289 | 0 | hsa_5468 | D08414 |
| 1 | hsa_4306 | D07792 | 0 | hsa_2100 | D02591 | 0 | hsa_7421 | D08415 |
| 1 | hsa_2908 | D07796 | 0 | hsa_7067 | D02591 | 0 | hsa_2099 | D08416 |
| 1 | hsa_2908 | D07797 | 0 | hsa_7068 | D02591 | 0 | hsa_5914 | D08416 |
| 1 | hsa_2908 | D07798 | 0 | hsa_5468 | D02592 | 0 | hsa_2099 | D08429 |
| 1 | hsa_2908 | D07799 | 0 | hsa_7068 | D02592 | 0 | hsa_5465 | D08429 |
| 1 | hsa_2908 | D07800 | 0 | hsa_4306 | D02754 | 0 | hsa_7421 | D08431 |
| 1 | hsa_2908 | D07801 | 0 | hsa_4306 | D02754 | 0 | hsa_2099 | D08465 |
| 1 | hsa_2908 | D07802 | 0 | hsa_5914 | D02754 | 0 | hsa_2099 | D08465 |
| 1 | hsa_2099 | D07826 | 0 | hsa_5468 | D02758 | 0 | hsa_6258 | D08465 |
| 1 | hsa_2908 | D07827 | 0 | hsa_5468 | D02815 | 0 | hsa_6256 | D08476 |
| 1 | hsa_2099 | D07918 | 0 | hsa_6258 | D02815 | 0 | hsa_6257 | D08476 |
| 1 | hsa_2099 | D07919 | 0 | hsa_7421 | D02815 | 0 | hsa_2100 | D08477 |
| 1 | hsa_2099 | D07920 | 0 | hsa_5914 | D02993 | 0 | hsa_5241 | D08491 |
| 1 | hsa_2099 | D07921 | 0 | hsa_2099 | D02996 | 0 | hsa_5241 | D08491 |
| 1 | hsa_2099 | D07928 | 0 | hsa_367 | D02996 | 0 | hsa_6256 | D08491 |
| 1 | hsa_5241 | D07939 | 0 | hsa_5465 | D02996 | 0 | hsa_5468 | D08554 |
| 1 | hsa_2908 | D07967 | 0 | hsa_9971 | D03062 | 0 | hsa_5916 | D08554 |
| 1 | hsa_2908 | D07972 | 0 | hsa_5241 | D03106 | 0 | hsa_7067 | D08559 |
| 1 | hsa_2908 | D07973 | 0 | hsa_5915 | D03144 | 0 | hsa_6257 | D08573 |
| 1 | hsa_2908 | D07980 | 0 | hsa_6257 | D03144 | 0 | hsa_5914 | D08574 |
| 1 | hsa_2908 | D07981 | 0 | hsa_6257 | D03145 | 0 | hsa_7068 | D08574 |
| 1 | hsa_2908 | D08036 | 0 | hsa_7068 | D03145 | 0 | hsa_367 | D08610 |
| 1 | hsa_5241 | D08052 | 0 | hsa_2100 | D03301 | 0 | hsa_367 | D08610 |
| 1 | hsa_5241 | D08053 | 0 | hsa_5467 | D03301 | 0 | hsa_6258 | D08610 |
| 1 | hsa_7067 | D08125 | 0 | hsa_7068 | D03301 | 0 | hsa_9971 | D08610 |
| 1 | hsa_7067 | D08128 | 0 | hsa_5468 | D03325 | 0 | hsa_5915 | D08620 |
| 1 | hsa_7067 | D08129 | 0 | hsa_6256 | D03325 | 0 | hsa_6258 | D08620 |
| 1 | hsa_2908 | D08147 | 0 | hsa_2099 | D03363 | 0 | hsa_2100 | D08660 |
| 1 | hsa_5241 | D08166 | 0 | hsa_5467 | D03363 | 0 | hsa_5915 | D08660 |
| 1 | hsa_5241 | D08167 | 0 | hsa_6256 | D03363 | 0 | hsa_7421 | D08660 |
| 1 | hsa_367 | D08193 | 0 | hsa_6257 | D03363 | 0 | hsa_4306 | D08845 |
| 1 | hsa_367 | D08196 | 0 | hsa_2100 | D03493 | 0 | hsa_5914 | D08845 |
| 1 | hsa_367 | D08197 | 0 | hsa_7068 | D03521 | 0 | hsa_5241 | D08868 |
| 1 | hsa_2908 | D08227 | 0 | hsa_7421 | D03521 | 0 | hsa_5465 | D08890 |
| 1 | hsa_367 | D08250 | 0 | hsa_2100 | D03561 | 0 | hsa_5914 | D08890 |
| 1 | hsa_367 | D08251 | 0 | hsa_5468 | D03561 | 0 | hsa_5915 | D08949 |
| 1 | hsa_9971 | D08277 | 0 | hsa_367 | D03594 | 0 | hsa_6258 | D08949 |
| 1 | hsa_5241 | D08281 | 0 | hsa_6257 | D03594 | 0 | hsa_4306 | D08958 |
| 1 | hsa_5241 | D08285 | 0 | hsa_6258 | D03594 | 0 | hsa_5915 | D08958 |
| 1 | hsa_2099 | D08301 | 0 | hsa_7068 | D03595 | 0 | hsa_7421 | D08958 |
| 1 | hsa_5468 | D08378 | 0 | hsa_7421 | D03696 | 0 | hsa_2100 | D09350 |
| 1 | hsa_367 | D08409 | 0 | hsa_7421 | D03698 | 0 | hsa_2100 | D09350 |
| 1 | hsa_2908 | D08412 | 0 | hsa_5467 | D03699 | 0 | hsa_367 | D09350 |
| 1 | hsa_2908 | D08413 | 0 | hsa_5468 | D03699 | 0 | hsa_5914 | D09360 |
| 1 | hsa_2908 | D08414 | 0 | hsa_2100 | D03792 | 0 | hsa_6256 | D09360 |
| 1 | hsa_2908 | D08415 | 0 | hsa_5465 | D03792 | 0 | hsa_5914 | D09365 |
| 1 | hsa_2908 | D08416 | 0 | hsa_7067 | D03792 | 0 | hsa_6258 | D09365 |
| 1 | hsa_5241 | D08429 | 0 | hsa_367 | D03799 | 0 | hsa_7068 | D09365 |
| 1 | hsa_5241 | D08431 | 0 | hsa_367 | D03799 | 0 | hsa_9971 | D09365 |
| 1 | hsa_2099 | D08465 | 0 | hsa_7067 | D03799 | 0 | hsa_2099 | D09381 |
| 1 | hsa_2908 | D08476 | 0 | hsa_5241 | D03812 | 0 | hsa_5467 | D09381 |
| 1 | hsa_5914 | D08477 | 0 | hsa_6258 | D03812 | 0 | hsa_5916 | D09381 |
| 1 | hsa_5915 | D08477 | 0 | hsa_2100 | D03813 | 0 | hsa_2099 | D09567 |
| 1 | hsa_5916 | D08477 | 0 | hsa_5916 | D03813 | 0 | hsa_5468 | D09567 |
| 1 | hsa_5468 | D08491 | 0 | hsa_7421 | D03813 | 0 | hsa_6256 | D09567 |
| 1 | hsa_7421 | D08554 | 0 | hsa_9971 | D03813 | 0 | hsa_2100 | D09571 |
| 1 | hsa_2099 | D08559 | 0 | hsa_5915 | D03911 | 0 | hsa_6258 | D09571 |
| 1 | hsa_367 | D08573 | 0 | hsa_5467 | D03912 | 0 | hsa_7421 | D09571 |
| 1 | hsa_367 | D08574 | 0 | hsa_5914 | D03912 | 0 | hsa_9971 | D09579 |
| 1 | hsa_2908 | D08610 | 0 | hsa_5914 | D03912 | 0 | hsa_9971 | D09579 |
| 1 | hsa_2099 | D08620 | 0 | hsa_5914 | D03912 | 0 | hsa_7068 | D09687 |
| 1 | hsa_2908 | D08660 | 0 | hsa_5915 | D03912 | 0 | hsa_5468 | D09796 |
| 1 | hsa_5465 | D08845 | 0 | hsa_5916 | D03912 | 0 | hsa_5916 | D09796 |
| 1 | hsa_7421 | D08868 | 0 | hsa_2100 | D03917 | 0 | hsa_6258 | D09796 |
| 1 | hsa_5465 | D08890 | 0 | hsa_367 | D03941 | 0 | hsa_4306 | D09899 |
| 1 | hsa_2099 | D08910 | 0 | hsa_6257 | D03941 | 0 | hsa_5915 | D09899 |
| 1 | hsa_5465 | D08949 | 0 | hsa_5241 | D04041 | 0 | hsa_5465 | D10008 |
| 1 | hsa_2099 | D08958 | 0 | hsa_6258 | D04041 | 0 | hsa_4306 | D10016 |
| 1 | hsa_5465 | D09350 | 0 | hsa_7068 | D04041 | 0 | hsa_6257 | D10016 |
| 1 | hsa_5467 | D09350 | 0 | hsa_367 | D04061 | 0 | hsa_5914 | D10136 |
| 1 | hsa_5468 | D09350 | 0 | hsa_5915 | D04061 | 0 | hsa_2099 | D10198 |
| 1 | hsa_9971 | D09360 | 0 | hsa_7068 | D04061 | 0 | hsa_5467 | D10198 |
| 1 | hsa_5916 | D09365 | 0 | hsa_2099 | D04063 | 0 | hsa_6256 | D10198 |
| 1 | hsa_7068 | D09381 | 0 | hsa_5468 | D04064 | 0 | hsa_6257 | D10198 |
| 1 | hsa_5241 | D09567 | 0 | hsa_6257 | D04064 | 0 | hsa_6258 | D10198 |
| 1 | hsa_5241 | D09571 | 0 | hsa_2099 | D04065 | 0 | hsa_2099 | D10218 |
| 1 | hsa_5468 | D09579 | 0 | hsa_5914 | D04065 | 0 | hsa_5465 | D10218 |
| 1 | hsa_5241 | D09687 | 0 | hsa_5914 | D04065 | 0 | hsa_5914 | D10218 |
| 1 | hsa_2908 | D09796 | 0 | hsa_6258 | D04065 | 0 | hsa_9971 | D10218 |
| 1 | hsa_2100 | D09899 | 0 | hsa_7421 | D04065 | 0 | hsa_5914 | D10221 |
| 1 | hsa_2099 | D10008 | 0 | hsa_367 | D04104 | 0 | hsa_6258 | D10221 |
| 1 | hsa_5241 | D10016 | 0 | hsa_5241 | D04104 | 0 | hsa_7068 | D10528 |
| 1 | hsa_2908 | D10136 | 0 | hsa_5468 | D04104 | 0 | hsa_2100 | D10606 |
| 1 | hsa_7421 | D10198 | 0 | hsa_5916 | D04104 | 0 | hsa_5915 | D10606 |
| 1 | hsa_367 | D10218 | 0 | hsa_7067 | D04104 | 0 | hsa_9971 | D10606 |
| 1 | hsa_367 | D10221 | 0 | hsa_5468 | D04162 | 0 | hsa_4306 | D10633 |
| 1 | hsa_4306 | D10528 | 0 | hsa_5914 | D04162 | 0 | hsa_7068 | D10633 |
| 1 | hsa_7421 | D10565 | 0 | hsa_6256 | D04162 | 0 | hsa_4306 | D10711 |
| 1 | hsa_2099 | D10606 | 0 | hsa_2099 | D04208 | 0 | hsa_5465 | D10711 |
| 1 | hsa_2908 | D10617 | 0 | hsa_6256 | D04208 | 0 | hsa_5915 | D10711 |
| 1 | hsa_4306 | D10633 | 0 | hsa_9971 | D04208 | 0 | hsa_6257 | D10711 |
| 1 | hsa_5465 | D10711 | 0 | hsa_5467 | D04214 | 0 | hsa_7421 | D10711 |
