# Supplementary material for: Predicting drug-target interactions from drug structure and protein sequence using novel convolutional neural networks
Source: BMC Bioinformatics. 2019 Dec 24;20(Suppl 25):689. doi: 10.1186/s12859-019-3263-x (PMC6929541; doi:10.1186/s12859-019-3263-x)
Supplement: Supplementary file 2 — Additional file 2 In this file, to obtain the optimal learning rate of our model, a series of different learning rates are explored on each types of protein families of both Dataset1 and Dataset2. [file 12859_2019_3263_MOESM2_ESM.docx]

**Additional file 2: Is learning rate 0.001 the optimal for our model?**

In an effort to provide the optimal learning rate of our model, a series of different learning rates are explored on each types of protein families of both *Dataset1* and *Dataset2*. The detailed results are shown in the Table 1 and Table 2.

**Table 1 The prediction results for all classes of protein families on both *Dataset1* and *Dataset2* with different learning rates.**

|  |  | ***Dataset1*** | | | | | ***Dataset2*** | | | | |
| --- | --- | --- | --- | --- | --- | --- | --- | --- | --- | --- | --- |
|  |  | **Acc** | **Sen** | **Pre** | **F1** | **AUC** | **Acc** | **Sen** | **Pre** | **F1** | **AUC** |
| **Enzymes** | **lr=0.01** | - | - | - | - | - | - | - | - | - | - |
|  | **lr=0.001** | 0.943 | 0.927 | 0.903 | 0.915 | 0.985 | 0.920 | 0.881 | 0.88 | 0.881 | 0.973 |
|  | **lr=0.0001** | 0.904 | 0.848 | 0.862 | 0.855 | 0.960 | 0.881 | 0.806 | 0.83 | 0.818 | 0.936 |
| **Ion Channels** | **lr=0.01** | - | - | - | - | - | - | - | - | - | - |
|  | **lr=0.001** | 0.919 | 0.894 | 0.867 | 0.881 | 0.97 | 0.900 | 0.948 | 0.792 | 0.863 | 0.949 |
|  | **lr=0.0001** | 0.892 | 0.864 | 0.821 | 0.842 | 0.952 | 0.891 | 0.909 | 0.794 | 0.848 | 0.943 |
| **GPCRs** | **lr=0.01** | - | - | - | - | - | - | - | - | - | - |
|  | **lr=0.001** | 0.884 | 0.818 | 0.831 | 0.824 | 0.945 | 0.920 | 0.899 | 0.866 | 0.882 | 0.968 |
|  | **lr=0.0001** | 0.85 | 0.794 | 0.765 | 0.779 | 0.916 | 0.883 | 0.853 | 0.806 | 0.829 | 0.942 |
| **Nuclear receptors** | **lr=0.01** | - | - | - | - | - | - | - | - | - | - |
|  | **lr=0.001** | 0.884 | 0.872 | 0.798 | 0.833 | 0.936 | 0.907 | 0.891 | 0.841 | 0.865 | 0.966 |
|  | **lr=0.0001** | 0.806 | 0.628 | 0.75 | 0.684 | 0.863 | 0.903 | 0.894 | 0.828 | 0.86 | 0.957 |

- means the loss value is a null value so that these metrics are unable to be calculated

**Table 2 The prediction results for all classes of protein families on both *Dataset1* and *Dataset2* with learning rates around 0.001.**

|  |  | ***Dataset1*** | | | | | ***Dataset2*** | | | | |
| --- | --- | --- | --- | --- | --- | --- | --- | --- | --- | --- | --- |
|  |  | **Acc** | **Sen** | **Pre** | **F1** | **AUC** | **Acc** | **Sen** | **Pre** | **F1** | **AUC** |
| **Enzymes** | **lr=0.0008** | 0.925 | 0.889 | 0.887 | 0.888 | 0.976 | 0.914 | 0.857 | 0.872 | 0.864 | 0.969 |
|  | **lr=0.0009** | 0.941 | 0.917 | 0.913 | 0.915 | 0.984 | 0.921 | 0.881 | 0.865 | 0.873 | 0.974 |
|  | **lr=0.001** | 0.943 | 0.927 | 0.903 | 0.915 | 0.985 | 0.920 | 0.881 | 0.88 | 0.880 | 0.973 |
|  | **lr=0.0011** | 0.935 | 0.891 | 0.909 | 0.900 | 0.976 | 0.915 | 0.864 | 0.881 | 0.872 | 0.966 |
|  | **lr=0.0012** | 0.934 | 0.885 | 0.914 | 0.899 | 0.980 | 0.914 | 0.867 | 0.874 | 0.870 | 0.965 |
| **Ion Channels** | **lr=0.0008** | 0.913 | 0.882 | 0.843 | 0.862 | 0.967 | 0.899 | 0.946 | 0.793 | 0.863 | 0.947 |
|  | **lr=0.0009** | 0.918 | 0.872 | 0.880 | 0.876 | 0.970 | 0.894 | 0.940 | 0.794 | 0.861 | 0.941 |
|  | **lr=0.001** | 0.919 | 0.894 | 0.867 | 0.881 | 0.970 | 0.900 | 0.948 | 0.792 | 0.863 | 0.949 |
|  | **lr=0.0011** | 0.917 | 0.904 | 0.856 | 0.879 | 0.971 | 0.898 | 0.947 | 0.790 | 0.861 | 0.946 |
|  | **lr=0.0012** | 0.921 | 0.883 | 0.880 | 0.881 | 0.973 | 0.898 | 0.937 | 0.795 | 0.860 | 0.947 |
| **GPCRs** | **lr=0.0008** | 0.881 | 0.863 | 0.797 | 0.829 | 0.945 | 0.911 | 0.884 | 0.854 | 0.869 | 0.963 |
|  | **lr=0.0009** | 0.876 | 0.868 | 0.783 | 0.823 | 0.943 | 0.914 | 0.878 | 0.866 | 0.872 | 0.966 |
|  | **lr=0.001** | 0.884 | 0.818 | 0.831 | 0.824 | 0.945 | 0.920 | 0.899 | 0.866 | 0.882 | 0.968 |
|  | **lr=0.0011** | 0.880 | 0.808 | 0.812 | 0.810 | 0.941 | 0.915 | 0.877 | 0.869 | 0.873 | 0.967 |
|  | **lr=0.0012** | 0.882 | 0.889 | 0.785 | 0.834 | 0.944 | 0.914 | 0.898 | 0.852 | 0.875 | 0.967 |
| **Nuclear Receptors** | **lr=0.0008** | 0.872 | 0.872 | 0.773 | 0.82 | 0.935 | 0.911 | 0.885 | 0.854 | 0.869 | 0.966 |
|  | **lr=0.0009** | 0.872 | 0.86 | 0.779 | 0.818 | 0.928 | 0.909 | 0.885 | 0.849 | 0.867 | 0.967 |
|  | **lr=0.001** | 0.884 | 0.872 | 0.798 | 0.833 | 0.936 | 0.907 | 0.891 | 0.841 | 0.865 | 0.966 |
|  | **lr=0.0011** | 0.864 | 0.756 | 0.823 | 0.788 | 0.929 | 0.905 | 0.86 | 0.857 | 0.858 | 0.966 |
|  | **lr=0.0012** | 0.880 | 0.826 | 0.816 | 0.821 | 0.935 | 0.910 | 0.897 | 0.844 | 0.869 | 0.967 |
